# Association Between COVID-19 Infection and Thyroid Cancer Development: A Retrospective Cohort Study Using the TriNetX Database

**DOI:** 10.3390/biomedicines13081933

**Published:** 2025-08-08

**Authors:** Hsin-Yi Wang, Yi-Ching Lin, Jing-Uei Hou, Chih-Hao Chao, Shih-Chuan Tsai

**Affiliations:** 1 Department of Nuclear Medicine, Taichung Veterans General Hospital, Taichung City 407219, Taiwan; hywang@vghtc.gov.tw (H.-Y.W.); dianayjlin@gmail.com (Y.-C.L.); hou_jerry15@yahoo.com.tw (J.-U.H.); 2 National Defense Medical Center, School of Medicine, Taipei City 11490, Taiwan; 3 Department of Public Health, China Medical University, Taichung City 406040, Taiwan; 4 Division of Chest Medicine, Department of Internal Medicine, Yuanlin Christian Hospital, Changhua County 510012, Taiwan; 5 Department of Medical Imaging and Radiological Technology, Institute of Radiological Science, Central Taiwan University of Science and Technology, Taichung City 406053, Taiwan

**Keywords:** COVID-19, hyperthyroidism, hypothyroidism thyroid cancer

## Abstract

**Background**: Coronavirus Disease 2019 has been associated with dysfunction in multiple endocrine organs, including the thyroid gland. While evidence suggests SARS-CoV-2 may influence thyroid function and promote oncogenesis through inflammation and cytokine storms, its role in thyroid cancer remains unclear. This study investigates whether COVID-19 is associated with an increased risk of thyroid cancer development. **Methods**: We conducted a retrospective cohort study using the TriNetX global federated health research database, encompassing data from 151 healthcare organizations. Adult patients with confirmed COVID-19 between 1 December 2019 and 31 December 2023, were included and compared to a matched cohort without COVID-19. Patients with prior thyroid cancer history or who had received COVID-19 vaccination were excluded in both groups. Propensity score matching (1:1) was performed for age, gender, and overweight/obesity status. The primary outcome was that new-onset thyroid cancer was diagnosed at least one year after COVID-19 diagnosis. Hazard ratios were calculated using Cox proportional hazards models, and subgroup analyses were performed based on age, gender, thyroid function status and treatment modalities. **Results**: After matching, a significantly higher thyroid cancer incidence was observed between the post-COVID and non-COVID groups. Subgroup analysis revealed a significantly higher risk of thyroid cancer development following COVID-19 diagnosis in patients who developed hyperthyroidism (HR 2.14, 95% CI: 1.04–4.46) or hypothyroid-ism (HR 1.83, 95% CI: 1.12–2.97) compared with the non-COVID population. Male patients also exhibited a higher risk of thyroid cancer after COVID-19 (HR 1.22, 95% CI 1.02–1.46). For patients with hyperthyroidism or hypothyroidism, those who had prior COVID-19 exhibited a relatively higher risk of developing thyroid cancer than those without a history of COVID-19 (HR 4.387, 95% CI: 2.08–9.24 for hyperthyroidism; HR 2.58, 95% CI: 1.58–4.22 for hypothyroidism). **Conclusions**: Patients with COVID-19 exhibited an increase in thyroid cancer risk, with specific subgroups—male adults and those with post-infectious thyroid dysfunction—also exhibiting increased risk. These findings suggest a potential relationship between SARS-CoV-2 and thyroid oncogenesis, warranting further prospective research.

## 1. Introduction

Coronavirus Disease 2019 (COVID-19), caused by the novel coronavirus SARS-CoV-2, has had a profound impact on global health and economies [1]. As of April 2025, over 770 million confirmed infections and 7 million deaths have been reported worldwide [2]. COVID-19 presents with a wide spectrum of clinical manifestations, ranging from asymptomatic cases to severe respiratory failure and multi-organ dysfunction [3]. In addition to the direct effect of SARS-CoV-2 viral invasion, indirect effects—such as abnormal inflammatory responses and cytokine storms—can also impair various endocrine organs, including the pancreas, adrenal glands, pituitary glands, and thyroid gland [4].

Thyroid cancer is the most prevalent endocrine malignancy [5], and its incidence has been rising rapidly worldwide in recent years [6]. It is classified into differentiated thyroid cancer (including papillary and follicular thyroid carcinoma), medullary thyroid cancer, and anaplastic thyroid cancer [7]. Among these, papillary thyroid carcinoma is the most common type, accounting for approximately 90% of cases [8,9]. Established risk factors for thyroid cancer include childhood exposure to ionizing radiation and obesity. Other potential risk factors include endocrine-disrupting chemicals and thyroid dysfunction [6]. The incidence of thyroid cancer is higher in women than in men [9]. In the United States, thyroid cancer ranks as the 13th most commonly diagnosed cancer overall and the 6th most common among women [10]. Prognosis is generally favorable; the 5-year survival rate exceeds 90% for localized or regionally metastatic disease but declines to approximately 54.9% in cases with distant metastases [10].

The thyroid gland produces thyroid hormones, including the prohormone tetraiodothyronine (T4) and the active hormone triiodothyronine (T3). T3 regulates basal metabolic rate by influencing the central nervous system, respiratory system, cardiovascular system, and skeletal muscle [11]. SARS-CoV-2 infection can disrupt thyroid function, potentially leading to conditions such as thyrotoxicosis, hypothyroidism, and the nonthyroidal illness syndrome [12].

SARS-CoV-2 RNA has been detected in thyroid tissue [13], suggesting direct viral invasion. SARS-CoV-2 enters target cells through angiotensin-converting enzyme II (ACE2) receptors, which are highly expressed in endocrine tissues, including the thyroid and pituitary glands [14,15]. The binding of SARS-CoV-2 to ACE2 receptors downregulates the angiotensin I receptor, leading to dysregulation of the renin–angiotensin–aldosterone system. This, in turn, results in inflammation, fibrosis, vasoconstriction, oxidation, and increased capillary permeability [16,17,18,19], all of which may contribute to cancer development. Additionally, SARS-CoV-2 infection induces profound inflammation and cytokine storms [20], which could promote cancer stem cell growth in target organs and are associated with the development of thyroid cancer [21,22]. The inflammation and oxidative stress induced by SARS-CoV-2 may also play a role in the development and progression of thyroid cancer [23]. Infection with SARS-CoV-2 may also induce the expression of nonstructural protein (nsp) 15, which affects the function of the tumor suppressor retinoblastoma protein [24,25], and nsp 3, which degrades the tumor suppressor gene P53 [26,27]. This pathway is considered one of the most likely mechanisms by which SARS-CoV-2 could promote cancer development [28].

However, the literature on the relationship between COVID-19 and thyroid cancer development remains limited, and no consensus has been established. To address this gap, we conducted a retrospective cohort study to investigate whether COVID-19 contributes to the development of thyroid cancer.

## 2. Materials and Methods

### 2.1. Setting

In this retrospective cohort study, we utilized data from the Global Collaborative Network within the TriNetX database, which includes information from 151 healthcare organizations (HCOs). TriNetX is a global federated health research network that provides access to electronic medical records, encompassing diagnoses, procedures, medications, laboratory results, and genomic data from large HCOs.

The TriNetX platform complies with the Health Insurance Portability & Accountability Act and the General Data Protection Regulation. The Western Institutional Review Board granted TriNetX a waiver of informed consent, as the platform only aggregates counts and statistical summaries of deidentified information.

### 2.2. Cohort

We included adult patients (aged ≥18 years) with a positive SARS-CoV-2 PCR test (Table 1) or a COVID-19 diagnosis (ICD-10 codes: U07.1, U07.2, or J12.82) recorded in the TriNetX database during the study period. Patients with a prior history of thyroid cancer (ICD-10 codes: C73 or Z85.850) or those who had received any COVID-19 vaccine (Table 2) were excluded. For the subgroup analysis on abnormal thyroid function, we also excluded patients with a history of hyperthyroidism (ICD-10 codes: E05, E05.8, E05.3, E05.31, and E06) or hypothyroidism (ICD10 codes: E03.3 and E03.8). The index date was defined as the date of the COVID-19 diagnosis.

**Figure 1:**
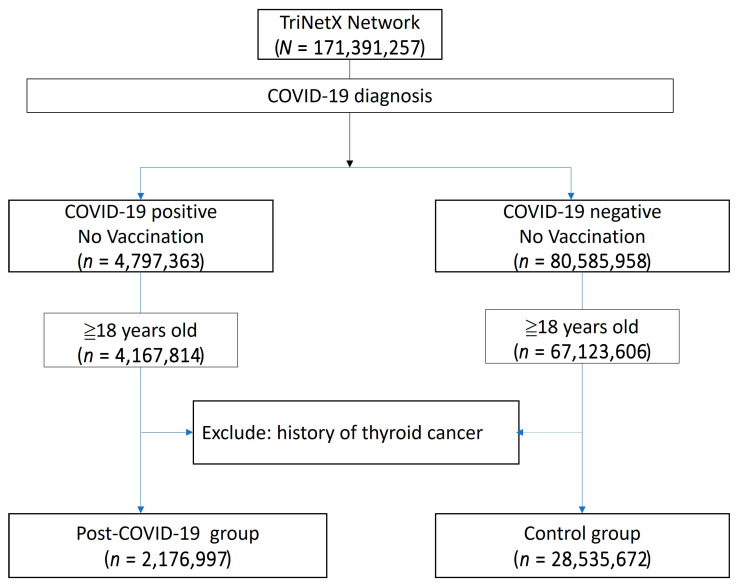
Flowchart illustrating the construction of the study cohort from the TriNetX database.

The study population was compared to a control group comprising individuals without a COVID-19 diagnosis, no history of COVID-19 vaccination, and no prior history of thyroid cancer. The primary outcome was a new diagnosis of thyroid cancer occurring at least one year after the COVID-19 diagnosis. A flowchart illustrating the cohort construction, including participants enrolled between 1 December 2019 and 31 December 2023, is presented in Figure 1. Detailed query criteria are provided in Supplementary File S1.

A subgroup analysis was performed to compare the risk of thyroid cancer development between patients with and without COVID-19 across different genders and age groups (18–40 years, 41–60 years, and over 60 years). We also assessed the risk among patients who received various antiviral therapies, as well as those who developed hyperthyroidism or hypothyroidism following COVID-19. Additional comparisons were made between COVID-19 patients aged 60 years and older and those aged 18 to 60 years, between those aged 40 years and older and those aged 18 to 40 years; between those who received antiviral therapy and those who did not, and between individuals with post-infectious thyroid dysfunction (hyperthyroidism or hypothyroidism) and those without any thyroid function alterations following infection. Finally, we compared COVID-19 patients who developed thyroid dysfunction with patients who had thyroid dysfunction in the absence of COVID-19. Detailed query criteria are provided in Supplementary Files S2 and S3.

### 2.3. Statistical Analysis

We performed 1:1 propensity score matching based on age at index, gender and overweight/obesity status, with the non-COVID cohort serving as the control group. In subsequent analyses, we additionally included personal history of irradiation and comorbidities (hypertension, ischemic heart disease, and diabetes mellitus) as matching covariates. Propensity score matching is an integrated function within the TriNetX platform. We selected the covariates to be included in the matching process, and the system conducted propensity score matching to compare outcomes between the cohorts.

The outcomes were compared using built-in functions within the TriNetX platform. We calculated the hazard ratio (HR) for thyroid cancer development in both groups. The proportional hazards assumption was tested using the generalized Schoenfeld approach, available in TriNetX. Survival probabilities were estimated using the Kaplan–Meier method. Statistical significance was defined as a *p*-value < 0.05, and a 95% confidence interval (CI) was used as additional evidence of statistical significance.

## 3. Results

**Figure 2:**
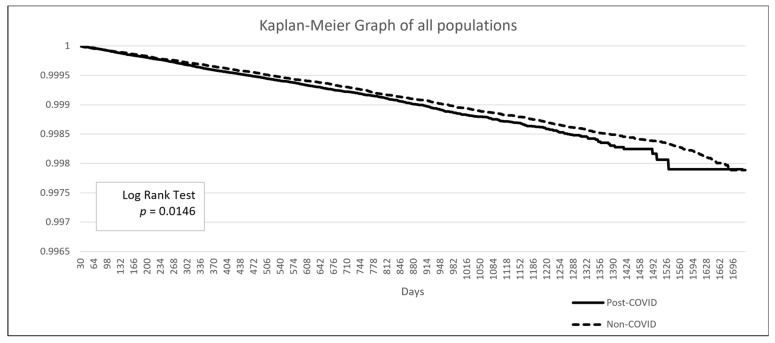
Kaplan–Meier survival curve comparing the risk of thyroid cancer between the post-COVID and non-COVID groups.

The baseline demographic data is presented in Table 3. The post-COVID group included 2,176,997 patients, while the non-COVID group comprised 28,535,672 patients. Due to the limitations of the TriNetX built-in function, propensity score matching in such large populations can only be performed on age, genders, and overweight/obesity status. After matching, the mean age was 48.8 years, and 57.9% of the population was female. A higher proportion of patients in the post-COVID group had comorbidities such as hypertensive diseases, ischemic heart disease, or diabetes mellitus. Additionally, a greater proportion of patients in the post-COVID group had a personal history of irradiation, although this represented less than 1% of the population (0.9% vs. 0.5%). The Kaplan–Meier analysis revealed a significant difference in the incidence of thyroid cancer following a COVID-19 diagnosis (HR: 1.104, 95% CI: 1.02–1.20, *p* = 0.0146) (Figure 2).

**Figure 3:**
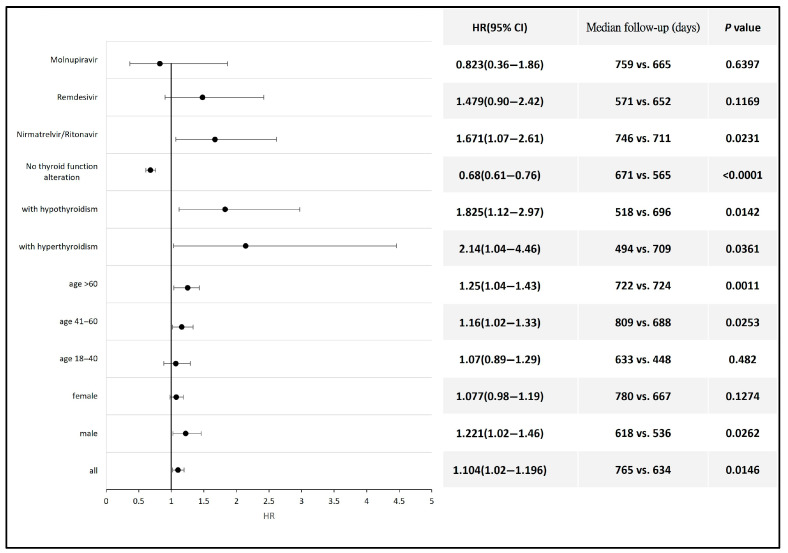
Forest plot showing the risk of thyroid cancer across different subgroups of COVID-19 patients compared to non-COVID-19 individuals or to those of the corresponding gender or age group.

The subgroup analysis revealed a significantly higher risk of thyroid cancer development following COVID-19 diagnosis in patients aged 60 years and older (HR 1.25, 95% CI: 1.04–1.43) and those aged between 41 and 60 years (HR 1.16, 95% CI: 1.02–1.33) compared with the non-COVID population within the same age range. Male patients also exhibited a higher risk of thyroid cancer after COVID-19 (HR 1.22, 95% CI 1.02–1.46). Additionally, the risk of thyroid cancer was also elevated in patients who developed hyperthyroidism (HR 2.14, 95% CI: 1.04–4.46) or hypothyroidism (HR 1.83, 95% CI: 1.12–2.97) compared with the non-COVID population. Antiviral therapy with Nirmatrelvir/Ritonavir was also associated with an increased risk of thyroid cancer (HR 1.67, 95% CI 1.07–2.61) compared with the non-COVID population. Antiviral agents such as Remdesivir and Molnupiravir had no significant impact on the incidence of thyroid cancer (Figure 3). Detailed data and a Kaplan–Meier graph are provided in Supplementary File S4.

**Figure 4:**
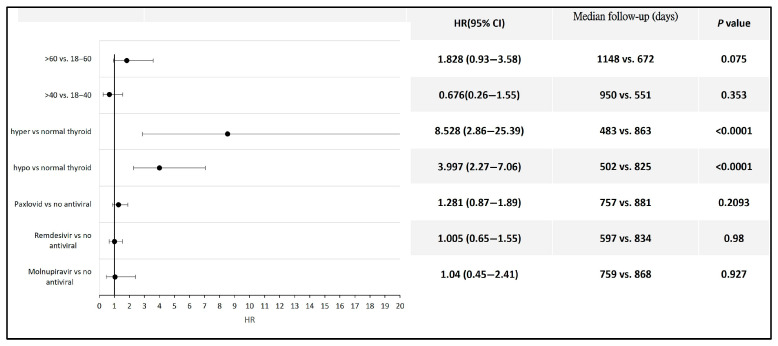
Forest plot illustrating thyroid cancer risk across different subpopulations following COVID-19.

Among the patients with COVID-19, those who developed hyperthyroidism or hypothyroidism had a significantly higher risk of developing thyroid cancer compared to those without thyroid function alterations following infection (HR 8.53, 95% CI: 2.86–25.39 for hyperthyroidism; HR 3.99, 95% CI: 2.27–7.06 for hypothyroidism). No statistically significant difference in thyroid cancer risk was observed between COVID-19 patients aged 60 years and older and those aged 18 to 60 years, between those aged 40 years and older and those aged 18 to 40 years, or between patients who received antiviral therapy and those who did not (Figure 4). Detailed data and Kaplan–Meier curves are provided in Supplementary File S5.

**Figure 5:**
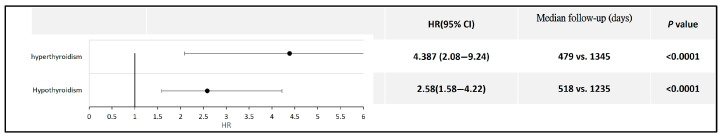
Forest plot comparing thyroid cancer risk in patients with thyroid dysfunction, with and without a history of COVID-19.

Furthermore, for patients with hyperthyroidism or hypothyroidism, those who had prior COVID-19 exhibited a relatively higher risk of developing thyroid cancer than those without a history of COVID-19 (HR 4.387, 95% CI: 2.08–9.24 for hyperthyroidism; HR 2.58, 95% CI: 1.58–4.22 for hypothyroidism) (Figure 5). Detailed data and Kaplan–Meier curves are provided in Supplementary File S6.

## 4. Discussion

This retrospective study using the TriNetX database demonstrated a direct association between COVID-19 and the overall development of thyroid cancer. Subgroup analysis revealed an increased risk of thyroid cancer in patients who developed hyperthyroidism or hypothyroidism following COVID-19, as well as in male patients. Individuals aged 60 years and older or those aged between 41 and 60 years, as well as those who received Nirmatrelvir/Ritonavir therapy, also exhibited a higher risk. However, among COVID-19 patients, only those who developed hyperthyroidism or hypothyroidism—compared with those without thyroid dysfunction—showed statistically significant results. Neither age (over 60 vs. 18–60 years or over 41 vs. 18–40 years) nor antiviral therapy (Nirmatrelvir/Ritonavir, Remdesivir, or Molnupiravir vs. no antiviral therapy) had a significant impact on thyroid cancer development.

There is limited evidence regarding the impact of COVID-19 on thyroid cancer development [29]. Xu et al. applied the Mendelian randomization (MR) method to analyze genome-wide association study data on COVID-19 susceptibility and severity in the European population. Their study suggested that hospitalization due to COVID-19 is associated with an increased risk of thyroid cancer development. However, MR results derived from genetic data did not show a significant association between COVID-19 susceptibility (risk of infection) or severity and thyroid cancer [30]. Hassan et al. conducted a retrospective study at an endocrine surgery center in the United Arab Emirates and found not only an increase in thyroid cancer cases undergoing surgery, but also more aggressive pathological features in the post-pandemic era (between January 2021 and December 2022) [31]. Our results demonstrated a direct association between COVID-19 diagnosis and the development of thyroid cancer. However, due to the limitations of the TriNetX database, we were unable to analyze the association between COVID-19 hospitalization and thyroid cancer development.

Given the indolent nature of thyroid cancer, the increased number of diagnoses observed after COVID-19 infection may partially reflect a diagnostic shift rather than a true rise in incidence. Several studies have reported delays in thyroid cancer screening and diagnosis during the pandemic, likely due to reduced healthcare access and concerns about aerosol-generating procedures such as fine-needle aspiration [32,33]. Bell et al. conducted a longitudinal analysis showing a decline in thyroid cancer diagnoses between March 2020 and December 2021, followed by a rebound exceeding pre-pandemic levels [34]. The authors attributed this to delayed diagnoses during the pandemic. Similarly, Virnceanu et al. noted not only a post-pandemic rise in incidence but also an increase in the presentation of giant thyroid cancers [35]. While such delays likely occurred equally across both COVID-19 and non-COVID-19 populations, this would only affect the results if patients with preexisting thyroid cancer had increased susceptibility to COVID-19. To address this issue, we note that to date, there is no evidence indicating increased susceptibility to COVID-19 among patients with thyroid cancer [29]. For example, a single-center study by Prete et al. reported no elevated prevalence of COVID-19 in patients with untreated thyroid cancer compared to the general population [36]. Thus, the likelihood of pre-existing, undiagnosed thyroid cancer should be similar between COVID-19 and non-COVID-19 groups.

Hyperthyroidism was previously thought to be associated with a decreased risk of thyroid cancer [37]. However, a systematic review and meta-analysis published in 2020 found that both hyperthyroidism and hypothyroidism are associated with an increased risk of thyroid cancer [38]. The potential for diagnostic bias may exist in those with pre-existing thyroid dysfunction, particularly due to increased medical attention and surveillance, such as thyroid ultrasonography. In our study, among COVID-19 patients, those who developed hyperthyroidism or hypothyroidism had a higher risk of thyroid cancer compared to those without thyroid dysfunction. To determine whether thyroid dysfunction or COVID-19 was primarily responsible for this increased risk, we specifically compared patients with thyroid dysfunction who had a prior COVID-19 infection to those with thyroid dysfunction who did not. Despite similar underlying thyroid conditions and likely comparable exposure to thyroid imaging, we observed a higher incidence of thyroid cancer in the post-COVID group. These findings suggest that the observed difference may not be solely attributed to increased ultrasound utilization associated with thyroid dysfunction. Instead, both thyroid dysfunction and COVID-19 itself may independently or synergistically contribute to an increased risk of thyroid cancer.

COVID-19 patients, especially those who were hospitalized, may have undergone more medical examinations, such as chest computed tomography scans to assess the severity of infection. These imaging studies could incidentally detect thyroid nodules, potentially leading to a higher likelihood of subsequent cancer diagnosis. This may partly explain our findings.

Many antiviral agents used to treat COVID-19 are known to reduce disease severity and shorten the duration of recovery [39,40,41]. Remdesivir, Nirmatrelvir/Ritonavir, and Molnupiravir have been shown to be effective in treating COVID-19 by inhibiting SARS-CoV-2 replication or viral RNA synthesis [42,43,44,45]. Additionally, Remdesivir has been reported to increase the ratio of T helper 2- to T helper 1-associated cytokines [39], while Nirmatrelvir/Ritonavir has been shown to reduce pro-inflammatory cytokine levels in COVID-19 patients [41,46]. In our study, compared to the non-COVID population, COVID-19 patients who received Nirmatrelvir/Ritonavir therapy exhibited an increased risk of thyroid cancer. However, when compared with COVID-19 patients who did not receive antiviral therapy, there was no significant difference in thyroid cancer risk after treatment with either antiviral agent. Therefore, none of these three antiviral agents appeared to reduce the incidence of thyroid cancer in our study.

Both mRNA and adenovirus-vectored COVID-19 vaccines have been reported to cause thyroiditis [47]. Thyroiditis can lead to alterations in thyroid function, potentially introducing bias into our analysis. Therefore, individuals who received any form of COVID-19 vaccination were excluded from this study.

Our study has several limitations. As noted by Ludwig et al. [48], studies utilizing electronic health records are subject to inherent biases. Retrospective cohort studies based on electronic data may suffer from incomplete access to raw clinical details, including hospitalization status, COVID-19 severity, patients’ vital signs, treatment regimens, thyroid cancer histological subtypes, and other important variables. Furthermore, due to limitations of the TriNetX platform, we were unable to perform full propensity score matching beyond age, gender, and obesity, which may affect the robustness of our comparisons. Differences in follow-up duration across subgroups may also lead to either an overestimation or an underestimation of thyroid cancer incidence. Additional sources of bias may arise from miscoding, inaccurate coding, or missing data related to comorbidities, socioeconomic status, gender and lifestyle factors, and family history of thyroid cancer. Finally, the observed increase in thyroid cancer incidence in certain subgroups may reflect delayed diagnoses or underdiagnosis during the pre-pandemic period, rather than a true increase in risk. The relatively small sample size, multiple comparisons, and absence of multivariable analysis in the subgroup analyses may also introduce bias.

## 5. Conclusions

This study identified an association between COVID-19 and the subsequent development of thyroid cancer across a large, diverse population. Subgroup analyses further revealed a significantly increased risk in male patients and in those who developed hyperthyroidism or hypothyroidism following COVID-19, suggesting that male sex and post-infectious thyroid dysfunction may amplify susceptibility to thyroid oncogenesis in the context of SARS-CoV-2 infection. However, limitations such as the retrospective design, incomplete clinical information, and constraints in propensity score matching warrant cautious interpretation of the findings. Prospective studies are needed to further clarify the relationship between COVID-19 and thyroid cancer.

## Figures and Tables

**Table 1:** Detailed criteria used to define a positive SARS-CoV-2 PCR test.

Code	Name
**UMLS:LNC:94500-6**	SARS-CoV-2 (COVID-19) RNA [Presence] in respiratory specimen by NAA with probe detection (labResult: Positive)
**UMLS:LNC:94309-2**	SARS-CoV-2 (COVID-19) RNA [Presence] in specimen by NAA with probe detection (labResult: Positive)
**UMLS:LNC:94565-9**	SARS-CoV-2 (COVID-19) RNA [Presence] in nasopharynx by NAA with non-probe detection (labResult: Positive)
**UMLS:LNC:94759-8**	SARS-CoV-2 (COVID-19) RNA [Presence] in nasopharynx by NAA with probe detection (labResult: Positive)
**UMLS:LNC:95608-6**	SARS-CoV-2 (COVID-19) RNA [Presence] in respiratory specimen by NAA with non-probe detection (labResult: Positive)
**UMLS:LNC:94845-5**	SARS-CoV-2 (COVID-19) RNA [Presence] in saliva (oral fluid) by NAA with probe detection (labResult: Positive)
**UMLS:LNC:95406-5**	SARS-CoV-2 (COVID-19) RNA [Presence] in nose by NAA with probe detection (labResult: Positive)

**Table 2:** Detailed criteria for defining SARS-CoV-2 vaccination status.

Code	Name
**NLM:CVX:208**	COVID-19, mRNA, LNP-S, PF, 30 mcg/0.3 mL dose
**NLM:CVX:207**	COVID-19, mRNA, LNP-S, PF, 100 mcg/0.5 mL dose or 50 mcg/0.25 mL dose
**NLM:CVX:212**	COVID-19 vaccine, vector-nr, rS-Ad26, PF, 0.5 mL
**NLM:RXNORM:OMOP5042939**	COVID-19 vaccine
**NLM:CVX:300**	COVID-19, mRNA, LNP-S, bivalent, PF, 30 mcg/0.3 mL dose
**NLM:CVX:217**	COVID-19, mRNA, LNP-S, PF, 30 mcg/0.3 mL dose, tris-sucrose
**NLM:CVX:229**	COVID-19, mRNA, LNP-S, bivalent, PF, 50 mcg/0.5 mL or 25 mcg/0.25 mL dose
**NLM:CVX:218**	COVID-19, mRNA, LNP-S, PF, 10 mcg/0.2 mL dose, tris-sucrose
**NLM:CVX:520**	COVID-19 mRNA, bivalent, original/Omicron BA.1, non-US vaccine product, Pfizer-BioNTech
**NLM:CVX:519**	COVID-19 mRNA, bivalent, original/Omicron BA.1, non-US vaccine (Spikevax Bivalent), Moderna
**NLM:CVX:301**	COVID-19, mRNA, LNP-S, bivalent, PF, 10 mcg/0.2 mL dose
**NLM:CVX:219**	COVID-19, mRNA, LNP-S, PF, 3 mcg/0.2 mL dose, tris-sucrose
**NLM:CVX:228**	COVID-19, mRNA, LNP-S, PF, pediatric 25 mcg/0.25 mL dose
**NLM:CVX:230**	COVID-19, mRNA, LNP-S, bivalent booster, PF, 10 mcg/0.2 mL
**NLM:CVX:221**	COVID-19, mRNA, LNP-S, PF, 50 mcg/0.5 mL dose
**NLM:CVX:210**	COVID-19 vaccine, vector-nr, rS-ChAdOx1, PF, 0.5 mL
**NLM:CVX:302**	COVID-19, mRNA, LNP-S, bivalent, PF, 3 mcg/0.2 mL dose
**NLM:CVX:511**	COVID-19 IV non-US vaccine (CoronaVac, Sinovac)
**NLM:RXNORM:2468231**	SARS-CoV-2 (COVID-19) vaccine, mRNA spike protein
**UMLS:CPT:91300**	Severe acute respiratory syndrome coronavirus 2 (SARS-CoV-2) (coronavirus disease [COVID-19]) vaccine, mRNA-LNP, spike protein, preservative-free, 30 mcg/0.3 mL dosage, diluent reconstituted, for intramuscular use
**UMLS:CPT:0001A**	Immunization administration by intramuscular injection of severe acute respiratory syndrome coronavirus 2 (SARS-CoV-2) (coronavirus disease [COVID-19]) vaccine, mRNA-LNP, spike protein, preservative-free, 30 mcg/0.3 mL dosage, diluent-reconstituted; first dose
**UMLS:CPT:0002A**	Immunization administration by intramuscular injection of severe acute respiratory syndrome coronavirus 2 (SARS-CoV-2) (coronavirus disease [COVID-19]) vaccine, mRNA-LNP, spike protein, preservative-free, 30 mcg/0.3 mL dosage, diluent-reconstituted; second dose
**UMLS:CPT:91301**	Severe acute respiratory syndrome coronavirus 2 (SARS-CoV-2) (coronavirus disease [COVID-19]) vaccine, mRNA-LNP, spike protein, preservative free, 100 mcg/0.5 mL dosage, for intramuscular use
**UMLS:CPT:0011A**	Immunization administration by intramuscular injection of severe acute respiratory syndrome coronavirus 2 (SARS-CoV-2) (coronavirus disease [COVID-19]) vaccine, mRNA-LNP, spike protein, preservative-free, 100 mcg/0.5 mL dosage; first dose
**UMLS:CPT:0012A**	Immunization administration by intramuscular injection of severe acute respiratory syndrome coronavirus 2 (SARS-CoV-2) (coronavirus disease [COVID-19]) vaccine, mRNA-LNP, spike protein, preservative-free, 100 mcg/0.5 mL dosage; second dose
**UMLS:SNOMED:840534001**	Administration of SARS-CoV-2 antigen vaccine
**NLM:CVX:213**	SARS-CoV-2 (COVID-19) vaccine
**UMLS:CPT:1036660**	Immunization administration by intramuscular injection of severe acute respiratory syndrome coronavirus 2 (SARS-CoV-2) (coronavirus disease [COVID-19]) vaccine, mRNA-LNP, spike protein, preservative-free, 30 mcg/0.3 mL dosage, diluent reconstituted
**UMLS:CPT:1036663**	Immunization administration by intramuscular injection of severe acute respiratory syndrome coronavirus 2 (SARS-CoV-2) (coronavirus disease [COVID-19]) vaccine, mRNA-LNP, spike protein, preservative-free, 100 mcg/0.5 mL dosage
**UMLS:CPT:0124A**	Immunization administration by intramuscular injection of severe acute respiratory syndrome coronavirus 2 (SARS-CoV-2) (coronavirus disease [COVID-19]) vaccine, mRNA-LNP, bivalent spike protein, preservative free, 30 mcg/0.3 mL dosage, tris-sucrose formulation; booster dose
**UMLS:CPT:0004A**	Immunization administration by intramuscular injection of severe acute respiratory syndrome coronavirus 2 (SARS-CoV-2) (coronavirus disease [COVID-19]) vaccine, mRNA-LNP, spike protein, preservative-free, 30 mcg/0.3 mL dosage, diluent-reconstituted; booster dose
**UMLS:CPT:0003A**	Immunization administration by intramuscular injection of severe acute respiratory syndrome coronavirus 2 (SARS-CoV-2) (coronavirus disease [COVID-19]) vaccine, mRNA-LNP, spike protein, preservative-free, 30 mcg/0.3 mL dosage, diluent-reconstituted; third dose
**UMLS:CPT:1037166**	Immunization administration by intramuscular injection of severe acute respiratory syndrome coronavirus 2 (SARS-CoV-2) (coronavirus disease [COVID-19]) vaccine, mRNA-LNP, spike protein, preservative-free, 30 mcg/0.3 mL dosage, tris-sucrose formulation
**UMLS:CPT:0054A**	Immunization administration by intramuscular injection of severe acute respiratory syndrome coronavirus 2 (SARS-CoV-2) (coronavirus disease [COVID-19]) vaccine, mRNA-LNP, spike protein, preservative-free, 30 mcg/0.3 mL dosage, tris-sucrose formulation; booster dose
**UMLS:CPT:0064A**	Immunization administration by intramuscular injection of severe acute respiratory syndrome coronavirus 2 (SARS-CoV-2) (coronavirus disease [COVID-19]) vaccine, mRNA-LNP, spike protein, preservative free, 50 mcg/0.25 mL dosage; booster dose
**UMLS:CPT:90480**	Immunization administration by intramuscular injection of severe acute respiratory syndrome coronavirus 2 (SARS-CoV-2) (coronavirus disease [COVID-19]) vaccine; single dose
**UMLS:CPT:1037171**	Immunization administration by intramuscular injection of severe acute respiratory syndrome coronavirus 2 (SARS-CoV-2) (coronavirus disease [COVID-19]) vaccine, mRNA-LNP, spike protein, preservative-free, 10 mcg/0.2 mL dosage, diluent reconstituted, tris-sucrose formulation
**UMLS:CPT:0071A**	Immunization administration by intramuscular injection of severe acute respiratory syndrome coronavirus 2 (SARS-CoV-2) (coronavirus disease [COVID-19]) vaccine, mRNA-LNP, spike protein, preservative-free, 10 mcg/0.2 mL dosage, diluent reconstituted, tris-sucrose formulation; first dose
**UMLS:CPT:0072A**	Immunization administration by intramuscular injection of severe acute respiratory syndrome coronavirus 2 (SARS-CoV-2) (coronavirus disease [COVID-19]) vaccine, mRNA-LNP, spike protein, preservative-free, 10 mcg/0.2 mL dosage, diluent reconstituted, tris-sucrose formulation; second dose
**NLM:RXNORM:2610319**	SARS-CoV-2 (COVID-19) vaccine, mRNA-BNT162b2 0.05 MG/ML/SARS-CoV-2 (COVID-19) vaccine, mRNA-BNT162b2 OMICRON (BA.4/BA.5) 0.05 MG/ML injectable suspension
**UMLS:CPT:91313**	Severe acute respiratory syndrome coronavirus 2 (SARS-CoV-2) (coronavirus disease [COVID-19]) vaccine, mRNA-LNP, spike protein, bivalent, preservative-free, 50 mcg/0.5 mL dosage, for intramuscular use
**UMLS:CPT:0134A**	Immunization administration by intramuscular injection of severe acute respiratory syndrome coronavirus 2 (SARS-CoV-2) (coronavirus disease [COVID-19]) vaccine, mRNA-LNP, spike protein, bivalent, preservative-free, 50 mcg/0.5 mL dosage; booster dose
**UMLS:CPT:1037175**	Immunization administration by intramuscular injection of severe acute respiratory syndrome coronavirus 2 (SARS-CoV-2) (coronavirus disease [COVID-19]) vaccine, DNA, spike protein, adenovirus type 26 (Ad26) vector, preservative-free, 5 × 1010 viral particles/0.5 mL dosage
**NLM:RXNORM:2610347**	0.3 ML SARS-CoV-2 (COVID-19) vaccine, mRNA-BNT162b2 0.05 MG/ML/SARS-CoV-2 (COVID-19) vaccine, mRNA-BNT162b2 OMICRON (BA.4/BA.5) 1 MG/ML injection
**UMLS:CPT:1037228**	Immunization administration by intramuscular injection of severe acute respiratory syndrome coronavirus 2 (SARS-CoV-2) (coronavirus disease [COVID-19]) vaccine, mRNA-LNP, spike protein, preservative-free, 3 mcg/0.2 mL dosage, diluent-reconstituted, tris-sucrose formulation
**UMLS:CPT:0013A**	Immunization administration by intramuscular injection of severe acute respiratory syndrome coronavirus 2 (SARS-CoV-2) (coronavirus disease [COVID-19]) vaccine, mRNA-LNP, spike protein, preservative-free, 100 mcg/0.5 mL dosage; third dose
**UMLS:CPT:0081A**	Immunization administration by intramuscular injection of severe acute respiratory syndrome coronavirus 2 (SARS-CoV-2) (coronavirus disease [COVID-19]) vaccine, mRNA-LNP, spike protein, preservative-free, 3 mcg/0.2 mL dosage, diluent-reconstituted, tris-sucrose formulation; first dose
**UMLS:CPT:0082A**	Immunization administration by intramuscular injection of severe acute respiratory syndrome coronavirus 2 (SARS-CoV-2) (coronavirus disease [COVID-19]) vaccine, mRNA-LNP, spike protein, preservative-free, 3 mcg/0.2 mL dosage, diluent-reconstituted, tris-sucrose formulation; second dose
**NLM:RXNORM:2610328**	SARS-CoV-2 (COVID-19) vaccine, mRNA-1273 0.05 MG/ML/SARS-CoV-2 (COVID-19) vaccine, mRNA-1273 OMICRON (BA.4/BA.5) 0.05 MG/ML injectable suspension
**UMLS:CPT:0154A**	Immunization administration by intramuscular injection of severe acute respiratory syndrome coronavirus 2 (SARS-CoV-2) (coronavirus disease [COVID-19]) vaccine, mRNA-LNP, bivalent spike protein, preservative free, 10 mcg/0.2 mL dosage, diluent reconstituted, tris-sucrose formulation; booster dose
**UMLS:CPT:0053A**	Immunization administration by intramuscular injection of severe acute respiratory syndrome coronavirus 2 (SARS-CoV-2) (coronavirus disease [COVID-19]) vaccine, mRNA-LNP, spike protein, preservative free, 30 mcg/0.3 mL dosage, tris-sucrose formulation; third dose
**UMLS:CPT:1037332**	Immunization administration by intramuscular injection of severe acute respiratory syndrome coronavirus 2 (SARS-CoV-2) (coronavirus disease [COVID-19]) vaccine, mRNA-LNP, spike protein, preservative free, 25 mcg/0.25 mL dosage
**UMLS:CPT:0052A**	Immunization administration by intramuscular injection of severe acute respiratory syndrome coronavirus 2 (SARS-CoV-2) (coronavirus disease [COVID-19]) vaccine, mRNA-LNP, spike protein, preservative free, 30 mcg/0.3 mL dosage, tris-sucrose formulation; second dose
**UMLS:CPT:0111A**	Immunization administration by intramuscular injection of severe acute respiratory syndrome coronavirus 2 (SARS-CoV-2) (coronavirus disease [COVID-19]) vaccine, mRNA-LNP, spike protein, preservative free, 25 mcg/0.25 mL dosage; first dose
**UMLS:CPT:0051A**	Immunization administration by intramuscular injection of severe acute respiratory syndrome coronavirus 2 (SARS-CoV-2) (coronavirus disease [COVID-19]) vaccine, mRNA-LNP, spike protein, preservative free, 30 mcg/0.3 mL dosage, tris-sucrose formulation; first dose
**UMLS:CPT:91311**	Severe acute respiratory syndrome coronavirus 2 (SARS-CoV-2) (coronavirus disease [COVID-19]) vaccine, mRNA-LNP, spike protein, preservative free, 25 mcg/0.25 mL dosage, for intramuscular use
**UMLS:CPT:0074A**	Immunization administration by intramuscular injection of severe acute respiratory syndrome coronavirus 2 (SARS-CoV-2) (coronavirus disease [COVID-19]) vaccine, mRNA-LNP, spike protein, preservative free, 10 mcg/0.2 mL dosage, diluent reconstituted, tris-sucrose formulation; booster dose
**UMLS:CPT:0112A**	Immunization administration by intramuscular injection of severe acute respiratory syndrome coronavirus 2 (SARS-CoV-2) (coronavirus disease [COVID-19]) vaccine, mRNA-LNP, spike protein, preservative free, 25 mcg/0.25 mL dosage; second dose
**UMLS:CPT:0083A**	Immunization administration by intramuscular injection of severe acute respiratory syndrome coronavirus 2 (SARS-CoV-2) (coronavirus disease [COVID-19]) vaccine, mRNA-LNP, spike protein, preservative free, 3 mcg/0.2 mL dosage, diluent reconstituted, tris-sucrose formulation; third dose
**UMLS:CPT:0073A**	Immunization administration by intramuscular injection of severe acute respiratory syndrome coronavirus 2 (SARS-CoV-2) (coronavirus disease [COVID-19]) vaccine, mRNA-LNP, spike protein, preservative free, 10 mcg/0.2 mL dosage, diluent reconstituted, tris-sucrose formulation; third dose
**UMLS:CPT:0173A**	Immunization administration by intramuscular injection of severe acute respiratory syndrome coronavirus 2 (SARS-CoV-2) (coronavirus disease [COVID-19]) vaccine, mRNA-LNP, bivalent spike protein, preservative free, 3 mcg/0.2 mL dosage, diluent reconstituted, tris-sucrose formulation; third dose
**UMLS:CPT:0164A**	Immunization administration by intramuscular injection of severe acute respiratory syndrome coronavirus 2 (SARS-CoV-2) (coronavirus disease [COVID-19]) vaccine, mRNA-LNP, spike protein, bivalent, preservative free, 10 mcg/0.2 mL dosage; booster dose
**UMLS:CPT:1037838**	Immunization administration by intramuscular injection of severe acute respiratory syndrome coronavirus 2 (SARS-CoV-2) (coronavirus disease [COVID-19]) vaccine, mRNA-LNP, spike protein, preservative free, 50 mcg/0.5 mL dosage
**UMLS:CPT:0094A**	Immunization administration by intramuscular injection of severe acute respiratory syndrome coronavirus 2 (SARS-CoV-2) (coronavirus disease [COVID-19]) vaccine, mRNA-LNP, spike protein, preservative free, 50 mcg/0.5 mL dosage; booster dose, when administered to individuals aged 18 years and over
**UMLS:CPT:0034A**	Immunization administration by intramuscular injection of severe acute respiratory syndrome coronavirus 2 (SARS-CoV-2) (coronavirus disease [COVID-19]) vaccine, DNA, spike protein, adenovirus type 26 (Ad26) vector, preservative free, 5 × 1010 viral particles/0.5 mL dosage; booster dose
**UMLS:CPT:0144A**	Immunization administration by intramuscular injection of severe acute respiratory syndrome coronavirus 2 (SARS-CoV-2) (coronavirus disease [COVID-19]) vaccine, mRNA-LNP, spike protein, bivalent, preservative free, 25 mcg/0.25 mL dosage; booster dose
**UMLS:CPT:0091A**	Immunization administration by intramuscular injection of severe acute respiratory syndrome coronavirus 2 (SARS-CoV-2) (coronavirus disease [COVID-19]) vaccine, mRNA-LNP, spike protein, preservative free, 50 mcg/0.5 mL dosage; first dose, when administered to individuals aged 6 through 11 years
**UMLS:CPT:0174A**	Immunization administration by intramuscular injection of severe acute respiratory syndrome coronavirus 2 (SARS-CoV-2) (coronavirus disease [COVID-19]) vaccine, mRNA-LNP, bivalent spike protein, preservative free, 3 mcg/0.2 mL dosage, diluent reconstituted, tris-sucrose formulation; booster
**UMLS:CPT:0092A**	Immunization administration by intramuscular injection of severe acute respiratory syndrome coronavirus 2 (SARS-CoV-2) (coronavirus disease [COVID-19]) vaccine, mRNA-LNP, spike protein, preservative free, 50 mcg/0.5 mL dosage; second dose, when administered to individuals aged 6 through 11 years
**UMLS:CPT:1036682**	Immunization administration by intramuscular injection of severe acute respiratory syndrome coronavirus 2 (SARS-CoV-2) (coronavirus disease [COVID-19]) vaccine, recombinant spike protein nanoparticle, saponin-based adjuvant, preservative free, 5 mcg/0.5 mL dosage
**UMLS:CPT:0041A**	Immunization administration by intramuscular injection of severe acute respiratory syndrome coronavirus 2 (SARS-CoV-2) (coronavirus disease [COVID-19]) vaccine, recombinant spike protein nanoparticle, saponin-based adjuvant, preservative free, 5 mcg/0.5 mL dosage; first dose
**UMLS:CPT:0113A**	Immunization administration by intramuscular injection of severe acute respiratory syndrome coronavirus 2 (SARS-CoV-2) (coronavirus disease [COVID-19]) vaccine, mRNA-LNP, spike protein, preservative free, 25 mcg/0.25 mL dosage; third dose
**UMLS:CPT:0042A**	Immunization administration by intramuscular injection of severe acute respiratory syndrome coronavirus 2 (SARS-CoV-2) (coronavirus disease [COVID-19]) vaccine, recombinant spike protein nanoparticle, saponin-based adjuvant, preservative free, 5 mcg/0.5 mL dosage; second dose
**UMLS:CPT:0093A**	Immunization administration by intramuscular injection of severe acute respiratory syndrome coronavirus 2 (SARS-CoV-2) (coronavirus disease [COVID-19]) vaccine, mRNA-LNP, spike protein, preservative free, 50 mcg/0.5 mL dosage; third dose, when administered to individuals aged 6 through 11 years
**UMLS:CPT:1036666**	Immunization administration by intramuscular injection of severe acute respiratory syndrome coronavirus 2 (SARS-CoV-2) (coronavirus disease [COVID-19]) vaccine, DNA, spike protein, chimpanzee adenovirus Oxford 1 (ChAdOx1) vector, preservative free, 5 × 1010 viral particles/0.5 mL dosage
**UMLS:CPT:0044A**	Immunization administration by intramuscular injection of severe acute respiratory syndrome coronavirus 2 (SARS-CoV-2) (coronavirus disease [COVID-19]) vaccine, recombinant spike protein nanoparticle, saponin-based adjuvant, preservative free, 5 mcg/0.5 mL dosage; booster

**Table 3:** Characteristics of the post-COVID group (Cohort 1) and non-COVID group (Cohort 2) before and after propensity score matching.

Before Propensity Score Matching						After Propensity Score Matching			
Cohort		Patients	Mean ± SD	*p*-Value	Std Diff.	Patients	Mean ± SD	*p*-Value	Std Diff.
1 2	Age at Index	2,176,997 28,535,672	48.8 +/− 20.1 48.5 +/− 20.4	<0.001	0.014	2,176,997 2,176,997	48.8 +/− 20.1 48.8 +/− 20.1	1	<0.001
Cohort		Patients	% of Cohort	*p*-Value	Std diff.	Patients	% of Cohort	*p*-Value	Std diff.
1 2	Male	916,516 12,413,017	42.1% 43.5%	<0.001	0.028	916,516 916,516	42.1% 42.1%	1	<0.001
1 2	Female	1,260,481 16,221,655	57.9% 56.5%	<0.001	0.028	1,260,481 1,260,481	57.9% 57.9%	1	<0.001
1 2	White	1,254,735 13,920,716	54.5% 49.4%	<0.001	0.102	1,254,735 1,174,062	54.5% 51.0%	<0.001	0.070
1 2	Asian	54,299 972,965	2.4% 3.5%	<0.001	0.065	54,299 74,568	2.4% 3.2%	<0.001	0.053
1 2	Black or African American	345,156 3,320,705	15.0% 11.8%	<0.001	0.094	345,156 295,739	15.0% 12.8%	<0.001	0.062
1 2	Other Race	66,799 1,072,646	2.9% 3.8%	<0.001	0.050	66,799 82,598	2.9% 3.6%	<0.001	0.039
1 2	Unknown Race	564,364 8,707,668	24.5% 30.9%	<0.001	0.143	564,364 659,678	24.5% 28.7%	<0.001	0.094
1 2	Personal history of nicotine dependence	197,813 790,130	8.6% 2.8%	<0.001	0.252	197,813 96,657	8.6% 4.2%	<0.001	0.180
1 2	Personal history of irradiation	19,856 101,810	0.9% 0.4%	<0.001	0.064	19,856 11,444	0.9% 0.5%	<0.001	0.044
1 2	Overweight and obesity	391,198 1,656,174	17.0% 5.9%	<0.001	0.355	391,198 391,198	17.0% 17.0%	1	<0.001
1 2	Diabetes mellitus	293,835 1,534,074	12.8% 5.4%	<0.001	0.257	293,835 189,472	12.8% 8.2%	<0.001	0.148
1 2	Hypertensive diseases	635,880 3,663,669	27.6% 13.0%	<0.001	0.370	635,880 413,136	27.6% 17.9%	<0.001	0.232
1 2	Ischemic heart diseases	213,759 1,160,544	9.3% 4.1%	<0.001	0.208	213,759 127,732	9.3% 5.5%	<0.001	0.143

## Data Availability

All data generated or analyzed during this study are included in this published article and its Supplementary Files.

## Supplementary Materials

The following supporting information can be downloaded at https://www.mdpi.com/article/10.3390/biomedicines13081933/s1. The original contributions presented in this study are included in the article/supplementary material. Further inquiries can be directed to the corresponding authors. Supplementary File S1: Detailed query criteria of post-COVID and non-COVID group. Query Criteria for Cohort post-COVID; Query Criteria for Cohort non-COVID. Supplementary File S2: Detailed query criteria of subgroups of population in post-COVID and non-COVID groups. Query Criteria for Cohort post-COVID, male; Query Criteria for Cohort non-COVID, male; Query Criteria for Cohort post-COVID, female; Query Criteria for Cohort non-COVID, female; Query Criteria for Cohort post-COVID, >60; Query Criteria for Cohort non-COVID, >60; Query Criteria for Cohort post-COVID, 41–60; Query Criteria for Cohort non-COVID, 41–60; Query Criteria for Cohort post-COVID, 18–40; Query Criteria for Cohort non-COVID, 18–40; Query Criteria for Cohort post-COVID, with hyperthyroidism; Query Criteria for Cohort post-COVID, with hypothyroidism; Query Criteria for Cohort post-COVID, s/p Nirmatrelvir/Ritonavir; Query Criteria for Cohort post-COVID, s/p Molnupiravir; Query Criteria for Cohort post-COVID, s/p Remdesivir. Supplementary File S3. Detailed query criteria of subgroup of population. Query Criteria for Cohort post-COVID, >41; Query Criteria for Cohort post-COVID, 18–40; Query Criteria for Cohort post-COVID, >60; Query Criteria for Cohort post-COVID, 18-60; Query Criteria for Cohort post-COVID, normal thyroid function; Query Criteria for Cohort post-COVID, with hypothyroidism; Query Criteria for Cohort post-COVID, with hyperthyroidism; Query Criteria for Cohort non-COVID, with hypothyroidism; Query Criteria for Cohort non-COVID, with hyperthyroidism. Supplementary File S4. Kaplan-Meier graph of thyroid cancer risk in different subgroups of COVID-19 patients. Supplementary Figure S4.1. Kaplan—Meier survival curve of thyroid cancer risk in male populations. Supplementary Figure S4.2. Kaplan—Meier survival curve of thyroid cancer risk in female populations. Supplementary Figure S4.3. Kaplan—Meier survival curve of thyroid cancer risk in populations aged between 18 and 40 years. Supplementary Figure S4.4. Kaplan—Meier survival curve of thyroid cancer risk in populations aged between 41 and 60 years. Supplementary Figure S4.5. Kaplan—Meier survival curve of thyroid cancer risk in populations aged over 60 years. Supplementary Figure S4.6. Kaplan—Meier survival curve of thyroid cancer risk in post-COVID populations who developed hyperthyroidism vs non-COVID populations. Supplementary Figure S4.7. Kaplan—Meier survival curve of thyroid cancer risk in post-COVID populations who developed hypothyroidism vs non-COVID populations. Supplementary Figure S4.8. Kaplan—Meier survival curve of thyroid cancer risk in post-COVID populations who had no thyroid function alteration vs non-COVID populations. Supplementary Figure S4.9. Kaplan—Meier survival curve of thyroid cancer risk in post-COVID populations who received Nirmatrelvir/Ritonavir treatment vs non-COVID populations. Supplementary Figure S4.10. Kaplan—Meier survival curve of thyroid cancer risk in post-COVID populations who received Remdesivir treatment vs non-COVID populations. Supplementary Figure S4.11. Kaplan—Meier survival curve of thyroid cancer risk in post-COVID populations who received Molnupiravir treatment vs non-COVID populations. Supplementary File S5. Kaplan-Meier graph of thyroid cancer risk in different subpopulations following COVID-19. Supplementary Figure S5.1. Kaplan—Meier survival curve of thyroid cancer risk in different age interval of post-COVID populations. Supplementary Figure S5.2. Kaplan—Meier survival curve of thyroid cancer risk in different age interval of post-COVID populations. Supplementary Figure S5.3. Kaplan—Meier survival curve of thyroid cancer risk in post-COVID populations with hyperthyroidism vs no thyroid function alteration. Supplementary Figure S5.4. Kaplan—Meier survival curve of thyroid cancer risk in post-COVID populations with hypothyroidism vs no thyroid function alteration. Supplementary Figure S5.5. Kaplan—Meier survival curve of thyroid cancer risk in post-COVID populations who received Nirmatrelvir/Ritonavir vs no antiviral treatment. Supplementary Figure S5.6. Kaplan—Meier survival curve of thyroid cancer risk in post-COVID populations who received Remdesivir vs no antiviral treatment. Supplementary Figure S5.7. Kaplan—Meier survival curve of thyroid cancer risk in post-COVID populations who received Molnupiravir vs no antiviral treatment. Supplementary File S6. Kaplan–Meier curve illustrating the risk of thyroid cancer in subpopulations with thyroid dysfunction, stratified by COVID-19 status. Supplementary Figure S6.1. Kaplan—Meier survival curve of thyroid cancer risk in hyperthyroidism populations with or without COVID-19. Supplementary Figure S6.2. Kaplan—Meier survival curve of thyroid cancer risk in hypothyroidism populations with or without COVID-19.

## Abbreviations

The following abbreviations are used in this manuscript:

ACE2	angiotensin-converting enzyme II
CI	confidence interval
HCOs	healthcare organizations
HR	hazard ratio
MR	Mendelian randomization
nsp	nonstructural protein

## References

[1] Lai C.C., Shih T.P., Ko W.C., Tang H.J., Hsueh P.R. (2020). Severe acute respiratory syndrome coronavirus 2 (SARS-CoV-2) and coronavirus disease-2019 (COVID-19): The epidemic and the challenges. Int. J. Antimicrob. Agents.

[2] WHO Coronavirus (COVID-19) Dashboard. https://covid19.who.int/.

[3] Huang C., Wang Y., Li X., Ren L., Zhao J., Hu Y., Zhang L., Fan G., Xu J., Gu X. (2020). Clinical features of patients infected with 2019 novel coronavirus in Wuhan, China. Lancet.

[4] Marazuela M., Giustina A., Puig-Domingo M. (2020). Endocrine and metabolic aspects of the COVID-19 pandemic. Rev. Endocr. Metab. Disord..

[5] Siegel R.L., Miller K.D., Fuchs H.E., Jemal A. (2021). Cancer Statistics, 2021. CA Cancer J. Clin..

[6] Kitahara C.M., Schneider A.B. (2022). Epidemiology of Thyroid Cancer. Cancer Epidemiol. Biomark. Prev..

[7] Wells S.A. (2016). Progress in Endocrine Neoplasia. Clin. Cancer Res..

[8] Almansoori A., Busch H., Bendardaf R., Hamoudi R. (2022). Thyroid cancer incidence in the United Arab Emirates: A retrospective study on association with age and gender. F1000Research.

[9] Howlader N., Noone A.M., Krapcho M., Miller D., Brest A., Yu M., Ruhl J., Tatalovich Z., Mariotto A., Lewis D.R. (2021). SEER Cancer Statistics Review, 1975–2018.

[10] Siegel R.L., Miller K.D., Fuchs H.E., Jemal A. (2022). Cancer statistics, 2022. CA Cancer J. Clin..

[11] Rossetti C.L., Cazarin J., Hecht F., Beltrao F.E.L., Ferreira A.C.F., Fortunato R.S., Ramos H.E., de Carvalho D.P. (2022). COVID-19 and thyroid function: What do we know so far?. Front. Endocrinol..

[12] Scappaticcio L., Pitoia F., Esposito K., Piccardo A., Trimboli P. (2021). Impact of COVID-19 on the thyroid gland: An update. Rev. Endocr. Metab. Disord..

[13] Li M.Y., Li L., Zhang Y., Wang X.S. (2020). Expression of the SARS-CoV-2 cell receptor gene ACE2 in a wide variety of human tissues. Infect. Dis. Poverty.

[14] Hoffmann M., Kleine-Weber H., Schroeder S., Kruger N., Herrler T., Erichsen S., Schiergens T.S., Herrler G., Wu N.H., Nitsche A. (2020). SARS-CoV-2 Cell Entry Depends on ACE2 and TMPRSS2 and Is Blocked by a Clinically Proven Protease Inhibitor. Cell.

[15] Han T., Kang J., Li G., Ge J., Gu J. (2020). Analysis of 2019-nCoV receptor ACE2 expression in different tissues and its significance study. Ann. Transl. Med..

[16] Gressens S.B., Leftheriotis G., Dussaule J.C., Flamant M., Levy B.I., Vidal-Petiot E. (2021). Controversial Roles of the Renin Angiotensin System and Its Modulators During the COVID-19 Pandemic. Front. Physiol..

[17] Rodrigues-Ferreira S., Nahmias C. (2015). G-protein coupled receptors of the renin-angiotensin system: New targets against breast cancer?. Front. Pharmacol..

[18] Alipoor S.D., Mortaz E., Jamaati H., Tabarsi P., Bayram H., Varahram M., Adcock I.M. (2021). COVID-19: Molecular and Cellular Response. Front. Cell. Infect. Microbiol..

[19] Mortaz E., Tabarsi P., Varahram M., Folkerts G., Adcock I.M. (2020). The Immune Response and Immunopathology of COVID-19. Front. Immunol..

[20] Merad M., Martin J.C. (2020). Pathological inflammation in patients with COVID-19: A key role for monocytes and macrophages. Nat. Rev. Immunol..

[21] Lee A.J.X., Purshouse K. (2021). COVID-19 and cancer registries: Learning from the first peak of the SARS-CoV-2 pandemic. Br. J. Cancer.

[22] Coperchini F., Chiovato L., Croce L., Magri F., Rotondi M. (2020). The cytokine storm in COVID-19: An overview of the involvement of the chemokine/chemokine-receptor system. Cytokine Growth Factor Rev..

[23] Naguib R. (2022). Potential relationships between COVID-19 and the thyroid gland: An update. J. Int. Med. Res..

[24] Bhardwaj K., Liu P., Leibowitz J.L., Kao C.C. (2012). The coronavirus endoribonuclease Nsp15 interacts with retinoblastoma tumor suppressor protein. J. Virol..

[25] Stingi A., Cirillo L. (2021). SARS-CoV-2 infection and cancer: Evidence for and against a role of SARS-CoV-2 in cancer onset. Bioessays.

[26] Ma-Lauer Y., Carbajo-Lozoya J., Hein M.Y., Muller M.A., Deng W., Lei J., Meyer B., Kusov Y., von Brunn B., Bairad D.R. (2016). p53 down-regulates SARS coronavirus replication and is targeted by the SARS-unique domain and PLpro via E3 ubiquitin ligase RCHY1. Proc. Natl. Acad. Sci. USA.

[27] Leng R.P., Lin Y., Ma W., Wu H., Lemmers B., Chung S., Parant J.M., Lozano G., Hakem R., Benchimol S. (2003). Pirh2, a p53-induced ubiquitin-protein ligase, promotes p53 degradation. Cell.

[28] Mesri E.A., Feitelson M.A., Munger K. (2014). Human viral oncogenesis: A cancer hallmarks analysis. Cell Host Microbe.

[29] Duntas L.H., Jonklaas J. (2021). COVID-19 and Thyroid Diseases: A Bidirectional Impact. J. Endocr. Soc..

[30] Xu D., Wang X., Zhou D. (2023). Exploring the Potential Association between COVID-19 and Thyroid Cancer: A Mendelian Randomization Study. ACS Omega.

[31] Hassan I., Hassan L., Bacha F., Al Salameh M., Gatee O., Hassan W. (2024). Papillary Thyroid Cancer Trends in the Wake of the COVID-19 Pandemic: Is There a Shift toward a More Aggressive Entity?. Diseases.

[32] Qu N., Hui Z., Shen Z., Kan C., Hou N., Sun X., Han F. (2022). Thyroid Cancer and COVID-19: Prospects for Therapeutic Approaches and Drug Development. Front. Endocrinol..

[33] Spartalis E., Plakopitis N., Theodori M.A., Karagiannis S.P., Athanasiadis D.I., Spartalis M., Boutzios G., Paschou S.A., Nikiteas N., Troupis T. (2021). Thyroid cancer surgery during the coronavirus disease 2019 pandemic: Perioperative management and oncological and anatomical considerations. Future Oncol..

[34] Bell R., Weinberger D.M., Venkatesh M., Fernandes-Taylor S., Francis D.O., Davies L. (2024). Thyroid Cancer Incidence During 2020 to 2021 COVID-19 Variant Waves. JAMA Otolaryngol. Head Neck Surg..

[35] Vrinceanu D., Dumitru M., Marinescu A., Serboiu C., Musat G., Radulescu M., Popa-Cherecheanu M., Ciornei C., Manole F. (2024). Management of Giant Thyroid Tumors in Patients with Multiple Comorbidities in a Tertiary Head and Neck Surgery Center. Biomedicines.

[36] Prete A., Falcone M., Bottici V., Giani C., Tiseo G., Agate L., Matrone A., Cappagli V., Valerio L., Lorusso L. (2021). Thyroid cancer and COVID-19: Experience at one single thyroid disease referral center. Endocrine.

[37] Means J.H. (1937). The Thyroid and Its Diseases.

[38] Tran T.V., Kitahara C.M., de Vathaire F., Boutron-Ruault M.C., Journy N. (2020). Thyroid dysfunction and cancer incidence: A systematic review and meta-analysis. Endocr. Relat. Cancer.

[39] Chan Y.H., Young B.E., Fong S.W., Ding Y., Goh Y.S., Chee R.S., Tan S.Y., Kalimuddin S., Tambyah P.A., Leo Y.S. (2021). Differential Cytokine Responses in Hospitalized COVID-19 Patients Limit Efficacy of Remdesivir. Front. Immunol..

[40] Tian L., Pang Z., Li M., Lou F., An X., Zhu S., Song L., Tong Y., Fan H., Fan J. (2022). Molnupiravir and Its Antiviral Activity Against COVID-19. Front. Immunol..

[41] Panza F., Fiorino F., Pastore G., Fiaschi L., Tumbarello M., Medaglini D., Ciabattini A., Montagnani F., Fabbiani M. (2023). Does Nirmatrelvir/Ritonavir Influence the Immune Response against SARS-CoV-2, Independently from Rebound?. Microorganisms.

[42] Agarwal A., Hunt B.J., Stegemann M., Rochwerg B., Lamontagne F., Siemieniuk R.A., Agoritsas T., Askie L., Lytvyn L., Leo Y.-S. (2020). A living WHO guideline on drugs for covid-19. BMJ.

[43] Akinosoglou K., Schinas G., Gogos C. (2022). Oral Antiviral Treatment for COVID-19: A Comprehensive Review on Nirmatrelvir/Ritonavir. Viruses.

[44] Gordon C.J., Tchesnokov E.P., Schinazi R.F., Gotte M. (2021). Molnupiravir promotes SARS-CoV-2 mutagenesis via the RNA template. J. Biol. Chem..

[45] Elfiky A.A. (2020). Ribavirin, Remdesivir, Sofosbuvir, Galidesivir, and Tenofovir against SARS-CoV-2 RNA dependent RNA polymerase (RdRp): A molecular docking study. Life Sci..

[46] Ghazavi A., Ganji A., Keshavarzian N., Rabiemajd S., Mosayebi G. (2021). Cytokine profile and disease severity in patients with COVID-19. Cytokine.

[47] Sendur S.N., Oguz S.H., Unluturk U. (2023). COVID-19 vaccination and thyroiditis. Best Pract. Res. Clin. Endocrinol. Metab..

[48] Ludwig R.J., Anson M., Zirpel H., Thaci D., Olbrich H., Bieber K., Kridin K., Dempfle A., Curman P., Zhao S.S. (2025). A comprehensive review of methodologies and application to use the real-world data and analytics platform TriNetX. Front. Pharmacol..

