# Supplementary material for: Association Between COVID-19 Infection and Thyroid Cancer Development: A Retrospective Cohort Study Using the TriNetX Database
Source: Biomedicines. 2025 Aug 8;13(8):1933. doi: 10.3390/biomedicines13081933 (PMC12383963; doi:10.3390/biomedicines13081933)
Supplement: Supplementary file 1 [file biomedicines-13-01933-s001.zip › Supplementary File S1.pdf]

Supplementary File S1: Detailed query criteria of post-COVID and non-COVID group

Query Criteria for Cohort post-COVID

This query was run on the network Global Collaborative Network with 144 HCO(s) queried and 143 HCO(s) responded. A total of 114 provider(s) responded with patients. The final cohort included 2,311,130 patients who matched the query criteria listed in the table below.

**Ungrouped terms**				
must have		demographics	Age	Age (at least 18 years (most recent occurrence))
	and any of	demographics	UMLS:HL7V3.0:Gender:M	Male
		demographics	UMLS:HL7V3.0:Gender:F	Female
**COVID-19, no vaccination**				
must have	any of	diagnosis	UMLS:ICD10CM:U07.1	COVID-19
		diagnosis	UMLS:ICD10CM:U07.2	COVID-19, virus not identified (WHO)
		diagnosis	UMLS:ICD10CM:J12.82	Pneumonia due to coronavirus disease 2019
		laboratory	UMLS:LNC:94500-6	SARS-CoV-2 (COVID-19) RNA [Presence] in Respiratory specimen by NAA with probe detection (labResult: Positive)
		laboratory	UMLS:LNC:94309-2	SARS-CoV-2 (COVID-19) RNA [Presence] in Specimen by NAA with probe detection (labResult: Positive)
		laboratory	UMLS:LNC:94565-9	SARS-CoV-2 (COVID-19) RNA [Presence] in Nasopharynx by NAA with non-probe detection (labResult: Positive)
		laboratory	UMLS:LNC:94759-8	SARS-CoV-2 (COVID-19) RNA [Presence] in Nasopharynx by NAA with probe detection (labResult: Positive)
		laboratory	UMLS:LNC:95608-6	SARS-CoV-2 (COVID-19) RNA [Presence] in Respiratory specimen

				by NAA with non-probe detection (labResult: Positive)
		laboratory	UMLS:LNC:94845-5	SARS-CoV-2 (COVID-19) RNA [Presence] in Saliva (oral fluid) by NAA with probe detection (labResult: Positive)
		laboratory	UMLS:LNC:95406-5	SARS-CoV-2 (COVID-19) RNA [Presence] in Nose by NAA with probe detection (labResult: Positive)
cannot have		medication	NLM:CVX:208	COVID-19, mRNA, LNP-S, PF, 30 mcg/0.3 mL dose
	or	medication	NLM:CVX:207	COVID-19, mRNA, LNP-S, PF, 100 mcg/0.5mL dose or 50 mcg/0.25mL dose
	or	medication	NLM:CVX:212	COVID-19 vaccine, vector-nr, rS-Ad26, PF, 0.5 mL
	or	medication	NLM:RXNORM:OMOP5042939	COVID-19 vaccine
	or	medication	NLM:CVX:300	COVID-19, mRNA, LNP-S, bivalent, PF, 30 mcg/0.3 mL dose
	or	medication	NLM:CVX:217	COVID-19, mRNA, LNP-S, PF, 30 mcg/0.3 mL dose, tris-sucrose
	or	medication	NLM:CVX:229	COVID-19, mRNA, LNP-S, bivalent, PF, 50 mcg/0.5 mL or 25mcg/0.25 mL dose
	or	medication	NLM:CVX:218	COVID-19, mRNA, LNP-S, PF, 10 mcg/0.2 mL dose, tris-sucrose
	or	medication	NLM:CVX:520	COVID-19 mRNA, bivalent, original/Omicron BA.1, Non-US Vaccine Product, Pfizer-BioNTech
	or	medication	NLM:CVX:519	COVID-19 mRNA, bivalent, original/Omicron BA.1, Non-US Vaccine (Spikevax Bivalent), Moderna
	or	medication	NLM:CVX:301	COVID-19, mRNA, LNP-S, bivalent, PF, 10 mcg/0.2 mL dose
	or	medication	NLM:CVX:219	COVID-19, mRNA, LNP-S, PF, 3 mcg/0.2 mL dose, tris-sucrose

or	medication	NLM:CVX:228	COVID-19, mRNA, LNP-S, PF, pediatric 25 mcg/0.25 mL dose
or	medication	NLM:CVX:230	COVID-19, mRNA, LNP-S, bivalent booster, PF, 10 mcg/0.2 mL
or	medication	NLM:CVX:221	COVID-19, mRNA, LNP-S, PF, 50 mcg/0.5 mL dose
or	medication	NLM:CVX:210	COVID-19 vaccine, vector-nr, rS-ChAdOx1, PF, 0.5 mL
or	medication	NLM:CVX:302	COVID-19, mRNA, LNP-S, bivalent, PF, 3 mcg/0.2 mL dose
or	medication	NLM:CVX:511	COVID-19 IV Non-US Vaccine (CoronaVac, Sinovac)
or	medication	NLM:RXNORM:2468231	SARS-CoV-2 (COVID-19) vaccine, mRNA spike protein
or	procedure	UMLS:CPT:91300	Severe acute respiratory syndrome coronavirus 2 (SARS-CoV-2) (coronavirus disease [COVID-19]) vaccine, mRNA-LNP, spike protein, preservative free, 30 mcg/0.3 mL dosage, diluent reconstituted, for intramuscular use
or	procedure	UMLS:CPT:0001A	Immunization administration by intramuscular injection of severe acute respiratory syndrome coronavirus 2 (SARS-CoV-2) (coronavirus disease [COVID-19]) vaccine, mRNA-LNP, spike protein, preservative free, 30 mcg/0.3 mL dosage, diluent reconstituted; first dose
or	procedure	UMLS:CPT:0002A	Immunization administration by intramuscular injection of severe acute respiratory syndrome coronavirus 2 (SARS-CoV-2) (coronavirus disease [COVID-19]) vaccine, mRNA-LNP, spike protein, preservative free, 30 mcg/0.3 mL

			dosage, diluent reconstituted; second dose
or	procedure	UMLS:CPT:91301	Severe acute respiratory syndrome coronavirus 2 (SARS-CoV-2) (coronavirus disease [COVID-19]) vaccine, mRNA-LNP, spike protein, preservative free, 100 mcg/0.5 mL dosage, for intramuscular use
or	procedure	UMLS:CPT:0011A	Immunization administration by intramuscular injection of severe acute respiratory syndrome coronavirus 2 (SARS-CoV-2) (coronavirus disease [COVID-19]) vaccine, mRNA-LNP, spike protein, preservative free, 100 mcg/0.5 mL dosage; first dose
or	procedure	UMLS:CPT:0012A	Immunization administration by intramuscular injection of severe acute respiratory syndrome coronavirus 2 (SARS-CoV-2) (coronavirus disease [COVID-19]) vaccine, mRNA-LNP, spike protein, preservative free, 100 mcg/0.5 mL dosage; second dose
or	procedure	UMLS:SNOMED:840534001	Administration of SARS-CoV-2 antigen vaccine
or	medication	NLM:CVX:213	SARS-CoV-2 (COVID-19) Vaccine
or	procedure	UMLS:CPT:1036660	Immunization administration by intramuscular injection of severe acute respiratory syndrome coronavirus 2 (SARS-CoV-2) (coronavirus disease [COVID-19]) vaccine, mRNA-LNP, spike protein, preservative free, 30 mcg/0.3 mL dosage, diluent reconstituted
or	procedure	UMLS:CPT:1036663	Immunization administration by intramuscular injection of severe acute respiratory syndrome

			coronavirus 2 (SARS-CoV-2) (coronavirus disease [COVID-19]) vaccine, mRNA-LNP, spike protein, preservative free, 100 mcg/0.5 mL dosage
or	procedure	UMLS:CPT:0124A	Immunization administration by intramuscular injection of severe acute respiratory syndrome coronavirus 2 (SARS-CoV-2) (coronavirus disease [COVID-19]) vaccine, mRNA-LNP, bivalent spike protein, preservative free, 30 mcg/0.3 mL dosage, tris-sucrose formulation, booster dose
or	procedure	UMLS:CPT:0004A	Immunization administration by intramuscular injection of severe acute respiratory syndrome coronavirus 2 (SARS-CoV-2) (coronavirus disease [COVID-19]) vaccine, mRNA-LNP, spike protein, preservative free, 30 mcg/0.3 mL dosage, diluent reconstituted; booster dose
or	procedure	UMLS:CPT:0003A	Immunization administration by intramuscular injection of severe acute respiratory syndrome coronavirus 2 (SARS-CoV-2) (coronavirus disease [COVID-19]) vaccine, mRNA-LNP, spike protein, preservative free, 30 mcg/0.3 mL dosage, diluent reconstituted; third dose
or	procedure	UMLS:CPT:1037166	Immunization administration by intramuscular injection of severe acute respiratory syndrome coronavirus 2 (SARS-CoV-2) (coronavirus disease [COVID-19]) vaccine, mRNA-LNP, spike protein,

			preservative free, 30 mcg/0.3 mL dosage, tris-sucrose formulation
or	procedure	UMLS:CPT:0054A	Immunization administration by intramuscular injection of severe acute respiratory syndrome coronavirus 2 (SARS-CoV-2) (coronavirus disease [COVID-19]) vaccine, mRNA-LNP, spike protein, preservative free, 30 mcg/0.3 mL dosage, tris-sucrose formulation; booster dose
or	procedure	UMLS:CPT:0064A	Immunization administration by intramuscular injection of severe acute respiratory syndrome coronavirus 2 (SARS-CoV-2) (coronavirus disease [COVID-19]) vaccine, mRNA-LNP, spike protein, preservative free, 50 mcg/0.25 mL dosage, booster dose
or	procedure	UMLS:CPT:90480	Immunization administration by intramuscular injection of severe acute respiratory syndrome coronavirus 2 (SARS-CoV-2) (coronavirus disease [COVID-19]) vaccine, single dose
or	procedure	UMLS:CPT:1037171	Immunization administration by intramuscular injection of severe acute respiratory syndrome coronavirus 2 (SARS-CoV-2) (coronavirus disease [COVID-19]) vaccine, mRNA-LNP, spike protein, preservative free, 10 mcg/0.2 mL dosage, diluent reconstituted, tris-sucrose formulation
or	procedure	UMLS:CPT:0071A	Immunization administration by intramuscular injection of severe acute respiratory syndrome coronavirus 2 (SARS-CoV-2)

			(coronavirus disease [COVID-19]) vaccine, mRNA-LNP, spike protein, preservative free, 10 mcg/0.2 mL dosage, diluent reconstituted, tris-sucrose formulation; first dose
or	procedure	UMLS:CPT:0072A	Immunization administration by intramuscular injection of severe acute respiratory syndrome coronavirus 2 (SARS-CoV-2) (coronavirus disease [COVID-19]) vaccine, mRNA-LNP, spike protein, preservative free, 10 mcg/0.2 mL dosage, diluent reconstituted, tris-sucrose formulation; second dose
or	medication	NLM:RXNORM:2610319	SARS-CoV-2 (COVID-19) vaccine, mRNA-BNT162b2 0.05 MG/ML / SARS-CoV-2 (COVID-19) vaccine, mRNA-BNT162b2 OMICRON (BA.4/BA.5) 0.05 MG/ML Injectable Suspension
or	procedure	UMLS:CPT:91313	Severe acute respiratory syndrome coronavirus 2 (SARS-CoV-2) (coronavirus disease [COVID-19]) vaccine, mRNA-LNP, spike protein, bivalent, preservative free, 50 mcg/0.5 mL dosage, for intramuscular use
or	procedure	UMLS:CPT:0134A	Immunization administration by intramuscular injection of severe acute respiratory syndrome coronavirus 2 (SARS-CoV-2) (coronavirus disease [COVID-19]) vaccine, mRNA-LNP, spike protein, bivalent, preservative free, 50 mcg/0.5 mL dosage, booster dose
or	procedure	UMLS:CPT:1037175	Immunization administration by intramuscular injection of severe acute respiratory syndrome

			coronavirus 2 (SARS-CoV-2) (coronavirus disease [COVID-19]) vaccine, DNA, spike protein, adenovirus type 26 (Ad26) vector, preservative free, 5x1010 viral particles/0.5 mL dosage
or	medication	NLM:RXNORM:2610347	0.3 ML SARS-CoV-2 (COVID-19) vaccine, mRNA-BNT162b2 0.05 MG/ML / SARS-CoV-2 (COVID-19) vaccine, mRNA-BNT162b2 OMICRON (BA.4/BA.5) -1 MG/ML Injection
or	procedure	UMLS:CPT:1037228	Immunization administration by intramuscular injection of severe acute respiratory syndrome coronavirus 2 (SARS-CoV-2) (coronavirus disease [COVID-19]) vaccine, mRNA-LNP, spike protein, preservative free, 3 mcg/0.2 mL dosage, diluent reconstituted, tris-sucrose formulation
or	procedure	UMLS:CPT:0013A	Immunization administration by intramuscular injection of severe acute respiratory syndrome coronavirus 2 (SARS-CoV-2) (coronavirus disease [COVID-19]) vaccine, mRNA-LNP, spike protein, preservative free, 100 mcg/0.5 mL dosage; third dose
or	procedure	UMLS:CPT:0081A	Immunization administration by intramuscular injection of severe acute respiratory syndrome coronavirus 2 (SARS-CoV-2) (coronavirus disease [COVID-19]) vaccine, mRNA-LNP, spike protein, preservative free, 3 mcg/0.2 mL dosage, diluent reconstituted, tris-sucrose formulation; first dose

or	procedure	UMLS:CPT:0082A	Immunization administration by intramuscular injection of severe acute respiratory syndrome coronavirus 2 (SARS-CoV-2) (coronavirus disease [COVID-19]) vaccine, mRNA-LNP, spike protein, preservative free, 3 mcg/0.2 mL dosage, diluent reconstituted, tris-sucrose formulation; second dose
or	medication	NLM:RXNORM:2610328	SARS-CoV-2 (COVID-19) vaccine, mRNA-1273 0.05 MG/ML / SARS-CoV-2 (COVID-19) vaccine, mRNA-1273 OMICRON (BA.4/BA.5) 0.05 MG/ML Injectable Suspension
or	procedure	UMLS:CPT:0154A	Immunization administration by intramuscular injection of severe acute respiratory syndrome coronavirus 2 (SARS-CoV-2) (coronavirus disease [COVID-19]) vaccine, mRNA-LNP, bivalent spike protein, preservative free, 10 mcg/0.2 mL dosage, diluent reconstituted, tris-sucrose formulation, booster dose
or	procedure	UMLS:CPT:0053A	Immunization administration by intramuscular injection of severe acute respiratory syndrome coronavirus 2 (SARS-CoV-2) (coronavirus disease [COVID-19]) vaccine, mRNA-LNP, spike protein, preservative free, 30 mcg/0.3 mL dosage, tris-sucrose formulation; third dose
or	procedure	UMLS:CPT:1037332	Immunization administration by intramuscular injection of severe acute respiratory syndrome coronavirus 2 (SARS-CoV-2) (coronavirus disease [COVID-19])

			vaccine, mRNA-LNP, spike protein, preservative free, 25 mcg/0.25 mL dosage
or	procedure	UMLS:CPT:0052A	Immunization administration by intramuscular injection of severe acute respiratory syndrome coronavirus 2 (SARS-CoV-2) (coronavirus disease [COVID-19]) vaccine, mRNA-LNP, spike protein, preservative free, 30 mcg/0.3 mL dosage, tris-sucrose formulation; second dose
or	procedure	UMLS:CPT:0111A	Immunization administration by intramuscular injection of severe acute respiratory syndrome coronavirus 2 (SARS-CoV-2) (coronavirus disease [COVID-19]) vaccine, mRNA-LNP, spike protein, preservative free, 25 mcg/0.25 mL dosage; first dose
or	procedure	UMLS:CPT:0051A	Immunization administration by intramuscular injection of severe acute respiratory syndrome coronavirus 2 (SARS-CoV-2) (coronavirus disease [COVID-19]) vaccine, mRNA-LNP, spike protein, preservative free, 30 mcg/0.3 mL dosage, tris-sucrose formulation; first dose
or	procedure	UMLS:CPT:91311	Severe acute respiratory syndrome coronavirus 2 (SARS-CoV-2) (coronavirus disease [COVID-19]) vaccine, mRNA-LNP, spike protein, preservative free, 25 mcg/0.25 mL dosage, for intramuscular use
or	procedure	UMLS:CPT:0074A	Immunization administration by intramuscular injection of severe acute respiratory syndrome

			coronavirus 2 (SARS-CoV-2) (coronavirus disease [COVID-19]) vaccine, mRNA-LNP, spike protein, preservative free, 10 mcg/0.2 mL dosage, diluent reconstituted, tris-sucrose formulation; booster dose
or	procedure	UMLS:CPT:0112A	Immunization administration by intramuscular injection of severe acute respiratory syndrome coronavirus 2 (SARS-CoV-2) (coronavirus disease [COVID-19]) vaccine, mRNA-LNP, spike protein, preservative free, 25 mcg/0.25 mL dosage; second dose
or	procedure	UMLS:CPT:0083A	Immunization administration by intramuscular injection of severe acute respiratory syndrome coronavirus 2 (SARS-CoV-2) (coronavirus disease [COVID-19]) vaccine, mRNA-LNP, spike protein, preservative free, 3 mcg/0.2 mL dosage, diluent reconstituted, tris-sucrose formulation; third dose
or	procedure	UMLS:CPT:0073A	Immunization administration by intramuscular injection of severe acute respiratory syndrome coronavirus 2 (SARS-CoV-2) (coronavirus disease [COVID-19]) vaccine, mRNA-LNP, spike protein, preservative free, 10 mcg/0.2 mL dosage, diluent reconstituted, tris-sucrose formulation; third dose
or	procedure	UMLS:CPT:0173A	Immunization administration by intramuscular injection of severe acute respiratory syndrome coronavirus 2 (SARS-CoV-2) (coronavirus disease [COVID-19]) vaccine, mRNA-LNP, bivalent spike

			protein, preservative free, 3 mcg/0.2 mL dosage, diluent reconstituted, tris-sucrose formulation, third dose
or	procedure	UMLS:CPT:0164A	Immunization administration by intramuscular injection of severe acute respiratory syndrome coronavirus 2 (SARS-CoV-2) (coronavirus disease [COVID-19]) vaccine, mRNA-LNP, spike protein, bivalent, preservative free, 10 mcg/0.2 mL dosage, booster dose
or	procedure	UMLS:CPT:1037838	Immunization administration by intramuscular injection of severe acute respiratory syndrome coronavirus 2 (SARS-CoV-2) (coronavirus disease [COVID-19]) vaccine, mRNA-LNP, spike protein, preservative free, 50 mcg/0.5 mL dosage
or	procedure	UMLS:CPT:0094A	Immunization administration by intramuscular injection of severe acute respiratory syndrome coronavirus 2 (SARS-CoV-2) (coronavirus disease [COVID-19]) vaccine, mRNA-LNP, spike protein, preservative free, 50 mcg/0.5 mL dosage; booster dose, when administered to individuals 18 years and over
or	procedure	UMLS:CPT:0034A	Immunization administration by intramuscular injection of severe acute respiratory syndrome coronavirus 2 (SARS-CoV-2) (coronavirus disease [COVID-19]) vaccine, DNA, spike protein, adenovirus type 26 (Ad26) vector, preservative free, 5x1010 viral

			particles/0.5 mL dosage; booster dose
or	procedure	UMLS:CPT:0144A	Immunization administration by intramuscular injection of severe acute respiratory syndrome coronavirus 2 (SARS-CoV-2) (coronavirus disease [COVID-19]) vaccine, mRNA-LNP, spike protein, bivalent, preservative free, 25 mcg/0.25 mL dosage, booster dose
or	procedure	UMLS:CPT:0091A	Immunization administration by intramuscular injection of severe acute respiratory syndrome coronavirus 2 (SARS-CoV-2) (coronavirus disease [COVID-19]) vaccine, mRNA-LNP, spike protein, preservative free, 50 mcg/0.5 mL dosage; first dose, when administered to individuals 6 through 11 years
or	procedure	UMLS:CPT:0174A	Immunization administration by intramuscular injection of severe acute respiratory syndrome coronavirus 2 (SARS-CoV-2) (coronavirus disease [COVID-19]) vaccine, mRNA-LNP, bivalent spike protein, preservative free, 3 mcg/0.2 mL dosage, diluent reconstituted, tris-sucrose formulation, booster
or	procedure	UMLS:CPT:0092A	Immunization administration by intramuscular injection of severe acute respiratory syndrome coronavirus 2 (SARS-CoV-2) (coronavirus disease [COVID-19]) vaccine, mRNA-LNP, spike protein, preservative free, 50 mcg/0.5 mL dosage; second dose, when

			administered to individuals 6 through 11 years
or	procedure	UMLS:CPT:1036682	Immunization administration by intramuscular injection of severe acute respiratory syndrome coronavirus 2 (SARS-CoV-2) (coronavirus disease [COVID-19]) vaccine, recombinant spike protein nanoparticle, saponin-based adjuvant, preservative free, 5 mcg/0.5 mL dosage
or	procedure	UMLS:CPT:0041A	Immunization administration by intramuscular injection of severe acute respiratory syndrome coronavirus 2 (SARS-CoV-2) (coronavirus disease [COVID-19]) vaccine, recombinant spike protein nanoparticle, saponin-based adjuvant, preservative free, 5 mcg/0.5 mL dosage; first dose
or	procedure	UMLS:CPT:0113A	Immunization administration by intramuscular injection of severe acute respiratory syndrome coronavirus 2 (SARS-CoV-2) (coronavirus disease [COVID-19]) vaccine, mRNA-LNP, spike protein, preservative free, 25 mcg/0.25 mL dosage; third dose
or	procedure	UMLS:CPT:0042A	Immunization administration by intramuscular injection of severe acute respiratory syndrome coronavirus 2 (SARS-CoV-2) (coronavirus disease [COVID-19]) vaccine, recombinant spike protein nanoparticle, saponin-based adjuvant, preservative free, 5 mcg/0.5 mL dosage; second dose

	or	procedure	UMLS:CPT:0093A	Immunization administration by intramuscular injection of severe acute respiratory syndrome coronavirus 2 (SARS-CoV-2) (coronavirus disease [COVID-19]) vaccine, mRNA-LNP, spike protein, preservative free, 50 mcg/0.5 mL dosage; third dose, when administered to individuals 6 through 11 years
	or	procedure	UMLS:CPT:1036666	Immunization administration by intramuscular injection of severe acute respiratory syndrome coronavirus 2 (SARS-CoV-2) (coronavirus disease [COVID-19]) vaccine, DNA, spike protein, chimpanzee adenovirus Oxford 1 (ChAdOx1) vector, preservative free, 5x1010 viral particles/0.5 mL dosage
	or	procedure	UMLS:CPT:0044A	Immunization administration by intramuscular injection of severe acute respiratory syndrome coronavirus 2 (SARS-CoV-2) (coronavirus disease [COVID-19]) vaccine, recombinant spike protein nanoparticle, saponin-based adjuvant, preservative free, 5 mcg/0.5mL dosage; booster
date constraint		The terms in this group occurred between Dec 1, 2019 and Dec 31, 2023		
**Group 2**				
**Group 2A COVID-19**				
must have	any of	diagnosis	UMLS:ICD10CM:U07.1	COVID-19
		diagnosis	UMLS:ICD10CM:U07.2	COVID-19, virus not identified (WHO)
		diagnosis	UMLS:ICD10CM:J12.82	Pneumonia due to coronavirus disease 2019
		laboratory	UMLS:LNC:94500-6	SARS-CoV-2 (COVID-19) RNA [Presence] in Respiratory specimen

				by NAA with probe detection (labResult: Positive)
		laboratory	UMLS:LNC:94309-2	SARS-CoV-2 (COVID-19) RNA [Presence] in Specimen by NAA with probe detection (labResult: Positive)
		laboratory	UMLS:LNC:94565-9	SARS-CoV-2 (COVID-19) RNA [Presence] in Nasopharynx by NAA with non-probe detection (labResult: Positive)
		laboratory	UMLS:LNC:94759-8	SARS-CoV-2 (COVID-19) RNA [Presence] in Nasopharynx by NAA with probe detection (labResult: Positive)
		laboratory	UMLS:LNC:95608-6	SARS-CoV-2 (COVID-19) RNA [Presence] in Respiratory specimen by NAA with non-probe detection (labResult: Positive)
		laboratory	UMLS:LNC:94845-5	SARS-CoV-2 (COVID-19) RNA [Presence] in Saliva (oral fluid) by NAA with probe detection (labResult: Positive)
		laboratory	UMLS:LNC:95406-5	SARS-CoV-2 (COVID-19) RNA [Presence] in Nose by NAA with probe detection (labResult: Positive)
	and	visit	TNX:Visit	Visit (Data Source: TriNetX)
date constraint		The terms in this group occurred at any time		
event relationship		Any instance of thyroid cancer history occurred at least 1 day before the first instance of COVID-19		
**Group 2B thyroid cancer history**				
cannot have		diagnosis	UMLS:ICD10CM:C73	Malignant neoplasm of thyroid gland
	or	diagnosis	UMLS:ICD10CM:Z85.850	Personal history of malignant neoplasm of thyroid

Query Criteria for Cohort non-COVID

This query was run on the network Global Collaborative Network with 147 HCO(s) queried and 147 HCO(s) responded. A total of 130 provider(s) responded with patients. The final

cohort included 30,680,965 patients who matched the query criteria listed in the table below.

**Ungrouped terms**				
must have		demographics	Age	Age (at least 18 years (most recent occurrence))
	and any of	demographics	UMLS:HL7V3.0:Gender:M	Male
		demographics	UMLS:HL7V3.0:Gender:F	Female
**Group 1**				
**COVID-19 negative, no vaccination**				
must have		visit	TNX:Visit	Visit
cannot have		medication	NLM:CVX:208	COVID-19, mRNA, LNP-S, PF, 30 mcg/0.3 mL dose
	or	medication	NLM:CVX:207	COVID-19, mRNA, LNP-S, PF, 100 mcg/0.5mL dose or 50 mcg/0.25mL dose
	or	medication	NLM:CVX:212	COVID-19 vaccine, vector-nr, rS-Ad26, PF, 0.5 mL
	or	medication	NLM:RXNORM:OMOP5042939	COVID-19 vaccine
	or	medication	NLM:CVX:300	COVID-19, mRNA, LNP-S, bivalent, PF, 30 mcg/0.3 mL dose
	or	medication	NLM:CVX:217	COVID-19, mRNA, LNP-S, PF, 30 mcg/0.3 mL dose, tris-sucrose
	or	medication	NLM:CVX:229	COVID-19, mRNA, LNP-S, bivalent, PF, 50 mcg/0.5 mL or 25mcg/0.25 mL dose
	or	medication	NLM:CVX:218	COVID-19, mRNA, LNP-S, PF, 10 mcg/0.2 mL dose, tris-sucrose
	or	medication	NLM:CVX:520	COVID-19 mRNA, bivalent, original/Omicron BA.1, Non-US Vaccine Product, Pfizer-BioNTech
	or	medication	NLM:CVX:519	COVID-19 mRNA, bivalent, original/Omicron BA.1, Non-US Vaccine (Spikevax Bivalent), Moderna
	or	medication	NLM:CVX:301	COVID-19, mRNA, LNP-S, bivalent, PF, 10 mcg/0.2 mL dose

or	medication	NLM:CVX:219	COVID-19, mRNA, LNP-S, PF, 3 mcg/0.2 mL dose, tris-sucrose
or	medication	NLM:CVX:228	COVID-19, mRNA, LNP-S, PF, pediatric 25 mcg/0.25 mL dose
or	medication	NLM:CVX:230	COVID-19, mRNA, LNP-S, bivalent booster, PF, 10 mcg/0.2 mL
or	medication	NLM:CVX:221	COVID-19, mRNA, LNP-S, PF, 50 mcg/0.5 mL dose
or	medication	NLM:CVX:210	COVID-19 vaccine, vector-nr, rS-ChAdOx1, PF, 0.5 mL
or	medication	NLM:CVX:302	COVID-19, mRNA, LNP-S, bivalent, PF, 3 mcg/0.2 mL dose
or	medication	NLM:CVX:511	COVID-19 IV Non-US Vaccine (CoronaVac, Sinovac)
or	medication	NLM:RXNORM:2468231	SARS-CoV-2 (COVID-19) vaccine, mRNA spike protein
or	procedure	UMLS:CPT:91300	Severe acute respiratory syndrome coronavirus 2 (SARS-CoV-2) (coronavirus disease [COVID-19]) vaccine, mRNA-LNP, spike protein, preservative free, 30 mcg/0.3 mL dosage, diluent reconstituted, for intramuscular use
or	procedure	UMLS:CPT:0001A	Immunization administration by intramuscular injection of severe acute respiratory syndrome coronavirus 2 (SARS-CoV-2) (coronavirus disease [COVID-19]) vaccine, mRNA-LNP, spike protein, preservative free, 30 mcg/0.3 mL dosage, diluent reconstituted; first dose
or	procedure	UMLS:CPT:0002A	Immunization administration by intramuscular injection of severe acute respiratory syndrome coronavirus 2 (SARS-CoV-2) (coronavirus disease [COVID-19]) vaccine, mRNA-LNP, spike protein,

			preservative free, 30 mcg/0.3 mL dosage, diluent reconstituted; second dose
or	procedure	UMLS:CPT:91301	Severe acute respiratory syndrome coronavirus 2 (SARS-CoV-2) (coronavirus disease [COVID-19]) vaccine, mRNA-LNP, spike protein, preservative free, 100 mcg/0.5 mL dosage, for intramuscular use
or	procedure	UMLS:CPT:0011A	Immunization administration by intramuscular injection of severe acute respiratory syndrome coronavirus 2 (SARS-CoV-2) (coronavirus disease [COVID-19]) vaccine, mRNA-LNP, spike protein, preservative free, 100 mcg/0.5 mL dosage; first dose
or	procedure	UMLS:CPT:0012A	Immunization administration by intramuscular injection of severe acute respiratory syndrome coronavirus 2 (SARS-CoV-2) (coronavirus disease [COVID-19]) vaccine, mRNA-LNP, spike protein, preservative free, 100 mcg/0.5 mL dosage; second dose
or	procedure	UMLS:SNOMED:840534001	Administration of SARS-CoV-2 antigen vaccine
or	medication	NLM:CVX:213	SARS-CoV-2 (COVID-19) Vaccine
or	procedure	UMLS:CPT:1036660	Immunization administration by intramuscular injection of severe acute respiratory syndrome coronavirus 2 (SARS-CoV-2) (coronavirus disease [COVID-19]) vaccine, mRNA-LNP, spike protein, preservative free, 30 mcg/0.3 mL dosage, diluent reconstituted
or	procedure	UMLS:CPT:1036663	Immunization administration by intramuscular injection of severe

			acute respiratory syndrome coronavirus 2 (SARS-CoV-2) (coronavirus disease [COVID-19]) vaccine, mRNA-LNP, spike protein, preservative free, 100 mcg/0.5 mL dosage
or	procedure	UMLS:CPT:0124A	Immunization administration by intramuscular injection of severe acute respiratory syndrome coronavirus 2 (SARS-CoV-2) (coronavirus disease [COVID-19]) vaccine, mRNA-LNP, bivalent spike protein, preservative free, 30 mcg/0.3 mL dosage, tris-sucrose formulation, booster dose
or	procedure	UMLS:CPT:0004A	Immunization administration by intramuscular injection of severe acute respiratory syndrome coronavirus 2 (SARS-CoV-2) (coronavirus disease [COVID-19]) vaccine, mRNA-LNP, spike protein, preservative free, 30 mcg/0.3 mL dosage, diluent reconstituted; booster dose
or	procedure	UMLS:CPT:0003A	Immunization administration by intramuscular injection of severe acute respiratory syndrome coronavirus 2 (SARS-CoV-2) (coronavirus disease [COVID-19]) vaccine, mRNA-LNP, spike protein, preservative free, 30 mcg/0.3 mL dosage, diluent reconstituted; third dose
or	procedure	UMLS:CPT:1037166	Immunization administration by intramuscular injection of severe acute respiratory syndrome coronavirus 2 (SARS-CoV-2) (coronavirus disease [COVID-19])

			vaccine, mRNA-LNP, spike protein, preservative free, 30 mcg/0.3 mL dosage, tris-sucrose formulation
or	procedure	UMLS:CPT:0054A	Immunization administration by intramuscular injection of severe acute respiratory syndrome coronavirus 2 (SARS-CoV-2) (coronavirus disease [COVID-19]) vaccine, mRNA-LNP, spike protein, preservative free, 30 mcg/0.3 mL dosage, tris-sucrose formulation; booster dose
or	procedure	UMLS:CPT:0064A	Immunization administration by intramuscular injection of severe acute respiratory syndrome coronavirus 2 (SARS-CoV-2) (coronavirus disease [COVID-19]) vaccine, mRNA-LNP, spike protein, preservative free, 50 mcg/0.25 mL dosage, booster dose
or	procedure	UMLS:CPT:90480	Immunization administration by intramuscular injection of severe acute respiratory syndrome coronavirus 2 (SARS-CoV-2) (coronavirus disease [COVID-19]) vaccine, single dose
or	procedure	UMLS:CPT:1037171	Immunization administration by intramuscular injection of severe acute respiratory syndrome coronavirus 2 (SARS-CoV-2) (coronavirus disease [COVID-19]) vaccine, mRNA-LNP, spike protein, preservative free, 10 mcg/0.2 mL dosage, diluent reconstituted, tris-sucrose formulation
or	procedure	UMLS:CPT:0071A	Immunization administration by intramuscular injection of severe acute respiratory syndrome

			coronavirus 2 (SARS-CoV-2) (coronavirus disease [COVID-19]) vaccine, mRNA-LNP, spike protein, preservative free, 10 mcg/0.2 mL dosage, diluent reconstituted, tris-sucrose formulation; first dose
or	procedure	UMLS:CPT:0072A	Immunization administration by intramuscular injection of severe acute respiratory syndrome coronavirus 2 (SARS-CoV-2) (coronavirus disease [COVID-19]) vaccine, mRNA-LNP, spike protein, preservative free, 10 mcg/0.2 mL dosage, diluent reconstituted, tris-sucrose formulation; second dose
or	medication	NLM:RXNORM:2610319	SARS-CoV-2 (COVID-19) vaccine, mRNA-BNT162b2 0.05 MG/ML / SARS-CoV-2 (COVID-19) vaccine, mRNA-BNT162b2 OMICRON (BA.4/BA.5) 0.05 MG/ML Injectable Suspension
or	procedure	UMLS:CPT:91313	Severe acute respiratory syndrome coronavirus 2 (SARS-CoV-2) (coronavirus disease [COVID-19]) vaccine, mRNA-LNP, spike protein, bivalent, preservative free, 50 mcg/0.5 mL dosage, for intramuscular use
or	procedure	UMLS:CPT:0134A	Immunization administration by intramuscular injection of severe acute respiratory syndrome coronavirus 2 (SARS-CoV-2) (coronavirus disease [COVID-19]) vaccine, mRNA-LNP, spike protein, bivalent, preservative free, 50 mcg/0.5 mL dosage, booster dose
or	procedure	UMLS:CPT:1037175	Immunization administration by intramuscular injection of severe

			acute respiratory syndrome coronavirus 2 (SARS-CoV-2) (coronavirus disease [COVID-19]) vaccine, DNA, spike protein, adenovirus type 26 (Ad26) vector, preservative free, 5x1010 viral particles/0.5 mL dosage
or	medication	NLM:RXNORM:2610347	0.3 ML SARS-CoV-2 (COVID-19) vaccine, mRNA-BNT162b2 0.05 MG/ML / SARS-CoV-2 (COVID-19) vaccine, mRNA-BNT162b2 OMICRON (BA.4/BA.5) -1 MG/ML Injection
or	procedure	UMLS:CPT:1037228	Immunization administration by intramuscular injection of severe acute respiratory syndrome coronavirus 2 (SARS-CoV-2) (coronavirus disease [COVID-19]) vaccine, mRNA-LNP, spike protein, preservative free, 3 mcg/0.2 mL dosage, diluent reconstituted, tris-sucrose formulation
or	procedure	UMLS:CPT:0013A	Immunization administration by intramuscular injection of severe acute respiratory syndrome coronavirus 2 (SARS-CoV-2) (coronavirus disease [COVID-19]) vaccine, mRNA-LNP, spike protein, preservative free, 100 mcg/0.5 mL dosage; third dose
or	procedure	UMLS:CPT:0081A	Immunization administration by intramuscular injection of severe acute respiratory syndrome coronavirus 2 (SARS-CoV-2) (coronavirus disease [COVID-19]) vaccine, mRNA-LNP, spike protein, preservative free, 3 mcg/0.2 mL dosage, diluent reconstituted, tris-sucrose formulation; first dose

or	procedure	UMLS:CPT:0082A	Immunization administration by intramuscular injection of severe acute respiratory syndrome coronavirus 2 (SARS-CoV-2) (coronavirus disease [COVID-19]) vaccine, mRNA-LNP, spike protein, preservative free, 3 mcg/0.2 mL dosage, diluent reconstituted, tris-sucrose formulation; second dose
or	medication	NLM:RXNORM:2610328	SARS-CoV-2 (COVID-19) vaccine, mRNA-1273 0.05 MG/ML / SARS-CoV-2 (COVID-19) vaccine, mRNA-1273 OMICRON (BA.4/BA.5) 0.05 MG/ML Injectable Suspension
or	procedure	UMLS:CPT:0154A	Immunization administration by intramuscular injection of severe acute respiratory syndrome coronavirus 2 (SARS-CoV-2) (coronavirus disease [COVID-19]) vaccine, mRNA-LNP, bivalent spike protein, preservative free, 10 mcg/0.2 mL dosage, diluent reconstituted, tris-sucrose formulation, booster dose
or	procedure	UMLS:CPT:0053A	Immunization administration by intramuscular injection of severe acute respiratory syndrome coronavirus 2 (SARS-CoV-2) (coronavirus disease [COVID-19]) vaccine, mRNA-LNP, spike protein, preservative free, 30 mcg/0.3 mL dosage, tris-sucrose formulation; third dose
or	procedure	UMLS:CPT:1037332	Immunization administration by intramuscular injection of severe acute respiratory syndrome coronavirus 2 (SARS-CoV-2) (coronavirus disease [COVID-19])

			vaccine, mRNA-LNP, spike protein, preservative free, 25 mcg/0.25 mL dosage
or	procedure	UMLS:CPT:0052A	Immunization administration by intramuscular injection of severe acute respiratory syndrome coronavirus 2 (SARS-CoV-2) (coronavirus disease [COVID-19]) vaccine, mRNA-LNP, spike protein, preservative free, 30 mcg/0.3 mL dosage, tris-sucrose formulation; second dose
or	procedure	UMLS:CPT:0111A	Immunization administration by intramuscular injection of severe acute respiratory syndrome coronavirus 2 (SARS-CoV-2) (coronavirus disease [COVID-19]) vaccine, mRNA-LNP, spike protein, preservative free, 25 mcg/0.25 mL dosage; first dose
or	procedure	UMLS:CPT:0051A	Immunization administration by intramuscular injection of severe acute respiratory syndrome coronavirus 2 (SARS-CoV-2) (coronavirus disease [COVID-19]) vaccine, mRNA-LNP, spike protein, preservative free, 30 mcg/0.3 mL dosage, tris-sucrose formulation; first dose
or	procedure	UMLS:CPT:91311	Severe acute respiratory syndrome coronavirus 2 (SARS-CoV-2) (coronavirus disease [COVID-19]) vaccine, mRNA-LNP, spike protein, preservative free, 25 mcg/0.25 mL dosage, for intramuscular use
or	procedure	UMLS:CPT:0074A	Immunization administration by intramuscular injection of severe acute respiratory syndrome

			coronavirus 2 (SARS-CoV-2) (coronavirus disease [COVID-19]) vaccine, mRNA-LNP, spike protein, preservative free, 10 mcg/0.2 mL dosage, diluent reconstituted, tris-sucrose formulation; booster dose
or	procedure	UMLS:CPT:0112A	Immunization administration by intramuscular injection of severe acute respiratory syndrome coronavirus 2 (SARS-CoV-2) (coronavirus disease [COVID-19]) vaccine, mRNA-LNP, spike protein, preservative free, 25 mcg/0.25 mL dosage; second dose
or	procedure	UMLS:CPT:0083A	Immunization administration by intramuscular injection of severe acute respiratory syndrome coronavirus 2 (SARS-CoV-2) (coronavirus disease [COVID-19]) vaccine, mRNA-LNP, spike protein, preservative free, 3 mcg/0.2 mL dosage, diluent reconstituted, tris-sucrose formulation; third dose
or	procedure	UMLS:CPT:0073A	Immunization administration by intramuscular injection of severe acute respiratory syndrome coronavirus 2 (SARS-CoV-2) (coronavirus disease [COVID-19]) vaccine, mRNA-LNP, spike protein, preservative free, 10 mcg/0.2 mL dosage, diluent reconstituted, tris-sucrose formulation; third dose
or	procedure	UMLS:CPT:0173A	Immunization administration by intramuscular injection of severe acute respiratory syndrome coronavirus 2 (SARS-CoV-2) (coronavirus disease [COVID-19]) vaccine, mRNA-LNP, bivalent spike

			protein, preservative free, 3 mcg/0.2 mL dosage, diluent reconstituted, tris-sucrose formulation, third dose
or	procedure	UMLS:CPT:0164A	Immunization administration by intramuscular injection of severe acute respiratory syndrome coronavirus 2 (SARS-CoV-2) (coronavirus disease [COVID-19]) vaccine, mRNA-LNP, spike protein, bivalent, preservative free, 10 mcg/0.2 mL dosage, booster dose
or	procedure	UMLS:CPT:1037838	Immunization administration by intramuscular injection of severe acute respiratory syndrome coronavirus 2 (SARS-CoV-2) (coronavirus disease [COVID-19]) vaccine, mRNA-LNP, spike protein, preservative free, 50 mcg/0.5 mL dosage
or	procedure	UMLS:CPT:0094A	Immunization administration by intramuscular injection of severe acute respiratory syndrome coronavirus 2 (SARS-CoV-2) (coronavirus disease [COVID-19]) vaccine, mRNA-LNP, spike protein, preservative free, 50 mcg/0.5 mL dosage; booster dose, when administered to individuals 18 years and over
or	procedure	UMLS:CPT:0034A	Immunization administration by intramuscular injection of severe acute respiratory syndrome coronavirus 2 (SARS-CoV-2) (coronavirus disease [COVID-19]) vaccine, DNA, spike protein, adenovirus type 26 (Ad26) vector, preservative free, 5x1010 viral

			particles/0.5 mL dosage; booster dose
or	procedure	UMLS:CPT:0144A	Immunization administration by intramuscular injection of severe acute respiratory syndrome coronavirus 2 (SARS-CoV-2) (coronavirus disease [COVID-19]) vaccine, mRNA-LNP, spike protein, bivalent, preservative free, 25 mcg/0.25 mL dosage, booster dose
or	procedure	UMLS:CPT:0091A	Immunization administration by intramuscular injection of severe acute respiratory syndrome coronavirus 2 (SARS-CoV-2) (coronavirus disease [COVID-19]) vaccine, mRNA-LNP, spike protein, preservative free, 50 mcg/0.5 mL dosage; first dose, when administered to individuals 6 through 11 years
or	procedure	UMLS:CPT:0174A	Immunization administration by intramuscular injection of severe acute respiratory syndrome coronavirus 2 (SARS-CoV-2) (coronavirus disease [COVID-19]) vaccine, mRNA-LNP, bivalent spike protein, preservative free, 3 mcg/0.2 mL dosage, diluent reconstituted, tris-sucrose formulation, booster
or	procedure	UMLS:CPT:0092A	Immunization administration by intramuscular injection of severe acute respiratory syndrome coronavirus 2 (SARS-CoV-2) (coronavirus disease [COVID-19]) vaccine, mRNA-LNP, spike protein, preservative free, 50 mcg/0.5 mL dosage; second dose, when

			administered to individuals 6 through 11 years
or	procedure	UMLS:CPT:1036682	Immunization administration by intramuscular injection of severe acute respiratory syndrome coronavirus 2 (SARS-CoV-2) (coronavirus disease [COVID-19]) vaccine, recombinant spike protein nanoparticle, saponin-based adjuvant, preservative free, 5 mcg/0.5 mL dosage
or	procedure	UMLS:CPT:0041A	Immunization administration by intramuscular injection of severe acute respiratory syndrome coronavirus 2 (SARS-CoV-2) (coronavirus disease [COVID-19]) vaccine, recombinant spike protein nanoparticle, saponin-based adjuvant, preservative free, 5 mcg/0.5 mL dosage; first dose
or	procedure	UMLS:CPT:0113A	Immunization administration by intramuscular injection of severe acute respiratory syndrome coronavirus 2 (SARS-CoV-2) (coronavirus disease [COVID-19]) vaccine, mRNA-LNP, spike protein, preservative free, 25 mcg/0.25 mL dosage; third dose
or	procedure	UMLS:CPT:0042A	Immunization administration by intramuscular injection of severe acute respiratory syndrome coronavirus 2 (SARS-CoV-2) (coronavirus disease [COVID-19]) vaccine, recombinant spike protein nanoparticle, saponin-based adjuvant, preservative free, 5 mcg/0.5 mL dosage; second dose

	or	procedure	UMLS:CPT:0093A	Immunization administration by intramuscular injection of severe acute respiratory syndrome coronavirus 2 (SARS-CoV-2) (coronavirus disease [COVID-19]) vaccine, mRNA-LNP, spike protein, preservative free, 50 mcg/0.5 mL dosage; third dose, when administered to individuals 6 through 11 years
	or	procedure	UMLS:CPT:1036666	Immunization administration by intramuscular injection of severe acute respiratory syndrome coronavirus 2 (SARS-CoV-2) (coronavirus disease [COVID-19]) vaccine, DNA, spike protein, chimpanzee adenovirus Oxford 1 (ChAdOx1) vector, preservative free, 5x1010 viral particles/0.5 mL dosage
	or	procedure	UMLS:CPT:0044A	Immunization administration by intramuscular injection of severe acute respiratory syndrome coronavirus 2 (SARS-CoV-2) (coronavirus disease [COVID-19]) vaccine, recombinant spike protein nanoparticle, saponin-based adjuvant, preservative free, 5 mcg/0.5mL dosage; booster
date constraint		The terms in this group occurred between Dec 1, 2019 and Dec 31, 2023		
**Group 2**				
**Group 2A COVID-19 negative**				
must have		visit	TNX:Visit	Visit (Data Source: TriNetX)
cannot have		diagnosis	UMLS:ICD10CM:U07.1	COVID-19
	or	laboratory	UMLS:LNC:95406-5	SARS-CoV-2 (COVID-19) RNA [Presence] in Nose by NAA with probe detection (labResult: Positive)
	or	laboratory	UMLS:LNC:94845-5	SARS-CoV-2 (COVID-19) RNA [Presence] in Saliva (oral fluid) by NAA

			with probe detection (labResult: Positive)
or	laboratory	UMLS:LNC:95608-6	SARS-CoV-2 (COVID-19) RNA [Presence] in Respiratory specimen by NAA with non-probe detection (labResult: Positive)
or	laboratory	UMLS:LNC:94759-8	SARS-CoV-2 (COVID-19) RNA [Presence] in Nasopharynx by NAA with probe detection (labResult: Positive)
or	laboratory	UMLS:LNC:94565-9	SARS-CoV-2 (COVID-19) RNA [Presence] in Nasopharynx by NAA with non-probe detection (labResult: Positive)
or	laboratory	UMLS:LNC:94309-2	SARS-CoV-2 (COVID-19) RNA [Presence] in Specimen by NAA with probe detection (labResult: Positive)
or	laboratory	UMLS:LNC:94500-6	SARS-CoV-2 (COVID-19) RNA [Presence] in Respiratory specimen by NAA with probe detection (labResult: Positive)
or	diagnosis	UMLS:ICD10CM:U07.2	COVID-19, virus not identified (WHO)
or	diagnosis	UMLS:ICD10CM:J12.82	Pneumonia due to coronavirus disease 2019
date constraint	The terms in this group occurred at any time		
event relationship	Any instance of thyroid cancer history occurred at least 1 day before the first instance of COVID-19 negative		
**Group 2B thyroid cancer history**			
cannot have	medication	NLM:CVX:208	COVID-19, mRNA, LNP-S, PF, 30 mcg/0.3 mL dose
or	medication	NLM:CVX:207	COVID-19, mRNA, LNP-S, PF, 100 mcg/0.5mL dose or 50 mcg/0.25mL dose
or	medication	NLM:CVX:300	COVID-19, mRNA, LNP-S, bivalent, PF, 30 mcg/0.3 mL dose
or	medication	NLM:CVX:217	COVID-19, mRNA, LNP-S, PF, 30 mcg/0.3 mL dose, tris-sucrose

or	medication	NLM:RXNORM:OMOP5042939	COVID-19 vaccine
or	medication	NLM:CVX:229	COVID-19, mRNA, LNP-S, bivalent, PF, 50 mcg/0.5 mL or 25mcg/0.25 mL dose
or	medication	NLM:CVX:218	COVID-19, mRNA, LNP-S, PF, 10 mcg/0.2 mL dose, tris-sucrose
or	medication	NLM:CVX:212	COVID-19 vaccine, vector-nr, rS-Ad26, PF, 0.5 mL
or	medication	NLM:CVX:519	COVID-19 mRNA, bivalent, original/Omicron BA.1, Non-US Vaccine (Spikevax Bivalent), Moderna
or	medication	NLM:CVX:301	COVID-19, mRNA, LNP-S, bivalent, PF, 10 mcg/0.2 mL dose
or	medication	NLM:CVX:228	COVID-19, mRNA, LNP-S, PF, pediatric 25 mcg/0.25 mL dose
or	medication	NLM:CVX:219	COVID-19, mRNA, LNP-S, PF, 3 mcg/0.2 mL dose, tris-sucrose
or	medication	NLM:CVX:520	COVID-19 mRNA, bivalent, original/Omicron BA.1, Non-US Vaccine Product, Pfizer-BioNTech
or	medication	NLM:CVX:221	COVID-19, mRNA, LNP-S, PF, 50 mcg/0.5 mL dose
or	medication	NLM:CVX:210	COVID-19 vaccine, vector-nr, rS-ChAdOx1, PF, 0.5 mL
or	medication	NLM:CVX:511	COVID-19 IV Non-US Vaccine (CoronaVac, Sinovac)
or	medication	NLM:CVX:230	COVID-19, mRNA, LNP-S, bivalent booster, PF, 10 mcg/0.2 mL
or	procedure	UMLS:CPT:0001A	Immunization administration by intramuscular injection of severe acute respiratory syndrome coronavirus 2 (SARS-CoV-2) (coronavirus disease [COVID-19]) vaccine, mRNA-LNP, spike protein, preservative free, 30 mcg/0.3 mL dosage, diluent reconstituted; first dose

or	procedure	UMLS:CPT:0002A	Immunization administration by intramuscular injection of severe acute respiratory syndrome coronavirus 2 (SARS-CoV-2) (coronavirus disease [COVID-19]) vaccine, mRNA-LNP, spike protein, preservative free, 30 mcg/0.3 mL dosage, diluent reconstituted; second dose
or	procedure	UMLS:CPT:91300	Severe acute respiratory syndrome coronavirus 2 (SARS-CoV-2) (coronavirus disease [COVID-19]) vaccine, mRNA-LNP, spike protein, preservative free, 30 mcg/0.3 mL dosage, diluent reconstituted, for intramuscular use
or	medication	NLM:RXNORM:2468231	SARS-CoV-2 (COVID-19) vaccine, mRNA spike protein
or	procedure	UMLS:CPT:0011A	Immunization administration by intramuscular injection of severe acute respiratory syndrome coronavirus 2 (SARS-CoV-2) (coronavirus disease [COVID-19]) vaccine, mRNA-LNP, spike protein, preservative free, 100 mcg/0.5 mL dosage; first dose
or	procedure	UMLS:CPT:0012A	Immunization administration by intramuscular injection of severe acute respiratory syndrome coronavirus 2 (SARS-CoV-2) (coronavirus disease [COVID-19]) vaccine, mRNA-LNP, spike protein, preservative free, 100 mcg/0.5 mL dosage; second dose
or	procedure	UMLS:SNOMED:840534001	Administration of SARS-CoV-2 antigen vaccine
or	medication	NLM:CVX:213	SARS-CoV-2 (COVID-19) Vaccine

or	procedure	UMLS:CPT:1036660	Immunization administration by intramuscular injection of severe acute respiratory syndrome coronavirus 2 (SARS-CoV-2) (coronavirus disease [COVID-19]) vaccine, mRNA-LNP, spike protein, preservative free, 30 mcg/0.3 mL dosage, diluent reconstituted
or	procedure	UMLS:CPT:91301	Severe acute respiratory syndrome coronavirus 2 (SARS-CoV-2) (coronavirus disease [COVID-19]) vaccine, mRNA-LNP, spike protein, preservative free, 100 mcg/0.5 mL dosage, for intramuscular use
or	medication	NLM:CVX:302	COVID-19, mRNA, LNP-S, bivalent, PF, 3 mcg/0.2 mL dose
or	procedure	UMLS:CPT:0124A	Immunization administration by intramuscular injection of severe acute respiratory syndrome coronavirus 2 (SARS-CoV-2) (coronavirus disease [COVID-19]) vaccine, mRNA-LNP, bivalent spike protein, preservative free, 30 mcg/0.3 mL dosage, tris-sucrose formulation, booster dose
or	procedure	UMLS:CPT:0004A	Immunization administration by intramuscular injection of severe acute respiratory syndrome coronavirus 2 (SARS-CoV-2) (coronavirus disease [COVID-19]) vaccine, mRNA-LNP, spike protein, preservative free, 30 mcg/0.3 mL dosage, diluent reconstituted; booster dose
or	procedure	UMLS:CPT:0003A	Immunization administration by intramuscular injection of severe acute respiratory syndrome coronavirus 2 (SARS-CoV-2)

			(coronavirus disease [COVID-19]) vaccine, mRNA-LNP, spike protein, preservative free, 30 mcg/0.3 mL dosage, diluent reconstituted; third dose
or	procedure	UMLS:CPT:1036663	Immunization administration by intramuscular injection of severe acute respiratory syndrome coronavirus 2 (SARS-CoV-2) (coronavirus disease [COVID-19]) vaccine, mRNA-LNP, spike protein, preservative free, 100 mcg/0.5 mL dosage
or	procedure	UMLS:CPT:1037166	Immunization administration by intramuscular injection of severe acute respiratory syndrome coronavirus 2 (SARS-CoV-2) (coronavirus disease [COVID-19]) vaccine, mRNA-LNP, spike protein, preservative free, 30 mcg/0.3 mL dosage, tris-sucrose formulation
or	procedure	UMLS:CPT:0054A	Immunization administration by intramuscular injection of severe acute respiratory syndrome coronavirus 2 (SARS-CoV-2) (coronavirus disease [COVID-19]) vaccine, mRNA-LNP, spike protein, preservative free, 30 mcg/0.3 mL dosage, tris-sucrose formulation; booster dose
or	procedure	UMLS:CPT:90480	Immunization administration by intramuscular injection of severe acute respiratory syndrome coronavirus 2 (SARS-CoV-2) (coronavirus disease [COVID-19]) vaccine, single dose
or	procedure	UMLS:CPT:1037171	Immunization administration by intramuscular injection of severe

			acute respiratory syndrome coronavirus 2 (SARS-CoV-2) (coronavirus disease [COVID-19]) vaccine, mRNA-LNP, spike protein, preservative free, 10 mcg/0.2 mL dosage, diluent reconstituted, tris-sucrose formulation
or	procedure	UMLS:CPT:0071A	Immunization administration by intramuscular injection of severe acute respiratory syndrome coronavirus 2 (SARS-CoV-2) (coronavirus disease [COVID-19]) vaccine, mRNA-LNP, spike protein, preservative free, 10 mcg/0.2 mL dosage, diluent reconstituted, tris-sucrose formulation; first dose
or	procedure	UMLS:CPT:0072A	Immunization administration by intramuscular injection of severe acute respiratory syndrome coronavirus 2 (SARS-CoV-2) (coronavirus disease [COVID-19]) vaccine, mRNA-LNP, spike protein, preservative free, 10 mcg/0.2 mL dosage, diluent reconstituted, tris-sucrose formulation; second dose
or	procedure	UMLS:CPT:91313	Severe acute respiratory syndrome coronavirus 2 (SARS-CoV-2) (coronavirus disease [COVID-19]) vaccine, mRNA-LNP, spike protein, bivalent, preservative free, 50 mcg/0.5 mL dosage, for intramuscular use
or	medication	NLM:RXNORM:2610319	SARS-CoV-2 (COVID-19) vaccine, mRNA-BNT162b2 0.05 MG/ML / SARS-CoV-2 (COVID-19) vaccine, mRNA-BNT162b2 OMICRON (BA.4/BA.5) 0.05 MG/ML Injectable Suspension

or	procedure	UMLS:CPT:0064A	Immunization administration by intramuscular injection of severe acute respiratory syndrome coronavirus 2 (SARS-CoV-2) (coronavirus disease [COVID-19]) vaccine, mRNA-LNP, spike protein, preservative free, 50 mcg/0.25 mL dosage, booster dose
or	procedure	UMLS:CPT:1037175	Immunization administration by intramuscular injection of severe acute respiratory syndrome coronavirus 2 (SARS-CoV-2) (coronavirus disease [COVID-19]) vaccine, DNA, spike protein, adenovirus type 26 (Ad26) vector, preservative free, 5x1010 viral particles/0.5 mL dosage
or	medication	NLM:RXNORM:2610347	0.3 ML SARS-CoV-2 (COVID-19) vaccine, mRNA-BNT162b2 0.05 MG/ML / SARS-CoV-2 (COVID-19) vaccine, mRNA-BNT162b2 OMICRON (BA.4/BA.5) -1 MG/ML Injection
or	procedure	UMLS:CPT:0134A	Immunization administration by intramuscular injection of severe acute respiratory syndrome coronavirus 2 (SARS-CoV-2) (coronavirus disease [COVID-19]) vaccine, mRNA-LNP, spike protein, bivalent, preservative free, 50 mcg/0.5 mL dosage, booster dose
or	procedure	UMLS:CPT:0013A	Immunization administration by intramuscular injection of severe acute respiratory syndrome coronavirus 2 (SARS-CoV-2) (coronavirus disease [COVID-19]) vaccine, mRNA-LNP, spike protein, preservative free, 100 mcg/0.5 mL dosage; third dose

or	procedure	UMLS:CPT:0081A	Immunization administration by intramuscular injection of severe acute respiratory syndrome coronavirus 2 (SARS-CoV-2) (coronavirus disease [COVID-19]) vaccine, mRNA-LNP, spike protein, preservative free, 3 mcg/0.2 mL dosage, diluent reconstituted, tris-sucrose formulation; first dose
or	procedure	UMLS:CPT:1037228	Immunization administration by intramuscular injection of severe acute respiratory syndrome coronavirus 2 (SARS-CoV-2) (coronavirus disease [COVID-19]) vaccine, mRNA-LNP, spike protein, preservative free, 3 mcg/0.2 mL dosage, diluent reconstituted, tris-sucrose formulation
or	procedure	UMLS:CPT:0082A	Immunization administration by intramuscular injection of severe acute respiratory syndrome coronavirus 2 (SARS-CoV-2) (coronavirus disease [COVID-19]) vaccine, mRNA-LNP, spike protein, preservative free, 3 mcg/0.2 mL dosage, diluent reconstituted, tris-sucrose formulation; second dose
or	procedure	UMLS:CPT:0154A	Immunization administration by intramuscular injection of severe acute respiratory syndrome coronavirus 2 (SARS-CoV-2) (coronavirus disease [COVID-19]) vaccine, mRNA-LNP, bivalent spike protein, preservative free, 10 mcg/0.2 mL dosage, diluent reconstituted, tris-sucrose formulation, booster dose

or	procedure	UMLS:CPT:0053A	Immunization administration by intramuscular injection of severe acute respiratory syndrome coronavirus 2 (SARS-CoV-2) (coronavirus disease [COVID-19]) vaccine, mRNA-LNP, spike protein, preservative free, 30 mcg/0.3 mL dosage, tris-sucrose formulation; third dose
or	medication	NLM:RXNORM:2610328	SARS-CoV-2 (COVID-19) vaccine, mRNA-1273 0.05 MG/ML / SARS-CoV-2 (COVID-19) vaccine, mRNA-1273 OMICRON (BA.4/BA.5) 0.05 MG/ML Injectable Suspension
or	procedure	UMLS:CPT:0111A	Immunization administration by intramuscular injection of severe acute respiratory syndrome coronavirus 2 (SARS-CoV-2) (coronavirus disease [COVID-19]) vaccine, mRNA-LNP, spike protein, preservative free, 25 mcg/0.25 mL dosage; first dose
or	procedure	UMLS:CPT:0052A	Immunization administration by intramuscular injection of severe acute respiratory syndrome coronavirus 2 (SARS-CoV-2) (coronavirus disease [COVID-19]) vaccine, mRNA-LNP, spike protein, preservative free, 30 mcg/0.3 mL dosage, tris-sucrose formulation; second dose
or	procedure	UMLS:CPT:0051A	Immunization administration by intramuscular injection of severe acute respiratory syndrome coronavirus 2 (SARS-CoV-2) (coronavirus disease [COVID-19]) vaccine, mRNA-LNP, spike protein, preservative free, 30 mcg/0.3 mL

			dosage, tris-sucrose formulation; first dose
or	procedure	UMLS:CPT:0074A	Immunization administration by intramuscular injection of severe acute respiratory syndrome coronavirus 2 (SARS-CoV-2) (coronavirus disease [COVID-19]) vaccine, mRNA-LNP, spike protein, preservative free, 10 mcg/0.2 mL dosage, diluent reconstituted, tris-sucrose formulation; booster dose
or	procedure	UMLS:CPT:1037332	Immunization administration by intramuscular injection of severe acute respiratory syndrome coronavirus 2 (SARS-CoV-2) (coronavirus disease [COVID-19]) vaccine, mRNA-LNP, spike protein, preservative free, 25 mcg/0.25 mL dosage
or	procedure	UMLS:CPT:0083A	Immunization administration by intramuscular injection of severe acute respiratory syndrome coronavirus 2 (SARS-CoV-2) (coronavirus disease [COVID-19]) vaccine, mRNA-LNP, spike protein, preservative free, 3 mcg/0.2 mL dosage, diluent reconstituted, tris-sucrose formulation; third dose
or	procedure	UMLS:CPT:0112A	Immunization administration by intramuscular injection of severe acute respiratory syndrome coronavirus 2 (SARS-CoV-2) (coronavirus disease [COVID-19]) vaccine, mRNA-LNP, spike protein, preservative free, 25 mcg/0.25 mL dosage; second dose
or	procedure	UMLS:CPT:0073A	Immunization administration by intramuscular injection of severe

			acute respiratory syndrome coronavirus 2 (SARS-CoV-2) (coronavirus disease [COVID-19]) vaccine, mRNA-LNP, spike protein, preservative free, 10 mcg/0.2 mL dosage, diluent reconstituted, tris-sucrose formulation; third dose
or	procedure	UMLS:CPT:0173A	Immunization administration by intramuscular injection of severe acute respiratory syndrome coronavirus 2 (SARS-CoV-2) (coronavirus disease [COVID-19]) vaccine, mRNA-LNP, bivalent spike protein, preservative free, 3 mcg/0.2 mL dosage, diluent reconstituted, tris-sucrose formulation, third dose
or	procedure	UMLS:CPT:0164A	Immunization administration by intramuscular injection of severe acute respiratory syndrome coronavirus 2 (SARS-CoV-2) (coronavirus disease [COVID-19]) vaccine, mRNA-LNP, spike protein, bivalent, preservative free, 10 mcg/0.2 mL dosage, booster dose
or	procedure	UMLS:CPT:1037838	Immunization administration by intramuscular injection of severe acute respiratory syndrome coronavirus 2 (SARS-CoV-2) (coronavirus disease [COVID-19]) vaccine, mRNA-LNP, spike protein, preservative free, 50 mcg/0.5 mL dosage
or	procedure	UMLS:CPT:0094A	Immunization administration by intramuscular injection of severe acute respiratory syndrome coronavirus 2 (SARS-CoV-2) (coronavirus disease [COVID-19]) vaccine, mRNA-LNP, spike protein,

			preservative free, 50 mcg/0.5 mL dosage; booster dose, when administered to individuals 18 years and over
or	procedure	UMLS:CPT:0034A	Immunization administration by intramuscular injection of severe acute respiratory syndrome coronavirus 2 (SARS-CoV-2) (coronavirus disease [COVID-19]) vaccine, DNA, spike protein, adenovirus type 26 (Ad26) vector, preservative free, 5x1010 viral particles/0.5 mL dosage; booster dose
or	procedure	UMLS:CPT:0144A	Immunization administration by intramuscular injection of severe acute respiratory syndrome coronavirus 2 (SARS-CoV-2) (coronavirus disease [COVID-19]) vaccine, mRNA-LNP, spike protein, bivalent, preservative free, 25 mcg/0.25 mL dosage, booster dose
or	procedure	UMLS:CPT:0091A	Immunization administration by intramuscular injection of severe acute respiratory syndrome coronavirus 2 (SARS-CoV-2) (coronavirus disease [COVID-19]) vaccine, mRNA-LNP, spike protein, preservative free, 50 mcg/0.5 mL dosage; first dose, when administered to individuals 6 through 11 years
or	procedure	UMLS:CPT:0174A	Immunization administration by intramuscular injection of severe acute respiratory syndrome coronavirus 2 (SARS-CoV-2) (coronavirus disease [COVID-19]) vaccine, mRNA-LNP, bivalent spike

			protein, preservative free, 3 mcg/0.2 mL dosage, diluent reconstituted, tris-sucrose formulation, booster
or	procedure	UMLS:CPT:0092A	Immunization administration by intramuscular injection of severe acute respiratory syndrome coronavirus 2 (SARS-CoV-2) (coronavirus disease [COVID-19]) vaccine, mRNA-LNP, spike protein, preservative free, 50 mcg/0.5 mL dosage; second dose, when administered to individuals 6 through 11 years
or	procedure	UMLS:CPT:1036682	Immunization administration by intramuscular injection of severe acute respiratory syndrome coronavirus 2 (SARS-CoV-2) (coronavirus disease [COVID-19]) vaccine, recombinant spike protein nanoparticle, saponin-based adjuvant, preservative free, 5 mcg/0.5 mL dosage
or	procedure	UMLS:CPT:0041A	Immunization administration by intramuscular injection of severe acute respiratory syndrome coronavirus 2 (SARS-CoV-2) (coronavirus disease [COVID-19]) vaccine, recombinant spike protein nanoparticle, saponin-based adjuvant, preservative free, 5 mcg/0.5 mL dosage; first dose
or	procedure	UMLS:CPT:91311	Severe acute respiratory syndrome coronavirus 2 (SARS-CoV-2) (coronavirus disease [COVID-19]) vaccine, mRNA-LNP, spike protein, preservative free, 25 mcg/0.25 mL dosage, for intramuscular use

or	procedure	UMLS:CPT:0113A	Immunization administration by intramuscular injection of severe acute respiratory syndrome coronavirus 2 (SARS-CoV-2) (coronavirus disease [COVID-19]) vaccine, mRNA-LNP, spike protein, preservative free, 25 mcg/0.25 mL dosage; third dose
or	procedure	UMLS:CPT:0093A	Immunization administration by intramuscular injection of severe acute respiratory syndrome coronavirus 2 (SARS-CoV-2) (coronavirus disease [COVID-19]) vaccine, mRNA-LNP, spike protein, preservative free, 50 mcg/0.5 mL dosage; third dose, when administered to individuals 6 through 11 years
or	procedure	UMLS:CPT:1036666	Immunization administration by intramuscular injection of severe acute respiratory syndrome coronavirus 2 (SARS-CoV-2) (coronavirus disease [COVID-19]) vaccine, DNA, spike protein, chimpanzee adenovirus Oxford 1 (ChAdOx1) vector, preservative free, 5x1010 viral particles/0.5 mL dosage
or	procedure	UMLS:CPT:0044A	Immunization administration by intramuscular injection of severe acute respiratory syndrome coronavirus 2 (SARS-CoV-2) (coronavirus disease [COVID-19]) vaccine, recombinant spike protein nanoparticle, saponin-based adjuvant, preservative free, 5 mcg/0.5mL dosage; booster
or	procedure	UMLS:CPT:0042A	Immunization administration by intramuscular injection of severe

			acute respiratory syndrome coronavirus 2 (SARS-CoV-2) (coronavirus disease [COVID-19]) vaccine, recombinant spike protein nanoparticle, saponin-based adjuvant, preservative free, 5 mcg/0.5 mL dosage; second dose
or	diagnosis	UMLS:ICD10CM:C73	Malignant neoplasm of thyroid gland
or	diagnosis	UMLS:ICD10CM:Z85.850	Personal history of malignant neoplasm of thyroid
