# Supplementary material for: Association Between COVID-19 Infection and Thyroid Cancer Development: A Retrospective Cohort Study Using the TriNetX Database
Source: Biomedicines. 2025 Aug 8;13(8):1933. doi: 10.3390/biomedicines13081933 (PMC12383963; doi:10.3390/biomedicines13081933)
Supplement: Supplementary file 1 [file biomedicines-13-01933-s001.zip › Supplementary File S2.pdf]

Supplementary File S2: Detailed query criteria of subgroups of population in post-COVID and non-COVID groups.

Query Criteria for Cohort post-COVID-19, male

This query was run on the network Global Collaborative Network with 148 HCO(s) queried and 148 HCO(s) responded. A total of 123 provider(s) responded with patients. The final cohort included 1,005,276 patients who matched the query criteria listed in the table below.

**Ungrouped terms**				
must have		demographics	Age	Age (at least 18 years (most recent occurrence))
	and	demographics	UMLS:HL7V3.0:Gender:M	Male
**Group 1**				
**COVID-19, no vaccination**				
must have	any of	diagnosis	UMLS:ICD10CM:U07.1	COVID-19
		diagnosis	UMLS:ICD10CM:U07.2	COVID-19, virus not identified (WHO)
		diagnosis	UMLS:ICD10CM:J12.82	Pneumonia due to coronavirus disease 2019
		laboratory	UMLS:LNC:94500-6	SARS-CoV-2 (COVID-19) RNA [Presence] in Respiratory specimen by NAA with probe detection (labResult: Positive)
		laboratory	UMLS:LNC:94309-2	SARS-CoV-2 (COVID-19) RNA [Presence] in Specimen by NAA with probe detection (labResult: Positive)
		laboratory	UMLS:LNC:94565-9	SARS-CoV-2 (COVID-19) RNA [Presence] in Nasopharynx by NAA with non-probe detection (labResult: Positive)
		laboratory	UMLS:LNC:94759-8	SARS-CoV-2 (COVID-19) RNA [Presence] in Nasopharynx by NAA with probe detection (labResult: Positive)
		laboratory	UMLS:LNC:95608-6	SARS-CoV-2 (COVID-19) RNA [Presence] in Respiratory

				specimen by NAA with non-probe detection (labResult: Positive)
		laboratory	UMLS:LNC:94845-5	SARS-CoV-2 (COVID-19) RNA [Presence] in Saliva (oral fluid) by NAA with probe detection (labResult: Positive)
		laboratory	UMLS:LNC:95406-5	SARS-CoV-2 (COVID-19) RNA [Presence] in Nose by NAA with probe detection (labResult: Positive)
cannot have		medication	NLM:CVX:208	COVID-19, mRNA, LNP-S, PF, 30 mcg/0.3 mL dose
	or	medication	NLM:CVX:207	COVID-19, mRNA, LNP-S, PF, 100 mcg/0.5mL dose or 50 mcg/0.25mL dose
	or	medication	NLM:CVX:212	COVID-19 vaccine, vector-nr, rS-Ad26, PF, 0.5 mL
	or	medication	NLM:RXNORM:OMOP 5042939	COVID-19 vaccine
	or	medication	NLM:CVX:300	COVID-19, mRNA, LNP-S, bivalent, PF, 30 mcg/0.3 mL dose
	or	medication	NLM:CVX:217	COVID-19, mRNA, LNP-S, PF, 30 mcg/0.3 mL dose, tris-sucrose
	or	medication	NLM:CVX:229	COVID-19, mRNA, LNP-S, bivalent, PF, 50 mcg/0.5 mL or 25mcg/0.25 mL dose
	or	medication	NLM:CVX:218	COVID-19, mRNA, LNP-S, PF, 10 mcg/0.2 mL dose, tris-sucrose
	or	medication	NLM:CVX:520	COVID-19 mRNA, bivalent, original/Omicron BA.1, Non-US Vaccine Product, Pfizer-BioNTech
	or	medication	NLM:CVX:519	COVID-19 mRNA, bivalent, original/Omicron BA.1, Non-US Vaccine (Spikevax Bivalent), Moderna

or	medication	NLM:CVX:301	COVID-19, mRNA, LNP-S, bivalent, PF, 10 mcg/0.2 mL dose
or	medication	NLM:CVX:219	COVID-19, mRNA, LNP-S, PF, 3 mcg/0.2 mL dose, tris-sucrose
or	medication	NLM:CVX:228	COVID-19, mRNA, LNP-S, PF, pediatric 25 mcg/0.25 mL dose
or	medication	NLM:CVX:230	COVID-19, mRNA, LNP-S, bivalent booster, PF, 10 mcg/0.2 mL
or	medication	NLM:CVX:221	COVID-19, mRNA, LNP-S, PF, 50 mcg/0.5 mL dose
or	medication	NLM:CVX:210	COVID-19 vaccine, vector-nr, rS-ChAdOx1, PF, 0.5 mL
or	medication	NLM:CVX:302	COVID-19, mRNA, LNP-S, bivalent, PF, 3 mcg/0.2 mL dose
or	medication	NLM:CVX:511	COVID-19 IV Non-US Vaccine (CoronaVac, Sinovac)
or	medication	NLM:RXNORM:2468231	SARS-CoV-2 (COVID-19) vaccine, mRNA spike protein
or	procedure	UMLS:CPT:91300	Severe acute respiratory syndrome coronavirus 2 (SARS-CoV-2) (coronavirus disease [COVID-19]) vaccine, mRNA-LNP, spike protein, preservative free, 30 mcg/0.3 mL dosage, diluent reconstituted, for intramuscular use
or	procedure	UMLS:CPT:0001A	Immunization administration by intramuscular injection of severe acute respiratory syndrome coronavirus 2 (SARS-CoV-2) (coronavirus disease [COVID-19]) vaccine, mRNA-LNP, spike protein, preservative free, 30 mcg/0.3 mL dosage, diluent reconstituted; first dose

or	procedure	UMLS:CPT:0002A	Immunization administration by intramuscular injection of severe acute respiratory syndrome coronavirus 2 (SARS-CoV-2) (coronavirus disease [COVID-19]) vaccine, mRNA-LNP, spike protein, preservative free, 30 mcg/0.3 mL dosage, diluent reconstituted; second dose
or	procedure	UMLS:CPT:91301	Severe acute respiratory syndrome coronavirus 2 (SARS-CoV-2) (coronavirus disease [COVID-19]) vaccine, mRNA-LNP, spike protein, preservative free, 100 mcg/0.5 mL dosage, for intramuscular use
or	procedure	UMLS:CPT:0011A	Immunization administration by intramuscular injection of severe acute respiratory syndrome coronavirus 2 (SARS-CoV-2) (coronavirus disease [COVID-19]) vaccine, mRNA-LNP, spike protein, preservative free, 100 mcg/0.5 mL dosage; first dose
or	procedure	UMLS:CPT:0012A	Immunization administration by intramuscular injection of severe acute respiratory syndrome coronavirus 2 (SARS-CoV-2) (coronavirus disease [COVID-19]) vaccine, mRNA-LNP, spike protein, preservative free, 100 mcg/0.5 mL dosage; second dose
or	procedure	UMLS:SNOMED:840534001	Administration of SARS-CoV-2 antigen vaccine

or	medication	NLM:CVX:213	SARS-CoV-2 (COVID-19) Vaccine
or	procedure	UMLS:CPT:1036660	Immunization administration by intramuscular injection of severe acute respiratory syndrome coronavirus 2 (SARS-CoV-2) (coronavirus disease [COVID-19]) vaccine, mRNA-LNP, spike protein, preservative free, 30 mcg/0.3 mL dosage, diluent reconstituted
or	procedure	UMLS:CPT:1036663	Immunization administration by intramuscular injection of severe acute respiratory syndrome coronavirus 2 (SARS-CoV-2) (coronavirus disease [COVID-19]) vaccine, mRNA-LNP, spike protein, preservative free, 100 mcg/0.5 mL dosage
or	procedure	UMLS:CPT:0124A	Immunization administration by intramuscular injection of severe acute respiratory syndrome coronavirus 2 (SARS-CoV-2) (coronavirus disease [COVID-19]) vaccine, mRNA-LNP, bivalent spike protein, preservative free, 30 mcg/0.3 mL dosage, tris-sucrose formulation, booster dose
or	procedure	UMLS:CPT:0004A	Immunization administration by intramuscular injection of severe acute respiratory syndrome coronavirus 2 (SARS-CoV-2) (coronavirus disease [COVID-19]) vaccine, mRNA-LNP, spike protein, preservative free, 30 mcg/0.3 mL dosage,

			diluent reconstituted; booster dose
or	procedure	UMLS:CPT:0003A	Immunization administration by intramuscular injection of severe acute respiratory syndrome coronavirus 2 (SARS-CoV-2) (coronavirus disease [COVID-19]) vaccine, mRNA-LNP, spike protein, preservative free, 30 mcg/0.3 mL dosage, diluent reconstituted; third dose
or	procedure	UMLS:CPT:1037166	Immunization administration by intramuscular injection of severe acute respiratory syndrome coronavirus 2 (SARS-CoV-2) (coronavirus disease [COVID-19]) vaccine, mRNA-LNP, spike protein, preservative free, 30 mcg/0.3 mL dosage, tris-sucrose formulation
or	procedure	UMLS:CPT:0054A	Immunization administration by intramuscular injection of severe acute respiratory syndrome coronavirus 2 (SARS-CoV-2) (coronavirus disease [COVID-19]) vaccine, mRNA-LNP, spike protein, preservative free, 30 mcg/0.3 mL dosage, tris-sucrose formulation; booster dose
or	procedure	UMLS:CPT:0064A	Immunization administration by intramuscular injection of severe acute respiratory syndrome coronavirus 2 (SARS-CoV-2) (coronavirus disease [COVID-19]) vaccine, mRNA-LNP, spike protein, preservative

			free, 50 mcg/0.25 mL dosage, booster dose
or	procedure	UMLS:CPT:90480	Immunization administration by intramuscular injection of severe acute respiratory syndrome coronavirus 2 (SARS-CoV-2) (coronavirus disease [COVID-19]) vaccine, single dose
or	procedure	UMLS:CPT:1037171	Immunization administration by intramuscular injection of severe acute respiratory syndrome coronavirus 2 (SARS-CoV-2) (coronavirus disease [COVID-19]) vaccine, mRNA-LNP, spike protein, preservative free, 10 mcg/0.2 mL dosage, diluent reconstituted, tris-sucrose formulation
or	procedure	UMLS:CPT:0071A	Immunization administration by intramuscular injection of severe acute respiratory syndrome coronavirus 2 (SARS-CoV-2) (coronavirus disease [COVID-19]) vaccine, mRNA-LNP, spike protein, preservative free, 10 mcg/0.2 mL dosage, diluent reconstituted, tris-sucrose formulation; first dose
or	procedure	UMLS:CPT:0072A	Immunization administration by intramuscular injection of severe acute respiratory syndrome coronavirus 2 (SARS-CoV-2) (coronavirus disease [COVID-19]) vaccine, mRNA-LNP, spike protein, preservative free, 10 mcg/0.2 mL dosage, diluent reconstituted, tris-

			sucrose formulation; second dose
or	medication	NLM:RXNORM:2610319	SARS-CoV-2 (COVID-19) vaccine, mRNA-BNT162b2 0.05 MG/ML / SARS-CoV-2 (COVID-19) vaccine, mRNA-BNT162b2 OMICRON (BA.4/BA.5) 0.05 MG/ML Injectable Suspension
or	procedure	UMLS:CPT:91313	Severe acute respiratory syndrome coronavirus 2 (SARS-CoV-2) (coronavirus disease [COVID-19]) vaccine, mRNA-LNP, spike protein, bivalent, preservative free, 50 mcg/0.5 mL dosage, for intramuscular use
or	procedure	UMLS:CPT:0134A	Immunization administration by intramuscular injection of severe acute respiratory syndrome coronavirus 2 (SARS-CoV-2) (coronavirus disease [COVID-19]) vaccine, mRNA-LNP, spike protein, bivalent, preservative free, 50 mcg/0.5 mL dosage, booster dose
or	procedure	UMLS:CPT:1037175	Immunization administration by intramuscular injection of severe acute respiratory syndrome coronavirus 2 (SARS-CoV-2) (coronavirus disease [COVID-19]) vaccine, DNA, spike protein, adenovirus type 26 (Ad26) vector, preservative free, 5x1010 viral particles/0.5 mL dosage
or	medication	NLM:RXNORM:2610347	0.3 ML SARS-CoV-2 (COVID-19) vaccine, mRNA-BNT162b2 0.05 MG/ML / SARS-CoV-2 (COVID-19) vaccine, mRNA-BNT162b2

			OMICRON (BA.4/BA.5) -1 MG/ML Injection
or	procedure	UMLS:CPT:1037228	Immunization administration by intramuscular injection of severe acute respiratory syndrome coronavirus 2 (SARS-CoV-2) (coronavirus disease [COVID-19]) vaccine, mRNA-LNP, spike protein, preservative free, 3 mcg/0.2 mL dosage, diluent reconstituted, tris-sucrose formulation
or	procedure	UMLS:CPT:0013A	Immunization administration by intramuscular injection of severe acute respiratory syndrome coronavirus 2 (SARS-CoV-2) (coronavirus disease [COVID-19]) vaccine, mRNA-LNP, spike protein, preservative free, 100 mcg/0.5 mL dosage; third dose
or	procedure	UMLS:CPT:0081A	Immunization administration by intramuscular injection of severe acute respiratory syndrome coronavirus 2 (SARS-CoV-2) (coronavirus disease [COVID-19]) vaccine, mRNA-LNP, spike protein, preservative free, 3 mcg/0.2 mL dosage, diluent reconstituted, tris-sucrose formulation; first dose
or	procedure	UMLS:CPT:0082A	Immunization administration by intramuscular injection of severe acute respiratory syndrome coronavirus 2 (SARS-CoV-2) (coronavirus disease [COVID-19]) vaccine, mRNA-LNP, spike protein, preservative

			free, 3 mcg/0.2 mL dosage, diluent reconstituted, tris-sucrose formulation; second dose
or	medication	NLM:RXNORM:2610328	SARS-CoV-2 (COVID-19) vaccine, mRNA-1273 0.05 MG/ML / SARS-CoV-2 (COVID-19) vaccine, mRNA-1273 OMICRON (BA.4/BA.5) 0.05 MG/ML Injectable Suspension
or	procedure	UMLS:CPT:0154A	Immunization administration by intramuscular injection of severe acute respiratory syndrome coronavirus 2 (SARS-CoV-2) (coronavirus disease [COVID-19]) vaccine, mRNA-LNP, bivalent spike protein, preservative free, 10 mcg/0.2 mL dosage, diluent reconstituted, tris-sucrose formulation, booster dose
or	procedure	UMLS:CPT:0053A	Immunization administration by intramuscular injection of severe acute respiratory syndrome coronavirus 2 (SARS-CoV-2) (coronavirus disease [COVID-19]) vaccine, mRNA-LNP, spike protein, preservative free, 30 mcg/0.3 mL dosage, tris-sucrose formulation; third dose
or	procedure	UMLS:CPT:1037332	Immunization administration by intramuscular injection of severe acute respiratory syndrome coronavirus 2 (SARS-CoV-2) (coronavirus disease [COVID-19]) vaccine, mRNA-

			LNP, spike protein, preservative free, 25 mcg/0.25 mL dosage
or	procedure	UMLS:CPT:0052A	Immunization administration by intramuscular injection of severe acute respiratory syndrome coronavirus 2 (SARS-CoV-2) (coronavirus disease [COVID-19]) vaccine, mRNA-LNP, spike protein, preservative free, 30 mcg/0.3 mL dosage, tris-sucrose formulation; second dose
or	procedure	UMLS:CPT:0111A	Immunization administration by intramuscular injection of severe acute respiratory syndrome coronavirus 2 (SARS-CoV-2) (coronavirus disease [COVID-19]) vaccine, mRNA-LNP, spike protein, preservative free, 25 mcg/0.25 mL dosage; first dose
or	procedure	UMLS:CPT:0051A	Immunization administration by intramuscular injection of severe acute respiratory syndrome coronavirus 2 (SARS-CoV-2) (coronavirus disease [COVID-19]) vaccine, mRNA-LNP, spike protein, preservative free, 30 mcg/0.3 mL dosage, tris-sucrose formulation; first dose
or	procedure	UMLS:CPT:91311	Severe acute respiratory syndrome coronavirus 2 (SARS-CoV-2) (coronavirus disease [COVID-19]) vaccine, mRNA-LNP, spike protein, preservative free, 25 mcg/0.25 mL dosage, for intramuscular use

or	procedure	UMLS:CPT:0074A	Immunization administration by intramuscular injection of severe acute respiratory syndrome coronavirus 2 (SARS-CoV-2) (coronavirus disease [COVID-19]) vaccine, mRNA-LNP, spike protein, preservative free, 10 mcg/0.2 mL dosage, diluent reconstituted, tris-sucrose formulation; booster dose
or	procedure	UMLS:CPT:0112A	Immunization administration by intramuscular injection of severe acute respiratory syndrome coronavirus 2 (SARS-CoV-2) (coronavirus disease [COVID-19]) vaccine, mRNA-LNP, spike protein, preservative free, 25 mcg/0.25 mL dosage; second dose
or	procedure	UMLS:CPT:0083A	Immunization administration by intramuscular injection of severe acute respiratory syndrome coronavirus 2 (SARS-CoV-2) (coronavirus disease [COVID-19]) vaccine, mRNA-LNP, spike protein, preservative free, 3 mcg/0.2 mL dosage, diluent reconstituted, tris-sucrose formulation; third dose
or	procedure	UMLS:CPT:0073A	Immunization administration by intramuscular injection of severe acute respiratory syndrome coronavirus 2 (SARS-CoV-2) (coronavirus disease [COVID-19]) vaccine, mRNA-LNP, spike protein, preservative free, 10 mcg/0.2 mL dosage,

			diluent reconstituted, tris-sucrose formulation; third dose
or	procedure	UMLS:CPT:0173A	Immunization administration by intramuscular injection of severe acute respiratory syndrome coronavirus 2 (SARS-CoV-2) (coronavirus disease [COVID-19]) vaccine, mRNA-LNP, bivalent spike protein, preservative free, 3 mcg/0.2 mL dosage, diluent reconstituted, tris-sucrose formulation, third dose
or	procedure	UMLS:CPT:0164A	Immunization administration by intramuscular injection of severe acute respiratory syndrome coronavirus 2 (SARS-CoV-2) (coronavirus disease [COVID-19]) vaccine, mRNA-LNP, spike protein, bivalent, preservative free, 10 mcg/0.2 mL dosage, booster dose
or	procedure	UMLS:CPT:1037838	Immunization administration by intramuscular injection of severe acute respiratory syndrome coronavirus 2 (SARS-CoV-2) (coronavirus disease [COVID-19]) vaccine, mRNA-LNP, spike protein, preservative free, 50 mcg/0.5 mL dosage
or	procedure	UMLS:CPT:0094A	Immunization administration by intramuscular injection of severe acute respiratory syndrome coronavirus 2 (SARS-CoV-2) (coronavirus disease [COVID-19]) vaccine, mRNA-LNP, spike protein, preservative free, 50 mcg/0.5 mL dosage;

			booster dose, when administered to individuals 18 years and over
or	procedure	UMLS:CPT:0034A	Immunization administration by intramuscular injection of severe acute respiratory syndrome coronavirus 2 (SARS-CoV-2) (coronavirus disease [COVID-19]) vaccine, DNA, spike protein, adenovirus type 26 (Ad26) vector, preservative free, 5x1010 viral particles/0.5 mL dosage; booster dose
or	procedure	UMLS:CPT:0144A	Immunization administration by intramuscular injection of severe acute respiratory syndrome coronavirus 2 (SARS-CoV-2) (coronavirus disease [COVID-19]) vaccine, mRNA-LNP, spike protein, bivalent, preservative free, 25 mcg/0.25 mL dosage, booster dose
or	procedure	UMLS:CPT:0091A	Immunization administration by intramuscular injection of severe acute respiratory syndrome coronavirus 2 (SARS-CoV-2) (coronavirus disease [COVID-19]) vaccine, mRNA-LNP, spike protein, preservative free, 50 mcg/0.5 mL dosage; first dose, when administered to individuals 6 through 11 years
or	procedure	UMLS:CPT:0174A	Immunization administration by intramuscular injection of severe acute respiratory syndrome coronavirus 2 (SARS-CoV-2) (coronavirus disease [COVID-19]) vaccine, mRNA-

			LNP, bivalent spike protein, preservative free, 3 mcg/0.2 mL dosage, diluent reconstituted, tris-sucrose formulation, booster
or	procedure	UMLS:CPT:0092A	Immunization administration by intramuscular injection of severe acute respiratory syndrome coronavirus 2 (SARS-CoV-2) (coronavirus disease [COVID-19]) vaccine, mRNA-LNP, spike protein, preservative free, 50 mcg/0.5 mL dosage; second dose, when administered to individuals 6 through 11 years
or	procedure	UMLS:CPT:1036682	Immunization administration by intramuscular injection of severe acute respiratory syndrome coronavirus 2 (SARS-CoV-2) (coronavirus disease [COVID-19]) vaccine, recombinant spike protein nanoparticle, saponin-based adjuvant, preservative free, 5 mcg/0.5 mL dosage
or	procedure	UMLS:CPT:0041A	Immunization administration by intramuscular injection of severe acute respiratory syndrome coronavirus 2 (SARS-CoV-2) (coronavirus disease [COVID-19]) vaccine, recombinant spike protein nanoparticle, saponin-based adjuvant, preservative free, 5 mcg/0.5 mL dosage; first dose
or	procedure	UMLS:CPT:0113A	Immunization administration by intramuscular injection of

			severe acute respiratory syndrome coronavirus 2 (SARS-CoV-2) (coronavirus disease [COVID-19]) vaccine, mRNA-LNP, spike protein, preservative free, 25 mcg/0.25 mL dosage; third dose
or	procedure	UMLS:CPT:0042A	Immunization administration by intramuscular injection of severe acute respiratory syndrome coronavirus 2 (SARS-CoV-2) (coronavirus disease [COVID-19]) vaccine, recombinant spike protein nanoparticle, saponin-based adjuvant, preservative free, 5 mcg/0.5 mL dosage; second dose
or	procedure	UMLS:CPT:0093A	Immunization administration by intramuscular injection of severe acute respiratory syndrome coronavirus 2 (SARS-CoV-2) (coronavirus disease [COVID-19]) vaccine, mRNA-LNP, spike protein, preservative free, 50 mcg/0.5 mL dosage; third dose, when administered to individuals 6 through 11 years
or	procedure	UMLS:CPT:1036666	Immunization administration by intramuscular injection of severe acute respiratory syndrome coronavirus 2 (SARS-CoV-2) (coronavirus disease [COVID-19]) vaccine, DNA, spike protein, chimpanzee adenovirus Oxford 1 (ChAdOx1) vector, preservative free, 5x1010 viral particles/0.5 mL dosage

	or	procedure	UMLS:CPT:0044A	Immunization administration by intramuscular injection of severe acute respiratory syndrome coronavirus 2 (SARS-CoV-2) (coronavirus disease [COVID-19]) vaccine, recombinant spike protein nanoparticle, saponin-based adjuvant, preservative free, 5 mcg/0.5mL dosage; booster
date constraint		The terms in this group occurred between Dec 1, 2019 and Dec 31, 2023		
**Group 2**				
**Group 2A COVID-19**				
must have	any of	diagnosis	UMLS:ICD10CM:U07.1	COVID-19
		diagnosis	UMLS:ICD10CM:U07.2	COVID-19, virus not identified (WHO)
		diagnosis	UMLS:ICD10CM:J12.82	Pneumonia due to coronavirus disease 2019
		laboratory	UMLS:LNC:94500-6	SARS-CoV-2 (COVID-19) RNA [Presence] in Respiratory specimen by NAA with probe detection (labResult: Positive)
		laboratory	UMLS:LNC:94309-2	SARS-CoV-2 (COVID-19) RNA [Presence] in Specimen by NAA with probe detection (labResult: Positive)
		laboratory	UMLS:LNC:94565-9	SARS-CoV-2 (COVID-19) RNA [Presence] in Nasopharynx by NAA with non-probe detection (labResult: Positive)
		laboratory	UMLS:LNC:94759-8	SARS-CoV-2 (COVID-19) RNA [Presence] in Nasopharynx by NAA with probe detection (labResult: Positive)
		laboratory	UMLS:LNC:95608-6	SARS-CoV-2 (COVID-19) RNA [Presence] in Respiratory specimen by NAA with non-

				probe detection (labResult: Positive)
		laboratory	UMLS:LNC:94845-5	SARS-CoV-2 (COVID-19) RNA [Presence] in Saliva (oral fluid) by NAA with probe detection (labResult: Positive)
		laboratory	UMLS:LNC:95406-5	SARS-CoV-2 (COVID-19) RNA [Presence] in Nose by NAA with probe detection (labResult: Positive)
	and	visit	TNX:Visit	Visit (Data Source: TriNetX)
date constraint		The terms in this group occurred at any time		
event relationship		Any instance of thyroid cancer history occurred at least 1 day before the first instance of COVID-19		
**Group 2B thyroid cancer history**				
cannot have		diagnosis	UMLS:ICD10CM:C73	Malignant neoplasm of thyroid gland
	or	diagnosis	UMLS:ICD10CM:Z85.850	Personal history of malignant neoplasm of thyroid

Query Criteria for Cohort non-COVID, male

This query was run on the network Global Collaborative Network with 148 HCO(s) queried and 148 HCO(s) responded. A total of 131 provider(s) responded with patients. The final cohort included 13,115,301 patients who matched the query criteria listed in the table below.

**Ungrouped terms**				
must have		demographics	Age	Age (at least 18 years (most recent occurrence))
	and	demographics	UMLS:HL7V3.0:Gender:M	Male
**Group 1**				
**COVID-19 negative, no vaccination**				
must have		visit	TNX:Visit	Visit
cannot have		medication	NLM:CVX:208	COVID-19, mRNA, LNP-S, PF, 30 mcg/0.3 mL dose

or	medication	NLM:CVX:207	COVID-19, mRNA, LNP-S, PF, 100 mcg/0.5mL dose or 50 mcg/0.25mL dose
or	medication	NLM:CVX:212	COVID-19 vaccine, vector-nr, rS-Ad26, PF, 0.5 mL
or	medication	NLM:RXNORM:OMOP 5042939	COVID-19 vaccine
or	medication	NLM:CVX:300	COVID-19, mRNA, LNP-S, bivalent, PF, 30 mcg/0.3 mL dose
or	medication	NLM:CVX:217	COVID-19, mRNA, LNP-S, PF, 30 mcg/0.3 mL dose, tris-sucrose
or	medication	NLM:CVX:229	COVID-19, mRNA, LNP-S, bivalent, PF, 50 mcg/0.5 mL or 25mcg/0.25 mL dose
or	medication	NLM:CVX:218	COVID-19, mRNA, LNP-S, PF, 10 mcg/0.2 mL dose, tris-sucrose
or	medication	NLM:CVX:520	COVID-19 mRNA, bivalent, original/Omicron BA.1, Non-US Vaccine Product, Pfizer-BioNTech
or	medication	NLM:CVX:519	COVID-19 mRNA, bivalent, original/Omicron BA.1, Non-US Vaccine (Spikevax Bivalent), Moderna
or	medication	NLM:CVX:301	COVID-19, mRNA, LNP-S, bivalent, PF, 10 mcg/0.2 mL dose
or	medication	NLM:CVX:219	COVID-19, mRNA, LNP-S, PF, 3 mcg/0.2 mL dose, tris-sucrose
or	medication	NLM:CVX:228	COVID-19, mRNA, LNP-S, PF, pediatric 25 mcg/0.25 mL dose
or	medication	NLM:CVX:230	COVID-19, mRNA, LNP-S, bivalent booster, PF, 10 mcg/0.2 mL
or	medication	NLM:CVX:221	COVID-19, mRNA, LNP-S, PF, 50 mcg/0.5 mL dose

or	medication	NLM:CVX:210	COVID-19 vaccine, vector-nr, rS-ChAdOx1, PF, 0.5 mL
or	medication	NLM:CVX:302	COVID-19, mRNA, LNP-S, bivalent, PF, 3 mcg/0.2 mL dose
or	medication	NLM:CVX:511	COVID-19 IV Non-US Vaccine (CoronaVac, Sinovac)
or	medication	NLM:RXNORM:2468231	SARS-CoV-2 (COVID-19) vaccine, mRNA spike protein
or	procedure	UMLS:CPT:91300	Severe acute respiratory syndrome coronavirus 2 (SARS-CoV-2) (coronavirus disease [COVID-19]) vaccine, mRNA-LNP, spike protein, preservative free, 30 mcg/0.3 mL dosage, diluent reconstituted, for intramuscular use
or	procedure	UMLS:CPT:0001A	Immunization administration by intramuscular injection of severe acute respiratory syndrome coronavirus 2 (SARS-CoV-2) (coronavirus disease [COVID-19]) vaccine, mRNA-LNP, spike protein, preservative free, 30 mcg/0.3 mL dosage, diluent reconstituted; first dose
or	procedure	UMLS:CPT:0002A	Immunization administration by intramuscular injection of severe acute respiratory syndrome coronavirus 2 (SARS-CoV-2) (coronavirus disease [COVID-19]) vaccine, mRNA-LNP, spike protein, preservative free, 30 mcg/0.3 mL dosage, diluent reconstituted; second dose
or	procedure	UMLS:CPT:91301	Severe acute respiratory syndrome coronavirus 2 (SARS-CoV-2) (coronavirus disease

			[COVID-19]) vaccine, mRNA-LNP, spike protein, preservative free, 100 mcg/0.5 mL dosage, for intramuscular use
or	procedure	UMLS:CPT:0011A	Immunization administration by intramuscular injection of severe acute respiratory syndrome coronavirus 2 (SARS-CoV-2) (coronavirus disease [COVID-19]) vaccine, mRNA-LNP, spike protein, preservative free, 100 mcg/0.5 mL dosage; first dose
or	procedure	UMLS:CPT:0012A	Immunization administration by intramuscular injection of severe acute respiratory syndrome coronavirus 2 (SARS-CoV-2) (coronavirus disease [COVID-19]) vaccine, mRNA-LNP, spike protein, preservative free, 100 mcg/0.5 mL dosage; second dose
or	procedure	UMLS:SNOMED:840534001	Administration of SARS-CoV-2 antigen vaccine
or	medication	NLM:CVX:213	SARS-CoV-2 (COVID-19) Vaccine
or	procedure	UMLS:CPT:1036660	Immunization administration by intramuscular injection of severe acute respiratory syndrome coronavirus 2 (SARS-CoV-2) (coronavirus disease [COVID-19]) vaccine, mRNA-LNP, spike protein, preservative free, 30 mcg/0.3 mL dosage, diluent reconstituted
or	procedure	UMLS:CPT:1036663	Immunization administration by intramuscular injection of severe acute respiratory

			syndrome coronavirus 2 (SARS-CoV-2) (coronavirus disease [COVID-19]) vaccine, mRNA-LNP, spike protein, preservative free, 100 mcg/0.5 mL dosage
or	procedure	UMLS:CPT:0124A	Immunization administration by intramuscular injection of severe acute respiratory syndrome coronavirus 2 (SARS-CoV-2) (coronavirus disease [COVID-19]) vaccine, mRNA-LNP, bivalent spike protein, preservative free, 30 mcg/0.3 mL dosage, tris-sucrose formulation, booster dose
or	procedure	UMLS:CPT:0004A	Immunization administration by intramuscular injection of severe acute respiratory syndrome coronavirus 2 (SARS-CoV-2) (coronavirus disease [COVID-19]) vaccine, mRNA-LNP, spike protein, preservative free, 30 mcg/0.3 mL dosage, diluent reconstituted; booster dose
or	procedure	UMLS:CPT:0003A	Immunization administration by intramuscular injection of severe acute respiratory syndrome coronavirus 2 (SARS-CoV-2) (coronavirus disease [COVID-19]) vaccine, mRNA-LNP, spike protein, preservative free, 30 mcg/0.3 mL dosage, diluent reconstituted; third dose
or	procedure	UMLS:CPT:1037166	Immunization administration by intramuscular injection of severe acute respiratory syndrome coronavirus 2 (SARS-

			CoV-2) (coronavirus disease [COVID-19]) vaccine, mRNA-LNP, spike protein, preservative free, 30 mcg/0.3 mL dosage, tris-sucrose formulation
or	procedure	UMLS:CPT:0054A	Immunization administration by intramuscular injection of severe acute respiratory syndrome coronavirus 2 (SARS-CoV-2) (coronavirus disease [COVID-19]) vaccine, mRNA-LNP, spike protein, preservative free, 30 mcg/0.3 mL dosage, tris-sucrose formulation; booster dose
or	procedure	UMLS:CPT:0064A	Immunization administration by intramuscular injection of severe acute respiratory syndrome coronavirus 2 (SARS-CoV-2) (coronavirus disease [COVID-19]) vaccine, mRNA-LNP, spike protein, preservative free, 50 mcg/0.25 mL dosage, booster dose
or	procedure	UMLS:CPT:90480	Immunization administration by intramuscular injection of severe acute respiratory syndrome coronavirus 2 (SARS-CoV-2) (coronavirus disease [COVID-19]) vaccine, single dose
or	procedure	UMLS:CPT:1037171	Immunization administration by intramuscular injection of severe acute respiratory syndrome coronavirus 2 (SARS-CoV-2) (coronavirus disease [COVID-19]) vaccine, mRNA-LNP, spike protein, preservative

			free, 10 mcg/0.2 mL dosage, diluent reconstituted, tris-sucrose formulation
or	procedure	UMLS:CPT:0071A	Immunization administration by intramuscular injection of severe acute respiratory syndrome coronavirus 2 (SARS-CoV-2) (coronavirus disease [COVID-19]) vaccine, mRNA-LNP, spike protein, preservative free, 10 mcg/0.2 mL dosage, diluent reconstituted, tris-sucrose formulation; first dose
or	procedure	UMLS:CPT:0072A	Immunization administration by intramuscular injection of severe acute respiratory syndrome coronavirus 2 (SARS-CoV-2) (coronavirus disease [COVID-19]) vaccine, mRNA-LNP, spike protein, preservative free, 10 mcg/0.2 mL dosage, diluent reconstituted, tris-sucrose formulation; second dose
or	medication	NLM:RXNORM:2610319	SARS-CoV-2 (COVID-19) vaccine, mRNA-BNT162b2 0.05 MG/ML / SARS-CoV-2 (COVID-19) vaccine, mRNA-BNT162b2 OMICRON (BA.4/BA.5) 0.05 MG/ML Injectable Suspension
or	procedure	UMLS:CPT:91313	Severe acute respiratory syndrome coronavirus 2 (SARS-CoV-2) (coronavirus disease [COVID-19]) vaccine, mRNA-LNP, spike protein, bivalent, preservative free, 50 mcg/0.5 mL dosage, for intramuscular use

or	procedure	UMLS:CPT:0134A	Immunization administration by intramuscular injection of severe acute respiratory syndrome coronavirus 2 (SARS-CoV-2) (coronavirus disease [COVID-19]) vaccine, mRNA-LNP, spike protein, bivalent, preservative free, 50 mcg/0.5 mL dosage, booster dose
or	procedure	UMLS:CPT:1037175	Immunization administration by intramuscular injection of severe acute respiratory syndrome coronavirus 2 (SARS-CoV-2) (coronavirus disease [COVID-19]) vaccine, DNA, spike protein, adenovirus type 26 (Ad26) vector, preservative free, 5x1010 viral particles/0.5 mL dosage
or	medication	NLM:RXNORM:2610347	0.3 ML SARS-CoV-2 (COVID-19) vaccine, mRNA-BNT162b2 0.05 MG/ML / SARS-CoV-2 (COVID-19) vaccine, mRNA-BNT162b2 OMICRON (BA.4/BA.5) -1 MG/ML Injection
or	procedure	UMLS:CPT:1037228	Immunization administration by intramuscular injection of severe acute respiratory syndrome coronavirus 2 (SARS-CoV-2) (coronavirus disease [COVID-19]) vaccine, mRNA-LNP, spike protein, preservative free, 3 mcg/0.2 mL dosage, diluent reconstituted, tris-sucrose formulation
or	procedure	UMLS:CPT:0013A	Immunization administration by intramuscular injection of severe acute respiratory

			syndrome coronavirus 2 (SARS-CoV-2) (coronavirus disease [COVID-19]) vaccine, mRNA-LNP, spike protein, preservative free, 100 mcg/0.5 mL dosage; third dose
or	procedure	UMLS:CPT:0081A	Immunization administration by intramuscular injection of severe acute respiratory syndrome coronavirus 2 (SARS-CoV-2) (coronavirus disease [COVID-19]) vaccine, mRNA-LNP, spike protein, preservative free, 3 mcg/0.2 mL dosage, diluent reconstituted, tris-sucrose formulation; first dose
or	procedure	UMLS:CPT:0082A	Immunization administration by intramuscular injection of severe acute respiratory syndrome coronavirus 2 (SARS-CoV-2) (coronavirus disease [COVID-19]) vaccine, mRNA-LNP, spike protein, preservative free, 3 mcg/0.2 mL dosage, diluent reconstituted, tris-sucrose formulation; second dose
or	medication	NLM:RXNORM:2610328	SARS-CoV-2 (COVID-19) vaccine, mRNA-1273 0.05 MG/ML / SARS-CoV-2 (COVID-19) vaccine, mRNA-1273 OMICRON (BA.4/BA.5) 0.05 MG/ML Injectable Suspension
or	procedure	UMLS:CPT:0154A	Immunization administration by intramuscular injection of severe acute respiratory syndrome coronavirus 2 (SARS-CoV-2) (coronavirus disease

			[COVID-19]) vaccine, mRNA-LNP, bivalent spike protein, preservative free, 10 mcg/0.2 mL dosage, diluent reconstituted, tris-sucrose formulation, booster dose
or	procedure	UMLS:CPT:0053A	Immunization administration by intramuscular injection of severe acute respiratory syndrome coronavirus 2 (SARS-CoV-2) (coronavirus disease [COVID-19]) vaccine, mRNA-LNP, spike protein, preservative free, 30 mcg/0.3 mL dosage, tris-sucrose formulation; third dose
or	procedure	UMLS:CPT:1037332	Immunization administration by intramuscular injection of severe acute respiratory syndrome coronavirus 2 (SARS-CoV-2) (coronavirus disease [COVID-19]) vaccine, mRNA-LNP, spike protein, preservative free, 25 mcg/0.25 mL dosage
or	procedure	UMLS:CPT:0052A	Immunization administration by intramuscular injection of severe acute respiratory syndrome coronavirus 2 (SARS-CoV-2) (coronavirus disease [COVID-19]) vaccine, mRNA-LNP, spike protein, preservative free, 30 mcg/0.3 mL dosage, tris-sucrose formulation; second dose
or	procedure	UMLS:CPT:0111A	Immunization administration by intramuscular injection of severe acute respiratory syndrome coronavirus 2 (SARS-

			CoV-2) (coronavirus disease [COVID-19]) vaccine, mRNA-LNP, spike protein, preservative free, 25 mcg/0.25 mL dosage; first dose
or	procedure	UMLS:CPT:0051A	Immunization administration by intramuscular injection of severe acute respiratory syndrome coronavirus 2 (SARS-CoV-2) (coronavirus disease [COVID-19]) vaccine, mRNA-LNP, spike protein, preservative free, 30 mcg/0.3 mL dosage, tris-sucrose formulation; first dose
or	procedure	UMLS:CPT:91311	Severe acute respiratory syndrome coronavirus 2 (SARS-CoV-2) (coronavirus disease [COVID-19]) vaccine, mRNA-LNP, spike protein, preservative free, 25 mcg/0.25 mL dosage, for intramuscular use
or	procedure	UMLS:CPT:0074A	Immunization administration by intramuscular injection of severe acute respiratory syndrome coronavirus 2 (SARS-CoV-2) (coronavirus disease [COVID-19]) vaccine, mRNA-LNP, spike protein, preservative free, 10 mcg/0.2 mL dosage, diluent reconstituted, tris-sucrose formulation; booster dose
or	procedure	UMLS:CPT:0112A	Immunization administration by intramuscular injection of severe acute respiratory syndrome coronavirus 2 (SARS-CoV-2) (coronavirus disease

			[COVID-19]) vaccine, mRNA-LNP, spike protein, preservative free, 25 mcg/0.25 mL dosage; second dose
or	procedure	UMLS:CPT:0083A	Immunization administration by intramuscular injection of severe acute respiratory syndrome coronavirus 2 (SARS-CoV-2) (coronavirus disease [COVID-19]) vaccine, mRNA-LNP, spike protein, preservative free, 3 mcg/0.2 mL dosage, diluent reconstituted, tris-sucrose formulation; third dose
or	procedure	UMLS:CPT:0073A	Immunization administration by intramuscular injection of severe acute respiratory syndrome coronavirus 2 (SARS-CoV-2) (coronavirus disease [COVID-19]) vaccine, mRNA-LNP, spike protein, preservative free, 10 mcg/0.2 mL dosage, diluent reconstituted, tris-sucrose formulation; third dose
or	procedure	UMLS:CPT:0173A	Immunization administration by intramuscular injection of severe acute respiratory syndrome coronavirus 2 (SARS-CoV-2) (coronavirus disease [COVID-19]) vaccine, mRNA-LNP, bivalent spike protein, preservative free, 3 mcg/0.2 mL dosage, diluent reconstituted, tris-sucrose formulation, third dose
or	procedure	UMLS:CPT:0164A	Immunization administration by intramuscular injection of severe acute respiratory

			syndrome coronavirus 2 (SARS-CoV-2) (coronavirus disease [COVID-19]) vaccine, mRNA-LNP, spike protein, bivalent, preservative free, 10 mcg/0.2 mL dosage, booster dose
or	procedure	UMLS:CPT:1037838	Immunization administration by intramuscular injection of severe acute respiratory syndrome coronavirus 2 (SARS-CoV-2) (coronavirus disease [COVID-19]) vaccine, mRNA-LNP, spike protein, preservative free, 50 mcg/0.5 mL dosage
or	procedure	UMLS:CPT:0094A	Immunization administration by intramuscular injection of severe acute respiratory syndrome coronavirus 2 (SARS-CoV-2) (coronavirus disease [COVID-19]) vaccine, mRNA-LNP, spike protein, preservative free, 50 mcg/0.5 mL dosage; booster dose, when administered to individuals 18 years and over
or	procedure	UMLS:CPT:0034A	Immunization administration by intramuscular injection of severe acute respiratory syndrome coronavirus 2 (SARS-CoV-2) (coronavirus disease [COVID-19]) vaccine, DNA, spike protein, adenovirus type 26 (Ad26) vector, preservative free, 5x1010 viral particles/0.5 mL dosage; booster dose
or	procedure	UMLS:CPT:0144A	Immunization administration by intramuscular injection of severe acute respiratory

			syndrome coronavirus 2 (SARS-CoV-2) (coronavirus disease [COVID-19]) vaccine, mRNA-LNP, spike protein, bivalent, preservative free, 25 mcg/0.25 mL dosage, booster dose
or	procedure	UMLS:CPT:0091A	Immunization administration by intramuscular injection of severe acute respiratory syndrome coronavirus 2 (SARS-CoV-2) (coronavirus disease [COVID-19]) vaccine, mRNA-LNP, spike protein, preservative free, 50 mcg/0.5 mL dosage; first dose, when administered to individuals 6 through 11 years
or	procedure	UMLS:CPT:0174A	Immunization administration by intramuscular injection of severe acute respiratory syndrome coronavirus 2 (SARS-CoV-2) (coronavirus disease [COVID-19]) vaccine, mRNA-LNP, bivalent spike protein, preservative free, 3 mcg/0.2 mL dosage, diluent reconstituted, tris-sucrose formulation, booster
or	procedure	UMLS:CPT:0092A	Immunization administration by intramuscular injection of severe acute respiratory syndrome coronavirus 2 (SARS-CoV-2) (coronavirus disease [COVID-19]) vaccine, mRNA-LNP, spike protein, preservative free, 50 mcg/0.5 mL dosage; second dose, when administered to individuals 6 through 11 years

or	procedure	UMLS:CPT:1036682	Immunization administration by intramuscular injection of severe acute respiratory syndrome coronavirus 2 (SARS-CoV-2) (coronavirus disease [COVID-19]) vaccine, recombinant spike protein nanoparticle, saponin-based adjuvant, preservative free, 5 mcg/0.5 mL dosage
or	procedure	UMLS:CPT:0041A	Immunization administration by intramuscular injection of severe acute respiratory syndrome coronavirus 2 (SARS-CoV-2) (coronavirus disease [COVID-19]) vaccine, recombinant spike protein nanoparticle, saponin-based adjuvant, preservative free, 5 mcg/0.5 mL dosage; first dose
or	procedure	UMLS:CPT:0113A	Immunization administration by intramuscular injection of severe acute respiratory syndrome coronavirus 2 (SARS-CoV-2) (coronavirus disease [COVID-19]) vaccine, mRNA-LNP, spike protein, preservative free, 25 mcg/0.25 mL dosage; third dose
or	procedure	UMLS:CPT:0042A	Immunization administration by intramuscular injection of severe acute respiratory syndrome coronavirus 2 (SARS-CoV-2) (coronavirus disease [COVID-19]) vaccine, recombinant spike protein nanoparticle, saponin-based adjuvant, preservative free, 5

				mcg/0.5 mL dosage; second dose
	or	procedure	UMLS:CPT:0093A	Immunization administration by intramuscular injection of severe acute respiratory syndrome coronavirus 2 (SARS-CoV-2) (coronavirus disease [COVID-19]) vaccine, mRNA-LNP, spike protein, preservative free, 50 mcg/0.5 mL dosage; third dose, when administered to individuals 6 through 11 years
	or	procedure	UMLS:CPT:1036666	Immunization administration by intramuscular injection of severe acute respiratory syndrome coronavirus 2 (SARS-CoV-2) (coronavirus disease [COVID-19]) vaccine, DNA, spike protein, chimpanzee adenovirus Oxford 1 (ChAdOx1) vector, preservative free, 5x1010 viral particles/0.5 mL dosage
	or	procedure	UMLS:CPT:0044A	Immunization administration by intramuscular injection of severe acute respiratory syndrome coronavirus 2 (SARS-CoV-2) (coronavirus disease [COVID-19]) vaccine, recombinant spike protein nanoparticle, saponin-based adjuvant, preservative free, 5 mcg/0.5mL dosage; booster
date constraint		The terms in this group occurred between Dec 1, 2019 and Dec 31, 2023		
**Group 2**				
**Group 2A COVID-19 negative**				
must have		visit	TNX:Visit	Visit (Data Source: TriNetX)
cannot have		diagnosis	UMLS:ICD10CM:U07.1	COVID-19

	or	laboratory	UMLS:LNC:95406-5	SARS-CoV-2 (COVID-19) RNA [Presence] in Nose by NAA with probe detection (labResult: Positive)
	or	laboratory	UMLS:LNC:94845-5	SARS-CoV-2 (COVID-19) RNA [Presence] in Saliva (oral fluid) by NAA with probe detection (labResult: Positive)
	or	laboratory	UMLS:LNC:95608-6	SARS-CoV-2 (COVID-19) RNA [Presence] in Respiratory specimen by NAA with non-probe detection (labResult: Positive)
	or	laboratory	UMLS:LNC:94759-8	SARS-CoV-2 (COVID-19) RNA [Presence] in Nasopharynx by NAA with probe detection (labResult: Positive)
	or	laboratory	UMLS:LNC:94565-9	SARS-CoV-2 (COVID-19) RNA [Presence] in Nasopharynx by NAA with non-probe detection (labResult: Positive)
	or	laboratory	UMLS:LNC:94309-2	SARS-CoV-2 (COVID-19) RNA [Presence] in Specimen by NAA with probe detection (labResult: Positive)
	or	laboratory	UMLS:LNC:94500-6	SARS-CoV-2 (COVID-19) RNA [Presence] in Respiratory specimen by NAA with probe detection (labResult: Positive)
	or	diagnosis	UMLS:ICD10CM:U07.2	COVID-19, virus not identified (WHO)
	or	diagnosis	UMLS:ICD10CM:J12.82	Pneumonia due to coronavirus disease 2019
date constraint	The terms in this group occurred at any time			
event relationship	Any instance of thyroid cancer history occurred at least 1 day before the first instance of COVID-19 negative			
**Group 2B thyroid cancer history**				

cannot have		diagnosis	UMLS:ICD10CM:C73	Malignant neoplasm of thyroid gland
	or	diagnosis	UMLS:ICD10CM:Z85.850	Personal history of malignant neoplasm of thyroid

Query Criteria for Cohort post-COVID, female
This query was run on the network Global Collaborative Network with 148 HCO(s) queried and 148 HCO(s) responded. A total of 122 provider(s) responded with patients. The final cohort included 1,343,578 patients who matched the query criteria listed in the table below.

**Ungrouped terms**				
must have		demographics	Age	Age (at least 18 years (most recent occurrence))
	and	demographics	UMLS:HL7V3.0:Gender:F	Female
**Group 1**				
**COVID-19, no vaccination**				
must have	any of	diagnosis	UMLS:ICD10CM:U07.1	COVID-19
		diagnosis	UMLS:ICD10CM:U07.2	COVID-19, virus not identified (WHO)
		diagnosis	UMLS:ICD10CM:J12.82	Pneumonia due to coronavirus disease 2019
		laboratory	UMLS:LNC:94500-6	SARS-CoV-2 (COVID-19) RNA [Presence] in Respiratory specimen by NAA with probe detection (labResult: Positive)
		laboratory	UMLS:LNC:94309-2	SARS-CoV-2 (COVID-19) RNA [Presence] in Specimen by NAA with probe detection (labResult: Positive)
		laboratory	UMLS:LNC:94565-9	SARS-CoV-2 (COVID-19) RNA [Presence] in Nasopharynx by NAA with non-probe detection (labResult: Positive)
		laboratory	UMLS:LNC:94759-8	SARS-CoV-2 (COVID-19) RNA [Presence] in Nasopharynx by

				NAA with probe detection (labResult: Positive)
		laboratory	UMLS:LNC:95608-6	SARS-CoV-2 (COVID-19) RNA [Presence] in Respiratory specimen by NAA with non-probe detection (labResult: Positive)
		laboratory	UMLS:LNC:94845-5	SARS-CoV-2 (COVID-19) RNA [Presence] in Saliva (oral fluid) by NAA with probe detection (labResult: Positive)
		laboratory	UMLS:LNC:95406-5	SARS-CoV-2 (COVID-19) RNA [Presence] in Nose by NAA with probe detection (labResult: Positive)
cannot have		medication	NLM:CVX:208	COVID-19, mRNA, LNP-S, PF, 30 mcg/0.3 mL dose
	or	medication	NLM:CVX:207	COVID-19, mRNA, LNP-S, PF, 100 mcg/0.5mL dose or 50 mcg/0.25mL dose
	or	medication	NLM:CVX:212	COVID-19 vaccine, vector-nr, rS-Ad26, PF, 0.5 mL
	or	medication	NLM:RXNORM:OMOP 5042939	COVID-19 vaccine
	or	medication	NLM:CVX:300	COVID-19, mRNA, LNP-S, bivalent, PF, 30 mcg/0.3 mL dose
	or	medication	NLM:CVX:217	COVID-19, mRNA, LNP-S, PF, 30 mcg/0.3 mL dose, tris-sucrose
	or	medication	NLM:CVX:229	COVID-19, mRNA, LNP-S, bivalent, PF, 50 mcg/0.5 mL or 25mcg/0.25 mL dose
	or	medication	NLM:CVX:218	COVID-19, mRNA, LNP-S, PF, 10 mcg/0.2 mL dose, tris-sucrose
	or	medication	NLM:CVX:520	COVID-19 mRNA, bivalent, original/Omicron BA.1, Non-US Vaccine Product, Pfizer-BioNTech

or	medication	NLM:CVX:519	COVID-19 mRNA, bivalent, original/Omicron BA.1, Non-US Vaccine (Spikevax Bivalent), Moderna
or	medication	NLM:CVX:301	COVID-19, mRNA, LNP-S, bivalent, PF, 10 mcg/0.2 mL dose
or	medication	NLM:CVX:219	COVID-19, mRNA, LNP-S, PF, 3 mcg/0.2 mL dose, tris-sucrose
or	medication	NLM:CVX:228	COVID-19, mRNA, LNP-S, PF, pediatric 25 mcg/0.25 mL dose
or	medication	NLM:CVX:230	COVID-19, mRNA, LNP-S, bivalent booster, PF, 10 mcg/0.2 mL
or	medication	NLM:CVX:221	COVID-19, mRNA, LNP-S, PF, 50 mcg/0.5 mL dose
or	medication	NLM:CVX:210	COVID-19 vaccine, vector-nr, rS-ChAdOx1, PF, 0.5 mL
or	medication	NLM:CVX:302	COVID-19, mRNA, LNP-S, bivalent, PF, 3 mcg/0.2 mL dose
or	medication	NLM:CVX:511	COVID-19 IV Non-US Vaccine (CoronaVac, Sinovac)
or	medication	NLM:RXNORM:2468231	SARS-CoV-2 (COVID-19) vaccine, mRNA spike protein
or	procedure	UMLS:CPT:91300	Severe acute respiratory syndrome coronavirus 2 (SARS-CoV-2) (coronavirus disease [COVID-19]) vaccine, mRNA-LNP, spike protein, preservative free, 30 mcg/0.3 mL dosage, diluent reconstituted, for intramuscular use
or	procedure	UMLS:CPT:0001A	Immunization administration by intramuscular injection of severe acute respiratory syndrome coronavirus 2 (SARS-CoV-2) (coronavirus disease [COVID-19]) vaccine, mRNA-

			LNP, spike protein, preservative free, 30 mcg/0.3 mL dosage, diluent reconstituted; first dose
or	procedure	UMLS:CPT:0002A	Immunization administration by intramuscular injection of severe acute respiratory syndrome coronavirus 2 (SARS-CoV-2) (coronavirus disease [COVID-19]) vaccine, mRNA-LNP, spike protein, preservative free, 30 mcg/0.3 mL dosage, diluent reconstituted; second dose
or	procedure	UMLS:CPT:91301	Severe acute respiratory syndrome coronavirus 2 (SARS-CoV-2) (coronavirus disease [COVID-19]) vaccine, mRNA-LNP, spike protein, preservative free, 100 mcg/0.5 mL dosage, for intramuscular use
or	procedure	UMLS:CPT:0011A	Immunization administration by intramuscular injection of severe acute respiratory syndrome coronavirus 2 (SARS-CoV-2) (coronavirus disease [COVID-19]) vaccine, mRNA-LNP, spike protein, preservative free, 100 mcg/0.5 mL dosage; first dose
or	procedure	UMLS:CPT:0012A	Immunization administration by intramuscular injection of severe acute respiratory syndrome coronavirus 2 (SARS-CoV-2) (coronavirus disease [COVID-19]) vaccine, mRNA-LNP, spike protein, preservative free, 100 mcg/0.5 mL dosage; second dose

or	procedure	UMLS:SNOMED:840534001	Administration of SARS-CoV-2 antigen vaccine
or	medication	NLM:CVX:213	SARS-CoV-2 (COVID-19) Vaccine
or	procedure	UMLS:CPT:1036660	Immunization administration by intramuscular injection of severe acute respiratory syndrome coronavirus 2 (SARS-CoV-2) (coronavirus disease [COVID-19]) vaccine, mRNA-LNP, spike protein, preservative free, 30 mcg/0.3 mL dosage, diluent reconstituted
or	procedure	UMLS:CPT:1036663	Immunization administration by intramuscular injection of severe acute respiratory syndrome coronavirus 2 (SARS-CoV-2) (coronavirus disease [COVID-19]) vaccine, mRNA-LNP, spike protein, preservative free, 100 mcg/0.5 mL dosage
or	procedure	UMLS:CPT:0124A	Immunization administration by intramuscular injection of severe acute respiratory syndrome coronavirus 2 (SARS-CoV-2) (coronavirus disease [COVID-19]) vaccine, mRNA-LNP, bivalent spike protein, preservative free, 30 mcg/0.3 mL dosage, tris-sucrose formulation, booster dose
or	procedure	UMLS:CPT:0004A	Immunization administration by intramuscular injection of severe acute respiratory syndrome coronavirus 2 (SARS-CoV-2) (coronavirus disease [COVID-19]) vaccine, mRNA-LNP, spike protein, preservative

			free, 30 mcg/0.3 mL dosage, diluent reconstituted; booster dose
or	procedure	UMLS:CPT:0003A	Immunization administration by intramuscular injection of severe acute respiratory syndrome coronavirus 2 (SARS-CoV-2) (coronavirus disease [COVID-19]) vaccine, mRNA-LNP, spike protein, preservative free, 30 mcg/0.3 mL dosage, diluent reconstituted; third dose
or	procedure	UMLS:CPT:1037166	Immunization administration by intramuscular injection of severe acute respiratory syndrome coronavirus 2 (SARS-CoV-2) (coronavirus disease [COVID-19]) vaccine, mRNA-LNP, spike protein, preservative free, 30 mcg/0.3 mL dosage, tris-sucrose formulation
or	procedure	UMLS:CPT:0054A	Immunization administration by intramuscular injection of severe acute respiratory syndrome coronavirus 2 (SARS-CoV-2) (coronavirus disease [COVID-19]) vaccine, mRNA-LNP, spike protein, preservative free, 30 mcg/0.3 mL dosage, tris-sucrose formulation; booster dose
or	procedure	UMLS:CPT:0064A	Immunization administration by intramuscular injection of severe acute respiratory syndrome coronavirus 2 (SARS-CoV-2) (coronavirus disease [COVID-19]) vaccine, mRNA-LNP, spike protein, preservative

			free, 50 mcg/0.25 mL dosage, booster dose
or	procedure	UMLS:CPT:90480	Immunization administration by intramuscular injection of severe acute respiratory syndrome coronavirus 2 (SARS-CoV-2) (coronavirus disease [COVID-19]) vaccine, single dose
or	procedure	UMLS:CPT:1037171	Immunization administration by intramuscular injection of severe acute respiratory syndrome coronavirus 2 (SARS-CoV-2) (coronavirus disease [COVID-19]) vaccine, mRNA-LNP, spike protein, preservative free, 10 mcg/0.2 mL dosage, diluent reconstituted, tris-sucrose formulation
or	procedure	UMLS:CPT:0071A	Immunization administration by intramuscular injection of severe acute respiratory syndrome coronavirus 2 (SARS-CoV-2) (coronavirus disease [COVID-19]) vaccine, mRNA-LNP, spike protein, preservative free, 10 mcg/0.2 mL dosage, diluent reconstituted, tris-sucrose formulation; first dose
or	procedure	UMLS:CPT:0072A	Immunization administration by intramuscular injection of severe acute respiratory syndrome coronavirus 2 (SARS-CoV-2) (coronavirus disease [COVID-19]) vaccine, mRNA-LNP, spike protein, preservative free, 10 mcg/0.2 mL dosage, diluent reconstituted, tris-

			sucrose formulation; second dose
or	medication	NLM:RXNORM:2610319	SARS-CoV-2 (COVID-19) vaccine, mRNA-BNT162b2 0.05 MG/ML / SARS-CoV-2 (COVID-19) vaccine, mRNA-BNT162b2 OMICRON (BA.4/BA.5) 0.05 MG/ML Injectable Suspension
or	procedure	UMLS:CPT:91313	Severe acute respiratory syndrome coronavirus 2 (SARS-CoV-2) (coronavirus disease [COVID-19]) vaccine, mRNA-LNP, spike protein, bivalent, preservative free, 50 mcg/0.5 mL dosage, for intramuscular use
or	procedure	UMLS:CPT:0134A	Immunization administration by intramuscular injection of severe acute respiratory syndrome coronavirus 2 (SARS-CoV-2) (coronavirus disease [COVID-19]) vaccine, mRNA-LNP, spike protein, bivalent, preservative free, 50 mcg/0.5 mL dosage, booster dose
or	procedure	UMLS:CPT:1037175	Immunization administration by intramuscular injection of severe acute respiratory syndrome coronavirus 2 (SARS-CoV-2) (coronavirus disease [COVID-19]) vaccine, DNA, spike protein, adenovirus type 26 (Ad26) vector, preservative free, 5x1010 viral particles/0.5 mL dosage
or	medication	NLM:RXNORM:2610347	0.3 ML SARS-CoV-2 (COVID-19) vaccine, mRNA-BNT162b2 0.05 MG/ML / SARS-CoV-2 (COVID-19) vaccine, mRNA-BNT162b2

			OMICRON (BA.4/BA.5) -1 MG/ML Injection
or	procedure	UMLS:CPT:1037228	Immunization administration by intramuscular injection of severe acute respiratory syndrome coronavirus 2 (SARS-CoV-2) (coronavirus disease [COVID-19]) vaccine, mRNA-LNP, spike protein, preservative free, 3 mcg/0.2 mL dosage, diluent reconstituted, tris-sucrose formulation
or	procedure	UMLS:CPT:0013A	Immunization administration by intramuscular injection of severe acute respiratory syndrome coronavirus 2 (SARS-CoV-2) (coronavirus disease [COVID-19]) vaccine, mRNA-LNP, spike protein, preservative free, 100 mcg/0.5 mL dosage; third dose
or	procedure	UMLS:CPT:0081A	Immunization administration by intramuscular injection of severe acute respiratory syndrome coronavirus 2 (SARS-CoV-2) (coronavirus disease [COVID-19]) vaccine, mRNA-LNP, spike protein, preservative free, 3 mcg/0.2 mL dosage, diluent reconstituted, tris-sucrose formulation; first dose
or	procedure	UMLS:CPT:0082A	Immunization administration by intramuscular injection of severe acute respiratory syndrome coronavirus 2 (SARS-CoV-2) (coronavirus disease [COVID-19]) vaccine, mRNA-LNP, spike protein, preservative

			free, 3 mcg/0.2 mL dosage, diluent reconstituted, tris-sucrose formulation; second dose
or	medication	NLM:RXNORM:2610328	SARS-CoV-2 (COVID-19) vaccine, mRNA-1273 0.05 MG/ML / SARS-CoV-2 (COVID-19) vaccine, mRNA-1273 OMICRON (BA.4/BA.5) 0.05 MG/ML Injectable Suspension
or	procedure	UMLS:CPT:0154A	Immunization administration by intramuscular injection of severe acute respiratory syndrome coronavirus 2 (SARS-CoV-2) (coronavirus disease [COVID-19]) vaccine, mRNA-LNP, bivalent spike protein, preservative free, 10 mcg/0.2 mL dosage, diluent reconstituted, tris-sucrose formulation, booster dose
or	procedure	UMLS:CPT:0053A	Immunization administration by intramuscular injection of severe acute respiratory syndrome coronavirus 2 (SARS-CoV-2) (coronavirus disease [COVID-19]) vaccine, mRNA-LNP, spike protein, preservative free, 30 mcg/0.3 mL dosage, tris-sucrose formulation; third dose
or	procedure	UMLS:CPT:1037332	Immunization administration by intramuscular injection of severe acute respiratory syndrome coronavirus 2 (SARS-CoV-2) (coronavirus disease [COVID-19]) vaccine, mRNA-

			LNP, spike protein, preservative free, 25 mcg/0.25 mL dosage
or	procedure	UMLS:CPT:0052A	Immunization administration by intramuscular injection of severe acute respiratory syndrome coronavirus 2 (SARS-CoV-2) (coronavirus disease [COVID-19]) vaccine, mRNA-LNP, spike protein, preservative free, 30 mcg/0.3 mL dosage, tris-sucrose formulation; second dose
or	procedure	UMLS:CPT:0111A	Immunization administration by intramuscular injection of severe acute respiratory syndrome coronavirus 2 (SARS-CoV-2) (coronavirus disease [COVID-19]) vaccine, mRNA-LNP, spike protein, preservative free, 25 mcg/0.25 mL dosage; first dose
or	procedure	UMLS:CPT:0051A	Immunization administration by intramuscular injection of severe acute respiratory syndrome coronavirus 2 (SARS-CoV-2) (coronavirus disease [COVID-19]) vaccine, mRNA-LNP, spike protein, preservative free, 30 mcg/0.3 mL dosage, tris-sucrose formulation; first dose
or	procedure	UMLS:CPT:91311	Severe acute respiratory syndrome coronavirus 2 (SARS-CoV-2) (coronavirus disease [COVID-19]) vaccine, mRNA-LNP, spike protein, preservative free, 25 mcg/0.25 mL dosage, for intramuscular use

or	procedure	UMLS:CPT:0074A	Immunization administration by intramuscular injection of severe acute respiratory syndrome coronavirus 2 (SARS-CoV-2) (coronavirus disease [COVID-19]) vaccine, mRNA-LNP, spike protein, preservative free, 10 mcg/0.2 mL dosage, diluent reconstituted, tris-sucrose formulation; booster dose
or	procedure	UMLS:CPT:0112A	Immunization administration by intramuscular injection of severe acute respiratory syndrome coronavirus 2 (SARS-CoV-2) (coronavirus disease [COVID-19]) vaccine, mRNA-LNP, spike protein, preservative free, 25 mcg/0.25 mL dosage; second dose
or	procedure	UMLS:CPT:0083A	Immunization administration by intramuscular injection of severe acute respiratory syndrome coronavirus 2 (SARS-CoV-2) (coronavirus disease [COVID-19]) vaccine, mRNA-LNP, spike protein, preservative free, 3 mcg/0.2 mL dosage, diluent reconstituted, tris-sucrose formulation; third dose
or	procedure	UMLS:CPT:0073A	Immunization administration by intramuscular injection of severe acute respiratory syndrome coronavirus 2 (SARS-CoV-2) (coronavirus disease [COVID-19]) vaccine, mRNA-LNP, spike protein, preservative free, 10 mcg/0.2 mL dosage,

			diluent reconstituted, tris-sucrose formulation; third dose
or	procedure	UMLS:CPT:0173A	Immunization administration by intramuscular injection of severe acute respiratory syndrome coronavirus 2 (SARS-CoV-2) (coronavirus disease [COVID-19]) vaccine, mRNA-LNP, bivalent spike protein, preservative free, 3 mcg/0.2 mL dosage, diluent reconstituted, tris-sucrose formulation, third dose
or	procedure	UMLS:CPT:0164A	Immunization administration by intramuscular injection of severe acute respiratory syndrome coronavirus 2 (SARS-CoV-2) (coronavirus disease [COVID-19]) vaccine, mRNA-LNP, spike protein, bivalent, preservative free, 10 mcg/0.2 mL dosage, booster dose
or	procedure	UMLS:CPT:1037838	Immunization administration by intramuscular injection of severe acute respiratory syndrome coronavirus 2 (SARS-CoV-2) (coronavirus disease [COVID-19]) vaccine, mRNA-LNP, spike protein, preservative free, 50 mcg/0.5 mL dosage
or	procedure	UMLS:CPT:0094A	Immunization administration by intramuscular injection of severe acute respiratory syndrome coronavirus 2 (SARS-CoV-2) (coronavirus disease [COVID-19]) vaccine, mRNA-LNP, spike protein, preservative free, 50 mcg/0.5 mL dosage;

			booster dose, when administered to individuals 18 years and over
or	procedure	UMLS:CPT:0034A	Immunization administration by intramuscular injection of severe acute respiratory syndrome coronavirus 2 (SARS-CoV-2) (coronavirus disease [COVID-19]) vaccine, DNA, spike protein, adenovirus type 26 (Ad26) vector, preservative free, 5x1010 viral particles/0.5 mL dosage; booster dose
or	procedure	UMLS:CPT:0144A	Immunization administration by intramuscular injection of severe acute respiratory syndrome coronavirus 2 (SARS-CoV-2) (coronavirus disease [COVID-19]) vaccine, mRNA-LNP, spike protein, bivalent, preservative free, 25 mcg/0.25 mL dosage, booster dose
or	procedure	UMLS:CPT:0091A	Immunization administration by intramuscular injection of severe acute respiratory syndrome coronavirus 2 (SARS-CoV-2) (coronavirus disease [COVID-19]) vaccine, mRNA-LNP, spike protein, preservative free, 50 mcg/0.5 mL dosage; first dose, when administered to individuals 6 through 11 years
or	procedure	UMLS:CPT:0174A	Immunization administration by intramuscular injection of severe acute respiratory syndrome coronavirus 2 (SARS-CoV-2) (coronavirus disease [COVID-19]) vaccine, mRNA-

			LNP, bivalent spike protein, preservative free, 3 mcg/0.2 mL dosage, diluent reconstituted, tris-sucrose formulation, booster
or	procedure	UMLS:CPT:0092A	Immunization administration by intramuscular injection of severe acute respiratory syndrome coronavirus 2 (SARS-CoV-2) (coronavirus disease [COVID-19]) vaccine, mRNA-LNP, spike protein, preservative free, 50 mcg/0.5 mL dosage; second dose, when administered to individuals 6 through 11 years
or	procedure	UMLS:CPT:1036682	Immunization administration by intramuscular injection of severe acute respiratory syndrome coronavirus 2 (SARS-CoV-2) (coronavirus disease [COVID-19]) vaccine, recombinant spike protein nanoparticle, saponin-based adjuvant, preservative free, 5 mcg/0.5 mL dosage
or	procedure	UMLS:CPT:0041A	Immunization administration by intramuscular injection of severe acute respiratory syndrome coronavirus 2 (SARS-CoV-2) (coronavirus disease [COVID-19]) vaccine, recombinant spike protein nanoparticle, saponin-based adjuvant, preservative free, 5 mcg/0.5 mL dosage; first dose
or	procedure	UMLS:CPT:0113A	Immunization administration by intramuscular injection of

			severe acute respiratory syndrome coronavirus 2 (SARS-CoV-2) (coronavirus disease [COVID-19]) vaccine, mRNA-LNP, spike protein, preservative free, 25 mcg/0.25 mL dosage; third dose
or	procedure	UMLS:CPT:0042A	Immunization administration by intramuscular injection of severe acute respiratory syndrome coronavirus 2 (SARS-CoV-2) (coronavirus disease [COVID-19]) vaccine, recombinant spike protein nanoparticle, saponin-based adjuvant, preservative free, 5 mcg/0.5 mL dosage; second dose
or	procedure	UMLS:CPT:0093A	Immunization administration by intramuscular injection of severe acute respiratory syndrome coronavirus 2 (SARS-CoV-2) (coronavirus disease [COVID-19]) vaccine, mRNA-LNP, spike protein, preservative free, 50 mcg/0.5 mL dosage; third dose, when administered to individuals 6 through 11 years
or	procedure	UMLS:CPT:1036666	Immunization administration by intramuscular injection of severe acute respiratory syndrome coronavirus 2 (SARS-CoV-2) (coronavirus disease [COVID-19]) vaccine, DNA, spike protein, chimpanzee adenovirus Oxford 1 (ChAdOx1) vector, preservative free, 5x1010 viral particles/0.5 mL dosage

	or	procedure	UMLS:CPT:0044A	Immunization administration by intramuscular injection of severe acute respiratory syndrome coronavirus 2 (SARS-CoV-2) (coronavirus disease [COVID-19]) vaccine, recombinant spike protein nanoparticle, saponin-based adjuvant, preservative free, 5 mcg/0.5mL dosage; booster
date constraint		The terms in this group occurred between Dec 1, 2019 and Dec 31, 2023		
**Group 2**				
**Group 2A COVID-19**				
must have	any of	diagnosis	UMLS:ICD10CM:U07.1	COVID-19
		diagnosis	UMLS:ICD10CM:U07.2	COVID-19, virus not identified (WHO)
		diagnosis	UMLS:ICD10CM:J12.82	Pneumonia due to coronavirus disease 2019
		laboratory	UMLS:LNC:94500-6	SARS-CoV-2 (COVID-19) RNA [Presence] in Respiratory specimen by NAA with probe detection (labResult: Positive)
		laboratory	UMLS:LNC:94309-2	SARS-CoV-2 (COVID-19) RNA [Presence] in Specimen by NAA with probe detection (labResult: Positive)
		laboratory	UMLS:LNC:94565-9	SARS-CoV-2 (COVID-19) RNA [Presence] in Nasopharynx by NAA with non-probe detection (labResult: Positive)
		laboratory	UMLS:LNC:94759-8	SARS-CoV-2 (COVID-19) RNA [Presence] in Nasopharynx by NAA with probe detection (labResult: Positive)
		laboratory	UMLS:LNC:95608-6	SARS-CoV-2 (COVID-19) RNA [Presence] in Respiratory specimen by NAA with non-

				probe detection (labResult: Positive)
		laboratory	UMLS:LNC:94845-5	SARS-CoV-2 (COVID-19) RNA [Presence] in Saliva (oral fluid) by NAA with probe detection (labResult: Positive)
		laboratory	UMLS:LNC:95406-5	SARS-CoV-2 (COVID-19) RNA [Presence] in Nose by NAA with probe detection (labResult: Positive)
	and	visit	TNX:Visit	Visit (Data Source: TriNetX)
date constraint		The terms in this group occurred at any time		
event relationship		Any instance of thyroid cancer history occurred at least 1 day before the first instance of COVID-19		
**Group 2B thyroid cancer history**				
cannot have		diagnosis	UMLS:ICD10CM:C73	Malignant neoplasm of thyroid gland
	or	diagnosis	UMLS:ICD10CM:Z85.850	Personal history of malignant neoplasm of thyroid

Query Criteria for Cohort non-COVID, female

This query was run on the network Global Collaborative Network with 148 HCO(s) queried and 148 HCO(s) responded. A total of 131 provider(s) responded with patients. The final cohort included 16,973,610 patients who matched the query criteria listed in the table below.

**Ungrouped terms**				
must have		demographics	Age	Age (at least 18 years (most recent occurrence))
	and	demographics	UMLS:HL7V3.0:Gender:F	Female
**Group 1**				
**COVID-19 negative, no vaccination**				
must have		visit	TNX:Visit	Visit
cannot have		medication	NLM:CVX:208	COVID-19, mRNA, LNP-S, PF, 30 mcg/0.3 mL dose

or	medication	NLM:CVX:207	COVID-19, mRNA, LNP-S, PF, 100 mcg/0.5mL dose or 50 mcg/0.25mL dose
or	medication	NLM:CVX:212	COVID-19 vaccine, vector-nr, rS-Ad26, PF, 0.5 mL
or	medication	NLM:RXNORM:OMOP 5042939	COVID-19 vaccine
or	medication	NLM:CVX:300	COVID-19, mRNA, LNP-S, bivalent, PF, 30 mcg/0.3 mL dose
or	medication	NLM:CVX:217	COVID-19, mRNA, LNP-S, PF, 30 mcg/0.3 mL dose, tris-sucrose
or	medication	NLM:CVX:229	COVID-19, mRNA, LNP-S, bivalent, PF, 50 mcg/0.5 mL or 25mcg/0.25 mL dose
or	medication	NLM:CVX:218	COVID-19, mRNA, LNP-S, PF, 10 mcg/0.2 mL dose, tris-sucrose
or	medication	NLM:CVX:520	COVID-19 mRNA, bivalent, original/Omicron BA.1, Non-US Vaccine Product, Pfizer-BioNTech
or	medication	NLM:CVX:519	COVID-19 mRNA, bivalent, original/Omicron BA.1, Non-US Vaccine (Spikevax Bivalent), Moderna
or	medication	NLM:CVX:301	COVID-19, mRNA, LNP-S, bivalent, PF, 10 mcg/0.2 mL dose
or	medication	NLM:CVX:219	COVID-19, mRNA, LNP-S, PF, 3 mcg/0.2 mL dose, tris-sucrose
or	medication	NLM:CVX:228	COVID-19, mRNA, LNP-S, PF, pediatric 25 mcg/0.25 mL dose
or	medication	NLM:CVX:230	COVID-19, mRNA, LNP-S, bivalent booster, PF, 10 mcg/0.2 mL
or	medication	NLM:CVX:221	COVID-19, mRNA, LNP-S, PF, 50 mcg/0.5 mL dose

or	medication	NLM:CVX:210	COVID-19 vaccine, vector-nr, rS-ChAdOx1, PF, 0.5 mL
or	medication	NLM:CVX:302	COVID-19, mRNA, LNP-S, bivalent, PF, 3 mcg/0.2 mL dose
or	medication	NLM:CVX:511	COVID-19 IV Non-US Vaccine (CoronaVac, Sinovac)
or	medication	NLM:RXNORM:2468231	SARS-CoV-2 (COVID-19) vaccine, mRNA spike protein
or	procedure	UMLS:CPT:91300	Severe acute respiratory syndrome coronavirus 2 (SARS-CoV-2) (coronavirus disease [COVID-19]) vaccine, mRNA-LNP, spike protein, preservative free, 30 mcg/0.3 mL dosage, diluent reconstituted, for intramuscular use
or	procedure	UMLS:CPT:0001A	Immunization administration by intramuscular injection of severe acute respiratory syndrome coronavirus 2 (SARS-CoV-2) (coronavirus disease [COVID-19]) vaccine, mRNA-LNP, spike protein, preservative free, 30 mcg/0.3 mL dosage, diluent reconstituted; first dose
or	procedure	UMLS:CPT:0002A	Immunization administration by intramuscular injection of severe acute respiratory syndrome coronavirus 2 (SARS-CoV-2) (coronavirus disease [COVID-19]) vaccine, mRNA-LNP, spike protein, preservative free, 30 mcg/0.3 mL dosage, diluent reconstituted; second dose
or	procedure	UMLS:CPT:91301	Severe acute respiratory syndrome coronavirus 2 (SARS-CoV-2) (coronavirus disease

			[COVID-19]) vaccine, mRNA-LNP, spike protein, preservative free, 100 mcg/0.5 mL dosage, for intramuscular use
or	procedure	UMLS:CPT:0011A	Immunization administration by intramuscular injection of severe acute respiratory syndrome coronavirus 2 (SARS-CoV-2) (coronavirus disease [COVID-19]) vaccine, mRNA-LNP, spike protein, preservative free, 100 mcg/0.5 mL dosage; first dose
or	procedure	UMLS:CPT:0012A	Immunization administration by intramuscular injection of severe acute respiratory syndrome coronavirus 2 (SARS-CoV-2) (coronavirus disease [COVID-19]) vaccine, mRNA-LNP, spike protein, preservative free, 100 mcg/0.5 mL dosage; second dose
or	procedure	UMLS:SNOMED:840534001	Administration of SARS-CoV-2 antigen vaccine
or	medication	NLM:CVX:213	SARS-CoV-2 (COVID-19) Vaccine
or	procedure	UMLS:CPT:1036660	Immunization administration by intramuscular injection of severe acute respiratory syndrome coronavirus 2 (SARS-CoV-2) (coronavirus disease [COVID-19]) vaccine, mRNA-LNP, spike protein, preservative free, 30 mcg/0.3 mL dosage, diluent reconstituted
or	procedure	UMLS:CPT:1036663	Immunization administration by intramuscular injection of severe acute respiratory

			syndrome coronavirus 2 (SARS-CoV-2) (coronavirus disease [COVID-19]) vaccine, mRNA-LNP, spike protein, preservative free, 100 mcg/0.5 mL dosage
or	procedure	UMLS:CPT:0124A	Immunization administration by intramuscular injection of severe acute respiratory syndrome coronavirus 2 (SARS-CoV-2) (coronavirus disease [COVID-19]) vaccine, mRNA-LNP, bivalent spike protein, preservative free, 30 mcg/0.3 mL dosage, tris-sucrose formulation, booster dose
or	procedure	UMLS:CPT:0004A	Immunization administration by intramuscular injection of severe acute respiratory syndrome coronavirus 2 (SARS-CoV-2) (coronavirus disease [COVID-19]) vaccine, mRNA-LNP, spike protein, preservative free, 30 mcg/0.3 mL dosage, diluent reconstituted; booster dose
or	procedure	UMLS:CPT:0003A	Immunization administration by intramuscular injection of severe acute respiratory syndrome coronavirus 2 (SARS-CoV-2) (coronavirus disease [COVID-19]) vaccine, mRNA-LNP, spike protein, preservative free, 30 mcg/0.3 mL dosage, diluent reconstituted; third dose
or	procedure	UMLS:CPT:1037166	Immunization administration by intramuscular injection of severe acute respiratory syndrome coronavirus 2 (SARS-

			CoV-2) (coronavirus disease [COVID-19]) vaccine, mRNA-LNP, spike protein, preservative free, 30 mcg/0.3 mL dosage, tris-sucrose formulation
or	procedure	UMLS:CPT:0054A	Immunization administration by intramuscular injection of severe acute respiratory syndrome coronavirus 2 (SARS-CoV-2) (coronavirus disease [COVID-19]) vaccine, mRNA-LNP, spike protein, preservative free, 30 mcg/0.3 mL dosage, tris-sucrose formulation; booster dose
or	procedure	UMLS:CPT:0064A	Immunization administration by intramuscular injection of severe acute respiratory syndrome coronavirus 2 (SARS-CoV-2) (coronavirus disease [COVID-19]) vaccine, mRNA-LNP, spike protein, preservative free, 50 mcg/0.25 mL dosage, booster dose
or	procedure	UMLS:CPT:90480	Immunization administration by intramuscular injection of severe acute respiratory syndrome coronavirus 2 (SARS-CoV-2) (coronavirus disease [COVID-19]) vaccine, single dose
or	procedure	UMLS:CPT:1037171	Immunization administration by intramuscular injection of severe acute respiratory syndrome coronavirus 2 (SARS-CoV-2) (coronavirus disease [COVID-19]) vaccine, mRNA-LNP, spike protein, preservative

			free, 10 mcg/0.2 mL dosage, diluent reconstituted, tris-sucrose formulation
or	procedure	UMLS:CPT:0071A	Immunization administration by intramuscular injection of severe acute respiratory syndrome coronavirus 2 (SARS-CoV-2) (coronavirus disease [COVID-19]) vaccine, mRNA-LNP, spike protein, preservative free, 10 mcg/0.2 mL dosage, diluent reconstituted, tris-sucrose formulation; first dose
or	procedure	UMLS:CPT:0072A	Immunization administration by intramuscular injection of severe acute respiratory syndrome coronavirus 2 (SARS-CoV-2) (coronavirus disease [COVID-19]) vaccine, mRNA-LNP, spike protein, preservative free, 10 mcg/0.2 mL dosage, diluent reconstituted, tris-sucrose formulation; second dose
or	medication	NLM:RXNORM:2610319	SARS-CoV-2 (COVID-19) vaccine, mRNA-BNT162b2 0.05 MG/ML / SARS-CoV-2 (COVID-19) vaccine, mRNA-BNT162b2 OMICRON (BA.4/BA.5) 0.05 MG/ML Injectable Suspension
or	procedure	UMLS:CPT:91313	Severe acute respiratory syndrome coronavirus 2 (SARS-CoV-2) (coronavirus disease [COVID-19]) vaccine, mRNA-LNP, spike protein, bivalent, preservative free, 50 mcg/0.5 mL dosage, for intramuscular use

or	procedure	UMLS:CPT:0134A	Immunization administration by intramuscular injection of severe acute respiratory syndrome coronavirus 2 (SARS-CoV-2) (coronavirus disease [COVID-19]) vaccine, mRNA-LNP, spike protein, bivalent, preservative free, 50 mcg/0.5 mL dosage, booster dose
or	procedure	UMLS:CPT:1037175	Immunization administration by intramuscular injection of severe acute respiratory syndrome coronavirus 2 (SARS-CoV-2) (coronavirus disease [COVID-19]) vaccine, DNA, spike protein, adenovirus type 26 (Ad26) vector, preservative free, 5x1010 viral particles/0.5 mL dosage
or	medication	NLM:RXNORM:2610347	0.3 ML SARS-CoV-2 (COVID-19) vaccine, mRNA-BNT162b2 0.05 MG/ML / SARS-CoV-2 (COVID-19) vaccine, mRNA-BNT162b2 OMICRON (BA.4/BA.5) -1 MG/ML Injection
or	procedure	UMLS:CPT:1037228	Immunization administration by intramuscular injection of severe acute respiratory syndrome coronavirus 2 (SARS-CoV-2) (coronavirus disease [COVID-19]) vaccine, mRNA-LNP, spike protein, preservative free, 3 mcg/0.2 mL dosage, diluent reconstituted, tris-sucrose formulation
or	procedure	UMLS:CPT:0013A	Immunization administration by intramuscular injection of severe acute respiratory

			syndrome coronavirus 2 (SARS-CoV-2) (coronavirus disease [COVID-19]) vaccine, mRNA-LNP, spike protein, preservative free, 100 mcg/0.5 mL dosage; third dose
or	procedure	UMLS:CPT:0081A	Immunization administration by intramuscular injection of severe acute respiratory syndrome coronavirus 2 (SARS-CoV-2) (coronavirus disease [COVID-19]) vaccine, mRNA-LNP, spike protein, preservative free, 3 mcg/0.2 mL dosage, diluent reconstituted, tris-sucrose formulation; first dose
or	procedure	UMLS:CPT:0082A	Immunization administration by intramuscular injection of severe acute respiratory syndrome coronavirus 2 (SARS-CoV-2) (coronavirus disease [COVID-19]) vaccine, mRNA-LNP, spike protein, preservative free, 3 mcg/0.2 mL dosage, diluent reconstituted, tris-sucrose formulation; second dose
or	medication	NLM:RXNORM:2610328	SARS-CoV-2 (COVID-19) vaccine, mRNA-1273 0.05 MG/ML / SARS-CoV-2 (COVID-19) vaccine, mRNA-1273 OMICRON (BA.4/BA.5) 0.05 MG/ML Injectable Suspension
or	procedure	UMLS:CPT:0154A	Immunization administration by intramuscular injection of severe acute respiratory syndrome coronavirus 2 (SARS-CoV-2) (coronavirus disease

			[COVID-19]) vaccine, mRNA-LNP, bivalent spike protein, preservative free, 10 mcg/0.2 mL dosage, diluent reconstituted, tris-sucrose formulation, booster dose
or	procedure	UMLS:CPT:0053A	Immunization administration by intramuscular injection of severe acute respiratory syndrome coronavirus 2 (SARS-CoV-2) (coronavirus disease [COVID-19]) vaccine, mRNA-LNP, spike protein, preservative free, 30 mcg/0.3 mL dosage, tris-sucrose formulation; third dose
or	procedure	UMLS:CPT:1037332	Immunization administration by intramuscular injection of severe acute respiratory syndrome coronavirus 2 (SARS-CoV-2) (coronavirus disease [COVID-19]) vaccine, mRNA-LNP, spike protein, preservative free, 25 mcg/0.25 mL dosage
or	procedure	UMLS:CPT:0052A	Immunization administration by intramuscular injection of severe acute respiratory syndrome coronavirus 2 (SARS-CoV-2) (coronavirus disease [COVID-19]) vaccine, mRNA-LNP, spike protein, preservative free, 30 mcg/0.3 mL dosage, tris-sucrose formulation; second dose
or	procedure	UMLS:CPT:0111A	Immunization administration by intramuscular injection of severe acute respiratory syndrome coronavirus 2 (SARS-

			CoV-2) (coronavirus disease [COVID-19]) vaccine, mRNA-LNP, spike protein, preservative free, 25 mcg/0.25 mL dosage; first dose
or	procedure	UMLS:CPT:0051A	Immunization administration by intramuscular injection of severe acute respiratory syndrome coronavirus 2 (SARS-CoV-2) (coronavirus disease [COVID-19]) vaccine, mRNA-LNP, spike protein, preservative free, 30 mcg/0.3 mL dosage, tris-sucrose formulation; first dose
or	procedure	UMLS:CPT:91311	Severe acute respiratory syndrome coronavirus 2 (SARS-CoV-2) (coronavirus disease [COVID-19]) vaccine, mRNA-LNP, spike protein, preservative free, 25 mcg/0.25 mL dosage, for intramuscular use
or	procedure	UMLS:CPT:0074A	Immunization administration by intramuscular injection of severe acute respiratory syndrome coronavirus 2 (SARS-CoV-2) (coronavirus disease [COVID-19]) vaccine, mRNA-LNP, spike protein, preservative free, 10 mcg/0.2 mL dosage, diluent reconstituted, tris-sucrose formulation; booster dose
or	procedure	UMLS:CPT:0112A	Immunization administration by intramuscular injection of severe acute respiratory syndrome coronavirus 2 (SARS-CoV-2) (coronavirus disease

			[COVID-19]) vaccine, mRNA-LNP, spike protein, preservative free, 25 mcg/0.25 mL dosage; second dose
or	procedure	UMLS:CPT:0083A	Immunization administration by intramuscular injection of severe acute respiratory syndrome coronavirus 2 (SARS-CoV-2) (coronavirus disease [COVID-19]) vaccine, mRNA-LNP, spike protein, preservative free, 3 mcg/0.2 mL dosage, diluent reconstituted, tris-sucrose formulation; third dose
or	procedure	UMLS:CPT:0073A	Immunization administration by intramuscular injection of severe acute respiratory syndrome coronavirus 2 (SARS-CoV-2) (coronavirus disease [COVID-19]) vaccine, mRNA-LNP, spike protein, preservative free, 10 mcg/0.2 mL dosage, diluent reconstituted, tris-sucrose formulation; third dose
or	procedure	UMLS:CPT:0173A	Immunization administration by intramuscular injection of severe acute respiratory syndrome coronavirus 2 (SARS-CoV-2) (coronavirus disease [COVID-19]) vaccine, mRNA-LNP, bivalent spike protein, preservative free, 3 mcg/0.2 mL dosage, diluent reconstituted, tris-sucrose formulation, third dose
or	procedure	UMLS:CPT:0164A	Immunization administration by intramuscular injection of severe acute respiratory

			syndrome coronavirus 2 (SARS-CoV-2) (coronavirus disease [COVID-19]) vaccine, mRNA-LNP, spike protein, bivalent, preservative free, 10 mcg/0.2 mL dosage, booster dose
or	procedure	UMLS:CPT:1037838	Immunization administration by intramuscular injection of severe acute respiratory syndrome coronavirus 2 (SARS-CoV-2) (coronavirus disease [COVID-19]) vaccine, mRNA-LNP, spike protein, preservative free, 50 mcg/0.5 mL dosage
or	procedure	UMLS:CPT:0094A	Immunization administration by intramuscular injection of severe acute respiratory syndrome coronavirus 2 (SARS-CoV-2) (coronavirus disease [COVID-19]) vaccine, mRNA-LNP, spike protein, preservative free, 50 mcg/0.5 mL dosage; booster dose, when administered to individuals 18 years and over
or	procedure	UMLS:CPT:0034A	Immunization administration by intramuscular injection of severe acute respiratory syndrome coronavirus 2 (SARS-CoV-2) (coronavirus disease [COVID-19]) vaccine, DNA, spike protein, adenovirus type 26 (Ad26) vector, preservative free, 5x1010 viral particles/0.5 mL dosage; booster dose
or	procedure	UMLS:CPT:0144A	Immunization administration by intramuscular injection of severe acute respiratory

			syndrome coronavirus 2 (SARS-CoV-2) (coronavirus disease [COVID-19]) vaccine, mRNA-LNP, spike protein, bivalent, preservative free, 25 mcg/0.25 mL dosage, booster dose
or	procedure	UMLS:CPT:0091A	Immunization administration by intramuscular injection of severe acute respiratory syndrome coronavirus 2 (SARS-CoV-2) (coronavirus disease [COVID-19]) vaccine, mRNA-LNP, spike protein, preservative free, 50 mcg/0.5 mL dosage; first dose, when administered to individuals 6 through 11 years
or	procedure	UMLS:CPT:0174A	Immunization administration by intramuscular injection of severe acute respiratory syndrome coronavirus 2 (SARS-CoV-2) (coronavirus disease [COVID-19]) vaccine, mRNA-LNP, bivalent spike protein, preservative free, 3 mcg/0.2 mL dosage, diluent reconstituted, tris-sucrose formulation, booster
or	procedure	UMLS:CPT:0092A	Immunization administration by intramuscular injection of severe acute respiratory syndrome coronavirus 2 (SARS-CoV-2) (coronavirus disease [COVID-19]) vaccine, mRNA-LNP, spike protein, preservative free, 50 mcg/0.5 mL dosage; second dose, when administered to individuals 6 through 11 years

or	procedure	UMLS:CPT:1036682	Immunization administration by intramuscular injection of severe acute respiratory syndrome coronavirus 2 (SARS-CoV-2) (coronavirus disease [COVID-19]) vaccine, recombinant spike protein nanoparticle, saponin-based adjuvant, preservative free, 5 mcg/0.5 mL dosage
or	procedure	UMLS:CPT:0041A	Immunization administration by intramuscular injection of severe acute respiratory syndrome coronavirus 2 (SARS-CoV-2) (coronavirus disease [COVID-19]) vaccine, recombinant spike protein nanoparticle, saponin-based adjuvant, preservative free, 5 mcg/0.5 mL dosage; first dose
or	procedure	UMLS:CPT:0113A	Immunization administration by intramuscular injection of severe acute respiratory syndrome coronavirus 2 (SARS-CoV-2) (coronavirus disease [COVID-19]) vaccine, mRNA-LNP, spike protein, preservative free, 25 mcg/0.25 mL dosage; third dose
or	procedure	UMLS:CPT:0042A	Immunization administration by intramuscular injection of severe acute respiratory syndrome coronavirus 2 (SARS-CoV-2) (coronavirus disease [COVID-19]) vaccine, recombinant spike protein nanoparticle, saponin-based adjuvant, preservative free, 5

				mcg/0.5 mL dosage; second dose
	or	procedure	UMLS:CPT:0093A	Immunization administration by intramuscular injection of severe acute respiratory syndrome coronavirus 2 (SARS-CoV-2) (coronavirus disease [COVID-19]) vaccine, mRNA-LNP, spike protein, preservative free, 50 mcg/0.5 mL dosage; third dose, when administered to individuals 6 through 11 years
	or	procedure	UMLS:CPT:1036666	Immunization administration by intramuscular injection of severe acute respiratory syndrome coronavirus 2 (SARS-CoV-2) (coronavirus disease [COVID-19]) vaccine, DNA, spike protein, chimpanzee adenovirus Oxford 1 (ChAdOx1) vector, preservative free, 5x1010 viral particles/0.5 mL dosage
	or	procedure	UMLS:CPT:0044A	Immunization administration by intramuscular injection of severe acute respiratory syndrome coronavirus 2 (SARS-CoV-2) (coronavirus disease [COVID-19]) vaccine, recombinant spike protein nanoparticle, saponin-based adjuvant, preservative free, 5 mcg/0.5mL dosage; booster
date constraint		The terms in this group occurred between Dec 1, 2019 and Dec 31, 2023		
**Group 2**				
**Group 2A COVID-19 negative**				
must have		visit	TNX:Visit	Visit (Data Source: TriNetX)
cannot have		diagnosis	UMLS:ICD10CM:U07.1	COVID-19

	or	laboratory	UMLS:LNC:95406-5	SARS-CoV-2 (COVID-19) RNA [Presence] in Nose by NAA with probe detection (labResult: Positive)
	or	laboratory	UMLS:LNC:94845-5	SARS-CoV-2 (COVID-19) RNA [Presence] in Saliva (oral fluid) by NAA with probe detection (labResult: Positive)
	or	laboratory	UMLS:LNC:95608-6	SARS-CoV-2 (COVID-19) RNA [Presence] in Respiratory specimen by NAA with non-probe detection (labResult: Positive)
	or	laboratory	UMLS:LNC:94759-8	SARS-CoV-2 (COVID-19) RNA [Presence] in Nasopharynx by NAA with probe detection (labResult: Positive)
	or	laboratory	UMLS:LNC:94565-9	SARS-CoV-2 (COVID-19) RNA [Presence] in Nasopharynx by NAA with non-probe detection (labResult: Positive)
	or	laboratory	UMLS:LNC:94309-2	SARS-CoV-2 (COVID-19) RNA [Presence] in Specimen by NAA with probe detection (labResult: Positive)
	or	laboratory	UMLS:LNC:94500-6	SARS-CoV-2 (COVID-19) RNA [Presence] in Respiratory specimen by NAA with probe detection (labResult: Positive)
	or	diagnosis	UMLS:ICD10CM:U07.2	COVID-19, virus not identified (WHO)
	or	diagnosis	UMLS:ICD10CM:J12.82	Pneumonia due to coronavirus disease 2019
date constraint	The terms in this group occurred at any time			
event relationship	Any instance of thyroid cancer history occurred at least 1 day before the first instance of COVID-19 negative			
**Group 2B thyroid cancer history**				

cannot have		diagnosis	UMLS:ICD10CM:C73	Malignant neoplasm of thyroid gland
	or	diagnosis	UMLS:ICD10CM:Z85.850	Personal history of malignant neoplasm of thyroid

Query Criteria for Cohort post-COVID, >60

This query was run on the network Global Collaborative Network with 148 HCO(s) queried and 148 HCO(s) responded. A total of 122 provider(s) responded with patients. The final cohort included 897,532 patients who matched the query criteria listed in the table below.

**Ungrouped terms**				
must have		demographics	Age	Age (at least 61 years (most recent occurrence))
	and any of	demographics	UMLS:HL7V3.0:Gender:M	Male
		demographics	UMLS:HL7V3.0:Gender:F	Female
**Group 1**				
**COVID-19, no vaccination**				
must have	any of	diagnosis	UMLS:ICD10CM:U07.1	COVID-19
		diagnosis	UMLS:ICD10CM:U07.2	COVID-19, virus not identified (WHO)
		diagnosis	UMLS:ICD10CM:J12.82	Pneumonia due to coronavirus disease 2019
		laboratory	UMLS:LNC:94500-6	SARS-CoV-2 (COVID-19) RNA [Presence] in Respiratory specimen by NAA with probe detection (labResult: Positive)
		laboratory	UMLS:LNC:94309-2	SARS-CoV-2 (COVID-19) RNA [Presence] in Specimen by NAA with probe detection (labResult: Positive)
		laboratory	UMLS:LNC:94565-9	SARS-CoV-2 (COVID-19) RNA [Presence] in Nasopharynx by NAA with non-probe detection (labResult: Positive)
		laboratory	UMLS:LNC:94759-8	SARS-CoV-2 (COVID-19) RNA [Presence] in Nasopharynx by

				NAA with probe detection (labResult: Positive)
		laboratory	UMLS:LNC:95608-6	SARS-CoV-2 (COVID-19) RNA [Presence] in Respiratory specimen by NAA with non-probe detection (labResult: Positive)
		laboratory	UMLS:LNC:94845-5	SARS-CoV-2 (COVID-19) RNA [Presence] in Saliva (oral fluid) by NAA with probe detection (labResult: Positive)
		laboratory	UMLS:LNC:95406-5	SARS-CoV-2 (COVID-19) RNA [Presence] in Nose by NAA with probe detection (labResult: Positive)
cannot have		medication	NLM:CVX:208	COVID-19, mRNA, LNP-S, PF, 30 mcg/0.3 mL dose
	or	medication	NLM:CVX:207	COVID-19, mRNA, LNP-S, PF, 100 mcg/0.5mL dose or 50 mcg/0.25mL dose
	or	medication	NLM:CVX:212	COVID-19 vaccine, vector-nr, rS-Ad26, PF, 0.5 mL
	or	medication	NLM:RXNORM:OMOP5042939	COVID-19 vaccine
	or	medication	NLM:CVX:300	COVID-19, mRNA, LNP-S, bivalent, PF, 30 mcg/0.3 mL dose
	or	medication	NLM:CVX:217	COVID-19, mRNA, LNP-S, PF, 30 mcg/0.3 mL dose, tris-sucrose
	or	medication	NLM:CVX:229	COVID-19, mRNA, LNP-S, bivalent, PF, 50 mcg/0.5 mL or 25mcg/0.25 mL dose
	or	medication	NLM:CVX:218	COVID-19, mRNA, LNP-S, PF, 10 mcg/0.2 mL dose, tris-sucrose
	or	medication	NLM:CVX:520	COVID-19 mRNA, bivalent, original/Omicron BA.1, Non-US Vaccine Product, Pfizer-BioNTech

or	medication	NLM:CVX:519	COVID-19 mRNA, bivalent, original/Omicron BA.1, Non-US Vaccine (Spikevax Bivalent), Moderna
or	medication	NLM:CVX:301	COVID-19, mRNA, LNP-S, bivalent, PF, 10 mcg/0.2 mL dose
or	medication	NLM:CVX:219	COVID-19, mRNA, LNP-S, PF, 3 mcg/0.2 mL dose, tris-sucrose
or	medication	NLM:CVX:228	COVID-19, mRNA, LNP-S, PF, pediatric 25 mcg/0.25 mL dose
or	medication	NLM:CVX:230	COVID-19, mRNA, LNP-S, bivalent booster, PF, 10 mcg/0.2 mL
or	medication	NLM:CVX:221	COVID-19, mRNA, LNP-S, PF, 50 mcg/0.5 mL dose
or	medication	NLM:CVX:210	COVID-19 vaccine, vector-nr, rS-ChAdOx1, PF, 0.5 mL
or	medication	NLM:CVX:302	COVID-19, mRNA, LNP-S, bivalent, PF, 3 mcg/0.2 mL dose
or	medication	NLM:CVX:511	COVID-19 IV Non-US Vaccine (CoronaVac, Sinovac)
or	medication	NLM:RXNORM:2468231	SARS-CoV-2 (COVID-19) vaccine, mRNA spike protein
or	procedure	UMLS:CPT:91300	Severe acute respiratory syndrome coronavirus 2 (SARS-CoV-2) (coronavirus disease [COVID-19]) vaccine, mRNA-LNP, spike protein, preservative free, 30 mcg/0.3 mL dosage, diluent reconstituted, for intramuscular use
or	procedure	UMLS:CPT:0001A	Immunization administration by intramuscular injection of severe acute respiratory syndrome coronavirus 2 (SARS-CoV-2) (coronavirus disease [COVID-19]) vaccine, mRNA-

			LNP, spike protein, preservative free, 30 mcg/0.3 mL dosage, diluent reconstituted; first dose
or	procedure	UMLS:CPT:0002A	Immunization administration by intramuscular injection of severe acute respiratory syndrome coronavirus 2 (SARS-CoV-2) (coronavirus disease [COVID-19]) vaccine, mRNA-LNP, spike protein, preservative free, 30 mcg/0.3 mL dosage, diluent reconstituted; second dose
or	procedure	UMLS:CPT:91301	Severe acute respiratory syndrome coronavirus 2 (SARS-CoV-2) (coronavirus disease [COVID-19]) vaccine, mRNA-LNP, spike protein, preservative free, 100 mcg/0.5 mL dosage, for intramuscular use
or	procedure	UMLS:CPT:0011A	Immunization administration by intramuscular injection of severe acute respiratory syndrome coronavirus 2 (SARS-CoV-2) (coronavirus disease [COVID-19]) vaccine, mRNA-LNP, spike protein, preservative free, 100 mcg/0.5 mL dosage; first dose
or	procedure	UMLS:CPT:0012A	Immunization administration by intramuscular injection of severe acute respiratory syndrome coronavirus 2 (SARS-CoV-2) (coronavirus disease [COVID-19]) vaccine, mRNA-LNP, spike protein, preservative free, 100 mcg/0.5 mL dosage; second dose

or	procedure	UMLS:SNOMED:840534001	Administration of SARS-CoV-2 antigen vaccine
or	medication	NLM:CVX:213	SARS-CoV-2 (COVID-19) Vaccine
or	procedure	UMLS:CPT:1036660	Immunization administration by intramuscular injection of severe acute respiratory syndrome coronavirus 2 (SARS-CoV-2) (coronavirus disease [COVID-19]) vaccine, mRNA-LNP, spike protein, preservative free, 30 mcg/0.3 mL dosage, diluent reconstituted
or	procedure	UMLS:CPT:1036663	Immunization administration by intramuscular injection of severe acute respiratory syndrome coronavirus 2 (SARS-CoV-2) (coronavirus disease [COVID-19]) vaccine, mRNA-LNP, spike protein, preservative free, 100 mcg/0.5 mL dosage
or	procedure	UMLS:CPT:0124A	Immunization administration by intramuscular injection of severe acute respiratory syndrome coronavirus 2 (SARS-CoV-2) (coronavirus disease [COVID-19]) vaccine, mRNA-LNP, bivalent spike protein, preservative free, 30 mcg/0.3 mL dosage, tris-sucrose formulation, booster dose
or	procedure	UMLS:CPT:0004A	Immunization administration by intramuscular injection of severe acute respiratory syndrome coronavirus 2 (SARS-CoV-2) (coronavirus disease [COVID-19]) vaccine, mRNA-LNP, spike protein, preservative

			free, 30 mcg/0.3 mL dosage, diluent reconstituted; booster dose
or	procedure	UMLS:CPT:0003A	Immunization administration by intramuscular injection of severe acute respiratory syndrome coronavirus 2 (SARS-CoV-2) (coronavirus disease [COVID-19]) vaccine, mRNA-LNP, spike protein, preservative free, 30 mcg/0.3 mL dosage, diluent reconstituted; third dose
or	procedure	UMLS:CPT:1037166	Immunization administration by intramuscular injection of severe acute respiratory syndrome coronavirus 2 (SARS-CoV-2) (coronavirus disease [COVID-19]) vaccine, mRNA-LNP, spike protein, preservative free, 30 mcg/0.3 mL dosage, tris-sucrose formulation
or	procedure	UMLS:CPT:0054A	Immunization administration by intramuscular injection of severe acute respiratory syndrome coronavirus 2 (SARS-CoV-2) (coronavirus disease [COVID-19]) vaccine, mRNA-LNP, spike protein, preservative free, 30 mcg/0.3 mL dosage, tris-sucrose formulation; booster dose
or	procedure	UMLS:CPT:0064A	Immunization administration by intramuscular injection of severe acute respiratory syndrome coronavirus 2 (SARS-CoV-2) (coronavirus disease [COVID-19]) vaccine, mRNA-LNP, spike protein, preservative

			free, 50 mcg/0.25 mL dosage, booster dose
or	procedure	UMLS:CPT:90480	Immunization administration by intramuscular injection of severe acute respiratory syndrome coronavirus 2 (SARS-CoV-2) (coronavirus disease [COVID-19]) vaccine, single dose
or	procedure	UMLS:CPT:1037171	Immunization administration by intramuscular injection of severe acute respiratory syndrome coronavirus 2 (SARS-CoV-2) (coronavirus disease [COVID-19]) vaccine, mRNA-LNP, spike protein, preservative free, 10 mcg/0.2 mL dosage, diluent reconstituted, tris-sucrose formulation
or	procedure	UMLS:CPT:0071A	Immunization administration by intramuscular injection of severe acute respiratory syndrome coronavirus 2 (SARS-CoV-2) (coronavirus disease [COVID-19]) vaccine, mRNA-LNP, spike protein, preservative free, 10 mcg/0.2 mL dosage, diluent reconstituted, tris-sucrose formulation; first dose
or	procedure	UMLS:CPT:0072A	Immunization administration by intramuscular injection of severe acute respiratory syndrome coronavirus 2 (SARS-CoV-2) (coronavirus disease [COVID-19]) vaccine, mRNA-LNP, spike protein, preservative free, 10 mcg/0.2 mL dosage, diluent reconstituted, tris-

			sucrose formulation; second dose
or	medication	NLM:RXNORM:2610319	SARS-CoV-2 (COVID-19) vaccine, mRNA-BNT162b2 0.05 MG/ML / SARS-CoV-2 (COVID-19) vaccine, mRNA-BNT162b2 OMICRON (BA.4/BA.5) 0.05 MG/ML Injectable Suspension
or	procedure	UMLS:CPT:91313	Severe acute respiratory syndrome coronavirus 2 (SARS-CoV-2) (coronavirus disease [COVID-19]) vaccine, mRNA-LNP, spike protein, bivalent, preservative free, 50 mcg/0.5 mL dosage, for intramuscular use
or	procedure	UMLS:CPT:0134A	Immunization administration by intramuscular injection of severe acute respiratory syndrome coronavirus 2 (SARS-CoV-2) (coronavirus disease [COVID-19]) vaccine, mRNA-LNP, spike protein, bivalent, preservative free, 50 mcg/0.5 mL dosage, booster dose
or	procedure	UMLS:CPT:1037175	Immunization administration by intramuscular injection of severe acute respiratory syndrome coronavirus 2 (SARS-CoV-2) (coronavirus disease [COVID-19]) vaccine, DNA, spike protein, adenovirus type 26 (Ad26) vector, preservative free, 5x1010 viral particles/0.5 mL dosage
or	medication	NLM:RXNORM:2610347	0.3 ML SARS-CoV-2 (COVID-19) vaccine, mRNA-BNT162b2 0.05 MG/ML / SARS-CoV-2 (COVID-19) vaccine, mRNA-BNT162b2

			OMICRON (BA.4/BA.5) -1 MG/ML Injection
or	procedure	UMLS:CPT:1037228	Immunization administration by intramuscular injection of severe acute respiratory syndrome coronavirus 2 (SARS-CoV-2) (coronavirus disease [COVID-19]) vaccine, mRNA-LNP, spike protein, preservative free, 3 mcg/0.2 mL dosage, diluent reconstituted, tris-sucrose formulation
or	procedure	UMLS:CPT:0013A	Immunization administration by intramuscular injection of severe acute respiratory syndrome coronavirus 2 (SARS-CoV-2) (coronavirus disease [COVID-19]) vaccine, mRNA-LNP, spike protein, preservative free, 100 mcg/0.5 mL dosage; third dose
or	procedure	UMLS:CPT:0081A	Immunization administration by intramuscular injection of severe acute respiratory syndrome coronavirus 2 (SARS-CoV-2) (coronavirus disease [COVID-19]) vaccine, mRNA-LNP, spike protein, preservative free, 3 mcg/0.2 mL dosage, diluent reconstituted, tris-sucrose formulation; first dose
or	procedure	UMLS:CPT:0082A	Immunization administration by intramuscular injection of severe acute respiratory syndrome coronavirus 2 (SARS-CoV-2) (coronavirus disease [COVID-19]) vaccine, mRNA-LNP, spike protein, preservative

			free, 3 mcg/0.2 mL dosage, diluent reconstituted, tris-sucrose formulation; second dose
or	medication	NLM:RXNORM:2610328	SARS-CoV-2 (COVID-19) vaccine, mRNA-1273 0.05 MG/ML / SARS-CoV-2 (COVID-19) vaccine, mRNA-1273 OMICRON (BA.4/BA.5) 0.05 MG/ML Injectable Suspension
or	procedure	UMLS:CPT:0154A	Immunization administration by intramuscular injection of severe acute respiratory syndrome coronavirus 2 (SARS-CoV-2) (coronavirus disease [COVID-19]) vaccine, mRNA-LNP, bivalent spike protein, preservative free, 10 mcg/0.2 mL dosage, diluent reconstituted, tris-sucrose formulation, booster dose
or	procedure	UMLS:CPT:0053A	Immunization administration by intramuscular injection of severe acute respiratory syndrome coronavirus 2 (SARS-CoV-2) (coronavirus disease [COVID-19]) vaccine, mRNA-LNP, spike protein, preservative free, 30 mcg/0.3 mL dosage, tris-sucrose formulation; third dose
or	procedure	UMLS:CPT:1037332	Immunization administration by intramuscular injection of severe acute respiratory syndrome coronavirus 2 (SARS-CoV-2) (coronavirus disease [COVID-19]) vaccine, mRNA-

			LNP, spike protein, preservative free, 25 mcg/0.25 mL dosage
or	procedure	UMLS:CPT:0052A	Immunization administration by intramuscular injection of severe acute respiratory syndrome coronavirus 2 (SARS-CoV-2) (coronavirus disease [COVID-19]) vaccine, mRNA-LNP, spike protein, preservative free, 30 mcg/0.3 mL dosage, tris-sucrose formulation; second dose
or	procedure	UMLS:CPT:0111A	Immunization administration by intramuscular injection of severe acute respiratory syndrome coronavirus 2 (SARS-CoV-2) (coronavirus disease [COVID-19]) vaccine, mRNA-LNP, spike protein, preservative free, 25 mcg/0.25 mL dosage; first dose
or	procedure	UMLS:CPT:0051A	Immunization administration by intramuscular injection of severe acute respiratory syndrome coronavirus 2 (SARS-CoV-2) (coronavirus disease [COVID-19]) vaccine, mRNA-LNP, spike protein, preservative free, 30 mcg/0.3 mL dosage, tris-sucrose formulation; first dose
or	procedure	UMLS:CPT:91311	Severe acute respiratory syndrome coronavirus 2 (SARS-CoV-2) (coronavirus disease [COVID-19]) vaccine, mRNA-LNP, spike protein, preservative free, 25 mcg/0.25 mL dosage, for intramuscular use

or	procedure	UMLS:CPT:0074A	Immunization administration by intramuscular injection of severe acute respiratory syndrome coronavirus 2 (SARS-CoV-2) (coronavirus disease [COVID-19]) vaccine, mRNA-LNP, spike protein, preservative free, 10 mcg/0.2 mL dosage, diluent reconstituted, tris-sucrose formulation; booster dose
or	procedure	UMLS:CPT:0112A	Immunization administration by intramuscular injection of severe acute respiratory syndrome coronavirus 2 (SARS-CoV-2) (coronavirus disease [COVID-19]) vaccine, mRNA-LNP, spike protein, preservative free, 25 mcg/0.25 mL dosage; second dose
or	procedure	UMLS:CPT:0083A	Immunization administration by intramuscular injection of severe acute respiratory syndrome coronavirus 2 (SARS-CoV-2) (coronavirus disease [COVID-19]) vaccine, mRNA-LNP, spike protein, preservative free, 3 mcg/0.2 mL dosage, diluent reconstituted, tris-sucrose formulation; third dose
or	procedure	UMLS:CPT:0073A	Immunization administration by intramuscular injection of severe acute respiratory syndrome coronavirus 2 (SARS-CoV-2) (coronavirus disease [COVID-19]) vaccine, mRNA-LNP, spike protein, preservative free, 10 mcg/0.2 mL dosage,

			diluent reconstituted, tris-sucrose formulation; third dose
or	procedure	UMLS:CPT:0173A	Immunization administration by intramuscular injection of severe acute respiratory syndrome coronavirus 2 (SARS-CoV-2) (coronavirus disease [COVID-19]) vaccine, mRNA-LNP, bivalent spike protein, preservative free, 3 mcg/0.2 mL dosage, diluent reconstituted, tris-sucrose formulation, third dose
or	procedure	UMLS:CPT:0164A	Immunization administration by intramuscular injection of severe acute respiratory syndrome coronavirus 2 (SARS-CoV-2) (coronavirus disease [COVID-19]) vaccine, mRNA-LNP, spike protein, bivalent, preservative free, 10 mcg/0.2 mL dosage, booster dose
or	procedure	UMLS:CPT:1037838	Immunization administration by intramuscular injection of severe acute respiratory syndrome coronavirus 2 (SARS-CoV-2) (coronavirus disease [COVID-19]) vaccine, mRNA-LNP, spike protein, preservative free, 50 mcg/0.5 mL dosage
or	procedure	UMLS:CPT:0094A	Immunization administration by intramuscular injection of severe acute respiratory syndrome coronavirus 2 (SARS-CoV-2) (coronavirus disease [COVID-19]) vaccine, mRNA-LNP, spike protein, preservative free, 50 mcg/0.5 mL dosage;

			booster dose, when administered to individuals 18 years and over
or	procedure	UMLS:CPT:0034A	Immunization administration by intramuscular injection of severe acute respiratory syndrome coronavirus 2 (SARS-CoV-2) (coronavirus disease [COVID-19]) vaccine, DNA, spike protein, adenovirus type 26 (Ad26) vector, preservative free, 5x1010 viral particles/0.5 mL dosage; booster dose
or	procedure	UMLS:CPT:0144A	Immunization administration by intramuscular injection of severe acute respiratory syndrome coronavirus 2 (SARS-CoV-2) (coronavirus disease [COVID-19]) vaccine, mRNA-LNP, spike protein, bivalent, preservative free, 25 mcg/0.25 mL dosage, booster dose
or	procedure	UMLS:CPT:0091A	Immunization administration by intramuscular injection of severe acute respiratory syndrome coronavirus 2 (SARS-CoV-2) (coronavirus disease [COVID-19]) vaccine, mRNA-LNP, spike protein, preservative free, 50 mcg/0.5 mL dosage; first dose, when administered to individuals 6 through 11 years
or	procedure	UMLS:CPT:0174A	Immunization administration by intramuscular injection of severe acute respiratory syndrome coronavirus 2 (SARS-CoV-2) (coronavirus disease [COVID-19]) vaccine, mRNA-

			LNP, bivalent spike protein, preservative free, 3 mcg/0.2 mL dosage, diluent reconstituted, tris-sucrose formulation, booster
or	procedure	UMLS:CPT:0092A	Immunization administration by intramuscular injection of severe acute respiratory syndrome coronavirus 2 (SARS-CoV-2) (coronavirus disease [COVID-19]) vaccine, mRNA-LNP, spike protein, preservative free, 50 mcg/0.5 mL dosage; second dose, when administered to individuals 6 through 11 years
or	procedure	UMLS:CPT:1036682	Immunization administration by intramuscular injection of severe acute respiratory syndrome coronavirus 2 (SARS-CoV-2) (coronavirus disease [COVID-19]) vaccine, recombinant spike protein nanoparticle, saponin-based adjuvant, preservative free, 5 mcg/0.5 mL dosage
or	procedure	UMLS:CPT:0041A	Immunization administration by intramuscular injection of severe acute respiratory syndrome coronavirus 2 (SARS-CoV-2) (coronavirus disease [COVID-19]) vaccine, recombinant spike protein nanoparticle, saponin-based adjuvant, preservative free, 5 mcg/0.5 mL dosage; first dose
or	procedure	UMLS:CPT:0113A	Immunization administration by intramuscular injection of

			severe acute respiratory syndrome coronavirus 2 (SARS-CoV-2) (coronavirus disease [COVID-19]) vaccine, mRNA-LNP, spike protein, preservative free, 25 mcg/0.25 mL dosage; third dose
or	procedure	UMLS:CPT:0042A	Immunization administration by intramuscular injection of severe acute respiratory syndrome coronavirus 2 (SARS-CoV-2) (coronavirus disease [COVID-19]) vaccine, recombinant spike protein nanoparticle, saponin-based adjuvant, preservative free, 5 mcg/0.5 mL dosage; second dose
or	procedure	UMLS:CPT:0093A	Immunization administration by intramuscular injection of severe acute respiratory syndrome coronavirus 2 (SARS-CoV-2) (coronavirus disease [COVID-19]) vaccine, mRNA-LNP, spike protein, preservative free, 50 mcg/0.5 mL dosage; third dose, when administered to individuals 6 through 11 years
or	procedure	UMLS:CPT:1036666	Immunization administration by intramuscular injection of severe acute respiratory syndrome coronavirus 2 (SARS-CoV-2) (coronavirus disease [COVID-19]) vaccine, DNA, spike protein, chimpanzee adenovirus Oxford 1 (ChAdOx1) vector, preservative free, 5x1010 viral particles/0.5 mL dosage

	or	procedure	UMLS:CPT:0044A	Immunization administration by intramuscular injection of severe acute respiratory syndrome coronavirus 2 (SARS-CoV-2) (coronavirus disease [COVID-19]) vaccine, recombinant spike protein nanoparticle, saponin-based adjuvant, preservative free, 5 mcg/0.5mL dosage; booster
date constraint		The terms in this group occurred between Dec 1, 2019 and Dec 31, 2023		
**Group 2**				
**Group 2A COVID-19**				
must have	any of	diagnosis	UMLS:ICD10CM:U07.1	COVID-19
		diagnosis	UMLS:ICD10CM:U07.2	COVID-19, virus not identified (WHO)
		diagnosis	UMLS:ICD10CM:J12.82	Pneumonia due to coronavirus disease 2019
		laboratory	UMLS:LNC:94500-6	SARS-CoV-2 (COVID-19) RNA [Presence] in Respiratory specimen by NAA with probe detection (labResult: Positive)
		laboratory	UMLS:LNC:94309-2	SARS-CoV-2 (COVID-19) RNA [Presence] in Specimen by NAA with probe detection (labResult: Positive)
		laboratory	UMLS:LNC:94565-9	SARS-CoV-2 (COVID-19) RNA [Presence] in Nasopharynx by NAA with non-probe detection (labResult: Positive)
		laboratory	UMLS:LNC:94759-8	SARS-CoV-2 (COVID-19) RNA [Presence] in Nasopharynx by NAA with probe detection (labResult: Positive)
		laboratory	UMLS:LNC:95608-6	SARS-CoV-2 (COVID-19) RNA [Presence] in Respiratory specimen by NAA with non-

				probe detection (labResult: Positive)
		laboratory	UMLS:LNC:94845-5	SARS-CoV-2 (COVID-19) RNA [Presence] in Saliva (oral fluid) by NAA with probe detection (labResult: Positive)
		laboratory	UMLS:LNC:95406-5	SARS-CoV-2 (COVID-19) RNA [Presence] in Nose by NAA with probe detection (labResult: Positive)
	and	visit	TNX:Visit	Visit (Data Source: TriNetX)
date constraint		The terms in this group occurred at any time		
event relationship		Any instance of thyroid cancer history occurred at least 1 day before the first instance of COVID-19		
**Group 2B thyroid cancer history**				
cannot have		diagnosis	UMLS:ICD10CM:C73	Malignant neoplasm of thyroid gland
	or	diagnosis	UMLS:ICD10CM:Z85.850	Personal history of malignant neoplasm of thyroid

Query Criteria for Cohort non-COVID, >60

This query was run on the network Global Collaborative Network with 148 HCO(s) queried and 148 HCO(s) responded. A total of 131 provider(s) responded with patients. The final cohort included 12,129,504 patients who matched the query criteria listed in the table below.

**Ungrouped terms**				
must have		demographics	Age	Age (at least 61 years (most recent occurrence))
	and any of	demographics	UMLS:HL7V3.0:Gender:M	Male
		demographics	UMLS:HL7V3.0:Gender:F	Female
**Group 1**				
**COVID-19 negative, no vaccination**				
must have		visit	TNX:Visit	Visit
cannot have		medication	NLM:CVX:208	COVID-19, mRNA, LNP-S, PF, 30 mcg/0.3 mL dose

or	medication	NLM:CVX:207	COVID-19, mRNA, LNP-S, PF, 100 mcg/0.5mL dose or 50 mcg/0.25mL dose
or	medication	NLM:CVX:212	COVID-19 vaccine, vector-nr, rS-Ad26, PF, 0.5 mL
or	medication	NLM:RXNORM:OMOP 5042939	COVID-19 vaccine
or	medication	NLM:CVX:300	COVID-19, mRNA, LNP-S, bivalent, PF, 30 mcg/0.3 mL dose
or	medication	NLM:CVX:217	COVID-19, mRNA, LNP-S, PF, 30 mcg/0.3 mL dose, tris-sucrose
or	medication	NLM:CVX:229	COVID-19, mRNA, LNP-S, bivalent, PF, 50 mcg/0.5 mL or 25mcg/0.25 mL dose
or	medication	NLM:CVX:218	COVID-19, mRNA, LNP-S, PF, 10 mcg/0.2 mL dose, tris-sucrose
or	medication	NLM:CVX:520	COVID-19 mRNA, bivalent, original/Omicron BA.1, Non-US Vaccine Product, Pfizer-BioNTech
or	medication	NLM:CVX:519	COVID-19 mRNA, bivalent, original/Omicron BA.1, Non-US Vaccine (Spikevax Bivalent), Moderna
or	medication	NLM:CVX:301	COVID-19, mRNA, LNP-S, bivalent, PF, 10 mcg/0.2 mL dose
or	medication	NLM:CVX:219	COVID-19, mRNA, LNP-S, PF, 3 mcg/0.2 mL dose, tris-sucrose
or	medication	NLM:CVX:228	COVID-19, mRNA, LNP-S, PF, pediatric 25 mcg/0.25 mL dose
or	medication	NLM:CVX:230	COVID-19, mRNA, LNP-S, bivalent booster, PF, 10 mcg/0.2 mL
or	medication	NLM:CVX:221	COVID-19, mRNA, LNP-S, PF, 50 mcg/0.5 mL dose

or	medication	NLM:CVX:210	COVID-19 vaccine, vector-nr, rS-ChAdOx1, PF, 0.5 mL
or	medication	NLM:CVX:302	COVID-19, mRNA, LNP-S, bivalent, PF, 3 mcg/0.2 mL dose
or	medication	NLM:CVX:511	COVID-19 IV Non-US Vaccine (CoronaVac, Sinovac)
or	medication	NLM:RXNORM:24682 31	SARS-CoV-2 (COVID-19) vaccine, mRNA spike protein
or	procedure	UMLS:CPT:91300	Severe acute respiratory syndrome coronavirus 2 (SARS-CoV-2) (coronavirus disease [COVID-19]) vaccine, mRNA-LNP, spike protein, preservative free, 30 mcg/0.3 mL dosage, diluent reconstituted, for intramuscular use
or	procedure	UMLS:CPT:0001A	Immunization administration by intramuscular injection of severe acute respiratory syndrome coronavirus 2 (SARS-CoV-2) (coronavirus disease [COVID-19]) vaccine, mRNA-LNP, spike protein, preservative free, 30 mcg/0.3 mL dosage, diluent reconstituted; first dose
or	procedure	UMLS:CPT:0002A	Immunization administration by intramuscular injection of severe acute respiratory syndrome coronavirus 2 (SARS-CoV-2) (coronavirus disease [COVID-19]) vaccine, mRNA-LNP, spike protein, preservative free, 30 mcg/0.3 mL dosage, diluent reconstituted; second dose
or	procedure	UMLS:CPT:91301	Severe acute respiratory syndrome coronavirus 2 (SARS-CoV-2) (coronavirus disease

			[COVID-19]) vaccine, mRNA-LNP, spike protein, preservative free, 100 mcg/0.5 mL dosage, for intramuscular use
or	procedure	UMLS:CPT:0011A	Immunization administration by intramuscular injection of severe acute respiratory syndrome coronavirus 2 (SARS-CoV-2) (coronavirus disease [COVID-19]) vaccine, mRNA-LNP, spike protein, preservative free, 100 mcg/0.5 mL dosage; first dose
or	procedure	UMLS:CPT:0012A	Immunization administration by intramuscular injection of severe acute respiratory syndrome coronavirus 2 (SARS-CoV-2) (coronavirus disease [COVID-19]) vaccine, mRNA-LNP, spike protein, preservative free, 100 mcg/0.5 mL dosage; second dose
or	procedure	UMLS:SNOMED:840534001	Administration of SARS-CoV-2 antigen vaccine
or	medication	NLM:CVX:213	SARS-CoV-2 (COVID-19) Vaccine
or	procedure	UMLS:CPT:1036660	Immunization administration by intramuscular injection of severe acute respiratory syndrome coronavirus 2 (SARS-CoV-2) (coronavirus disease [COVID-19]) vaccine, mRNA-LNP, spike protein, preservative free, 30 mcg/0.3 mL dosage, diluent reconstituted
or	procedure	UMLS:CPT:1036663	Immunization administration by intramuscular injection of severe acute respiratory

			syndrome coronavirus 2 (SARS-CoV-2) (coronavirus disease [COVID-19]) vaccine, mRNA-LNP, spike protein, preservative free, 100 mcg/0.5 mL dosage
or	procedure	UMLS:CPT:0124A	Immunization administration by intramuscular injection of severe acute respiratory syndrome coronavirus 2 (SARS-CoV-2) (coronavirus disease [COVID-19]) vaccine, mRNA-LNP, bivalent spike protein, preservative free, 30 mcg/0.3 mL dosage, tris-sucrose formulation, booster dose
or	procedure	UMLS:CPT:0004A	Immunization administration by intramuscular injection of severe acute respiratory syndrome coronavirus 2 (SARS-CoV-2) (coronavirus disease [COVID-19]) vaccine, mRNA-LNP, spike protein, preservative free, 30 mcg/0.3 mL dosage, diluent reconstituted; booster dose
or	procedure	UMLS:CPT:0003A	Immunization administration by intramuscular injection of severe acute respiratory syndrome coronavirus 2 (SARS-CoV-2) (coronavirus disease [COVID-19]) vaccine, mRNA-LNP, spike protein, preservative free, 30 mcg/0.3 mL dosage, diluent reconstituted; third dose
or	procedure	UMLS:CPT:1037166	Immunization administration by intramuscular injection of severe acute respiratory syndrome coronavirus 2 (SARS-

			CoV-2) (coronavirus disease [COVID-19]) vaccine, mRNA-LNP, spike protein, preservative free, 30 mcg/0.3 mL dosage, tris-sucrose formulation
or	procedure	UMLS:CPT:0054A	Immunization administration by intramuscular injection of severe acute respiratory syndrome coronavirus 2 (SARS-CoV-2) (coronavirus disease [COVID-19]) vaccine, mRNA-LNP, spike protein, preservative free, 30 mcg/0.3 mL dosage, tris-sucrose formulation; booster dose
or	procedure	UMLS:CPT:0064A	Immunization administration by intramuscular injection of severe acute respiratory syndrome coronavirus 2 (SARS-CoV-2) (coronavirus disease [COVID-19]) vaccine, mRNA-LNP, spike protein, preservative free, 50 mcg/0.25 mL dosage, booster dose
or	procedure	UMLS:CPT:90480	Immunization administration by intramuscular injection of severe acute respiratory syndrome coronavirus 2 (SARS-CoV-2) (coronavirus disease [COVID-19]) vaccine, single dose
or	procedure	UMLS:CPT:1037171	Immunization administration by intramuscular injection of severe acute respiratory syndrome coronavirus 2 (SARS-CoV-2) (coronavirus disease [COVID-19]) vaccine, mRNA-LNP, spike protein, preservative

			free, 10 mcg/0.2 mL dosage, diluent reconstituted, tris-sucrose formulation
or	procedure	UMLS:CPT:0071A	Immunization administration by intramuscular injection of severe acute respiratory syndrome coronavirus 2 (SARS-CoV-2) (coronavirus disease [COVID-19]) vaccine, mRNA-LNP, spike protein, preservative free, 10 mcg/0.2 mL dosage, diluent reconstituted, tris-sucrose formulation; first dose
or	procedure	UMLS:CPT:0072A	Immunization administration by intramuscular injection of severe acute respiratory syndrome coronavirus 2 (SARS-CoV-2) (coronavirus disease [COVID-19]) vaccine, mRNA-LNP, spike protein, preservative free, 10 mcg/0.2 mL dosage, diluent reconstituted, tris-sucrose formulation; second dose
or	medication	NLM:RXNORM:2610319	SARS-CoV-2 (COVID-19) vaccine, mRNA-BNT162b2 0.05 MG/ML / SARS-CoV-2 (COVID-19) vaccine, mRNA-BNT162b2 OMICRON (BA.4/BA.5) 0.05 MG/ML Injectable Suspension
or	procedure	UMLS:CPT:91313	Severe acute respiratory syndrome coronavirus 2 (SARS-CoV-2) (coronavirus disease [COVID-19]) vaccine, mRNA-LNP, spike protein, bivalent, preservative free, 50 mcg/0.5 mL dosage, for intramuscular use

or	procedure	UMLS:CPT:0134A	Immunization administration by intramuscular injection of severe acute respiratory syndrome coronavirus 2 (SARS-CoV-2) (coronavirus disease [COVID-19]) vaccine, mRNA-LNP, spike protein, bivalent, preservative free, 50 mcg/0.5 mL dosage, booster dose
or	procedure	UMLS:CPT:1037175	Immunization administration by intramuscular injection of severe acute respiratory syndrome coronavirus 2 (SARS-CoV-2) (coronavirus disease [COVID-19]) vaccine, DNA, spike protein, adenovirus type 26 (Ad26) vector, preservative free, 5x1010 viral particles/0.5 mL dosage
or	medication	NLM:RXNORM:2610347	0.3 ML SARS-CoV-2 (COVID-19) vaccine, mRNA-BNT162b2 0.05 MG/ML / SARS-CoV-2 (COVID-19) vaccine, mRNA-BNT162b2 OMICRON (BA.4/BA.5) -1 MG/ML Injection
or	procedure	UMLS:CPT:1037228	Immunization administration by intramuscular injection of severe acute respiratory syndrome coronavirus 2 (SARS-CoV-2) (coronavirus disease [COVID-19]) vaccine, mRNA-LNP, spike protein, preservative free, 3 mcg/0.2 mL dosage, diluent reconstituted, tris-sucrose formulation
or	procedure	UMLS:CPT:0013A	Immunization administration by intramuscular injection of severe acute respiratory

			syndrome coronavirus 2 (SARS-CoV-2) (coronavirus disease [COVID-19]) vaccine, mRNA-LNP, spike protein, preservative free, 100 mcg/0.5 mL dosage; third dose
or	procedure	UMLS:CPT:0081A	Immunization administration by intramuscular injection of severe acute respiratory syndrome coronavirus 2 (SARS-CoV-2) (coronavirus disease [COVID-19]) vaccine, mRNA-LNP, spike protein, preservative free, 3 mcg/0.2 mL dosage, diluent reconstituted, tris-sucrose formulation; first dose
or	procedure	UMLS:CPT:0082A	Immunization administration by intramuscular injection of severe acute respiratory syndrome coronavirus 2 (SARS-CoV-2) (coronavirus disease [COVID-19]) vaccine, mRNA-LNP, spike protein, preservative free, 3 mcg/0.2 mL dosage, diluent reconstituted, tris-sucrose formulation; second dose
or	medication	NLM:RXNORM:2610328	SARS-CoV-2 (COVID-19) vaccine, mRNA-1273 0.05 MG/ML / SARS-CoV-2 (COVID-19) vaccine, mRNA-1273 OMICRON (BA.4/BA.5) 0.05 MG/ML Injectable Suspension
or	procedure	UMLS:CPT:0154A	Immunization administration by intramuscular injection of severe acute respiratory syndrome coronavirus 2 (SARS-CoV-2) (coronavirus disease

			[COVID-19]) vaccine, mRNA-LNP, bivalent spike protein, preservative free, 10 mcg/0.2 mL dosage, diluent reconstituted, tris-sucrose formulation, booster dose
or	procedure	UMLS:CPT:0053A	Immunization administration by intramuscular injection of severe acute respiratory syndrome coronavirus 2 (SARS-CoV-2) (coronavirus disease [COVID-19]) vaccine, mRNA-LNP, spike protein, preservative free, 30 mcg/0.3 mL dosage, tris-sucrose formulation; third dose
or	procedure	UMLS:CPT:1037332	Immunization administration by intramuscular injection of severe acute respiratory syndrome coronavirus 2 (SARS-CoV-2) (coronavirus disease [COVID-19]) vaccine, mRNA-LNP, spike protein, preservative free, 25 mcg/0.25 mL dosage
or	procedure	UMLS:CPT:0052A	Immunization administration by intramuscular injection of severe acute respiratory syndrome coronavirus 2 (SARS-CoV-2) (coronavirus disease [COVID-19]) vaccine, mRNA-LNP, spike protein, preservative free, 30 mcg/0.3 mL dosage, tris-sucrose formulation; second dose
or	procedure	UMLS:CPT:0111A	Immunization administration by intramuscular injection of severe acute respiratory syndrome coronavirus 2 (SARS-

			CoV-2) (coronavirus disease [COVID-19]) vaccine, mRNA-LNP, spike protein, preservative free, 25 mcg/0.25 mL dosage; first dose
or	procedure	UMLS:CPT:0051A	Immunization administration by intramuscular injection of severe acute respiratory syndrome coronavirus 2 (SARS-CoV-2) (coronavirus disease [COVID-19]) vaccine, mRNA-LNP, spike protein, preservative free, 30 mcg/0.3 mL dosage, tris-sucrose formulation; first dose
or	procedure	UMLS:CPT:91311	Severe acute respiratory syndrome coronavirus 2 (SARS-CoV-2) (coronavirus disease [COVID-19]) vaccine, mRNA-LNP, spike protein, preservative free, 25 mcg/0.25 mL dosage, for intramuscular use
or	procedure	UMLS:CPT:0074A	Immunization administration by intramuscular injection of severe acute respiratory syndrome coronavirus 2 (SARS-CoV-2) (coronavirus disease [COVID-19]) vaccine, mRNA-LNP, spike protein, preservative free, 10 mcg/0.2 mL dosage, diluent reconstituted, tris-sucrose formulation; booster dose
or	procedure	UMLS:CPT:0112A	Immunization administration by intramuscular injection of severe acute respiratory syndrome coronavirus 2 (SARS-CoV-2) (coronavirus disease

			[COVID-19]) vaccine, mRNA-LNP, spike protein, preservative free, 25 mcg/0.25 mL dosage; second dose
or	procedure	UMLS:CPT:0083A	Immunization administration by intramuscular injection of severe acute respiratory syndrome coronavirus 2 (SARS-CoV-2) (coronavirus disease [COVID-19]) vaccine, mRNA-LNP, spike protein, preservative free, 3 mcg/0.2 mL dosage, diluent reconstituted, tris-sucrose formulation; third dose
or	procedure	UMLS:CPT:0073A	Immunization administration by intramuscular injection of severe acute respiratory syndrome coronavirus 2 (SARS-CoV-2) (coronavirus disease [COVID-19]) vaccine, mRNA-LNP, spike protein, preservative free, 10 mcg/0.2 mL dosage, diluent reconstituted, tris-sucrose formulation; third dose
or	procedure	UMLS:CPT:0173A	Immunization administration by intramuscular injection of severe acute respiratory syndrome coronavirus 2 (SARS-CoV-2) (coronavirus disease [COVID-19]) vaccine, mRNA-LNP, bivalent spike protein, preservative free, 3 mcg/0.2 mL dosage, diluent reconstituted, tris-sucrose formulation, third dose
or	procedure	UMLS:CPT:0164A	Immunization administration by intramuscular injection of severe acute respiratory

			syndrome coronavirus 2 (SARS-CoV-2) (coronavirus disease [COVID-19]) vaccine, mRNA-LNP, spike protein, bivalent, preservative free, 10 mcg/0.2 mL dosage, booster dose
or	procedure	UMLS:CPT:1037838	Immunization administration by intramuscular injection of severe acute respiratory syndrome coronavirus 2 (SARS-CoV-2) (coronavirus disease [COVID-19]) vaccine, mRNA-LNP, spike protein, preservative free, 50 mcg/0.5 mL dosage
or	procedure	UMLS:CPT:0094A	Immunization administration by intramuscular injection of severe acute respiratory syndrome coronavirus 2 (SARS-CoV-2) (coronavirus disease [COVID-19]) vaccine, mRNA-LNP, spike protein, preservative free, 50 mcg/0.5 mL dosage; booster dose, when administered to individuals 18 years and over
or	procedure	UMLS:CPT:0034A	Immunization administration by intramuscular injection of severe acute respiratory syndrome coronavirus 2 (SARS-CoV-2) (coronavirus disease [COVID-19]) vaccine, DNA, spike protein, adenovirus type 26 (Ad26) vector, preservative free, 5x1010 viral particles/0.5 mL dosage; booster dose
or	procedure	UMLS:CPT:0144A	Immunization administration by intramuscular injection of severe acute respiratory

			syndrome coronavirus 2 (SARS-CoV-2) (coronavirus disease [COVID-19]) vaccine, mRNA-LNP, spike protein, bivalent, preservative free, 25 mcg/0.25 mL dosage, booster dose
or	procedure	UMLS:CPT:0091A	Immunization administration by intramuscular injection of severe acute respiratory syndrome coronavirus 2 (SARS-CoV-2) (coronavirus disease [COVID-19]) vaccine, mRNA-LNP, spike protein, preservative free, 50 mcg/0.5 mL dosage; first dose, when administered to individuals 6 through 11 years
or	procedure	UMLS:CPT:0174A	Immunization administration by intramuscular injection of severe acute respiratory syndrome coronavirus 2 (SARS-CoV-2) (coronavirus disease [COVID-19]) vaccine, mRNA-LNP, bivalent spike protein, preservative free, 3 mcg/0.2 mL dosage, diluent reconstituted, tris-sucrose formulation, booster
or	procedure	UMLS:CPT:0092A	Immunization administration by intramuscular injection of severe acute respiratory syndrome coronavirus 2 (SARS-CoV-2) (coronavirus disease [COVID-19]) vaccine, mRNA-LNP, spike protein, preservative free, 50 mcg/0.5 mL dosage; second dose, when administered to individuals 6 through 11 years

or	procedure	UMLS:CPT:1036682	Immunization administration by intramuscular injection of severe acute respiratory syndrome coronavirus 2 (SARS-CoV-2) (coronavirus disease [COVID-19]) vaccine, recombinant spike protein nanoparticle, saponin-based adjuvant, preservative free, 5 mcg/0.5 mL dosage
or	procedure	UMLS:CPT:0041A	Immunization administration by intramuscular injection of severe acute respiratory syndrome coronavirus 2 (SARS-CoV-2) (coronavirus disease [COVID-19]) vaccine, recombinant spike protein nanoparticle, saponin-based adjuvant, preservative free, 5 mcg/0.5 mL dosage; first dose
or	procedure	UMLS:CPT:0113A	Immunization administration by intramuscular injection of severe acute respiratory syndrome coronavirus 2 (SARS-CoV-2) (coronavirus disease [COVID-19]) vaccine, mRNA-LNP, spike protein, preservative free, 25 mcg/0.25 mL dosage; third dose
or	procedure	UMLS:CPT:0042A	Immunization administration by intramuscular injection of severe acute respiratory syndrome coronavirus 2 (SARS-CoV-2) (coronavirus disease [COVID-19]) vaccine, recombinant spike protein nanoparticle, saponin-based adjuvant, preservative free, 5

				mcg/0.5 mL dosage; second dose
	or	procedure	UMLS:CPT:0093A	Immunization administration by intramuscular injection of severe acute respiratory syndrome coronavirus 2 (SARS-CoV-2) (coronavirus disease [COVID-19]) vaccine, mRNA-LNP, spike protein, preservative free, 50 mcg/0.5 mL dosage; third dose, when administered to individuals 6 through 11 years
	or	procedure	UMLS:CPT:1036666	Immunization administration by intramuscular injection of severe acute respiratory syndrome coronavirus 2 (SARS-CoV-2) (coronavirus disease [COVID-19]) vaccine, DNA, spike protein, chimpanzee adenovirus Oxford 1 (ChAdOx1) vector, preservative free, 5x1010 viral particles/0.5 mL dosage
	or	procedure	UMLS:CPT:0044A	Immunization administration by intramuscular injection of severe acute respiratory syndrome coronavirus 2 (SARS-CoV-2) (coronavirus disease [COVID-19]) vaccine, recombinant spike protein nanoparticle, saponin-based adjuvant, preservative free, 5 mcg/0.5mL dosage; booster
date constraint		The terms in this group occurred between Dec 1, 2019 and Dec 31, 2023		
**Group 2**				
**Group 2A COVID-19 negative**				
must have		visit	TNX:Visit	Visit (Data Source: TriNetX)
cannot have		diagnosis	UMLS:ICD10CM:U07.1	COVID-19

	or	laboratory	UMLS:LNC:95406-5	SARS-CoV-2 (COVID-19) RNA [Presence] in Nose by NAA with probe detection (labResult: Positive)
	or	laboratory	UMLS:LNC:94845-5	SARS-CoV-2 (COVID-19) RNA [Presence] in Saliva (oral fluid) by NAA with probe detection (labResult: Positive)
	or	laboratory	UMLS:LNC:95608-6	SARS-CoV-2 (COVID-19) RNA [Presence] in Respiratory specimen by NAA with non-probe detection (labResult: Positive)
	or	laboratory	UMLS:LNC:94759-8	SARS-CoV-2 (COVID-19) RNA [Presence] in Nasopharynx by NAA with probe detection (labResult: Positive)
	or	laboratory	UMLS:LNC:94565-9	SARS-CoV-2 (COVID-19) RNA [Presence] in Nasopharynx by NAA with non-probe detection (labResult: Positive)
	or	laboratory	UMLS:LNC:94309-2	SARS-CoV-2 (COVID-19) RNA [Presence] in Specimen by NAA with probe detection (labResult: Positive)
	or	laboratory	UMLS:LNC:94500-6	SARS-CoV-2 (COVID-19) RNA [Presence] in Respiratory specimen by NAA with probe detection (labResult: Positive)
	or	diagnosis	UMLS:ICD10CM:U07.2	COVID-19, virus not identified (WHO)
	or	diagnosis	UMLS:ICD10CM:J12.82	Pneumonia due to coronavirus disease 2019
date constraint	The terms in this group occurred at any time			
event relationship	Any instance of thyroid cancer history occurred at least 1 day before the first instance of COVID-19 negative			
**Group 2B thyroid cancer history**				

cannot have		diagnosis	UMLS:ICD10CM:C73	Malignant neoplasm of thyroid gland
	or	diagnosis	UMLS:ICD10CM:Z85.850	Personal history of malignant neoplasm of thyroid

Query Criteria for Cohort post-COVID, 41-60

This query was run on the network Global Collaborative Network with 148 HCO(s) queried and 148 HCO(s) responded. A total of 121 provider(s) responded with patients. The final cohort included 749,488 patients who matched the query criteria listed in the table below.

**Ungrouped terms**				
must have		demographics	Age	Age (between 41 and 60 years (most recent occurrence))
	and any of	demographics	UMLS:HL7V3.0:Gender:M	Male
		demographics	UMLS:HL7V3.0:Gender:F	Female
**Group 1**				
**COVID-19, no vaccination**				
must have	any of	diagnosis	UMLS:ICD10CM:U07.1	COVID-19
		diagnosis	UMLS:ICD10CM:U07.2	COVID-19, virus not identified (WHO)
		diagnosis	UMLS:ICD10CM:J12.82	Pneumonia due to coronavirus disease 2019
		laboratory	UMLS:LNC:94500-6	SARS-CoV-2 (COVID-19) RNA [Presence] in Respiratory specimen by NAA with probe detection (labResult: Positive)
		laboratory	UMLS:LNC:94309-2	SARS-CoV-2 (COVID-19) RNA [Presence] in Specimen by NAA with probe detection (labResult: Positive)
		laboratory	UMLS:LNC:94565-9	SARS-CoV-2 (COVID-19) RNA [Presence] in Nasopharynx by NAA with non-probe detection (labResult: Positive)

		laboratory	UMLS:LNC:94759-8	SARS-CoV-2 (COVID-19) RNA [Presence] in Nasopharynx by NAA with probe detection (labResult: Positive)
		laboratory	UMLS:LNC:95608-6	SARS-CoV-2 (COVID-19) RNA [Presence] in Respiratory specimen by NAA with non-probe detection (labResult: Positive)
		laboratory	UMLS:LNC:94845-5	SARS-CoV-2 (COVID-19) RNA [Presence] in Saliva (oral fluid) by NAA with probe detection (labResult: Positive)
		laboratory	UMLS:LNC:95406-5	SARS-CoV-2 (COVID-19) RNA [Presence] in Nose by NAA with probe detection (labResult: Positive)
cannot have		medication	NLM:CVX:208	COVID-19, mRNA, LNP-S, PF, 30 mcg/0.3 mL dose
	or	medication	NLM:CVX:207	COVID-19, mRNA, LNP-S, PF, 100 mcg/0.5mL dose or 50 mcg/0.25mL dose
	or	medication	NLM:CVX:212	COVID-19 vaccine, vector-nr, rS-Ad26, PF, 0.5 mL
	or	medication	NLM:RXNORM:OMOP5042939	COVID-19 vaccine
	or	medication	NLM:CVX:300	COVID-19, mRNA, LNP-S, bivalent, PF, 30 mcg/0.3 mL dose
	or	medication	NLM:CVX:217	COVID-19, mRNA, LNP-S, PF, 30 mcg/0.3 mL dose, tris-sucrose
	or	medication	NLM:CVX:229	COVID-19, mRNA, LNP-S, bivalent, PF, 50 mcg/0.5 mL or 25mcg/0.25 mL dose
	or	medication	NLM:CVX:218	COVID-19, mRNA, LNP-S, PF, 10 mcg/0.2 mL dose, tris-sucrose
	or	medication	NLM:CVX:520	COVID-19 mRNA, bivalent, original/Omicron BA.1, Non-US

			Vaccine Product, Pfizer-BioNTech
or	medication	NLM:CVX:519	COVID-19 mRNA, bivalent, original/Omicron BA.1, Non-US Vaccine (Spikevax Bivalent), Moderna
or	medication	NLM:CVX:301	COVID-19, mRNA, LNP-S, bivalent, PF, 10 mcg/0.2 mL dose
or	medication	NLM:CVX:219	COVID-19, mRNA, LNP-S, PF, 3 mcg/0.2 mL dose, tris-sucrose
or	medication	NLM:CVX:228	COVID-19, mRNA, LNP-S, PF, pediatric 25 mcg/0.25 mL dose
or	medication	NLM:CVX:230	COVID-19, mRNA, LNP-S, bivalent booster, PF, 10 mcg/0.2 mL
or	medication	NLM:CVX:221	COVID-19, mRNA, LNP-S, PF, 50 mcg/0.5 mL dose
or	medication	NLM:CVX:210	COVID-19 vaccine, vector-nr, rS-ChAdOx1, PF, 0.5 mL
or	medication	NLM:CVX:302	COVID-19, mRNA, LNP-S, bivalent, PF, 3 mcg/0.2 mL dose
or	medication	NLM:CVX:511	COVID-19 IV Non-US Vaccine (CoronaVac, Sinovac)
or	medication	NLM:RXNORM:2468231	SARS-CoV-2 (COVID-19) vaccine, mRNA spike protein
or	procedure	UMLS:CPT:91300	Severe acute respiratory syndrome coronavirus 2 (SARS-CoV-2) (coronavirus disease [COVID-19]) vaccine, mRNA-LNP, spike protein, preservative free, 30 mcg/0.3 mL dosage, diluent reconstituted, for intramuscular use
or	procedure	UMLS:CPT:0001A	Immunization administration by intramuscular injection of severe acute respiratory syndrome coronavirus 2 (SARS-

			CoV-2) (coronavirus disease [COVID-19]) vaccine, mRNA-LNP, spike protein, preservative free, 30 mcg/0.3 mL dosage, diluent reconstituted; first dose
or	procedure	UMLS:CPT:0002A	Immunization administration by intramuscular injection of severe acute respiratory syndrome coronavirus 2 (SARS-CoV-2) (coronavirus disease [COVID-19]) vaccine, mRNA-LNP, spike protein, preservative free, 30 mcg/0.3 mL dosage, diluent reconstituted; second dose
or	procedure	UMLS:CPT:91301	Severe acute respiratory syndrome coronavirus 2 (SARS-CoV-2) (coronavirus disease [COVID-19]) vaccine, mRNA-LNP, spike protein, preservative free, 100 mcg/0.5 mL dosage, for intramuscular use
or	procedure	UMLS:CPT:0011A	Immunization administration by intramuscular injection of severe acute respiratory syndrome coronavirus 2 (SARS-CoV-2) (coronavirus disease [COVID-19]) vaccine, mRNA-LNP, spike protein, preservative free, 100 mcg/0.5 mL dosage; first dose
or	procedure	UMLS:CPT:0012A	Immunization administration by intramuscular injection of severe acute respiratory syndrome coronavirus 2 (SARS-CoV-2) (coronavirus disease [COVID-19]) vaccine, mRNA-LNP, spike protein, preservative

			free, 100 mcg/0.5 mL dosage; second dose
or	procedure	UMLS:SNOMED:840534001	Administration of SARS-CoV-2 antigen vaccine
or	medication	NLM:CVX:213	SARS-CoV-2 (COVID-19) Vaccine
or	procedure	UMLS:CPT:1036660	Immunization administration by intramuscular injection of severe acute respiratory syndrome coronavirus 2 (SARS-CoV-2) (coronavirus disease [COVID-19]) vaccine, mRNA-LNP, spike protein, preservative free, 30 mcg/0.3 mL dosage, diluent reconstituted
or	procedure	UMLS:CPT:1036663	Immunization administration by intramuscular injection of severe acute respiratory syndrome coronavirus 2 (SARS-CoV-2) (coronavirus disease [COVID-19]) vaccine, mRNA-LNP, spike protein, preservative free, 100 mcg/0.5 mL dosage
or	procedure	UMLS:CPT:0124A	Immunization administration by intramuscular injection of severe acute respiratory syndrome coronavirus 2 (SARS-CoV-2) (coronavirus disease [COVID-19]) vaccine, mRNA-LNP, bivalent spike protein, preservative free, 30 mcg/0.3 mL dosage, tris-sucrose formulation, booster dose
or	procedure	UMLS:CPT:0004A	Immunization administration by intramuscular injection of severe acute respiratory syndrome coronavirus 2 (SARS-CoV-2) (coronavirus disease

			[COVID-19]) vaccine, mRNA-LNP, spike protein, preservative free, 30 mcg/0.3 mL dosage, diluent reconstituted; booster dose
or	procedure	UMLS:CPT:0003A	Immunization administration by intramuscular injection of severe acute respiratory syndrome coronavirus 2 (SARS-CoV-2) (coronavirus disease [COVID-19]) vaccine, mRNA-LNP, spike protein, preservative free, 30 mcg/0.3 mL dosage, diluent reconstituted; third dose
or	procedure	UMLS:CPT:1037166	Immunization administration by intramuscular injection of severe acute respiratory syndrome coronavirus 2 (SARS-CoV-2) (coronavirus disease [COVID-19]) vaccine, mRNA-LNP, spike protein, preservative free, 30 mcg/0.3 mL dosage, tris-sucrose formulation
or	procedure	UMLS:CPT:0054A	Immunization administration by intramuscular injection of severe acute respiratory syndrome coronavirus 2 (SARS-CoV-2) (coronavirus disease [COVID-19]) vaccine, mRNA-LNP, spike protein, preservative free, 30 mcg/0.3 mL dosage, tris-sucrose formulation; booster dose
or	procedure	UMLS:CPT:0064A	Immunization administration by intramuscular injection of severe acute respiratory syndrome coronavirus 2 (SARS-CoV-2) (coronavirus disease

			[COVID-19]) vaccine, mRNA-LNP, spike protein, preservative free, 50 mcg/0.25 mL dosage, booster dose
or	procedure	UMLS:CPT:90480	Immunization administration by intramuscular injection of severe acute respiratory syndrome coronavirus 2 (SARS-CoV-2) (coronavirus disease [COVID-19]) vaccine, single dose
or	procedure	UMLS:CPT:1037171	Immunization administration by intramuscular injection of severe acute respiratory syndrome coronavirus 2 (SARS-CoV-2) (coronavirus disease [COVID-19]) vaccine, mRNA-LNP, spike protein, preservative free, 10 mcg/0.2 mL dosage, diluent reconstituted, tris-sucrose formulation
or	procedure	UMLS:CPT:0071A	Immunization administration by intramuscular injection of severe acute respiratory syndrome coronavirus 2 (SARS-CoV-2) (coronavirus disease [COVID-19]) vaccine, mRNA-LNP, spike protein, preservative free, 10 mcg/0.2 mL dosage, diluent reconstituted, tris-sucrose formulation; first dose
or	procedure	UMLS:CPT:0072A	Immunization administration by intramuscular injection of severe acute respiratory syndrome coronavirus 2 (SARS-CoV-2) (coronavirus disease [COVID-19]) vaccine, mRNA-LNP, spike protein, preservative

			free, 10 mcg/0.2 mL dosage, diluent reconstituted, tris-sucrose formulation; second dose
or	medication	NLM:RXNORM:2610319	SARS-CoV-2 (COVID-19) vaccine, mRNA-BNT162b2 0.05 MG/ML / SARS-CoV-2 (COVID-19) vaccine, mRNA-BNT162b2 OMICRON (BA.4/BA.5) 0.05 MG/ML Injectable Suspension
or	procedure	UMLS:CPT:91313	Severe acute respiratory syndrome coronavirus 2 (SARS-CoV-2) (coronavirus disease [COVID-19]) vaccine, mRNA-LNP, spike protein, bivalent, preservative free, 50 mcg/0.5 mL dosage, for intramuscular use
or	procedure	UMLS:CPT:0134A	Immunization administration by intramuscular injection of severe acute respiratory syndrome coronavirus 2 (SARS-CoV-2) (coronavirus disease [COVID-19]) vaccine, mRNA-LNP, spike protein, bivalent, preservative free, 50 mcg/0.5 mL dosage, booster dose
or	procedure	UMLS:CPT:1037175	Immunization administration by intramuscular injection of severe acute respiratory syndrome coronavirus 2 (SARS-CoV-2) (coronavirus disease [COVID-19]) vaccine, DNA, spike protein, adenovirus type 26 (Ad26) vector, preservative free, 5x1010 viral particles/0.5 mL dosage
or	medication	NLM:RXNORM:2610347	0.3 ML SARS-CoV-2 (COVID-19) vaccine, mRNA-BNT162b2 0.05

			MG/ML / SARS-CoV-2 (COVID-19) vaccine, mRNA-BNT162b2 OMICRON (BA.4/BA.5) -1 MG/ML Injection
or	procedure	UMLS:CPT:1037228	Immunization administration by intramuscular injection of severe acute respiratory syndrome coronavirus 2 (SARS-CoV-2) (coronavirus disease [COVID-19]) vaccine, mRNA-LNP, spike protein, preservative free, 3 mcg/0.2 mL dosage, diluent reconstituted, tris-sucrose formulation
or	procedure	UMLS:CPT:0013A	Immunization administration by intramuscular injection of severe acute respiratory syndrome coronavirus 2 (SARS-CoV-2) (coronavirus disease [COVID-19]) vaccine, mRNA-LNP, spike protein, preservative free, 100 mcg/0.5 mL dosage; third dose
or	procedure	UMLS:CPT:0081A	Immunization administration by intramuscular injection of severe acute respiratory syndrome coronavirus 2 (SARS-CoV-2) (coronavirus disease [COVID-19]) vaccine, mRNA-LNP, spike protein, preservative free, 3 mcg/0.2 mL dosage, diluent reconstituted, tris-sucrose formulation; first dose
or	procedure	UMLS:CPT:0082A	Immunization administration by intramuscular injection of severe acute respiratory syndrome coronavirus 2 (SARS-CoV-2) (coronavirus disease

			[COVID-19]) vaccine, mRNA-LNP, spike protein, preservative free, 3 mcg/0.2 mL dosage, diluent reconstituted, tris-sucrose formulation; second dose
or	medication	NLM:RXNORM:2610328	SARS-CoV-2 (COVID-19) vaccine, mRNA-1273 0.05 MG/ML / SARS-CoV-2 (COVID-19) vaccine, mRNA-1273 OMICRON (BA.4/BA.5) 0.05 MG/ML Injectable Suspension
or	procedure	UMLS:CPT:0154A	Immunization administration by intramuscular injection of severe acute respiratory syndrome coronavirus 2 (SARS-CoV-2) (coronavirus disease [COVID-19]) vaccine, mRNA-LNP, bivalent spike protein, preservative free, 10 mcg/0.2 mL dosage, diluent reconstituted, tris-sucrose formulation, booster dose
or	procedure	UMLS:CPT:0053A	Immunization administration by intramuscular injection of severe acute respiratory syndrome coronavirus 2 (SARS-CoV-2) (coronavirus disease [COVID-19]) vaccine, mRNA-LNP, spike protein, preservative free, 30 mcg/0.3 mL dosage, tris-sucrose formulation; third dose
or	procedure	UMLS:CPT:1037332	Immunization administration by intramuscular injection of severe acute respiratory syndrome coronavirus 2 (SARS-CoV-2) (coronavirus disease

			[COVID-19]) vaccine, mRNA-LNP, spike protein, preservative free, 25 mcg/0.25 mL dosage
or	procedure	UMLS:CPT:0052A	Immunization administration by intramuscular injection of severe acute respiratory syndrome coronavirus 2 (SARS-CoV-2) (coronavirus disease [COVID-19]) vaccine, mRNA-LNP, spike protein, preservative free, 30 mcg/0.3 mL dosage, tris-sucrose formulation; second dose
or	procedure	UMLS:CPT:0111A	Immunization administration by intramuscular injection of severe acute respiratory syndrome coronavirus 2 (SARS-CoV-2) (coronavirus disease [COVID-19]) vaccine, mRNA-LNP, spike protein, preservative free, 25 mcg/0.25 mL dosage; first dose
or	procedure	UMLS:CPT:0051A	Immunization administration by intramuscular injection of severe acute respiratory syndrome coronavirus 2 (SARS-CoV-2) (coronavirus disease [COVID-19]) vaccine, mRNA-LNP, spike protein, preservative free, 30 mcg/0.3 mL dosage, tris-sucrose formulation; first dose
or	procedure	UMLS:CPT:91311	Severe acute respiratory syndrome coronavirus 2 (SARS-CoV-2) (coronavirus disease [COVID-19]) vaccine, mRNA-LNP, spike protein, preservative

			free, 25 mcg/0.25 mL dosage, for intramuscular use
or	procedure	UMLS:CPT:0074A	Immunization administration by intramuscular injection of severe acute respiratory syndrome coronavirus 2 (SARS-CoV-2) (coronavirus disease [COVID-19]) vaccine, mRNA-LNP, spike protein, preservative free, 10 mcg/0.2 mL dosage, diluent reconstituted, tris-sucrose formulation; booster dose
or	procedure	UMLS:CPT:0112A	Immunization administration by intramuscular injection of severe acute respiratory syndrome coronavirus 2 (SARS-CoV-2) (coronavirus disease [COVID-19]) vaccine, mRNA-LNP, spike protein, preservative free, 25 mcg/0.25 mL dosage; second dose
or	procedure	UMLS:CPT:0083A	Immunization administration by intramuscular injection of severe acute respiratory syndrome coronavirus 2 (SARS-CoV-2) (coronavirus disease [COVID-19]) vaccine, mRNA-LNP, spike protein, preservative free, 3 mcg/0.2 mL dosage, diluent reconstituted, tris-sucrose formulation; third dose
or	procedure	UMLS:CPT:0073A	Immunization administration by intramuscular injection of severe acute respiratory syndrome coronavirus 2 (SARS-CoV-2) (coronavirus disease [COVID-19]) vaccine, mRNA-

			LNP, spike protein, preservative free, 10 mcg/0.2 mL dosage, diluent reconstituted, tris-sucrose formulation; third dose
or	procedure	UMLS:CPT:0173A	Immunization administration by intramuscular injection of severe acute respiratory syndrome coronavirus 2 (SARS-CoV-2) (coronavirus disease [COVID-19]) vaccine, mRNA-LNP, bivalent spike protein, preservative free, 3 mcg/0.2 mL dosage, diluent reconstituted, tris-sucrose formulation, third dose
or	procedure	UMLS:CPT:0164A	Immunization administration by intramuscular injection of severe acute respiratory syndrome coronavirus 2 (SARS-CoV-2) (coronavirus disease [COVID-19]) vaccine, mRNA-LNP, spike protein, bivalent, preservative free, 10 mcg/0.2 mL dosage, booster dose
or	procedure	UMLS:CPT:1037838	Immunization administration by intramuscular injection of severe acute respiratory syndrome coronavirus 2 (SARS-CoV-2) (coronavirus disease [COVID-19]) vaccine, mRNA-LNP, spike protein, preservative free, 50 mcg/0.5 mL dosage
or	procedure	UMLS:CPT:0094A	Immunization administration by intramuscular injection of severe acute respiratory syndrome coronavirus 2 (SARS-CoV-2) (coronavirus disease [COVID-19]) vaccine, mRNA-

			LNP, spike protein, preservative free, 50 mcg/0.5 mL dosage; booster dose, when administered to individuals 18 years and over
or	procedure	UMLS:CPT:0034A	Immunization administration by intramuscular injection of severe acute respiratory syndrome coronavirus 2 (SARS-CoV-2) (coronavirus disease [COVID-19]) vaccine, DNA, spike protein, adenovirus type 26 (Ad26) vector, preservative free, 5x1010 viral particles/0.5 mL dosage; booster dose
or	procedure	UMLS:CPT:0144A	Immunization administration by intramuscular injection of severe acute respiratory syndrome coronavirus 2 (SARS-CoV-2) (coronavirus disease [COVID-19]) vaccine, mRNA-LNP, spike protein, bivalent, preservative free, 25 mcg/0.25 mL dosage, booster dose
or	procedure	UMLS:CPT:0091A	Immunization administration by intramuscular injection of severe acute respiratory syndrome coronavirus 2 (SARS-CoV-2) (coronavirus disease [COVID-19]) vaccine, mRNA-LNP, spike protein, preservative free, 50 mcg/0.5 mL dosage; first dose, when administered to individuals 6 through 11 years
or	procedure	UMLS:CPT:0174A	Immunization administration by intramuscular injection of severe acute respiratory syndrome coronavirus 2 (SARS-

			CoV-2) (coronavirus disease [COVID-19]) vaccine, mRNA-LNP, bivalent spike protein, preservative free, 3 mcg/0.2 mL dosage, diluent reconstituted, tris-sucrose formulation, booster
or	procedure	UMLS:CPT:0092A	Immunization administration by intramuscular injection of severe acute respiratory syndrome coronavirus 2 (SARS-CoV-2) (coronavirus disease [COVID-19]) vaccine, mRNA-LNP, spike protein, preservative free, 50 mcg/0.5 mL dosage; second dose, when administered to individuals 6 through 11 years
or	procedure	UMLS:CPT:1036682	Immunization administration by intramuscular injection of severe acute respiratory syndrome coronavirus 2 (SARS-CoV-2) (coronavirus disease [COVID-19]) vaccine, recombinant spike protein nanoparticle, saponin-based adjuvant, preservative free, 5 mcg/0.5 mL dosage
or	procedure	UMLS:CPT:0041A	Immunization administration by intramuscular injection of severe acute respiratory syndrome coronavirus 2 (SARS-CoV-2) (coronavirus disease [COVID-19]) vaccine, recombinant spike protein nanoparticle, saponin-based adjuvant, preservative free, 5 mcg/0.5 mL dosage; first dose

or	procedure	UMLS:CPT:0113A	Immunization administration by intramuscular injection of severe acute respiratory syndrome coronavirus 2 (SARS-CoV-2) (coronavirus disease [COVID-19]) vaccine, mRNA-LNP, spike protein, preservative free, 25 mcg/0.25 mL dosage; third dose
or	procedure	UMLS:CPT:0042A	Immunization administration by intramuscular injection of severe acute respiratory syndrome coronavirus 2 (SARS-CoV-2) (coronavirus disease [COVID-19]) vaccine, recombinant spike protein nanoparticle, saponin-based adjuvant, preservative free, 5 mcg/0.5 mL dosage; second dose
or	procedure	UMLS:CPT:0093A	Immunization administration by intramuscular injection of severe acute respiratory syndrome coronavirus 2 (SARS-CoV-2) (coronavirus disease [COVID-19]) vaccine, mRNA-LNP, spike protein, preservative free, 50 mcg/0.5 mL dosage; third dose, when administered to individuals 6 through 11 years
or	procedure	UMLS:CPT:1036666	Immunization administration by intramuscular injection of severe acute respiratory syndrome coronavirus 2 (SARS-CoV-2) (coronavirus disease [COVID-19]) vaccine, DNA, spike protein, chimpanzee adenovirus Oxford 1 (ChAdOx1) vector,

				preservative free, 5x1010 viral particles/0.5 mL dosage
	or	procedure	UMLS:CPT:0044A	Immunization administration by intramuscular injection of severe acute respiratory syndrome coronavirus 2 (SARS-CoV-2) (coronavirus disease [COVID-19]) vaccine, recombinant spike protein nanoparticle, saponin-based adjuvant, preservative free, 5 mcg/0.5mL dosage; booster
date constraint		The terms in this group occurred between Dec 1, 2019 and Dec 31, 2023		
**Group 2**				
**Group 2A COVID-19**				
must have	any of	diagnosis	UMLS:ICD10CM:U07.1	COVID-19
		diagnosis	UMLS:ICD10CM:U07.2	COVID-19, virus not identified (WHO)
		diagnosis	UMLS:ICD10CM:J12.82	Pneumonia due to coronavirus disease 2019
		laboratory	UMLS:LNC:94500-6	SARS-CoV-2 (COVID-19) RNA [Presence] in Respiratory specimen by NAA with probe detection (labResult: Positive)
		laboratory	UMLS:LNC:94309-2	SARS-CoV-2 (COVID-19) RNA [Presence] in Specimen by NAA with probe detection (labResult: Positive)
		laboratory	UMLS:LNC:94565-9	SARS-CoV-2 (COVID-19) RNA [Presence] in Nasopharynx by NAA with non-probe detection (labResult: Positive)
		laboratory	UMLS:LNC:94759-8	SARS-CoV-2 (COVID-19) RNA [Presence] in Nasopharynx by NAA with probe detection (labResult: Positive)

		laboratory	UMLS:LNC:95608-6	SARS-CoV-2 (COVID-19) RNA [Presence] in Respiratory specimen by NAA with non-probe detection (labResult: Positive)
		laboratory	UMLS:LNC:94845-5	SARS-CoV-2 (COVID-19) RNA [Presence] in Saliva (oral fluid) by NAA with probe detection (labResult: Positive)
		laboratory	UMLS:LNC:95406-5	SARS-CoV-2 (COVID-19) RNA [Presence] in Nose by NAA with probe detection (labResult: Positive)
	and	visit	TNX:Visit	Visit (Data Source: TriNetX)
date constraint		The terms in this group occurred at any time		
event relationship		Any instance of thyroid cancer history occurred at least 1 day before the first instance of COVID-19		
**Group 2B thyroid cancer history**				
cannot have		diagnosis	UMLS:ICD10CM:C73	Malignant neoplasm of thyroid gland
	or	diagnosis	UMLS:ICD10CM:Z85.850	Personal history of malignant neoplasm of thyroid

Query Criteria for Cohort non-COVID, 41-60

This query was run on the network Global Collaborative Network with 148 HCO(s) queried and 148 HCO(s) responded. A total of 130 provider(s) responded with patients. The final cohort included 9,079,452 patients who matched the query criteria listed in the table below.

**Ungrouped terms**				
must have		demographics	Age	Age (between 41 and 60 years (most recent occurrence))
	and any of	demographics	UMLS:HL7V3.0:Gender:M	Male
		demographics	UMLS:HL7V3.0:Gender:F	Female
**Group 1**				
**COVID-19 negative, no vaccination**				
must have		visit	TNX:Visit	Visit

cannot have		medication	NLM:CVX:208	COVID-19, mRNA, LNP-S, PF, 30 mcg/0.3 mL dose
	or	medication	NLM:CVX:207	COVID-19, mRNA, LNP-S, PF, 100 mcg/0.5mL dose or 50 mcg/0.25mL dose
	or	medication	NLM:CVX:212	COVID-19 vaccine, vector-nr, rS-Ad26, PF, 0.5 mL
	or	medication	NLM:RXNORM:OMOP 5042939	COVID-19 vaccine
	or	medication	NLM:CVX:300	COVID-19, mRNA, LNP-S, bivalent, PF, 30 mcg/0.3 mL dose
	or	medication	NLM:CVX:217	COVID-19, mRNA, LNP-S, PF, 30 mcg/0.3 mL dose, tris-sucrose
	or	medication	NLM:CVX:229	COVID-19, mRNA, LNP-S, bivalent, PF, 50 mcg/0.5 mL or 25mcg/0.25 mL dose
	or	medication	NLM:CVX:218	COVID-19, mRNA, LNP-S, PF, 10 mcg/0.2 mL dose, tris-sucrose
	or	medication	NLM:CVX:520	COVID-19 mRNA, bivalent, original/Omicron BA.1, Non-US Vaccine Product, Pfizer-BioNTech
	or	medication	NLM:CVX:519	COVID-19 mRNA, bivalent, original/Omicron BA.1, Non-US Vaccine (Spikevax Bivalent), Moderna
	or	medication	NLM:CVX:301	COVID-19, mRNA, LNP-S, bivalent, PF, 10 mcg/0.2 mL dose
	or	medication	NLM:CVX:219	COVID-19, mRNA, LNP-S, PF, 3 mcg/0.2 mL dose, tris-sucrose
	or	medication	NLM:CVX:228	COVID-19, mRNA, LNP-S, PF, pediatric 25 mcg/0.25 mL dose
	or	medication	NLM:CVX:230	COVID-19, mRNA, LNP-S, bivalent booster, PF, 10 mcg/0.2 mL

or	medication	NLM:CVX:221	COVID-19, mRNA, LNP-S, PF, 50 mcg/0.5 mL dose
or	medication	NLM:CVX:210	COVID-19 vaccine, vector-nr, rS-ChAdOx1, PF, 0.5 mL
or	medication	NLM:CVX:302	COVID-19, mRNA, LNP-S, bivalent, PF, 3 mcg/0.2 mL dose
or	medication	NLM:CVX:511	COVID-19 IV Non-US Vaccine (CoronaVac, Sinovac)
or	medication	NLM:RXNORM:2468231	SARS-CoV-2 (COVID-19) vaccine, mRNA spike protein
or	procedure	UMLS:CPT:91300	Severe acute respiratory syndrome coronavirus 2 (SARS-CoV-2) (coronavirus disease [COVID-19]) vaccine, mRNA-LNP, spike protein, preservative free, 30 mcg/0.3 mL dosage, diluent reconstituted, for intramuscular use
or	procedure	UMLS:CPT:0001A	Immunization administration by intramuscular injection of severe acute respiratory syndrome coronavirus 2 (SARS-CoV-2) (coronavirus disease [COVID-19]) vaccine, mRNA-LNP, spike protein, preservative free, 30 mcg/0.3 mL dosage, diluent reconstituted; first dose
or	procedure	UMLS:CPT:0002A	Immunization administration by intramuscular injection of severe acute respiratory syndrome coronavirus 2 (SARS-CoV-2) (coronavirus disease [COVID-19]) vaccine, mRNA-LNP, spike protein, preservative free, 30 mcg/0.3 mL dosage, diluent reconstituted; second dose

or	procedure	UMLS:CPT:91301	Severe acute respiratory syndrome coronavirus 2 (SARS-CoV-2) (coronavirus disease [COVID-19]) vaccine, mRNA-LNP, spike protein, preservative free, 100 mcg/0.5 mL dosage, for intramuscular use
or	procedure	UMLS:CPT:0011A	Immunization administration by intramuscular injection of severe acute respiratory syndrome coronavirus 2 (SARS-CoV-2) (coronavirus disease [COVID-19]) vaccine, mRNA-LNP, spike protein, preservative free, 100 mcg/0.5 mL dosage; first dose
or	procedure	UMLS:CPT:0012A	Immunization administration by intramuscular injection of severe acute respiratory syndrome coronavirus 2 (SARS-CoV-2) (coronavirus disease [COVID-19]) vaccine, mRNA-LNP, spike protein, preservative free, 100 mcg/0.5 mL dosage; second dose
or	procedure	UMLS:SNOMED:840534001	Administration of SARS-CoV-2 antigen vaccine
or	medication	NLM:CVX:213	SARS-CoV-2 (COVID-19) Vaccine
or	procedure	UMLS:CPT:1036660	Immunization administration by intramuscular injection of severe acute respiratory syndrome coronavirus 2 (SARS-CoV-2) (coronavirus disease [COVID-19]) vaccine, mRNA-LNP, spike protein, preservative free, 30 mcg/0.3 mL dosage, diluent reconstituted

or	procedure	UMLS:CPT:1036663	Immunization administration by intramuscular injection of severe acute respiratory syndrome coronavirus 2 (SARS-CoV-2) (coronavirus disease [COVID-19]) vaccine, mRNA-LNP, spike protein, preservative free, 100 mcg/0.5 mL dosage
or	procedure	UMLS:CPT:0124A	Immunization administration by intramuscular injection of severe acute respiratory syndrome coronavirus 2 (SARS-CoV-2) (coronavirus disease [COVID-19]) vaccine, mRNA-LNP, bivalent spike protein, preservative free, 30 mcg/0.3 mL dosage, tris-sucrose formulation, booster dose
or	procedure	UMLS:CPT:0004A	Immunization administration by intramuscular injection of severe acute respiratory syndrome coronavirus 2 (SARS-CoV-2) (coronavirus disease [COVID-19]) vaccine, mRNA-LNP, spike protein, preservative free, 30 mcg/0.3 mL dosage, diluent reconstituted; booster dose
or	procedure	UMLS:CPT:0003A	Immunization administration by intramuscular injection of severe acute respiratory syndrome coronavirus 2 (SARS-CoV-2) (coronavirus disease [COVID-19]) vaccine, mRNA-LNP, spike protein, preservative free, 30 mcg/0.3 mL dosage, diluent reconstituted; third dose

or	procedure	UMLS:CPT:1037166	Immunization administration by intramuscular injection of severe acute respiratory syndrome coronavirus 2 (SARS-CoV-2) (coronavirus disease [COVID-19]) vaccine, mRNA-LNP, spike protein, preservative free, 30 mcg/0.3 mL dosage, tris-sucrose formulation
or	procedure	UMLS:CPT:0054A	Immunization administration by intramuscular injection of severe acute respiratory syndrome coronavirus 2 (SARS-CoV-2) (coronavirus disease [COVID-19]) vaccine, mRNA-LNP, spike protein, preservative free, 30 mcg/0.3 mL dosage, tris-sucrose formulation; booster dose
or	procedure	UMLS:CPT:0064A	Immunization administration by intramuscular injection of severe acute respiratory syndrome coronavirus 2 (SARS-CoV-2) (coronavirus disease [COVID-19]) vaccine, mRNA-LNP, spike protein, preservative free, 50 mcg/0.25 mL dosage, booster dose
or	procedure	UMLS:CPT:90480	Immunization administration by intramuscular injection of severe acute respiratory syndrome coronavirus 2 (SARS-CoV-2) (coronavirus disease [COVID-19]) vaccine, single dose
or	procedure	UMLS:CPT:1037171	Immunization administration by intramuscular injection of severe acute respiratory

			syndrome coronavirus 2 (SARS-CoV-2) (coronavirus disease [COVID-19]) vaccine, mRNA-LNP, spike protein, preservative free, 10 mcg/0.2 mL dosage, diluent reconstituted, tris-sucrose formulation
or	procedure	UMLS:CPT:0071A	Immunization administration by intramuscular injection of severe acute respiratory syndrome coronavirus 2 (SARS-CoV-2) (coronavirus disease [COVID-19]) vaccine, mRNA-LNP, spike protein, preservative free, 10 mcg/0.2 mL dosage, diluent reconstituted, tris-sucrose formulation; first dose
or	procedure	UMLS:CPT:0072A	Immunization administration by intramuscular injection of severe acute respiratory syndrome coronavirus 2 (SARS-CoV-2) (coronavirus disease [COVID-19]) vaccine, mRNA-LNP, spike protein, preservative free, 10 mcg/0.2 mL dosage, diluent reconstituted, tris-sucrose formulation; second dose
or	medication	NLM:RXNORM:2610319	SARS-CoV-2 (COVID-19) vaccine, mRNA-BNT162b2 0.05 MG/ML / SARS-CoV-2 (COVID-19) vaccine, mRNA-BNT162b2 OMICRON (BA.4/BA.5) 0.05 MG/ML Injectable Suspension
or	procedure	UMLS:CPT:91313	Severe acute respiratory syndrome coronavirus 2 (SARS-CoV-2) (coronavirus disease [COVID-19]) vaccine, mRNA-

			LNP, spike protein, bivalent, preservative free, 50 mcg/0.5 mL dosage, for intramuscular use
or	procedure	UMLS:CPT:0134A	Immunization administration by intramuscular injection of severe acute respiratory syndrome coronavirus 2 (SARS-CoV-2) (coronavirus disease [COVID-19]) vaccine, mRNA-LNP, spike protein, bivalent, preservative free, 50 mcg/0.5 mL dosage, booster dose
or	procedure	UMLS:CPT:1037175	Immunization administration by intramuscular injection of severe acute respiratory syndrome coronavirus 2 (SARS-CoV-2) (coronavirus disease [COVID-19]) vaccine, DNA, spike protein, adenovirus type 26 (Ad26) vector, preservative free, 5x1010 viral particles/0.5 mL dosage
or	medication	NLM:RXNORM:2610347	0.3 ML SARS-CoV-2 (COVID-19) vaccine, mRNA-BNT162b2 0.05 MG/ML / SARS-CoV-2 (COVID-19) vaccine, mRNA-BNT162b2 OMICRON (BA.4/BA.5) -1 MG/ML Injection
or	procedure	UMLS:CPT:1037228	Immunization administration by intramuscular injection of severe acute respiratory syndrome coronavirus 2 (SARS-CoV-2) (coronavirus disease [COVID-19]) vaccine, mRNA-LNP, spike protein, preservative free, 3 mcg/0.2 mL dosage, diluent reconstituted, tris-sucrose formulation

or	procedure	UMLS:CPT:0013A	Immunization administration by intramuscular injection of severe acute respiratory syndrome coronavirus 2 (SARS-CoV-2) (coronavirus disease [COVID-19]) vaccine, mRNA-LNP, spike protein, preservative free, 100 mcg/0.5 mL dosage; third dose
or	procedure	UMLS:CPT:0081A	Immunization administration by intramuscular injection of severe acute respiratory syndrome coronavirus 2 (SARS-CoV-2) (coronavirus disease [COVID-19]) vaccine, mRNA-LNP, spike protein, preservative free, 3 mcg/0.2 mL dosage, diluent reconstituted, tris-sucrose formulation; first dose
or	procedure	UMLS:CPT:0082A	Immunization administration by intramuscular injection of severe acute respiratory syndrome coronavirus 2 (SARS-CoV-2) (coronavirus disease [COVID-19]) vaccine, mRNA-LNP, spike protein, preservative free, 3 mcg/0.2 mL dosage, diluent reconstituted, tris-sucrose formulation; second dose
or	medication	NLM:RXNORM:2610328	SARS-CoV-2 (COVID-19) vaccine, mRNA-1273 0.05 MG/ML / SARS-CoV-2 (COVID-19) vaccine, mRNA-1273 OMICRON (BA.4/BA.5) 0.05 MG/ML Injectable Suspension
or	procedure	UMLS:CPT:0154A	Immunization administration by intramuscular injection of

			severe acute respiratory syndrome coronavirus 2 (SARS-CoV-2) (coronavirus disease [COVID-19]) vaccine, mRNA-LNP, bivalent spike protein, preservative free, 10 mcg/0.2 mL dosage, diluent reconstituted, tris-sucrose formulation, booster dose
or	procedure	UMLS:CPT:0053A	Immunization administration by intramuscular injection of severe acute respiratory syndrome coronavirus 2 (SARS-CoV-2) (coronavirus disease [COVID-19]) vaccine, mRNA-LNP, spike protein, preservative free, 30 mcg/0.3 mL dosage, tris-sucrose formulation; third dose
or	procedure	UMLS:CPT:1037332	Immunization administration by intramuscular injection of severe acute respiratory syndrome coronavirus 2 (SARS-CoV-2) (coronavirus disease [COVID-19]) vaccine, mRNA-LNP, spike protein, preservative free, 25 mcg/0.25 mL dosage
or	procedure	UMLS:CPT:0052A	Immunization administration by intramuscular injection of severe acute respiratory syndrome coronavirus 2 (SARS-CoV-2) (coronavirus disease [COVID-19]) vaccine, mRNA-LNP, spike protein, preservative free, 30 mcg/0.3 mL dosage, tris-sucrose formulation; second dose

or	procedure	UMLS:CPT:0111A	Immunization administration by intramuscular injection of severe acute respiratory syndrome coronavirus 2 (SARS-CoV-2) (coronavirus disease [COVID-19]) vaccine, mRNA-LNP, spike protein, preservative free, 25 mcg/0.25 mL dosage; first dose
or	procedure	UMLS:CPT:0051A	Immunization administration by intramuscular injection of severe acute respiratory syndrome coronavirus 2 (SARS-CoV-2) (coronavirus disease [COVID-19]) vaccine, mRNA-LNP, spike protein, preservative free, 30 mcg/0.3 mL dosage, tris-sucrose formulation; first dose
or	procedure	UMLS:CPT:91311	Severe acute respiratory syndrome coronavirus 2 (SARS-CoV-2) (coronavirus disease [COVID-19]) vaccine, mRNA-LNP, spike protein, preservative free, 25 mcg/0.25 mL dosage, for intramuscular use
or	procedure	UMLS:CPT:0074A	Immunization administration by intramuscular injection of severe acute respiratory syndrome coronavirus 2 (SARS-CoV-2) (coronavirus disease [COVID-19]) vaccine, mRNA-LNP, spike protein, preservative free, 10 mcg/0.2 mL dosage, diluent reconstituted, tris-sucrose formulation; booster dose

or	procedure	UMLS:CPT:0112A	Immunization administration by intramuscular injection of severe acute respiratory syndrome coronavirus 2 (SARS-CoV-2) (coronavirus disease [COVID-19]) vaccine, mRNA-LNP, spike protein, preservative free, 25 mcg/0.25 mL dosage; second dose
or	procedure	UMLS:CPT:0083A	Immunization administration by intramuscular injection of severe acute respiratory syndrome coronavirus 2 (SARS-CoV-2) (coronavirus disease [COVID-19]) vaccine, mRNA-LNP, spike protein, preservative free, 3 mcg/0.2 mL dosage, diluent reconstituted, tris-sucrose formulation; third dose
or	procedure	UMLS:CPT:0073A	Immunization administration by intramuscular injection of severe acute respiratory syndrome coronavirus 2 (SARS-CoV-2) (coronavirus disease [COVID-19]) vaccine, mRNA-LNP, spike protein, preservative free, 10 mcg/0.2 mL dosage, diluent reconstituted, tris-sucrose formulation; third dose
or	procedure	UMLS:CPT:0173A	Immunization administration by intramuscular injection of severe acute respiratory syndrome coronavirus 2 (SARS-CoV-2) (coronavirus disease [COVID-19]) vaccine, mRNA-LNP, bivalent spike protein, preservative free, 3 mcg/0.2 mL dosage, diluent reconstituted,

			tris-sucrose formulation, third dose
or	procedure	UMLS:CPT:0164A	Immunization administration by intramuscular injection of severe acute respiratory syndrome coronavirus 2 (SARS-CoV-2) (coronavirus disease [COVID-19]) vaccine, mRNA-LNP, spike protein, bivalent, preservative free, 10 mcg/0.2 mL dosage, booster dose
or	procedure	UMLS:CPT:1037838	Immunization administration by intramuscular injection of severe acute respiratory syndrome coronavirus 2 (SARS-CoV-2) (coronavirus disease [COVID-19]) vaccine, mRNA-LNP, spike protein, preservative free, 50 mcg/0.5 mL dosage
or	procedure	UMLS:CPT:0094A	Immunization administration by intramuscular injection of severe acute respiratory syndrome coronavirus 2 (SARS-CoV-2) (coronavirus disease [COVID-19]) vaccine, mRNA-LNP, spike protein, preservative free, 50 mcg/0.5 mL dosage; booster dose, when administered to individuals 18 years and over
or	procedure	UMLS:CPT:0034A	Immunization administration by intramuscular injection of severe acute respiratory syndrome coronavirus 2 (SARS-CoV-2) (coronavirus disease [COVID-19]) vaccine, DNA, spike protein, adenovirus type 26 (Ad26) vector, preservative free,

			5x1010 viral particles/0.5 mL dosage; booster dose
or	procedure	UMLS:CPT:0144A	Immunization administration by intramuscular injection of severe acute respiratory syndrome coronavirus 2 (SARS-CoV-2) (coronavirus disease [COVID-19]) vaccine, mRNA-LNP, spike protein, bivalent, preservative free, 25 mcg/0.25 mL dosage, booster dose
or	procedure	UMLS:CPT:0091A	Immunization administration by intramuscular injection of severe acute respiratory syndrome coronavirus 2 (SARS-CoV-2) (coronavirus disease [COVID-19]) vaccine, mRNA-LNP, spike protein, preservative free, 50 mcg/0.5 mL dosage; first dose, when administered to individuals 6 through 11 years
or	procedure	UMLS:CPT:0174A	Immunization administration by intramuscular injection of severe acute respiratory syndrome coronavirus 2 (SARS-CoV-2) (coronavirus disease [COVID-19]) vaccine, mRNA-LNP, bivalent spike protein, preservative free, 3 mcg/0.2 mL dosage, diluent reconstituted, tris-sucrose formulation, booster
or	procedure	UMLS:CPT:0092A	Immunization administration by intramuscular injection of severe acute respiratory syndrome coronavirus 2 (SARS-CoV-2) (coronavirus disease [COVID-19]) vaccine, mRNA-

			LNP, spike protein, preservative free, 50 mcg/0.5 mL dosage; second dose, when administered to individuals 6 through 11 years
or	procedure	UMLS:CPT:1036682	Immunization administration by intramuscular injection of severe acute respiratory syndrome coronavirus 2 (SARS-CoV-2) (coronavirus disease [COVID-19]) vaccine, recombinant spike protein nanoparticle, saponin-based adjuvant, preservative free, 5 mcg/0.5 mL dosage
or	procedure	UMLS:CPT:0041A	Immunization administration by intramuscular injection of severe acute respiratory syndrome coronavirus 2 (SARS-CoV-2) (coronavirus disease [COVID-19]) vaccine, recombinant spike protein nanoparticle, saponin-based adjuvant, preservative free, 5 mcg/0.5 mL dosage; first dose
or	procedure	UMLS:CPT:0113A	Immunization administration by intramuscular injection of severe acute respiratory syndrome coronavirus 2 (SARS-CoV-2) (coronavirus disease [COVID-19]) vaccine, mRNA-LNP, spike protein, preservative free, 25 mcg/0.25 mL dosage; third dose
or	procedure	UMLS:CPT:0042A	Immunization administration by intramuscular injection of severe acute respiratory syndrome coronavirus 2 (SARS-

				CoV-2) (coronavirus disease [COVID-19]) vaccine, recombinant spike protein nanoparticle, saponin-based adjuvant, preservative free, 5 mcg/0.5 mL dosage; second dose
	or	procedure	UMLS:CPT:0093A	Immunization administration by intramuscular injection of severe acute respiratory syndrome coronavirus 2 (SARS-CoV-2) (coronavirus disease [COVID-19]) vaccine, mRNA-LNP, spike protein, preservative free, 50 mcg/0.5 mL dosage; third dose, when administered to individuals 6 through 11 years
	or	procedure	UMLS:CPT:1036666	Immunization administration by intramuscular injection of severe acute respiratory syndrome coronavirus 2 (SARS-CoV-2) (coronavirus disease [COVID-19]) vaccine, DNA, spike protein, chimpanzee adenovirus Oxford 1 (ChAdOx1) vector, preservative free, 5x1010 viral particles/0.5 mL dosage
	or	procedure	UMLS:CPT:0044A	Immunization administration by intramuscular injection of severe acute respiratory syndrome coronavirus 2 (SARS-CoV-2) (coronavirus disease [COVID-19]) vaccine, recombinant spike protein nanoparticle, saponin-based adjuvant, preservative free, 5 mcg/0.5mL dosage; booster
date constraint		The terms in this group occurred between Dec 1, 2019 and Dec 31, 2023		

Group 2				
**Group 2A COVID-19 negative**				
must have		visit	TNX:Visit	Visit (Data Source: TriNetX)
cannot have		diagnosis	UMLS:ICD10CM:U07.1	COVID-19
	or	laboratory	UMLS:LNC:95406-5	SARS-CoV-2 (COVID-19) RNA [Presence] in Nose by NAA with probe detection (labResult: Positive)
	or	laboratory	UMLS:LNC:94845-5	SARS-CoV-2 (COVID-19) RNA [Presence] in Saliva (oral fluid) by NAA with probe detection (labResult: Positive)
	or	laboratory	UMLS:LNC:95608-6	SARS-CoV-2 (COVID-19) RNA [Presence] in Respiratory specimen by NAA with non-probe detection (labResult: Positive)
	or	laboratory	UMLS:LNC:94759-8	SARS-CoV-2 (COVID-19) RNA [Presence] in Nasopharynx by NAA with probe detection (labResult: Positive)
	or	laboratory	UMLS:LNC:94565-9	SARS-CoV-2 (COVID-19) RNA [Presence] in Nasopharynx by NAA with non-probe detection (labResult: Positive)
	or	laboratory	UMLS:LNC:94309-2	SARS-CoV-2 (COVID-19) RNA [Presence] in Specimen by NAA with probe detection (labResult: Positive)
	or	laboratory	UMLS:LNC:94500-6	SARS-CoV-2 (COVID-19) RNA [Presence] in Respiratory specimen by NAA with probe detection (labResult: Positive)
	or	diagnosis	UMLS:ICD10CM:U07.2	COVID-19, virus not identified (WHO)
	or	diagnosis	UMLS:ICD10CM:J12.82	Pneumonia due to coronavirus disease 2019

date constraint		The terms in this group occurred at any time		
event relationship		Any instance of thyroid cancer history occurred at least 1 day before the first instance of COVID-19 negative		
**Group 2B thyroid cancer history**				
cannot have		diagnosis	UMLS:ICD10CM:C73	Malignant neoplasm of thyroid gland
	or	diagnosis	UMLS:ICD10CM:Z85.850	Personal history of malignant neoplasm of thyroid

Query Criteria for Cohort post-COVID, 18-40

This query was run on the network Global Collaborative Network with 148 HCO(s) queried and 148 HCO(s) responded. A total of 122 provider(s) responded with patients. The final cohort included 799,295 patients who matched the query criteria listed in the table below.

**Ungrouped terms**				
must have		demographics	Age	Age (between 18 and 40 years (most recent occurrence))
	and any of	demographics	UMLS:HL7V3.0:Gender:M	Male
		demographics	UMLS:HL7V3.0:Gender:F	Female
**Group 1**				
**COVID-19, no vaccination**				
must have	any of	diagnosis	UMLS:ICD10CM:U07.1	COVID-19
		diagnosis	UMLS:ICD10CM:U07.2	COVID-19, virus not identified (WHO)
		diagnosis	UMLS:ICD10CM:J12.82	Pneumonia due to coronavirus disease 2019
		laboratory	UMLS:LNC:94500-6	SARS-CoV-2 (COVID-19) RNA [Presence] in Respiratory specimen by NAA with probe detection (labResult: Positive)
		laboratory	UMLS:LNC:94309-2	SARS-CoV-2 (COVID-19) RNA [Presence] in Specimen by NAA with probe detection (labResult: Positive)
		laboratory	UMLS:LNC:94565-9	SARS-CoV-2 (COVID-19) RNA [Presence] in Nasopharynx by

				NAA with non-probe detection (labResult: Positive)
		laboratory	UMLS:LNC:94759-8	SARS-CoV-2 (COVID-19) RNA [Presence] in Nasopharynx by NAA with probe detection (labResult: Positive)
		laboratory	UMLS:LNC:95608-6	SARS-CoV-2 (COVID-19) RNA [Presence] in Respiratory specimen by NAA with non-probe detection (labResult: Positive)
		laboratory	UMLS:LNC:94845-5	SARS-CoV-2 (COVID-19) RNA [Presence] in Saliva (oral fluid) by NAA with probe detection (labResult: Positive)
		laboratory	UMLS:LNC:95406-5	SARS-CoV-2 (COVID-19) RNA [Presence] in Nose by NAA with probe detection (labResult: Positive)
cannot have		medication	NLM:CVX:208	COVID-19, mRNA, LNP-S, PF, 30 mcg/0.3 mL dose
	or	medication	NLM:CVX:207	COVID-19, mRNA, LNP-S, PF, 100 mcg/0.5mL dose or 50 mcg/0.25mL dose
	or	medication	NLM:CVX:212	COVID-19 vaccine, vector-nr, rS-Ad26, PF, 0.5 mL
	or	medication	NLM:RXNORM:OMOP 5042939	COVID-19 vaccine
	or	medication	NLM:CVX:300	COVID-19, mRNA, LNP-S, bivalent, PF, 30 mcg/0.3 mL dose
	or	medication	NLM:CVX:217	COVID-19, mRNA, LNP-S, PF, 30 mcg/0.3 mL dose, tris-sucrose
	or	medication	NLM:CVX:229	COVID-19, mRNA, LNP-S, bivalent, PF, 50 mcg/0.5 mL or 25mcg/0.25 mL dose
	or	medication	NLM:CVX:218	COVID-19, mRNA, LNP-S, PF, 10 mcg/0.2 mL dose, tris-sucrose

or	medication	NLM:CVX:520	COVID-19 mRNA, bivalent, original/Omicron BA.1, Non-US Vaccine Product, Pfizer-BioNTech
or	medication	NLM:CVX:519	COVID-19 mRNA, bivalent, original/Omicron BA.1, Non-US Vaccine (Spikevax Bivalent), Moderna
or	medication	NLM:CVX:301	COVID-19, mRNA, LNP-S, bivalent, PF, 10 mcg/0.2 mL dose
or	medication	NLM:CVX:219	COVID-19, mRNA, LNP-S, PF, 3 mcg/0.2 mL dose, tris-sucrose
or	medication	NLM:CVX:228	COVID-19, mRNA, LNP-S, PF, pediatric 25 mcg/0.25 mL dose
or	medication	NLM:CVX:230	COVID-19, mRNA, LNP-S, bivalent booster, PF, 10 mcg/0.2 mL
or	medication	NLM:CVX:221	COVID-19, mRNA, LNP-S, PF, 50 mcg/0.5 mL dose
or	medication	NLM:CVX:210	COVID-19 vaccine, vector-nr, rS-ChAdOx1, PF, 0.5 mL
or	medication	NLM:CVX:302	COVID-19, mRNA, LNP-S, bivalent, PF, 3 mcg/0.2 mL dose
or	medication	NLM:CVX:511	COVID-19 IV Non-US Vaccine (CoronaVac, Sinovac)
or	medication	NLM:RXNORM:2468231	SARS-CoV-2 (COVID-19) vaccine, mRNA spike protein
or	procedure	UMLS:CPT:91300	Severe acute respiratory syndrome coronavirus 2 (SARS-CoV-2) (coronavirus disease [COVID-19]) vaccine, mRNA-LNP, spike protein, preservative free, 30 mcg/0.3 mL dosage, diluent reconstituted, for intramuscular use
or	procedure	UMLS:CPT:0001A	Immunization administration by intramuscular injection of

			severe acute respiratory syndrome coronavirus 2 (SARS-CoV-2) (coronavirus disease [COVID-19]) vaccine, mRNA-LNP, spike protein, preservative free, 30 mcg/0.3 mL dosage, diluent reconstituted; first dose
or	procedure	UMLS:CPT:0002A	Immunization administration by intramuscular injection of severe acute respiratory syndrome coronavirus 2 (SARS-CoV-2) (coronavirus disease [COVID-19]) vaccine, mRNA-LNP, spike protein, preservative free, 30 mcg/0.3 mL dosage, diluent reconstituted; second dose
or	procedure	UMLS:CPT:91301	Severe acute respiratory syndrome coronavirus 2 (SARS-CoV-2) (coronavirus disease [COVID-19]) vaccine, mRNA-LNP, spike protein, preservative free, 100 mcg/0.5 mL dosage, for intramuscular use
or	procedure	UMLS:CPT:0011A	Immunization administration by intramuscular injection of severe acute respiratory syndrome coronavirus 2 (SARS-CoV-2) (coronavirus disease [COVID-19]) vaccine, mRNA-LNP, spike protein, preservative free, 100 mcg/0.5 mL dosage; first dose
or	procedure	UMLS:CPT:0012A	Immunization administration by intramuscular injection of severe acute respiratory syndrome coronavirus 2 (SARS-CoV-2) (coronavirus disease

			[COVID-19]) vaccine, mRNA-LNP, spike protein, preservative free, 100 mcg/0.5 mL dosage; second dose
or	procedure	UMLS:SNOMED:840534001	Administration of SARS-CoV-2 antigen vaccine
or	medication	NLM:CVX:213	SARS-CoV-2 (COVID-19) Vaccine
or	procedure	UMLS:CPT:1036660	Immunization administration by intramuscular injection of severe acute respiratory syndrome coronavirus 2 (SARS-CoV-2) (coronavirus disease [COVID-19]) vaccine, mRNA-LNP, spike protein, preservative free, 30 mcg/0.3 mL dosage, diluent reconstituted
or	procedure	UMLS:CPT:1036663	Immunization administration by intramuscular injection of severe acute respiratory syndrome coronavirus 2 (SARS-CoV-2) (coronavirus disease [COVID-19]) vaccine, mRNA-LNP, spike protein, preservative free, 100 mcg/0.5 mL dosage
or	procedure	UMLS:CPT:0124A	Immunization administration by intramuscular injection of severe acute respiratory syndrome coronavirus 2 (SARS-CoV-2) (coronavirus disease [COVID-19]) vaccine, mRNA-LNP, bivalent spike protein, preservative free, 30 mcg/0.3 mL dosage, tris-sucrose formulation, booster dose
or	procedure	UMLS:CPT:0004A	Immunization administration by intramuscular injection of severe acute respiratory

			syndrome coronavirus 2 (SARS-CoV-2) (coronavirus disease [COVID-19]) vaccine, mRNA-LNP, spike protein, preservative free, 30 mcg/0.3 mL dosage, diluent reconstituted; booster dose
or	procedure	UMLS:CPT:0003A	Immunization administration by intramuscular injection of severe acute respiratory syndrome coronavirus 2 (SARS-CoV-2) (coronavirus disease [COVID-19]) vaccine, mRNA-LNP, spike protein, preservative free, 30 mcg/0.3 mL dosage, diluent reconstituted; third dose
or	procedure	UMLS:CPT:1037166	Immunization administration by intramuscular injection of severe acute respiratory syndrome coronavirus 2 (SARS-CoV-2) (coronavirus disease [COVID-19]) vaccine, mRNA-LNP, spike protein, preservative free, 30 mcg/0.3 mL dosage, tris-sucrose formulation
or	procedure	UMLS:CPT:0054A	Immunization administration by intramuscular injection of severe acute respiratory syndrome coronavirus 2 (SARS-CoV-2) (coronavirus disease [COVID-19]) vaccine, mRNA-LNP, spike protein, preservative free, 30 mcg/0.3 mL dosage, tris-sucrose formulation; booster dose
or	procedure	UMLS:CPT:0064A	Immunization administration by intramuscular injection of severe acute respiratory

			syndrome coronavirus 2 (SARS-CoV-2) (coronavirus disease [COVID-19]) vaccine, mRNA-LNP, spike protein, preservative free, 50 mcg/0.25 mL dosage, booster dose
or	procedure	UMLS:CPT:90480	Immunization administration by intramuscular injection of severe acute respiratory syndrome coronavirus 2 (SARS-CoV-2) (coronavirus disease [COVID-19]) vaccine, single dose
or	procedure	UMLS:CPT:1037171	Immunization administration by intramuscular injection of severe acute respiratory syndrome coronavirus 2 (SARS-CoV-2) (coronavirus disease [COVID-19]) vaccine, mRNA-LNP, spike protein, preservative free, 10 mcg/0.2 mL dosage, diluent reconstituted, tris-sucrose formulation
or	procedure	UMLS:CPT:0071A	Immunization administration by intramuscular injection of severe acute respiratory syndrome coronavirus 2 (SARS-CoV-2) (coronavirus disease [COVID-19]) vaccine, mRNA-LNP, spike protein, preservative free, 10 mcg/0.2 mL dosage, diluent reconstituted, tris-sucrose formulation; first dose
or	procedure	UMLS:CPT:0072A	Immunization administration by intramuscular injection of severe acute respiratory syndrome coronavirus 2 (SARS-CoV-2) (coronavirus disease

			[COVID-19]) vaccine, mRNA-LNP, spike protein, preservative free, 10 mcg/0.2 mL dosage, diluent reconstituted, tris-sucrose formulation; second dose
or	medication	NLM:RXNORM:2610319	SARS-CoV-2 (COVID-19) vaccine, mRNA-BNT162b2 0.05 MG/ML / SARS-CoV-2 (COVID-19) vaccine, mRNA-BNT162b2 OMICRON (BA.4/BA.5) 0.05 MG/ML Injectable Suspension
or	procedure	UMLS:CPT:91313	Severe acute respiratory syndrome coronavirus 2 (SARS-CoV-2) (coronavirus disease [COVID-19]) vaccine, mRNA-LNP, spike protein, bivalent, preservative free, 50 mcg/0.5 mL dosage, for intramuscular use
or	procedure	UMLS:CPT:0134A	Immunization administration by intramuscular injection of severe acute respiratory syndrome coronavirus 2 (SARS-CoV-2) (coronavirus disease [COVID-19]) vaccine, mRNA-LNP, spike protein, bivalent, preservative free, 50 mcg/0.5 mL dosage, booster dose
or	procedure	UMLS:CPT:1037175	Immunization administration by intramuscular injection of severe acute respiratory syndrome coronavirus 2 (SARS-CoV-2) (coronavirus disease [COVID-19]) vaccine, DNA, spike protein, adenovirus type 26 (Ad26) vector, preservative free, 5x1010 viral particles/0.5 mL dosage

or	medication	NLM:RXNORM:2610347	0.3 ML SARS-CoV-2 (COVID-19) vaccine, mRNA-BNT162b2 0.05 MG/ML / SARS-CoV-2 (COVID-19) vaccine, mRNA-BNT162b2 OMICRON (BA.4/BA.5) -1 MG/ML Injection
or	procedure	UMLS:CPT:1037228	Immunization administration by intramuscular injection of severe acute respiratory syndrome coronavirus 2 (SARS-CoV-2) (coronavirus disease [COVID-19]) vaccine, mRNA-LNP, spike protein, preservative free, 3 mcg/0.2 mL dosage, diluent reconstituted, tris-sucrose formulation
or	procedure	UMLS:CPT:0013A	Immunization administration by intramuscular injection of severe acute respiratory syndrome coronavirus 2 (SARS-CoV-2) (coronavirus disease [COVID-19]) vaccine, mRNA-LNP, spike protein, preservative free, 100 mcg/0.5 mL dosage; third dose
or	procedure	UMLS:CPT:0081A	Immunization administration by intramuscular injection of severe acute respiratory syndrome coronavirus 2 (SARS-CoV-2) (coronavirus disease [COVID-19]) vaccine, mRNA-LNP, spike protein, preservative free, 3 mcg/0.2 mL dosage, diluent reconstituted, tris-sucrose formulation; first dose
or	procedure	UMLS:CPT:0082A	Immunization administration by intramuscular injection of severe acute respiratory

			syndrome coronavirus 2 (SARS-CoV-2) (coronavirus disease [COVID-19]) vaccine, mRNA-LNP, spike protein, preservative free, 3 mcg/0.2 mL dosage, diluent reconstituted, tris-sucrose formulation; second dose
or	medication	NLM:RXNORM:2610328	SARS-CoV-2 (COVID-19) vaccine, mRNA-1273 0.05 MG/ML / SARS-CoV-2 (COVID-19) vaccine, mRNA-1273 OMICRON (BA.4/BA.5) 0.05 MG/ML Injectable Suspension
or	procedure	UMLS:CPT:0154A	Immunization administration by intramuscular injection of severe acute respiratory syndrome coronavirus 2 (SARS-CoV-2) (coronavirus disease [COVID-19]) vaccine, mRNA-LNP, bivalent spike protein, preservative free, 10 mcg/0.2 mL dosage, diluent reconstituted, tris-sucrose formulation, booster dose
or	procedure	UMLS:CPT:0053A	Immunization administration by intramuscular injection of severe acute respiratory syndrome coronavirus 2 (SARS-CoV-2) (coronavirus disease [COVID-19]) vaccine, mRNA-LNP, spike protein, preservative free, 30 mcg/0.3 mL dosage, tris-sucrose formulation; third dose
or	procedure	UMLS:CPT:1037332	Immunization administration by intramuscular injection of severe acute respiratory

			syndrome coronavirus 2 (SARS-CoV-2) (coronavirus disease [COVID-19]) vaccine, mRNA-LNP, spike protein, preservative free, 25 mcg/0.25 mL dosage
or	procedure	UMLS:CPT:0052A	Immunization administration by intramuscular injection of severe acute respiratory syndrome coronavirus 2 (SARS-CoV-2) (coronavirus disease [COVID-19]) vaccine, mRNA-LNP, spike protein, preservative free, 30 mcg/0.3 mL dosage, tris-sucrose formulation; second dose
or	procedure	UMLS:CPT:0111A	Immunization administration by intramuscular injection of severe acute respiratory syndrome coronavirus 2 (SARS-CoV-2) (coronavirus disease [COVID-19]) vaccine, mRNA-LNP, spike protein, preservative free, 25 mcg/0.25 mL dosage; first dose
or	procedure	UMLS:CPT:0051A	Immunization administration by intramuscular injection of severe acute respiratory syndrome coronavirus 2 (SARS-CoV-2) (coronavirus disease [COVID-19]) vaccine, mRNA-LNP, spike protein, preservative free, 30 mcg/0.3 mL dosage, tris-sucrose formulation; first dose
or	procedure	UMLS:CPT:91311	Severe acute respiratory syndrome coronavirus 2 (SARS-CoV-2) (coronavirus disease [COVID-19]) vaccine, mRNA-

			LNP, spike protein, preservative free, 25 mcg/0.25 mL dosage, for intramuscular use
or	procedure	UMLS:CPT:0074A	Immunization administration by intramuscular injection of severe acute respiratory syndrome coronavirus 2 (SARS-CoV-2) (coronavirus disease [COVID-19]) vaccine, mRNA-LNP, spike protein, preservative free, 10 mcg/0.2 mL dosage, diluent reconstituted, tris-sucrose formulation; booster dose
or	procedure	UMLS:CPT:0112A	Immunization administration by intramuscular injection of severe acute respiratory syndrome coronavirus 2 (SARS-CoV-2) (coronavirus disease [COVID-19]) vaccine, mRNA-LNP, spike protein, preservative free, 25 mcg/0.25 mL dosage; second dose
or	procedure	UMLS:CPT:0083A	Immunization administration by intramuscular injection of severe acute respiratory syndrome coronavirus 2 (SARS-CoV-2) (coronavirus disease [COVID-19]) vaccine, mRNA-LNP, spike protein, preservative free, 3 mcg/0.2 mL dosage, diluent reconstituted, tris-sucrose formulation; third dose
or	procedure	UMLS:CPT:0073A	Immunization administration by intramuscular injection of severe acute respiratory syndrome coronavirus 2 (SARS-CoV-2) (coronavirus disease

			[COVID-19]) vaccine, mRNA-LNP, spike protein, preservative free, 10 mcg/0.2 mL dosage, diluent reconstituted, tris-sucrose formulation; third dose
or	procedure	UMLS:CPT:0173A	Immunization administration by intramuscular injection of severe acute respiratory syndrome coronavirus 2 (SARS-CoV-2) (coronavirus disease [COVID-19]) vaccine, mRNA-LNP, bivalent spike protein, preservative free, 3 mcg/0.2 mL dosage, diluent reconstituted, tris-sucrose formulation, third dose
or	procedure	UMLS:CPT:0164A	Immunization administration by intramuscular injection of severe acute respiratory syndrome coronavirus 2 (SARS-CoV-2) (coronavirus disease [COVID-19]) vaccine, mRNA-LNP, spike protein, bivalent, preservative free, 10 mcg/0.2 mL dosage, booster dose
or	procedure	UMLS:CPT:1037838	Immunization administration by intramuscular injection of severe acute respiratory syndrome coronavirus 2 (SARS-CoV-2) (coronavirus disease [COVID-19]) vaccine, mRNA-LNP, spike protein, preservative free, 50 mcg/0.5 mL dosage
or	procedure	UMLS:CPT:0094A	Immunization administration by intramuscular injection of severe acute respiratory syndrome coronavirus 2 (SARS-CoV-2) (coronavirus disease

			[COVID-19]) vaccine, mRNA-LNP, spike protein, preservative free, 50 mcg/0.5 mL dosage; booster dose, when administered to individuals 18 years and over
or	procedure	UMLS:CPT:0034A	Immunization administration by intramuscular injection of severe acute respiratory syndrome coronavirus 2 (SARS-CoV-2) (coronavirus disease [COVID-19]) vaccine, DNA, spike protein, adenovirus type 26 (Ad26) vector, preservative free, 5x1010 viral particles/0.5 mL dosage; booster dose
or	procedure	UMLS:CPT:0144A	Immunization administration by intramuscular injection of severe acute respiratory syndrome coronavirus 2 (SARS-CoV-2) (coronavirus disease [COVID-19]) vaccine, mRNA-LNP, spike protein, bivalent, preservative free, 25 mcg/0.25 mL dosage, booster dose
or	procedure	UMLS:CPT:0091A	Immunization administration by intramuscular injection of severe acute respiratory syndrome coronavirus 2 (SARS-CoV-2) (coronavirus disease [COVID-19]) vaccine, mRNA-LNP, spike protein, preservative free, 50 mcg/0.5 mL dosage; first dose, when administered to individuals 6 through 11 years
or	procedure	UMLS:CPT:0174A	Immunization administration by intramuscular injection of severe acute respiratory

			syndrome coronavirus 2 (SARS-CoV-2) (coronavirus disease [COVID-19]) vaccine, mRNA-LNP, bivalent spike protein, preservative free, 3 mcg/0.2 mL dosage, diluent reconstituted, tris-sucrose formulation, booster
or	procedure	UMLS:CPT:0092A	Immunization administration by intramuscular injection of severe acute respiratory syndrome coronavirus 2 (SARS-CoV-2) (coronavirus disease [COVID-19]) vaccine, mRNA-LNP, spike protein, preservative free, 50 mcg/0.5 mL dosage; second dose, when administered to individuals 6 through 11 years
or	procedure	UMLS:CPT:1036682	Immunization administration by intramuscular injection of severe acute respiratory syndrome coronavirus 2 (SARS-CoV-2) (coronavirus disease [COVID-19]) vaccine, recombinant spike protein nanoparticle, saponin-based adjuvant, preservative free, 5 mcg/0.5 mL dosage
or	procedure	UMLS:CPT:0041A	Immunization administration by intramuscular injection of severe acute respiratory syndrome coronavirus 2 (SARS-CoV-2) (coronavirus disease [COVID-19]) vaccine, recombinant spike protein nanoparticle, saponin-based

			adjuvant, preservative free, 5 mcg/0.5 mL dosage; first dose
or	procedure	UMLS:CPT:0113A	Immunization administration by intramuscular injection of severe acute respiratory syndrome coronavirus 2 (SARS-CoV-2) (coronavirus disease [COVID-19]) vaccine, mRNA-LNP, spike protein, preservative free, 25 mcg/0.25 mL dosage; third dose
or	procedure	UMLS:CPT:0042A	Immunization administration by intramuscular injection of severe acute respiratory syndrome coronavirus 2 (SARS-CoV-2) (coronavirus disease [COVID-19]) vaccine, recombinant spike protein nanoparticle, saponin-based adjuvant, preservative free, 5 mcg/0.5 mL dosage; second dose
or	procedure	UMLS:CPT:0093A	Immunization administration by intramuscular injection of severe acute respiratory syndrome coronavirus 2 (SARS-CoV-2) (coronavirus disease [COVID-19]) vaccine, mRNA-LNP, spike protein, preservative free, 50 mcg/0.5 mL dosage; third dose, when administered to individuals 6 through 11 years
or	procedure	UMLS:CPT:1036666	Immunization administration by intramuscular injection of severe acute respiratory syndrome coronavirus 2 (SARS-CoV-2) (coronavirus disease [COVID-19]) vaccine, DNA, spike

				protein, chimpanzee adenovirus Oxford 1 (ChAdOx1) vector, preservative free, 5x1010 viral particles/0.5 mL dosage
	or	procedure	UMLS:CPT:0044A	Immunization administration by intramuscular injection of severe acute respiratory syndrome coronavirus 2 (SARS-CoV-2) (coronavirus disease [COVID-19]) vaccine, recombinant spike protein nanoparticle, saponin-based adjuvant, preservative free, 5 mcg/0.5mL dosage; booster
date constraint		The terms in this group occurred between Dec 1, 2019 and Dec 31, 2023		
**Group 2**				
**Group 2A COVID-19**				
must have	any of	diagnosis	UMLS:ICD10CM:U07.1	COVID-19
		diagnosis	UMLS:ICD10CM:U07.2	COVID-19, virus not identified (WHO)
		diagnosis	UMLS:ICD10CM:J12.82	Pneumonia due to coronavirus disease 2019
		laboratory	UMLS:LNC:94500-6	SARS-CoV-2 (COVID-19) RNA [Presence] in Respiratory specimen by NAA with probe detection (labResult: Positive)
		laboratory	UMLS:LNC:94309-2	SARS-CoV-2 (COVID-19) RNA [Presence] in Specimen by NAA with probe detection (labResult: Positive)
		laboratory	UMLS:LNC:94565-9	SARS-CoV-2 (COVID-19) RNA [Presence] in Nasopharynx by NAA with non-probe detection (labResult: Positive)
		laboratory	UMLS:LNC:94759-8	SARS-CoV-2 (COVID-19) RNA [Presence] in Nasopharynx by

				NAA with probe detection (labResult: Positive)
		laboratory	UMLS:LNC:95608-6	SARS-CoV-2 (COVID-19) RNA [Presence] in Respiratory specimen by NAA with non-probe detection (labResult: Positive)
		laboratory	UMLS:LNC:94845-5	SARS-CoV-2 (COVID-19) RNA [Presence] in Saliva (oral fluid) by NAA with probe detection (labResult: Positive)
		laboratory	UMLS:LNC:95406-5	SARS-CoV-2 (COVID-19) RNA [Presence] in Nose by NAA with probe detection (labResult: Positive)
	and	visit	TNX:Visit	Visit (Data Source: TriNetX)
date constraint		The terms in this group occurred at any time		
event relationship		Any instance of thyroid cancer history occurred at least 1 day before the first instance of COVID-19		
**Group 2B thyroid cancer history**				
cannot have		diagnosis	UMLS:ICD10CM:C73	Malignant neoplasm of thyroid gland
	or	diagnosis	UMLS:ICD10CM:Z85.850	Personal history of malignant neoplasm of thyroid

Query Criteria for Cohort non-COVID, 18-40

This query was run on the network Global Collaborative Network with 148 HCO(s) queried and 148 HCO(s) responded. A total of 131 provider(s) responded with patients. The final cohort included 10,161,157 patients who matched the query criteria listed in the table below.

**Ungrouped terms**				
must have		demographics	Age	Age (between 18 and 40 years (most recent occurrence))
	and any of	demographics	UMLS:HL7V3.0:Gender:M	Male
		demographics	UMLS:HL7V3.0:Gender:F	Female
**Group 1**				

COVID-19 negative, no vaccination				
must have		visit	TNX:Visit	Visit
cannot have		medication	NLM:CVX:208	COVID-19, mRNA, LNP-S, PF, 30 mcg/0.3 mL dose
	or	medication	NLM:CVX:207	COVID-19, mRNA, LNP-S, PF, 100 mcg/0.5mL dose or 50 mcg/0.25mL dose
	or	medication	NLM:CVX:212	COVID-19 vaccine, vector-nr, rS-Ad26, PF, 0.5 mL
	or	medication	NLM:RXNORM:OMOP 5042939	COVID-19 vaccine
	or	medication	NLM:CVX:300	COVID-19, mRNA, LNP-S, bivalent, PF, 30 mcg/0.3 mL dose
	or	medication	NLM:CVX:217	COVID-19, mRNA, LNP-S, PF, 30 mcg/0.3 mL dose, tris-sucrose
	or	medication	NLM:CVX:229	COVID-19, mRNA, LNP-S, bivalent, PF, 50 mcg/0.5 mL or 25mcg/0.25 mL dose
	or	medication	NLM:CVX:218	COVID-19, mRNA, LNP-S, PF, 10 mcg/0.2 mL dose, tris-sucrose
	or	medication	NLM:CVX:520	COVID-19 mRNA, bivalent, original/Omicron BA.1, Non-US Vaccine Product, Pfizer-BioNTech
	or	medication	NLM:CVX:519	COVID-19 mRNA, bivalent, original/Omicron BA.1, Non-US Vaccine (Spikevax Bivalent), Moderna
	or	medication	NLM:CVX:301	COVID-19, mRNA, LNP-S, bivalent, PF, 10 mcg/0.2 mL dose
	or	medication	NLM:CVX:219	COVID-19, mRNA, LNP-S, PF, 3 mcg/0.2 mL dose, tris-sucrose
	or	medication	NLM:CVX:228	COVID-19, mRNA, LNP-S, PF, pediatric 25 mcg/0.25 mL dose

or	medication	NLM:CVX:230	COVID-19, mRNA, LNP-S, bivalent booster, PF, 10 mcg/0.2 mL
or	medication	NLM:CVX:221	COVID-19, mRNA, LNP-S, PF, 50 mcg/0.5 mL dose
or	medication	NLM:CVX:210	COVID-19 vaccine, vector-nr, rS-ChAdOx1, PF, 0.5 mL
or	medication	NLM:CVX:302	COVID-19, mRNA, LNP-S, bivalent, PF, 3 mcg/0.2 mL dose
or	medication	NLM:CVX:511	COVID-19 IV Non-US Vaccine (CoronaVac, Sinovac)
or	medication	NLM:RXNORM:2468231	SARS-CoV-2 (COVID-19) vaccine, mRNA spike protein
or	procedure	UMLS:CPT:91300	Severe acute respiratory syndrome coronavirus 2 (SARS-CoV-2) (coronavirus disease [COVID-19]) vaccine, mRNA-LNP, spike protein, preservative free, 30 mcg/0.3 mL dosage, diluent reconstituted, for intramuscular use
or	procedure	UMLS:CPT:0001A	Immunization administration by intramuscular injection of severe acute respiratory syndrome coronavirus 2 (SARS-CoV-2) (coronavirus disease [COVID-19]) vaccine, mRNA-LNP, spike protein, preservative free, 30 mcg/0.3 mL dosage, diluent reconstituted; first dose
or	procedure	UMLS:CPT:0002A	Immunization administration by intramuscular injection of severe acute respiratory syndrome coronavirus 2 (SARS-CoV-2) (coronavirus disease [COVID-19]) vaccine, mRNA-LNP, spike protein, preservative free, 30 mcg/0.3 mL dosage,

			diluent reconstituted; second dose
or	procedure	UMLS:CPT:91301	Severe acute respiratory syndrome coronavirus 2 (SARS-CoV-2) (coronavirus disease [COVID-19]) vaccine, mRNA-LNP, spike protein, preservative free, 100 mcg/0.5 mL dosage, for intramuscular use
or	procedure	UMLS:CPT:0011A	Immunization administration by intramuscular injection of severe acute respiratory syndrome coronavirus 2 (SARS-CoV-2) (coronavirus disease [COVID-19]) vaccine, mRNA-LNP, spike protein, preservative free, 100 mcg/0.5 mL dosage; first dose
or	procedure	UMLS:CPT:0012A	Immunization administration by intramuscular injection of severe acute respiratory syndrome coronavirus 2 (SARS-CoV-2) (coronavirus disease [COVID-19]) vaccine, mRNA-LNP, spike protein, preservative free, 100 mcg/0.5 mL dosage; second dose
or	procedure	UMLS:SNOMED:840534001	Administration of SARS-CoV-2 antigen vaccine
or	medication	NLM:CVX:213	SARS-CoV-2 (COVID-19) Vaccine
or	procedure	UMLS:CPT:1036660	Immunization administration by intramuscular injection of severe acute respiratory syndrome coronavirus 2 (SARS-CoV-2) (coronavirus disease [COVID-19]) vaccine, mRNA-LNP, spike protein, preservative

			free, 30 mcg/0.3 mL dosage, diluent reconstituted
or	procedure	UMLS:CPT:1036663	Immunization administration by intramuscular injection of severe acute respiratory syndrome coronavirus 2 (SARS-CoV-2) (coronavirus disease [COVID-19]) vaccine, mRNA-LNP, spike protein, preservative free, 100 mcg/0.5 mL dosage
or	procedure	UMLS:CPT:0124A	Immunization administration by intramuscular injection of severe acute respiratory syndrome coronavirus 2 (SARS-CoV-2) (coronavirus disease [COVID-19]) vaccine, mRNA-LNP, bivalent spike protein, preservative free, 30 mcg/0.3 mL dosage, tris-sucrose formulation, booster dose
or	procedure	UMLS:CPT:0004A	Immunization administration by intramuscular injection of severe acute respiratory syndrome coronavirus 2 (SARS-CoV-2) (coronavirus disease [COVID-19]) vaccine, mRNA-LNP, spike protein, preservative free, 30 mcg/0.3 mL dosage, diluent reconstituted; booster dose
or	procedure	UMLS:CPT:0003A	Immunization administration by intramuscular injection of severe acute respiratory syndrome coronavirus 2 (SARS-CoV-2) (coronavirus disease [COVID-19]) vaccine, mRNA-LNP, spike protein, preservative

			free, 30 mcg/0.3 mL dosage, diluent reconstituted; third dose
or	procedure	UMLS:CPT:1037166	Immunization administration by intramuscular injection of severe acute respiratory syndrome coronavirus 2 (SARS-CoV-2) (coronavirus disease [COVID-19]) vaccine, mRNA-LNP, spike protein, preservative free, 30 mcg/0.3 mL dosage, tris-sucrose formulation
or	procedure	UMLS:CPT:0054A	Immunization administration by intramuscular injection of severe acute respiratory syndrome coronavirus 2 (SARS-CoV-2) (coronavirus disease [COVID-19]) vaccine, mRNA-LNP, spike protein, preservative free, 30 mcg/0.3 mL dosage, tris-sucrose formulation; booster dose
or	procedure	UMLS:CPT:0064A	Immunization administration by intramuscular injection of severe acute respiratory syndrome coronavirus 2 (SARS-CoV-2) (coronavirus disease [COVID-19]) vaccine, mRNA-LNP, spike protein, preservative free, 50 mcg/0.25 mL dosage, booster dose
or	procedure	UMLS:CPT:90480	Immunization administration by intramuscular injection of severe acute respiratory syndrome coronavirus 2 (SARS-CoV-2) (coronavirus disease [COVID-19]) vaccine, single dose

or	procedure	UMLS:CPT:1037171	Immunization administration by intramuscular injection of severe acute respiratory syndrome coronavirus 2 (SARS-CoV-2) (coronavirus disease [COVID-19]) vaccine, mRNA-LNP, spike protein, preservative free, 10 mcg/0.2 mL dosage, diluent reconstituted, tris-sucrose formulation
or	procedure	UMLS:CPT:0071A	Immunization administration by intramuscular injection of severe acute respiratory syndrome coronavirus 2 (SARS-CoV-2) (coronavirus disease [COVID-19]) vaccine, mRNA-LNP, spike protein, preservative free, 10 mcg/0.2 mL dosage, diluent reconstituted, tris-sucrose formulation; first dose
or	procedure	UMLS:CPT:0072A	Immunization administration by intramuscular injection of severe acute respiratory syndrome coronavirus 2 (SARS-CoV-2) (coronavirus disease [COVID-19]) vaccine, mRNA-LNP, spike protein, preservative free, 10 mcg/0.2 mL dosage, diluent reconstituted, tris-sucrose formulation; second dose
or	medication	NLM:RXNORM:2610319	SARS-CoV-2 (COVID-19) vaccine, mRNA-BNT162b2 0.05 MG/ML / SARS-CoV-2 (COVID-19) vaccine, mRNA-BNT162b2 OMICRON (BA.4/BA.5) 0.05 MG/ML Injectable Suspension

or	procedure	UMLS:CPT:91313	Severe acute respiratory syndrome coronavirus 2 (SARS-CoV-2) (coronavirus disease [COVID-19]) vaccine, mRNA-LNP, spike protein, bivalent, preservative free, 50 mcg/0.5 mL dosage, for intramuscular use
or	procedure	UMLS:CPT:0134A	Immunization administration by intramuscular injection of severe acute respiratory syndrome coronavirus 2 (SARS-CoV-2) (coronavirus disease [COVID-19]) vaccine, mRNA-LNP, spike protein, bivalent, preservative free, 50 mcg/0.5 mL dosage, booster dose
or	procedure	UMLS:CPT:1037175	Immunization administration by intramuscular injection of severe acute respiratory syndrome coronavirus 2 (SARS-CoV-2) (coronavirus disease [COVID-19]) vaccine, DNA, spike protein, adenovirus type 26 (Ad26) vector, preservative free, 5x1010 viral particles/0.5 mL dosage
or	medication	NLM:RXNORM:2610347	0.3 ML SARS-CoV-2 (COVID-19) vaccine, mRNA-BNT162b2 0.05 MG/ML / SARS-CoV-2 (COVID-19) vaccine, mRNA-BNT162b2 OMICRON (BA.4/BA.5) -1 MG/ML Injection
or	procedure	UMLS:CPT:1037228	Immunization administration by intramuscular injection of severe acute respiratory syndrome coronavirus 2 (SARS-CoV-2) (coronavirus disease [COVID-19]) vaccine, mRNA-

			LNP, spike protein, preservative free, 3 mcg/0.2 mL dosage, diluent reconstituted, tris-sucrose formulation
or	procedure	UMLS:CPT:0013A	Immunization administration by intramuscular injection of severe acute respiratory syndrome coronavirus 2 (SARS-CoV-2) (coronavirus disease [COVID-19]) vaccine, mRNA-LNP, spike protein, preservative free, 100 mcg/0.5 mL dosage; third dose
or	procedure	UMLS:CPT:0081A	Immunization administration by intramuscular injection of severe acute respiratory syndrome coronavirus 2 (SARS-CoV-2) (coronavirus disease [COVID-19]) vaccine, mRNA-LNP, spike protein, preservative free, 3 mcg/0.2 mL dosage, diluent reconstituted, tris-sucrose formulation; first dose
or	procedure	UMLS:CPT:0082A	Immunization administration by intramuscular injection of severe acute respiratory syndrome coronavirus 2 (SARS-CoV-2) (coronavirus disease [COVID-19]) vaccine, mRNA-LNP, spike protein, preservative free, 3 mcg/0.2 mL dosage, diluent reconstituted, tris-sucrose formulation; second dose
or	medication	NLM:RXNORM:2610328	SARS-CoV-2 (COVID-19) vaccine, mRNA-1273 0.05 MG/ML / SARS-CoV-2 (COVID-19) vaccine, mRNA-1273

			OMICRON (BA.4/BA.5) 0.05 MG/ML Injectable Suspension
or	procedure	UMLS:CPT:0154A	Immunization administration by intramuscular injection of severe acute respiratory syndrome coronavirus 2 (SARS-CoV-2) (coronavirus disease [COVID-19]) vaccine, mRNA-LNP, bivalent spike protein, preservative free, 10 mcg/0.2 mL dosage, diluent reconstituted, tris-sucrose formulation, booster dose
or	procedure	UMLS:CPT:0053A	Immunization administration by intramuscular injection of severe acute respiratory syndrome coronavirus 2 (SARS-CoV-2) (coronavirus disease [COVID-19]) vaccine, mRNA-LNP, spike protein, preservative free, 30 mcg/0.3 mL dosage, tris-sucrose formulation; third dose
or	procedure	UMLS:CPT:1037332	Immunization administration by intramuscular injection of severe acute respiratory syndrome coronavirus 2 (SARS-CoV-2) (coronavirus disease [COVID-19]) vaccine, mRNA-LNP, spike protein, preservative free, 25 mcg/0.25 mL dosage
or	procedure	UMLS:CPT:0052A	Immunization administration by intramuscular injection of severe acute respiratory syndrome coronavirus 2 (SARS-CoV-2) (coronavirus disease [COVID-19]) vaccine, mRNA-LNP, spike protein, preservative

			free, 30 mcg/0.3 mL dosage, tris-sucrose formulation; second dose
or	procedure	UMLS:CPT:0111A	Immunization administration by intramuscular injection of severe acute respiratory syndrome coronavirus 2 (SARS-CoV-2) (coronavirus disease [COVID-19]) vaccine, mRNA-LNP, spike protein, preservative free, 25 mcg/0.25 mL dosage; first dose
or	procedure	UMLS:CPT:0051A	Immunization administration by intramuscular injection of severe acute respiratory syndrome coronavirus 2 (SARS-CoV-2) (coronavirus disease [COVID-19]) vaccine, mRNA-LNP, spike protein, preservative free, 30 mcg/0.3 mL dosage, tris-sucrose formulation; first dose
or	procedure	UMLS:CPT:91311	Severe acute respiratory syndrome coronavirus 2 (SARS-CoV-2) (coronavirus disease [COVID-19]) vaccine, mRNA-LNP, spike protein, preservative free, 25 mcg/0.25 mL dosage, for intramuscular use
or	procedure	UMLS:CPT:0074A	Immunization administration by intramuscular injection of severe acute respiratory syndrome coronavirus 2 (SARS-CoV-2) (coronavirus disease [COVID-19]) vaccine, mRNA-LNP, spike protein, preservative free, 10 mcg/0.2 mL dosage, diluent reconstituted, tris-

			sucrose formulation; booster dose
or	procedure	UMLS:CPT:0112A	Immunization administration by intramuscular injection of severe acute respiratory syndrome coronavirus 2 (SARS-CoV-2) (coronavirus disease [COVID-19]) vaccine, mRNA-LNP, spike protein, preservative free, 25 mcg/0.25 mL dosage; second dose
or	procedure	UMLS:CPT:0083A	Immunization administration by intramuscular injection of severe acute respiratory syndrome coronavirus 2 (SARS-CoV-2) (coronavirus disease [COVID-19]) vaccine, mRNA-LNP, spike protein, preservative free, 3 mcg/0.2 mL dosage, diluent reconstituted, tris-sucrose formulation; third dose
or	procedure	UMLS:CPT:0073A	Immunization administration by intramuscular injection of severe acute respiratory syndrome coronavirus 2 (SARS-CoV-2) (coronavirus disease [COVID-19]) vaccine, mRNA-LNP, spike protein, preservative free, 10 mcg/0.2 mL dosage, diluent reconstituted, tris-sucrose formulation; third dose
or	procedure	UMLS:CPT:0173A	Immunization administration by intramuscular injection of severe acute respiratory syndrome coronavirus 2 (SARS-CoV-2) (coronavirus disease [COVID-19]) vaccine, mRNA-LNP, bivalent spike protein,

			preservative free, 3 mcg/0.2 mL dosage, diluent reconstituted, tris-sucrose formulation, third dose
or	procedure	UMLS:CPT:0164A	Immunization administration by intramuscular injection of severe acute respiratory syndrome coronavirus 2 (SARS-CoV-2) (coronavirus disease [COVID-19]) vaccine, mRNA-LNP, spike protein, bivalent, preservative free, 10 mcg/0.2 mL dosage, booster dose
or	procedure	UMLS:CPT:1037838	Immunization administration by intramuscular injection of severe acute respiratory syndrome coronavirus 2 (SARS-CoV-2) (coronavirus disease [COVID-19]) vaccine, mRNA-LNP, spike protein, preservative free, 50 mcg/0.5 mL dosage
or	procedure	UMLS:CPT:0094A	Immunization administration by intramuscular injection of severe acute respiratory syndrome coronavirus 2 (SARS-CoV-2) (coronavirus disease [COVID-19]) vaccine, mRNA-LNP, spike protein, preservative free, 50 mcg/0.5 mL dosage; booster dose, when administered to individuals 18 years and over
or	procedure	UMLS:CPT:0034A	Immunization administration by intramuscular injection of severe acute respiratory syndrome coronavirus 2 (SARS-CoV-2) (coronavirus disease [COVID-19]) vaccine, DNA, spike

			protein, adenovirus type 26 (Ad26) vector, preservative free, 5x1010 viral particles/0.5 mL dosage; booster dose
or	procedure	UMLS:CPT:0144A	Immunization administration by intramuscular injection of severe acute respiratory syndrome coronavirus 2 (SARS-CoV-2) (coronavirus disease [COVID-19]) vaccine, mRNA-LNP, spike protein, bivalent, preservative free, 25 mcg/0.25 mL dosage, booster dose
or	procedure	UMLS:CPT:0091A	Immunization administration by intramuscular injection of severe acute respiratory syndrome coronavirus 2 (SARS-CoV-2) (coronavirus disease [COVID-19]) vaccine, mRNA-LNP, spike protein, preservative free, 50 mcg/0.5 mL dosage; first dose, when administered to individuals 6 through 11 years
or	procedure	UMLS:CPT:0174A	Immunization administration by intramuscular injection of severe acute respiratory syndrome coronavirus 2 (SARS-CoV-2) (coronavirus disease [COVID-19]) vaccine, mRNA-LNP, bivalent spike protein, preservative free, 3 mcg/0.2 mL dosage, diluent reconstituted, tris-sucrose formulation, booster
or	procedure	UMLS:CPT:0092A	Immunization administration by intramuscular injection of severe acute respiratory syndrome coronavirus 2 (SARS-

			CoV-2) (coronavirus disease [COVID-19]) vaccine, mRNA-LNP, spike protein, preservative free, 50 mcg/0.5 mL dosage; second dose, when administered to individuals 6 through 11 years
or	procedure	UMLS:CPT:1036682	Immunization administration by intramuscular injection of severe acute respiratory syndrome coronavirus 2 (SARS-CoV-2) (coronavirus disease [COVID-19]) vaccine, recombinant spike protein nanoparticle, saponin-based adjuvant, preservative free, 5 mcg/0.5 mL dosage
or	procedure	UMLS:CPT:0041A	Immunization administration by intramuscular injection of severe acute respiratory syndrome coronavirus 2 (SARS-CoV-2) (coronavirus disease [COVID-19]) vaccine, recombinant spike protein nanoparticle, saponin-based adjuvant, preservative free, 5 mcg/0.5 mL dosage; first dose
or	procedure	UMLS:CPT:0113A	Immunization administration by intramuscular injection of severe acute respiratory syndrome coronavirus 2 (SARS-CoV-2) (coronavirus disease [COVID-19]) vaccine, mRNA-LNP, spike protein, preservative free, 25 mcg/0.25 mL dosage; third dose
or	procedure	UMLS:CPT:0042A	Immunization administration by intramuscular injection of

			severe acute respiratory syndrome coronavirus 2 (SARS-CoV-2) (coronavirus disease [COVID-19]) vaccine, recombinant spike protein nanoparticle, saponin-based adjuvant, preservative free, 5 mcg/0.5 mL dosage; second dose
or	procedure	UMLS:CPT:0093A	Immunization administration by intramuscular injection of severe acute respiratory syndrome coronavirus 2 (SARS-CoV-2) (coronavirus disease [COVID-19]) vaccine, mRNA-LNP, spike protein, preservative free, 50 mcg/0.5 mL dosage; third dose, when administered to individuals 6 through 11 years
or	procedure	UMLS:CPT:1036666	Immunization administration by intramuscular injection of severe acute respiratory syndrome coronavirus 2 (SARS-CoV-2) (coronavirus disease [COVID-19]) vaccine, DNA, spike protein, chimpanzee adenovirus Oxford 1 (ChAdOx1) vector, preservative free, 5x1010 viral particles/0.5 mL dosage
or	procedure	UMLS:CPT:0044A	Immunization administration by intramuscular injection of severe acute respiratory syndrome coronavirus 2 (SARS-CoV-2) (coronavirus disease [COVID-19]) vaccine, recombinant spike protein nanoparticle, saponin-based

				adjuvant, preservative free, 5 mcg/0.5mL dosage; booster
date constraint		The terms in this group occurred between Dec 1, 2019 and Dec 31, 2023		
**Group 2**				
**Group 2A COVID-19 negative**				
must have		visit	TNX:Visit	Visit (Data Source: TriNetX)
cannot have		diagnosis	UMLS:ICD10CM:U07.1	COVID-19
	or	laboratory	UMLS:LNC:95406-5	SARS-CoV-2 (COVID-19) RNA [Presence] in Nose by NAA with probe detection (labResult: Positive)
	or	laboratory	UMLS:LNC:94845-5	SARS-CoV-2 (COVID-19) RNA [Presence] in Saliva (oral fluid) by NAA with probe detection (labResult: Positive)
	or	laboratory	UMLS:LNC:95608-6	SARS-CoV-2 (COVID-19) RNA [Presence] in Respiratory specimen by NAA with non-probe detection (labResult: Positive)
	or	laboratory	UMLS:LNC:94759-8	SARS-CoV-2 (COVID-19) RNA [Presence] in Nasopharynx by NAA with probe detection (labResult: Positive)
	or	laboratory	UMLS:LNC:94565-9	SARS-CoV-2 (COVID-19) RNA [Presence] in Nasopharynx by NAA with non-probe detection (labResult: Positive)
	or	laboratory	UMLS:LNC:94309-2	SARS-CoV-2 (COVID-19) RNA [Presence] in Specimen by NAA with probe detection (labResult: Positive)
	or	laboratory	UMLS:LNC:94500-6	SARS-CoV-2 (COVID-19) RNA [Presence] in Respiratory specimen by NAA with probe detection (labResult: Positive)

	or	diagnosis	UMLS:ICD10CM:U07.2	COVID-19, virus not identified (WHO)
	or	diagnosis	UMLS:ICD10CM:J12.82	Pneumonia due to coronavirus disease 2019
date constraint		The terms in this group occurred at any time		
event relationship		Any instance of thyroid cancer history occurred at least 1 day before the first instance of COVID-19 negative		
**Group 2B thyroid cancer history**				
cannot have		diagnosis	UMLS:ICD10CM:C73	Malignant neoplasm of thyroid gland
	or	diagnosis	UMLS:ICD10CM:Z85.850	Personal history of malignant neoplasm of thyroid

Query Criteria for Cohort post-COVID with hyperthyroidism

This query was run on the network Global Collaborative Network with 147 HCO(s) queried and 147 HCO(s) responded. A total of 98 provider(s) responded with patients. The final cohort included 10,921 patients who matched the query criteria listed in the table below.

**Ungrouped terms**				
must have		demographics	Age	Age (at least 18 years (most recent occurrence))
	and any of	demographics	UMLS:HL7V3.0:Gender:M	Male
		demographics	UMLS:HL7V3.0:Gender:F	Female
**Group 1**				
**Group 1A COVID-19, no vaccination**				
must have	any of	diagnosis	UMLS:ICD10CM:U07.1	COVID-19
		diagnosis	UMLS:ICD10CM:U07.2	COVID-19, virus not identified (WHO)
		diagnosis	UMLS:ICD10CM:J12.82	Pneumonia due to coronavirus disease 2019
		laboratory	UMLS:LNC:94500-6	SARS-CoV-2 (COVID-19) RNA [Presence] in Respiratory specimen by NAA with probe detection (labResult: Positive)

		laboratory	UMLS:LNC:94309-2	SARS-CoV-2 (COVID-19) RNA [Presence] in Specimen by NAA with probe detection (labResult: Positive)
		laboratory	UMLS:LNC:94565-9	SARS-CoV-2 (COVID-19) RNA [Presence] in Nasopharynx by NAA with non-probe detection (labResult: Positive)
		laboratory	UMLS:LNC:94759-8	SARS-CoV-2 (COVID-19) RNA [Presence] in Nasopharynx by NAA with probe detection (labResult: Positive)
		laboratory	UMLS:LNC:95608-6	SARS-CoV-2 (COVID-19) RNA [Presence] in Respiratory specimen by NAA with non-probe detection (labResult: Positive)
		laboratory	UMLS:LNC:94845-5	SARS-CoV-2 (COVID-19) RNA [Presence] in Saliva (oral fluid) by NAA with probe detection (labResult: Positive)
		laboratory	UMLS:LNC:95406-5	SARS-CoV-2 (COVID-19) RNA [Presence] in Nose by NAA with probe detection (labResult: Positive)
cannot have		medication	NLM:CVX:208	COVID-19, mRNA, LNP-S, PF, 30 mcg/0.3 mL dose
	or	medication	NLM:CVX:207	COVID-19, mRNA, LNP-S, PF, 100 mcg/0.5mL dose or 50 mcg/0.25mL dose
	or	medication	NLM:CVX:212	COVID-19 vaccine, vector-nr, rS-Ad26, PF, 0.5 mL
	or	medication	NLM:RXNORM:OMOP 5042939	COVID-19 vaccine
	or	medication	NLM:CVX:300	COVID-19, mRNA, LNP-S, bivalent, PF, 30 mcg/0.3 mL dose

or	medication	NLM:CVX:217	COVID-19, mRNA, LNP-S, PF, 30 mcg/0.3 mL dose, tris-sucrose
or	medication	NLM:CVX:229	COVID-19, mRNA, LNP-S, bivalent, PF, 50 mcg/0.5 mL or 25mcg/0.25 mL dose
or	medication	NLM:CVX:218	COVID-19, mRNA, LNP-S, PF, 10 mcg/0.2 mL dose, tris-sucrose
or	medication	NLM:CVX:520	COVID-19 mRNA, bivalent, original/Omicron BA.1, Non-US Vaccine Product, Pfizer-BioNTech
or	medication	NLM:CVX:519	COVID-19 mRNA, bivalent, original/Omicron BA.1, Non-US Vaccine (Spikevax Bivalent), Moderna
or	medication	NLM:CVX:301	COVID-19, mRNA, LNP-S, bivalent, PF, 10 mcg/0.2 mL dose
or	medication	NLM:CVX:219	COVID-19, mRNA, LNP-S, PF, 3 mcg/0.2 mL dose, tris-sucrose
or	medication	NLM:CVX:228	COVID-19, mRNA, LNP-S, PF, pediatric 25 mcg/0.25 mL dose
or	medication	NLM:CVX:230	COVID-19, mRNA, LNP-S, bivalent booster, PF, 10 mcg/0.2 mL
or	medication	NLM:CVX:221	COVID-19, mRNA, LNP-S, PF, 50 mcg/0.5 mL dose
or	medication	NLM:CVX:210	COVID-19 vaccine, vector-nr, rS-ChAdOx1, PF, 0.5 mL
or	medication	NLM:CVX:302	COVID-19, mRNA, LNP-S, bivalent, PF, 3 mcg/0.2 mL dose
or	medication	NLM:CVX:511	COVID-19 IV Non-US Vaccine (CoronaVac, Sinovac)
or	medication	NLM:RXNORM:2468231	SARS-CoV-2 (COVID-19) vaccine, mRNA spike protein
or	procedure	UMLS:CPT:91300	Severe acute respiratory syndrome coronavirus 2 (SARS-CoV-2) (coronavirus disease

			[COVID-19]) vaccine, mRNA-LNP, spike protein, preservative free, 30 mcg/0.3 mL dosage, diluent reconstituted, for intramuscular use
or	procedure	UMLS:CPT:0001A	Immunization administration by intramuscular injection of severe acute respiratory syndrome coronavirus 2 (SARS-CoV-2) (coronavirus disease [COVID-19]) vaccine, mRNA-LNP, spike protein, preservative free, 30 mcg/0.3 mL dosage, diluent reconstituted; first dose
or	procedure	UMLS:CPT:0002A	Immunization administration by intramuscular injection of severe acute respiratory syndrome coronavirus 2 (SARS-CoV-2) (coronavirus disease [COVID-19]) vaccine, mRNA-LNP, spike protein, preservative free, 30 mcg/0.3 mL dosage, diluent reconstituted; second dose
or	procedure	UMLS:CPT:91301	Severe acute respiratory syndrome coronavirus 2 (SARS-CoV-2) (coronavirus disease [COVID-19]) vaccine, mRNA-LNP, spike protein, preservative free, 100 mcg/0.5 mL dosage, for intramuscular use
or	procedure	UMLS:CPT:0011A	Immunization administration by intramuscular injection of severe acute respiratory syndrome coronavirus 2 (SARS-CoV-2) (coronavirus disease [COVID-19]) vaccine, mRNA-LNP, spike protein, preservative

			free, 100 mcg/0.5 mL dosage; first dose
or	procedure	UMLS:CPT:0012A	Immunization administration by intramuscular injection of severe acute respiratory syndrome coronavirus 2 (SARS-CoV-2) (coronavirus disease [COVID-19]) vaccine, mRNA-LNP, spike protein, preservative free, 100 mcg/0.5 mL dosage; second dose
or	procedure	UMLS:SNOMED:840534001	Administration of SARS-CoV-2 antigen vaccine
or	medication	NLM:CVX:213	SARS-CoV-2 (COVID-19) Vaccine
or	procedure	UMLS:CPT:1036660	Immunization administration by intramuscular injection of severe acute respiratory syndrome coronavirus 2 (SARS-CoV-2) (coronavirus disease [COVID-19]) vaccine, mRNA-LNP, spike protein, preservative free, 30 mcg/0.3 mL dosage, diluent reconstituted
or	procedure	UMLS:CPT:1036663	Immunization administration by intramuscular injection of severe acute respiratory syndrome coronavirus 2 (SARS-CoV-2) (coronavirus disease [COVID-19]) vaccine, mRNA-LNP, spike protein, preservative free, 100 mcg/0.5 mL dosage
or	procedure	UMLS:CPT:0124A	Immunization administration by intramuscular injection of severe acute respiratory syndrome coronavirus 2 (SARS-CoV-2) (coronavirus disease [COVID-19]) vaccine, mRNA-

			LNP, bivalent spike protein, preservative free, 30 mcg/0.3 mL dosage, tris-sucrose formulation, booster dose
or	procedure	UMLS:CPT:0004A	Immunization administration by intramuscular injection of severe acute respiratory syndrome coronavirus 2 (SARS-CoV-2) (coronavirus disease [COVID-19]) vaccine, mRNA-LNP, spike protein, preservative free, 30 mcg/0.3 mL dosage, diluent reconstituted; booster dose
or	procedure	UMLS:CPT:0003A	Immunization administration by intramuscular injection of severe acute respiratory syndrome coronavirus 2 (SARS-CoV-2) (coronavirus disease [COVID-19]) vaccine, mRNA-LNP, spike protein, preservative free, 30 mcg/0.3 mL dosage, diluent reconstituted; third dose
or	procedure	UMLS:CPT:1037166	Immunization administration by intramuscular injection of severe acute respiratory syndrome coronavirus 2 (SARS-CoV-2) (coronavirus disease [COVID-19]) vaccine, mRNA-LNP, spike protein, preservative free, 30 mcg/0.3 mL dosage, tris-sucrose formulation
or	procedure	UMLS:CPT:0054A	Immunization administration by intramuscular injection of severe acute respiratory syndrome coronavirus 2 (SARS-CoV-2) (coronavirus disease [COVID-19]) vaccine, mRNA-

			LNP, spike protein, preservative free, 30 mcg/0.3 mL dosage, tris-sucrose formulation; booster dose
or	procedure	UMLS:CPT:0064A	Immunization administration by intramuscular injection of severe acute respiratory syndrome coronavirus 2 (SARS-CoV-2) (coronavirus disease [COVID-19]) vaccine, mRNA-LNP, spike protein, preservative free, 50 mcg/0.25 mL dosage, booster dose
or	procedure	UMLS:CPT:90480	Immunization administration by intramuscular injection of severe acute respiratory syndrome coronavirus 2 (SARS-CoV-2) (coronavirus disease [COVID-19]) vaccine, single dose
or	procedure	UMLS:CPT:1037171	Immunization administration by intramuscular injection of severe acute respiratory syndrome coronavirus 2 (SARS-CoV-2) (coronavirus disease [COVID-19]) vaccine, mRNA-LNP, spike protein, preservative free, 10 mcg/0.2 mL dosage, diluent reconstituted, tris-sucrose formulation
or	procedure	UMLS:CPT:0071A	Immunization administration by intramuscular injection of severe acute respiratory syndrome coronavirus 2 (SARS-CoV-2) (coronavirus disease [COVID-19]) vaccine, mRNA-LNP, spike protein, preservative free, 10 mcg/0.2 mL dosage,

			diluent reconstituted, tris-sucrose formulation; first dose
or	procedure	UMLS:CPT:0072A	Immunization administration by intramuscular injection of severe acute respiratory syndrome coronavirus 2 (SARS-CoV-2) (coronavirus disease [COVID-19]) vaccine, mRNA-LNP, spike protein, preservative free, 10 mcg/0.2 mL dosage, diluent reconstituted, tris-sucrose formulation; second dose
or	medication	NLM:RXNORM:2610319	SARS-CoV-2 (COVID-19) vaccine, mRNA-BNT162b2 0.05 MG/ML / SARS-CoV-2 (COVID-19) vaccine, mRNA-BNT162b2 OMICRON (BA.4/BA.5) 0.05 MG/ML Injectable Suspension
or	procedure	UMLS:CPT:91313	Severe acute respiratory syndrome coronavirus 2 (SARS-CoV-2) (coronavirus disease [COVID-19]) vaccine, mRNA-LNP, spike protein, bivalent, preservative free, 50 mcg/0.5 mL dosage, for intramuscular use
or	procedure	UMLS:CPT:0134A	Immunization administration by intramuscular injection of severe acute respiratory syndrome coronavirus 2 (SARS-CoV-2) (coronavirus disease [COVID-19]) vaccine, mRNA-LNP, spike protein, bivalent, preservative free, 50 mcg/0.5 mL dosage, booster dose
or	procedure	UMLS:CPT:1037175	Immunization administration by intramuscular injection of severe acute respiratory

			syndrome coronavirus 2 (SARS-CoV-2) (coronavirus disease [COVID-19]) vaccine, DNA, spike protein, adenovirus type 26 (Ad26) vector, preservative free, 5x1010 viral particles/0.5 mL dosage
or	medication	NLM:RXNORM:2610347	0.3 ML SARS-CoV-2 (COVID-19) vaccine, mRNA-BNT162b2 0.05 MG/ML / SARS-CoV-2 (COVID-19) vaccine, mRNA-BNT162b2 OMICRON (BA.4/BA.5) -1 MG/ML Injection
or	procedure	UMLS:CPT:1037228	Immunization administration by intramuscular injection of severe acute respiratory syndrome coronavirus 2 (SARS-CoV-2) (coronavirus disease [COVID-19]) vaccine, mRNA-LNP, spike protein, preservative free, 3 mcg/0.2 mL dosage, diluent reconstituted, tris-sucrose formulation
or	procedure	UMLS:CPT:0013A	Immunization administration by intramuscular injection of severe acute respiratory syndrome coronavirus 2 (SARS-CoV-2) (coronavirus disease [COVID-19]) vaccine, mRNA-LNP, spike protein, preservative free, 100 mcg/0.5 mL dosage; third dose
or	procedure	UMLS:CPT:0081A	Immunization administration by intramuscular injection of severe acute respiratory syndrome coronavirus 2 (SARS-CoV-2) (coronavirus disease [COVID-19]) vaccine, mRNA-

			LNP, spike protein, preservative free, 3 mcg/0.2 mL dosage, diluent reconstituted, tris-sucrose formulation; first dose
or	procedure	UMLS:CPT:0082A	Immunization administration by intramuscular injection of severe acute respiratory syndrome coronavirus 2 (SARS-CoV-2) (coronavirus disease [COVID-19]) vaccine, mRNA-LNP, spike protein, preservative free, 3 mcg/0.2 mL dosage, diluent reconstituted, tris-sucrose formulation; second dose
or	medication	NLM:RXNORM:2610328	SARS-CoV-2 (COVID-19) vaccine, mRNA-1273 0.05 MG/ML / SARS-CoV-2 (COVID-19) vaccine, mRNA-1273 OMICRON (BA.4/BA.5) 0.05 MG/ML Injectable Suspension
or	procedure	UMLS:CPT:0154A	Immunization administration by intramuscular injection of severe acute respiratory syndrome coronavirus 2 (SARS-CoV-2) (coronavirus disease [COVID-19]) vaccine, mRNA-LNP, bivalent spike protein, preservative free, 10 mcg/0.2 mL dosage, diluent reconstituted, tris-sucrose formulation, booster dose
or	procedure	UMLS:CPT:0053A	Immunization administration by intramuscular injection of severe acute respiratory syndrome coronavirus 2 (SARS-CoV-2) (coronavirus disease [COVID-19]) vaccine, mRNA-

			LNP, spike protein, preservative free, 30 mcg/0.3 mL dosage, tris-sucrose formulation; third dose
or	procedure	UMLS:CPT:1037332	Immunization administration by intramuscular injection of severe acute respiratory syndrome coronavirus 2 (SARS-CoV-2) (coronavirus disease [COVID-19]) vaccine, mRNA-LNP, spike protein, preservative free, 25 mcg/0.25 mL dosage
or	procedure	UMLS:CPT:0052A	Immunization administration by intramuscular injection of severe acute respiratory syndrome coronavirus 2 (SARS-CoV-2) (coronavirus disease [COVID-19]) vaccine, mRNA-LNP, spike protein, preservative free, 30 mcg/0.3 mL dosage, tris-sucrose formulation; second dose
or	procedure	UMLS:CPT:0111A	Immunization administration by intramuscular injection of severe acute respiratory syndrome coronavirus 2 (SARS-CoV-2) (coronavirus disease [COVID-19]) vaccine, mRNA-LNP, spike protein, preservative free, 25 mcg/0.25 mL dosage; first dose
or	procedure	UMLS:CPT:0051A	Immunization administration by intramuscular injection of severe acute respiratory syndrome coronavirus 2 (SARS-CoV-2) (coronavirus disease [COVID-19]) vaccine, mRNA-LNP, spike protein, preservative

			free, 30 mcg/0.3 mL dosage, tris-sucrose formulation; first dose
or	procedure	UMLS:CPT:91311	Severe acute respiratory syndrome coronavirus 2 (SARS-CoV-2) (coronavirus disease [COVID-19]) vaccine, mRNA-LNP, spike protein, preservative free, 25 mcg/0.25 mL dosage, for intramuscular use
or	procedure	UMLS:CPT:0074A	Immunization administration by intramuscular injection of severe acute respiratory syndrome coronavirus 2 (SARS-CoV-2) (coronavirus disease [COVID-19]) vaccine, mRNA-LNP, spike protein, preservative free, 10 mcg/0.2 mL dosage, diluent reconstituted, tris-sucrose formulation; booster dose
or	procedure	UMLS:CPT:0112A	Immunization administration by intramuscular injection of severe acute respiratory syndrome coronavirus 2 (SARS-CoV-2) (coronavirus disease [COVID-19]) vaccine, mRNA-LNP, spike protein, preservative free, 25 mcg/0.25 mL dosage; second dose
or	procedure	UMLS:CPT:0083A	Immunization administration by intramuscular injection of severe acute respiratory syndrome coronavirus 2 (SARS-CoV-2) (coronavirus disease [COVID-19]) vaccine, mRNA-LNP, spike protein, preservative free, 3 mcg/0.2 mL dosage,

			diluent reconstituted, tris-sucrose formulation; third dose
or	procedure	UMLS:CPT:0073A	Immunization administration by intramuscular injection of severe acute respiratory syndrome coronavirus 2 (SARS-CoV-2) (coronavirus disease [COVID-19]) vaccine, mRNA-LNP, spike protein, preservative free, 10 mcg/0.2 mL dosage, diluent reconstituted, tris-sucrose formulation; third dose
or	procedure	UMLS:CPT:0173A	Immunization administration by intramuscular injection of severe acute respiratory syndrome coronavirus 2 (SARS-CoV-2) (coronavirus disease [COVID-19]) vaccine, mRNA-LNP, bivalent spike protein, preservative free, 3 mcg/0.2 mL dosage, diluent reconstituted, tris-sucrose formulation, third dose
or	procedure	UMLS:CPT:0164A	Immunization administration by intramuscular injection of severe acute respiratory syndrome coronavirus 2 (SARS-CoV-2) (coronavirus disease [COVID-19]) vaccine, mRNA-LNP, spike protein, bivalent, preservative free, 10 mcg/0.2 mL dosage, booster dose
or	procedure	UMLS:CPT:1037838	Immunization administration by intramuscular injection of severe acute respiratory syndrome coronavirus 2 (SARS-CoV-2) (coronavirus disease [COVID-19]) vaccine, mRNA-

			LNP, spike protein, preservative free, 50 mcg/0.5 mL dosage
or	procedure	UMLS:CPT:0094A	Immunization administration by intramuscular injection of severe acute respiratory syndrome coronavirus 2 (SARS-CoV-2) (coronavirus disease [COVID-19]) vaccine, mRNA-LNP, spike protein, preservative free, 50 mcg/0.5 mL dosage; booster dose, when administered to individuals 18 years and over
or	procedure	UMLS:CPT:0034A	Immunization administration by intramuscular injection of severe acute respiratory syndrome coronavirus 2 (SARS-CoV-2) (coronavirus disease [COVID-19]) vaccine, DNA, spike protein, adenovirus type 26 (Ad26) vector, preservative free, 5x1010 viral particles/0.5 mL dosage; booster dose
or	procedure	UMLS:CPT:0144A	Immunization administration by intramuscular injection of severe acute respiratory syndrome coronavirus 2 (SARS-CoV-2) (coronavirus disease [COVID-19]) vaccine, mRNA-LNP, spike protein, bivalent, preservative free, 25 mcg/0.25 mL dosage, booster dose
or	procedure	UMLS:CPT:0091A	Immunization administration by intramuscular injection of severe acute respiratory syndrome coronavirus 2 (SARS-CoV-2) (coronavirus disease [COVID-19]) vaccine, mRNA-

			LNP, spike protein, preservative free, 50 mcg/0.5 mL dosage; first dose, when administered to individuals 6 through 11 years
or	procedure	UMLS:CPT:0174A	Immunization administration by intramuscular injection of severe acute respiratory syndrome coronavirus 2 (SARS-CoV-2) (coronavirus disease [COVID-19]) vaccine, mRNA-LNP, bivalent spike protein, preservative free, 3 mcg/0.2 mL dosage, diluent reconstituted, tris-sucrose formulation, booster
or	procedure	UMLS:CPT:0092A	Immunization administration by intramuscular injection of severe acute respiratory syndrome coronavirus 2 (SARS-CoV-2) (coronavirus disease [COVID-19]) vaccine, mRNA-LNP, spike protein, preservative free, 50 mcg/0.5 mL dosage; second dose, when administered to individuals 6 through 11 years
or	procedure	UMLS:CPT:1036682	Immunization administration by intramuscular injection of severe acute respiratory syndrome coronavirus 2 (SARS-CoV-2) (coronavirus disease [COVID-19]) vaccine, recombinant spike protein nanoparticle, saponin-based adjuvant, preservative free, 5 mcg/0.5 mL dosage
or	procedure	UMLS:CPT:0041A	Immunization administration by intramuscular injection of

			severe acute respiratory syndrome coronavirus 2 (SARS-CoV-2) (coronavirus disease [COVID-19]) vaccine, recombinant spike protein nanoparticle, saponin-based adjuvant, preservative free, 5 mcg/0.5 mL dosage; first dose
or	procedure	UMLS:CPT:0113A	Immunization administration by intramuscular injection of severe acute respiratory syndrome coronavirus 2 (SARS-CoV-2) (coronavirus disease [COVID-19]) vaccine, mRNA-LNP, spike protein, preservative free, 25 mcg/0.25 mL dosage; third dose
or	procedure	UMLS:CPT:0042A	Immunization administration by intramuscular injection of severe acute respiratory syndrome coronavirus 2 (SARS-CoV-2) (coronavirus disease [COVID-19]) vaccine, recombinant spike protein nanoparticle, saponin-based adjuvant, preservative free, 5 mcg/0.5 mL dosage; second dose
or	procedure	UMLS:CPT:0093A	Immunization administration by intramuscular injection of severe acute respiratory syndrome coronavirus 2 (SARS-CoV-2) (coronavirus disease [COVID-19]) vaccine, mRNA-LNP, spike protein, preservative free, 50 mcg/0.5 mL dosage; third dose, when administered to individuals 6 through 11 years

		or	procedure	UMLS:CPT:1036666	Immunization administration by intramuscular injection of severe acute respiratory syndrome coronavirus 2 (SARS-CoV-2) (coronavirus disease [COVID-19]) vaccine, DNA, spike protein, chimpanzee adenovirus Oxford 1 (ChAdOx1) vector, preservative free, 5x1010 viral particles/0.5 mL dosage
		or	procedure	UMLS:CPT:0044A	Immunization administration by intramuscular injection of severe acute respiratory syndrome coronavirus 2 (SARS-CoV-2) (coronavirus disease [COVID-19]) vaccine, recombinant spike protein nanoparticle, saponin-based adjuvant, preservative free, 5 mcg/0.5mL dosage; booster
	date constraint		The terms in this group occurred between Dec 1, 2019 and Dec 31, 2023		
	event relationship		Any instance of hyperthyroidism occurred at least 1 day after any instance of COVID-19, no vaccination		
	**Group 1B hyperthyroidism**				
	must have	any of	diagnosis	UMLS:ICD10CM:E05	Thyrotoxicosis [hyperthyroidism]
			diagnosis	UMLS:ICD10CM:E06	Thyroiditis
**Group 2**					
	**Group 2A COVID-19**				
	must have	any of	diagnosis	UMLS:ICD10CM:U07.1	COVID-19
			diagnosis	UMLS:ICD10CM:U07.2	COVID-19, virus not identified (WHO)
			diagnosis	UMLS:ICD10CM:J12.82	Pneumonia due to coronavirus disease 2019
			laboratory	UMLS:LNC:94500-6	SARS-CoV-2 (COVID-19) RNA [Presence] in Respiratory specimen by NAA with probe detection (labResult: Positive)

		laboratory	UMLS:LNC:94309-2	SARS-CoV-2 (COVID-19) RNA [Presence] in Specimen by NAA with probe detection (labResult: Positive)
		laboratory	UMLS:LNC:94565-9	SARS-CoV-2 (COVID-19) RNA [Presence] in Nasopharynx by NAA with non-probe detection (labResult: Positive)
		laboratory	UMLS:LNC:94759-8	SARS-CoV-2 (COVID-19) RNA [Presence] in Nasopharynx by NAA with probe detection (labResult: Positive)
		laboratory	UMLS:LNC:95608-6	SARS-CoV-2 (COVID-19) RNA [Presence] in Respiratory specimen by NAA with non-probe detection (labResult: Positive)
		laboratory	UMLS:LNC:94845-5	SARS-CoV-2 (COVID-19) RNA [Presence] in Saliva (oral fluid) by NAA with probe detection (labResult: Positive)
		laboratory	UMLS:LNC:95406-5	SARS-CoV-2 (COVID-19) RNA [Presence] in Nose by NAA with probe detection (labResult: Positive)
	and	visit	TNX:Visit	Visit (Data Source: TriNetX)
date constraint		The terms in this group occurred at any time		
event relationship		Any instance of Hx of thyroid abnormality and thyroid ca occurred on or before any instance of COVID-19		
**Group 2B Hx of thyroid abnormality and thyroid ca**				
cannot have		diagnosis	UMLS:ICD10CM:E03	Other hypothyroidism
	or	diagnosis	UMLS:ICD10CM:E03.9	Hypothyroidism, unspecified
	or	diagnosis	UMLS:ICD10CM:E03.8	Other specified hypothyroidism
	or	diagnosis	UMLS:ICD10CM:E03.3	Postinfectious hypothyroidism

or	diagnosis	UMLS:ICD10CM:E06	Thyroiditis
or	diagnosis	UMLS:ICD10CM:C73	Malignant neoplasm of thyroid gland
or	diagnosis	UMLS:ICD10CM:Z85.850	Personal history of malignant neoplasm of thyroid
or	diagnosis	UMLS:ICD10CM:E05	Thyrotoxicosis [hyperthyroidism]
or	diagnosis	UMLS:ICD10CM:E05.80	Other thyrotoxicosis without thyrotoxic crisis or storm
or	diagnosis	UMLS:ICD10CM:E05.30	Thyrotoxicosis from ectopic thyroid tissue without thyrotoxic crisis or storm
or	diagnosis	UMLS:ICD10CM:E05.31	Thyrotoxicosis from ectopic thyroid tissue with thyrotoxic crisis or storm

Query Criteria for Cohort post-COVID with hypothyroidism

This query was run on the network Global Collaborative Network with 147 HCO(s) queried and 147 HCO(s) responded. A total of 103 provider(s) responded with patients. The final cohort included 41,883 patients who matched the query criteria listed in the table below.

**Ungrouped terms**				
must have		demographics	Age	Age (at least 18 years (most recent occurrence))
	and any of	demographics	UMLS:HL7V3.0:Gender:M	Male
		demographics	UMLS:HL7V3.0:Gender:F	Female
**Group 1**				
**Group 1A COVID-19, no vaccination**				
must have	any of	diagnosis	UMLS:ICD10CM:U07.1	COVID-19
		diagnosis	UMLS:ICD10CM:U07.2	COVID-19, virus not identified (WHO)
		diagnosis	UMLS:ICD10CM:J12.82	Pneumonia due to coronavirus disease 2019
		laboratory	UMLS:LNC:94500-6	SARS-CoV-2 (COVID-19) RNA [Presence] in Respiratory

				specimen by NAA with probe detection (labResult: Positive)
		laboratory	UMLS:LNC:94309-2	SARS-CoV-2 (COVID-19) RNA [Presence] in Specimen by NAA with probe detection (labResult: Positive)
		laboratory	UMLS:LNC:94565-9	SARS-CoV-2 (COVID-19) RNA [Presence] in Nasopharynx by NAA with non-probe detection (labResult: Positive)
		laboratory	UMLS:LNC:94759-8	SARS-CoV-2 (COVID-19) RNA [Presence] in Nasopharynx by NAA with probe detection (labResult: Positive)
		laboratory	UMLS:LNC:95608-6	SARS-CoV-2 (COVID-19) RNA [Presence] in Respiratory specimen by NAA with non-probe detection (labResult: Positive)
		laboratory	UMLS:LNC:94845-5	SARS-CoV-2 (COVID-19) RNA [Presence] in Saliva (oral fluid) by NAA with probe detection (labResult: Positive)
		laboratory	UMLS:LNC:95406-5	SARS-CoV-2 (COVID-19) RNA [Presence] in Nose by NAA with probe detection (labResult: Positive)
cannot have		medication	NLM:CVX:208	COVID-19, mRNA, LNP-S, PF, 30 mcg/0.3 mL dose
	or	medication	NLM:CVX:207	COVID-19, mRNA, LNP-S, PF, 100 mcg/0.5mL dose or 50 mcg/0.25mL dose
	or	medication	NLM:CVX:212	COVID-19 vaccine, vector-nr, rS-Ad26, PF, 0.5 mL
	or	medication	NLM:RXNORM:OMOP 5042939	COVID-19 vaccine

or	medication	NLM:CVX:300	COVID-19, mRNA, LNP-S, bivalent, PF, 30 mcg/0.3 mL dose
or	medication	NLM:CVX:217	COVID-19, mRNA, LNP-S, PF, 30 mcg/0.3 mL dose, tris-sucrose
or	medication	NLM:CVX:229	COVID-19, mRNA, LNP-S, bivalent, PF, 50 mcg/0.5 mL or 25mcg/0.25 mL dose
or	medication	NLM:CVX:218	COVID-19, mRNA, LNP-S, PF, 10 mcg/0.2 mL dose, tris-sucrose
or	medication	NLM:CVX:520	COVID-19 mRNA, bivalent, original/Omicron BA.1, Non-US Vaccine Product, Pfizer-BioNTech
or	medication	NLM:CVX:519	COVID-19 mRNA, bivalent, original/Omicron BA.1, Non-US Vaccine (Spikevax Bivalent), Moderna
or	medication	NLM:CVX:301	COVID-19, mRNA, LNP-S, bivalent, PF, 10 mcg/0.2 mL dose
or	medication	NLM:CVX:219	COVID-19, mRNA, LNP-S, PF, 3 mcg/0.2 mL dose, tris-sucrose
or	medication	NLM:CVX:228	COVID-19, mRNA, LNP-S, PF, pediatric 25 mcg/0.25 mL dose
or	medication	NLM:CVX:230	COVID-19, mRNA, LNP-S, bivalent booster, PF, 10 mcg/0.2 mL
or	medication	NLM:CVX:221	COVID-19, mRNA, LNP-S, PF, 50 mcg/0.5 mL dose
or	medication	NLM:CVX:210	COVID-19 vaccine, vector-nr, rS-ChAdOx1, PF, 0.5 mL
or	medication	NLM:CVX:302	COVID-19, mRNA, LNP-S, bivalent, PF, 3 mcg/0.2 mL dose
or	medication	NLM:CVX:511	COVID-19 IV Non-US Vaccine (CoronaVac, Sinovac)
or	medication	NLM:RXNORM:2468231	SARS-CoV-2 (COVID-19) vaccine, mRNA spike protein

or	procedure	UMLS:CPT:91300	Severe acute respiratory syndrome coronavirus 2 (SARS-CoV-2) (coronavirus disease [COVID-19]) vaccine, mRNA-LNP, spike protein, preservative free, 30 mcg/0.3 mL dosage, diluent reconstituted, for intramuscular use
or	procedure	UMLS:CPT:0001A	Immunization administration by intramuscular injection of severe acute respiratory syndrome coronavirus 2 (SARS-CoV-2) (coronavirus disease [COVID-19]) vaccine, mRNA-LNP, spike protein, preservative free, 30 mcg/0.3 mL dosage, diluent reconstituted; first dose
or	procedure	UMLS:CPT:0002A	Immunization administration by intramuscular injection of severe acute respiratory syndrome coronavirus 2 (SARS-CoV-2) (coronavirus disease [COVID-19]) vaccine, mRNA-LNP, spike protein, preservative free, 30 mcg/0.3 mL dosage, diluent reconstituted; second dose
or	procedure	UMLS:CPT:91301	Severe acute respiratory syndrome coronavirus 2 (SARS-CoV-2) (coronavirus disease [COVID-19]) vaccine, mRNA-LNP, spike protein, preservative free, 100 mcg/0.5 mL dosage, for intramuscular use
or	procedure	UMLS:CPT:0011A	Immunization administration by intramuscular injection of severe acute respiratory syndrome coronavirus 2 (SARS-

			CoV-2) (coronavirus disease [COVID-19]) vaccine, mRNA-LNP, spike protein, preservative free, 100 mcg/0.5 mL dosage; first dose
or	procedure	UMLS:CPT:0012A	Immunization administration by intramuscular injection of severe acute respiratory syndrome coronavirus 2 (SARS-CoV-2) (coronavirus disease [COVID-19]) vaccine, mRNA-LNP, spike protein, preservative free, 100 mcg/0.5 mL dosage; second dose
or	procedure	UMLS:SNOMED:840534001	Administration of SARS-CoV-2 antigen vaccine
or	medication	NLM:CVX:213	SARS-CoV-2 (COVID-19) Vaccine
or	procedure	UMLS:CPT:1036660	Immunization administration by intramuscular injection of severe acute respiratory syndrome coronavirus 2 (SARS-CoV-2) (coronavirus disease [COVID-19]) vaccine, mRNA-LNP, spike protein, preservative free, 30 mcg/0.3 mL dosage, diluent reconstituted
or	procedure	UMLS:CPT:1036663	Immunization administration by intramuscular injection of severe acute respiratory syndrome coronavirus 2 (SARS-CoV-2) (coronavirus disease [COVID-19]) vaccine, mRNA-LNP, spike protein, preservative free, 100 mcg/0.5 mL dosage
or	procedure	UMLS:CPT:0124A	Immunization administration by intramuscular injection of severe acute respiratory

			syndrome coronavirus 2 (SARS-CoV-2) (coronavirus disease [COVID-19]) vaccine, mRNA-LNP, bivalent spike protein, preservative free, 30 mcg/0.3 mL dosage, tris-sucrose formulation, booster dose
or	procedure	UMLS:CPT:0004A	Immunization administration by intramuscular injection of severe acute respiratory syndrome coronavirus 2 (SARS-CoV-2) (coronavirus disease [COVID-19]) vaccine, mRNA-LNP, spike protein, preservative free, 30 mcg/0.3 mL dosage, diluent reconstituted; booster dose
or	procedure	UMLS:CPT:0003A	Immunization administration by intramuscular injection of severe acute respiratory syndrome coronavirus 2 (SARS-CoV-2) (coronavirus disease [COVID-19]) vaccine, mRNA-LNP, spike protein, preservative free, 30 mcg/0.3 mL dosage, diluent reconstituted; third dose
or	procedure	UMLS:CPT:1037166	Immunization administration by intramuscular injection of severe acute respiratory syndrome coronavirus 2 (SARS-CoV-2) (coronavirus disease [COVID-19]) vaccine, mRNA-LNP, spike protein, preservative free, 30 mcg/0.3 mL dosage, tris-sucrose formulation
or	procedure	UMLS:CPT:0054A	Immunization administration by intramuscular injection of severe acute respiratory

			syndrome coronavirus 2 (SARS-CoV-2) (coronavirus disease [COVID-19]) vaccine, mRNA-LNP, spike protein, preservative free, 30 mcg/0.3 mL dosage, tris-sucrose formulation; booster dose
or	procedure	UMLS:CPT:0064A	Immunization administration by intramuscular injection of severe acute respiratory syndrome coronavirus 2 (SARS-CoV-2) (coronavirus disease [COVID-19]) vaccine, mRNA-LNP, spike protein, preservative free, 50 mcg/0.25 mL dosage, booster dose
or	procedure	UMLS:CPT:90480	Immunization administration by intramuscular injection of severe acute respiratory syndrome coronavirus 2 (SARS-CoV-2) (coronavirus disease [COVID-19]) vaccine, single dose
or	procedure	UMLS:CPT:1037171	Immunization administration by intramuscular injection of severe acute respiratory syndrome coronavirus 2 (SARS-CoV-2) (coronavirus disease [COVID-19]) vaccine, mRNA-LNP, spike protein, preservative free, 10 mcg/0.2 mL dosage, diluent reconstituted, tris-sucrose formulation
or	procedure	UMLS:CPT:0071A	Immunization administration by intramuscular injection of severe acute respiratory syndrome coronavirus 2 (SARS-CoV-2) (coronavirus disease

			[COVID-19]) vaccine, mRNA-LNP, spike protein, preservative free, 10 mcg/0.2 mL dosage, diluent reconstituted, tris-sucrose formulation; first dose
or	procedure	UMLS:CPT:0072A	Immunization administration by intramuscular injection of severe acute respiratory syndrome coronavirus 2 (SARS-CoV-2) (coronavirus disease [COVID-19]) vaccine, mRNA-LNP, spike protein, preservative free, 10 mcg/0.2 mL dosage, diluent reconstituted, tris-sucrose formulation; second dose
or	medication	NLM:RXNORM:2610319	SARS-CoV-2 (COVID-19) vaccine, mRNA-BNT162b2 0.05 MG/ML / SARS-CoV-2 (COVID-19) vaccine, mRNA-BNT162b2 OMICRON (BA.4/BA.5) 0.05 MG/ML Injectable Suspension
or	procedure	UMLS:CPT:91313	Severe acute respiratory syndrome coronavirus 2 (SARS-CoV-2) (coronavirus disease [COVID-19]) vaccine, mRNA-LNP, spike protein, bivalent, preservative free, 50 mcg/0.5 mL dosage, for intramuscular use
or	procedure	UMLS:CPT:0134A	Immunization administration by intramuscular injection of severe acute respiratory syndrome coronavirus 2 (SARS-CoV-2) (coronavirus disease [COVID-19]) vaccine, mRNA-LNP, spike protein, bivalent, preservative free, 50 mcg/0.5 mL dosage, booster dose

or	procedure	UMLS:CPT:1037175	Immunization administration by intramuscular injection of severe acute respiratory syndrome coronavirus 2 (SARS-CoV-2) (coronavirus disease [COVID-19]) vaccine, DNA, spike protein, adenovirus type 26 (Ad26) vector, preservative free, 5x1010 viral particles/0.5 mL dosage
or	medication	NLM:RXNORM:2610347	0.3 ML SARS-CoV-2 (COVID-19) vaccine, mRNA-BNT162b2 0.05 MG/ML / SARS-CoV-2 (COVID-19) vaccine, mRNA-BNT162b2 OMICRON (BA.4/BA.5) -1 MG/ML Injection
or	procedure	UMLS:CPT:1037228	Immunization administration by intramuscular injection of severe acute respiratory syndrome coronavirus 2 (SARS-CoV-2) (coronavirus disease [COVID-19]) vaccine, mRNA-LNP, spike protein, preservative free, 3 mcg/0.2 mL dosage, diluent reconstituted, tris-sucrose formulation
or	procedure	UMLS:CPT:0013A	Immunization administration by intramuscular injection of severe acute respiratory syndrome coronavirus 2 (SARS-CoV-2) (coronavirus disease [COVID-19]) vaccine, mRNA-LNP, spike protein, preservative free, 100 mcg/0.5 mL dosage; third dose
or	procedure	UMLS:CPT:0081A	Immunization administration by intramuscular injection of severe acute respiratory

			syndrome coronavirus 2 (SARS-CoV-2) (coronavirus disease [COVID-19]) vaccine, mRNA-LNP, spike protein, preservative free, 3 mcg/0.2 mL dosage, diluent reconstituted, tris-sucrose formulation; first dose
or	procedure	UMLS:CPT:0082A	Immunization administration by intramuscular injection of severe acute respiratory syndrome coronavirus 2 (SARS-CoV-2) (coronavirus disease [COVID-19]) vaccine, mRNA-LNP, spike protein, preservative free, 3 mcg/0.2 mL dosage, diluent reconstituted, tris-sucrose formulation; second dose
or	medication	NLM:RXNORM:2610328	SARS-CoV-2 (COVID-19) vaccine, mRNA-1273 0.05 MG/ML / SARS-CoV-2 (COVID-19) vaccine, mRNA-1273 OMICRON (BA.4/BA.5) 0.05 MG/ML Injectable Suspension
or	procedure	UMLS:CPT:0154A	Immunization administration by intramuscular injection of severe acute respiratory syndrome coronavirus 2 (SARS-CoV-2) (coronavirus disease [COVID-19]) vaccine, mRNA-LNP, bivalent spike protein, preservative free, 10 mcg/0.2 mL dosage, diluent reconstituted, tris-sucrose formulation, booster dose
or	procedure	UMLS:CPT:0053A	Immunization administration by intramuscular injection of severe acute respiratory

			syndrome coronavirus 2 (SARS-CoV-2) (coronavirus disease [COVID-19]) vaccine, mRNA-LNP, spike protein, preservative free, 30 mcg/0.3 mL dosage, tris-sucrose formulation; third dose
or	procedure	UMLS:CPT:1037332	Immunization administration by intramuscular injection of severe acute respiratory syndrome coronavirus 2 (SARS-CoV-2) (coronavirus disease [COVID-19]) vaccine, mRNA-LNP, spike protein, preservative free, 25 mcg/0.25 mL dosage
or	procedure	UMLS:CPT:0052A	Immunization administration by intramuscular injection of severe acute respiratory syndrome coronavirus 2 (SARS-CoV-2) (coronavirus disease [COVID-19]) vaccine, mRNA-LNP, spike protein, preservative free, 30 mcg/0.3 mL dosage, tris-sucrose formulation; second dose
or	procedure	UMLS:CPT:0111A	Immunization administration by intramuscular injection of severe acute respiratory syndrome coronavirus 2 (SARS-CoV-2) (coronavirus disease [COVID-19]) vaccine, mRNA-LNP, spike protein, preservative free, 25 mcg/0.25 mL dosage; first dose
or	procedure	UMLS:CPT:0051A	Immunization administration by intramuscular injection of severe acute respiratory syndrome coronavirus 2 (SARS-

			CoV-2) (coronavirus disease [COVID-19]) vaccine, mRNA-LNP, spike protein, preservative free, 30 mcg/0.3 mL dosage, tris-sucrose formulation; first dose
or	procedure	UMLS:CPT:91311	Severe acute respiratory syndrome coronavirus 2 (SARS-CoV-2) (coronavirus disease [COVID-19]) vaccine, mRNA-LNP, spike protein, preservative free, 25 mcg/0.25 mL dosage, for intramuscular use
or	procedure	UMLS:CPT:0074A	Immunization administration by intramuscular injection of severe acute respiratory syndrome coronavirus 2 (SARS-CoV-2) (coronavirus disease [COVID-19]) vaccine, mRNA-LNP, spike protein, preservative free, 10 mcg/0.2 mL dosage, diluent reconstituted, tris-sucrose formulation; booster dose
or	procedure	UMLS:CPT:0112A	Immunization administration by intramuscular injection of severe acute respiratory syndrome coronavirus 2 (SARS-CoV-2) (coronavirus disease [COVID-19]) vaccine, mRNA-LNP, spike protein, preservative free, 25 mcg/0.25 mL dosage; second dose
or	procedure	UMLS:CPT:0083A	Immunization administration by intramuscular injection of severe acute respiratory syndrome coronavirus 2 (SARS-CoV-2) (coronavirus disease

			[COVID-19]) vaccine, mRNA-LNP, spike protein, preservative free, 3 mcg/0.2 mL dosage, diluent reconstituted, tris-sucrose formulation; third dose
or	procedure	UMLS:CPT:0073A	Immunization administration by intramuscular injection of severe acute respiratory syndrome coronavirus 2 (SARS-CoV-2) (coronavirus disease [COVID-19]) vaccine, mRNA-LNP, spike protein, preservative free, 10 mcg/0.2 mL dosage, diluent reconstituted, tris-sucrose formulation; third dose
or	procedure	UMLS:CPT:0173A	Immunization administration by intramuscular injection of severe acute respiratory syndrome coronavirus 2 (SARS-CoV-2) (coronavirus disease [COVID-19]) vaccine, mRNA-LNP, bivalent spike protein, preservative free, 3 mcg/0.2 mL dosage, diluent reconstituted, tris-sucrose formulation, third dose
or	procedure	UMLS:CPT:0164A	Immunization administration by intramuscular injection of severe acute respiratory syndrome coronavirus 2 (SARS-CoV-2) (coronavirus disease [COVID-19]) vaccine, mRNA-LNP, spike protein, bivalent, preservative free, 10 mcg/0.2 mL dosage, booster dose
or	procedure	UMLS:CPT:1037838	Immunization administration by intramuscular injection of severe acute respiratory

			syndrome coronavirus 2 (SARS-CoV-2) (coronavirus disease [COVID-19]) vaccine, mRNA-LNP, spike protein, preservative free, 50 mcg/0.5 mL dosage
or	procedure	UMLS:CPT:0094A	Immunization administration by intramuscular injection of severe acute respiratory syndrome coronavirus 2 (SARS-CoV-2) (coronavirus disease [COVID-19]) vaccine, mRNA-LNP, spike protein, preservative free, 50 mcg/0.5 mL dosage; booster dose, when administered to individuals 18 years and over
or	procedure	UMLS:CPT:0034A	Immunization administration by intramuscular injection of severe acute respiratory syndrome coronavirus 2 (SARS-CoV-2) (coronavirus disease [COVID-19]) vaccine, DNA, spike protein, adenovirus type 26 (Ad26) vector, preservative free, 5x1010 viral particles/0.5 mL dosage; booster dose
or	procedure	UMLS:CPT:0144A	Immunization administration by intramuscular injection of severe acute respiratory syndrome coronavirus 2 (SARS-CoV-2) (coronavirus disease [COVID-19]) vaccine, mRNA-LNP, spike protein, bivalent, preservative free, 25 mcg/0.25 mL dosage, booster dose
or	procedure	UMLS:CPT:0091A	Immunization administration by intramuscular injection of severe acute respiratory

			syndrome coronavirus 2 (SARS-CoV-2) (coronavirus disease [COVID-19]) vaccine, mRNA-LNP, spike protein, preservative free, 50 mcg/0.5 mL dosage; first dose, when administered to individuals 6 through 11 years
or	procedure	UMLS:CPT:0174A	Immunization administration by intramuscular injection of severe acute respiratory syndrome coronavirus 2 (SARS-CoV-2) (coronavirus disease [COVID-19]) vaccine, mRNA-LNP, bivalent spike protein, preservative free, 3 mcg/0.2 mL dosage, diluent reconstituted, tris-sucrose formulation, booster
or	procedure	UMLS:CPT:0092A	Immunization administration by intramuscular injection of severe acute respiratory syndrome coronavirus 2 (SARS-CoV-2) (coronavirus disease [COVID-19]) vaccine, mRNA-LNP, spike protein, preservative free, 50 mcg/0.5 mL dosage; second dose, when administered to individuals 6 through 11 years
or	procedure	UMLS:CPT:1036682	Immunization administration by intramuscular injection of severe acute respiratory syndrome coronavirus 2 (SARS-CoV-2) (coronavirus disease [COVID-19]) vaccine, recombinant spike protein nanoparticle, saponin-based

			adjuvant, preservative free, 5 mcg/0.5 mL dosage
or	procedure	UMLS:CPT:0041A	Immunization administration by intramuscular injection of severe acute respiratory syndrome coronavirus 2 (SARS-CoV-2) (coronavirus disease [COVID-19]) vaccine, recombinant spike protein nanoparticle, saponin-based adjuvant, preservative free, 5 mcg/0.5 mL dosage; first dose
or	procedure	UMLS:CPT:0113A	Immunization administration by intramuscular injection of severe acute respiratory syndrome coronavirus 2 (SARS-CoV-2) (coronavirus disease [COVID-19]) vaccine, mRNA-LNP, spike protein, preservative free, 25 mcg/0.25 mL dosage; third dose
or	procedure	UMLS:CPT:0042A	Immunization administration by intramuscular injection of severe acute respiratory syndrome coronavirus 2 (SARS-CoV-2) (coronavirus disease [COVID-19]) vaccine, recombinant spike protein nanoparticle, saponin-based adjuvant, preservative free, 5 mcg/0.5 mL dosage; second dose
or	procedure	UMLS:CPT:0093A	Immunization administration by intramuscular injection of severe acute respiratory syndrome coronavirus 2 (SARS-CoV-2) (coronavirus disease [COVID-19]) vaccine, mRNA-

				LNP, spike protein, preservative free, 50 mcg/0.5 mL dosage; third dose, when administered to individuals 6 through 11 years
	or	procedure	UMLS:CPT:1036666	Immunization administration by intramuscular injection of severe acute respiratory syndrome coronavirus 2 (SARS-CoV-2) (coronavirus disease [COVID-19]) vaccine, DNA, spike protein, chimpanzee adenovirus Oxford 1 (ChAdOx1) vector, preservative free, 5x1010 viral particles/0.5 mL dosage
	or	procedure	UMLS:CPT:0044A	Immunization administration by intramuscular injection of severe acute respiratory syndrome coronavirus 2 (SARS-CoV-2) (coronavirus disease [COVID-19]) vaccine, recombinant spike protein nanoparticle, saponin-based adjuvant, preservative free, 5 mcg/0.5mL dosage; booster
date constraint		The terms in this group occurred between Dec 1, 2019 and Dec 31, 2023		
event relationship		Any instance of hyperthyroidism occurred at least 1 day after any instance of COVID-19, no vaccination		
**Group 1B hyperthyroidism**				
must have	any of	diagnosis	UMLS:ICD10CM:E03	Other hypothyroidism
		diagnosis	UMLS:ICD10CM:E03.9	Hypothyroidism, unspecified
		diagnosis	UMLS:ICD10CM:E03.8	Other specified hypothyroidism
		diagnosis	UMLS:ICD10CM:E06	Thyroiditis
		diagnosis	UMLS:ICD10CM:E03.3	Postinfectious hypothyroidism

Group 2

Group 2A COVID-19

must have	any of	diagnosis	UMLS:ICD10CM:U07.1	COVID-19
		diagnosis	UMLS:ICD10CM:U07.2	COVID-19, virus not identified (WHO)
		diagnosis	UMLS:ICD10CM:J12.82	Pneumonia due to coronavirus disease 2019
		laboratory	UMLS:LNC:94500-6	SARS-CoV-2 (COVID-19) RNA [Presence] in Respiratory specimen by NAA with probe detection (labResult: Positive)
		laboratory	UMLS:LNC:94309-2	SARS-CoV-2 (COVID-19) RNA [Presence] in Specimen by NAA with probe detection (labResult: Positive)
		laboratory	UMLS:LNC:94565-9	SARS-CoV-2 (COVID-19) RNA [Presence] in Nasopharynx by NAA with non-probe detection (labResult: Positive)
		laboratory	UMLS:LNC:94759-8	SARS-CoV-2 (COVID-19) RNA [Presence] in Nasopharynx by NAA with probe detection (labResult: Positive)
		laboratory	UMLS:LNC:95608-6	SARS-CoV-2 (COVID-19) RNA [Presence] in Respiratory specimen by NAA with non-probe detection (labResult: Positive)
		laboratory	UMLS:LNC:94845-5	SARS-CoV-2 (COVID-19) RNA [Presence] in Saliva (oral fluid) by NAA with probe detection (labResult: Positive)
		laboratory	UMLS:LNC:95406-5	SARS-CoV-2 (COVID-19) RNA [Presence] in Nose by NAA with probe detection (labResult: Positive)
	and	visit	TNX:Visit	Visit (Data Source: TriNetX)
date constraint		The terms in this group occurred at any time		

event relationship		Any instance of Hx of thyroid abnormality and thyroid ca occurred on or before any instance of COVID-19		
**Group 2B Hx of thyroid abnormality and thyroid ca**				
cannot have		diagnosis	UMLS:ICD10CM:E03	Other hypothyroidism
	or	diagnosis	UMLS:ICD10CM:E03.9	Hypothyroidism, unspecified
	or	diagnosis	UMLS:ICD10CM:E03.8	Other specified hypothyroidism
	or	diagnosis	UMLS:ICD10CM:E03.3	Postinfectious hypothyroidism
	or	diagnosis	UMLS:ICD10CM:E06	Thyroiditis
	or	diagnosis	UMLS:ICD10CM:C73	Malignant neoplasm of thyroid gland
	or	diagnosis	UMLS:ICD10CM:Z85.850	Personal history of malignant neoplasm of thyroid
	or	diagnosis	UMLS:ICD10CM:E05	Thyrotoxicosis [hyperthyroidism]
	or	diagnosis	UMLS:ICD10CM:E05.80	Other thyrotoxicosis without thyrotoxic crisis or storm
	or	diagnosis	UMLS:ICD10CM:E05.30	Thyrotoxicosis from ectopic thyroid tissue without thyrotoxic crisis or storm
	or	diagnosis	UMLS:ICD10CM:E05.31	Thyrotoxicosis from ectopic thyroid tissue with thyrotoxic crisis or storm

Query Criteria for Cohort post-COVID s/p Nirmatrelvir/Ritonavir
This query was run on the network Global Collaborative Network with 148 HCO(s) queried and 148 HCO(s) responded. A total of 56 provider(s) responded with patients. The final cohort included 90,998 patients who matched the query criteria listed in the table below.

**Ungrouped terms**				
must have		demographics	Age	Age (at least 18 years (most recent occurrence))
	and any of	demographics	UMLS:HL7V3.0:Gender:M	Male
		demographics	UMLS:HL7V3.0:Gender:F	Female
**Group 1**				

Group 1A COVID-19, no vaccination				
must have	any of	diagnosis	UMLS:ICD10CM:U07.1	COVID-19
		diagnosis	UMLS:ICD10CM:U07.2	COVID-19, virus not identified (WHO)
		diagnosis	UMLS:ICD10CM:J12.82	Pneumonia due to coronavirus disease 2019
		laboratory	UMLS:LNC:94500-6	SARS-CoV-2 (COVID-19) RNA [Presence] in Respiratory specimen by NAA with probe detection (labResult: Positive)
		laboratory	UMLS:LNC:94309-2	SARS-CoV-2 (COVID-19) RNA [Presence] in Specimen by NAA with probe detection (labResult: Positive)
		laboratory	UMLS:LNC:94565-9	SARS-CoV-2 (COVID-19) RNA [Presence] in Nasopharynx by NAA with non-probe detection (labResult: Positive)
		laboratory	UMLS:LNC:94759-8	SARS-CoV-2 (COVID-19) RNA [Presence] in Nasopharynx by NAA with probe detection (labResult: Positive)
		laboratory	UMLS:LNC:95608-6	SARS-CoV-2 (COVID-19) RNA [Presence] in Respiratory specimen by NAA with non-probe detection (labResult: Positive)
		laboratory	UMLS:LNC:94845-5	SARS-CoV-2 (COVID-19) RNA [Presence] in Saliva (oral fluid) by NAA with probe detection (labResult: Positive)
		laboratory	UMLS:LNC:95406-5	SARS-CoV-2 (COVID-19) RNA [Presence] in Nose by NAA with probe detection (labResult: Positive)
cannot have		medication	NLM:CVX:208	COVID-19, mRNA, LNP-S, PF, 30 mcg/0.3 mL dose

or	medication	NLM:CVX:207	COVID-19, mRNA, LNP-S, PF, 100 mcg/0.5mL dose or 50 mcg/0.25mL dose
or	medication	NLM:CVX:212	COVID-19 vaccine, vector-nr, rS-Ad26, PF, 0.5 mL
or	medication	NLM:RXNORM:OMOP 5042939	COVID-19 vaccine
or	medication	NLM:CVX:300	COVID-19, mRNA, LNP-S, bivalent, PF, 30 mcg/0.3 mL dose
or	medication	NLM:CVX:217	COVID-19, mRNA, LNP-S, PF, 30 mcg/0.3 mL dose, tris-sucrose
or	medication	NLM:CVX:229	COVID-19, mRNA, LNP-S, bivalent, PF, 50 mcg/0.5 mL or 25mcg/0.25 mL dose
or	medication	NLM:CVX:218	COVID-19, mRNA, LNP-S, PF, 10 mcg/0.2 mL dose, tris-sucrose
or	medication	NLM:CVX:520	COVID-19 mRNA, bivalent, original/Omicron BA.1, Non-US Vaccine Product, Pfizer-BioNTech
or	medication	NLM:CVX:519	COVID-19 mRNA, bivalent, original/Omicron BA.1, Non-US Vaccine (Spikevax Bivalent), Moderna
or	medication	NLM:CVX:301	COVID-19, mRNA, LNP-S, bivalent, PF, 10 mcg/0.2 mL dose
or	medication	NLM:CVX:219	COVID-19, mRNA, LNP-S, PF, 3 mcg/0.2 mL dose, tris-sucrose
or	medication	NLM:CVX:228	COVID-19, mRNA, LNP-S, PF, pediatric 25 mcg/0.25 mL dose
or	medication	NLM:CVX:230	COVID-19, mRNA, LNP-S, bivalent booster, PF, 10 mcg/0.2 mL
or	medication	NLM:CVX:221	COVID-19, mRNA, LNP-S, PF, 50 mcg/0.5 mL dose

or	medication	NLM:CVX:210	COVID-19 vaccine, vector-nr, rS-ChAdOx1, PF, 0.5 mL
or	medication	NLM:CVX:302	COVID-19, mRNA, LNP-S, bivalent, PF, 3 mcg/0.2 mL dose
or	medication	NLM:CVX:511	COVID-19 IV Non-US Vaccine (CoronaVac, Sinovac)
or	medication	NLM:RXNORM:2468231	SARS-CoV-2 (COVID-19) vaccine, mRNA spike protein
or	procedure	UMLS:CPT:91300	Severe acute respiratory syndrome coronavirus 2 (SARS-CoV-2) (coronavirus disease [COVID-19]) vaccine, mRNA-LNP, spike protein, preservative free, 30 mcg/0.3 mL dosage, diluent reconstituted, for intramuscular use
or	procedure	UMLS:CPT:0001A	Immunization administration by intramuscular injection of severe acute respiratory syndrome coronavirus 2 (SARS-CoV-2) (coronavirus disease [COVID-19]) vaccine, mRNA-LNP, spike protein, preservative free, 30 mcg/0.3 mL dosage, diluent reconstituted; first dose
or	procedure	UMLS:CPT:0002A	Immunization administration by intramuscular injection of severe acute respiratory syndrome coronavirus 2 (SARS-CoV-2) (coronavirus disease [COVID-19]) vaccine, mRNA-LNP, spike protein, preservative free, 30 mcg/0.3 mL dosage, diluent reconstituted; second dose
or	procedure	UMLS:CPT:91301	Severe acute respiratory syndrome coronavirus 2 (SARS-CoV-2) (coronavirus disease

			[COVID-19]) vaccine, mRNA-LNP, spike protein, preservative free, 100 mcg/0.5 mL dosage, for intramuscular use
or	procedure	UMLS:CPT:0011A	Immunization administration by intramuscular injection of severe acute respiratory syndrome coronavirus 2 (SARS-CoV-2) (coronavirus disease [COVID-19]) vaccine, mRNA-LNP, spike protein, preservative free, 100 mcg/0.5 mL dosage; first dose
or	procedure	UMLS:CPT:0012A	Immunization administration by intramuscular injection of severe acute respiratory syndrome coronavirus 2 (SARS-CoV-2) (coronavirus disease [COVID-19]) vaccine, mRNA-LNP, spike protein, preservative free, 100 mcg/0.5 mL dosage; second dose
or	procedure	UMLS:SNOMED:840534001	Administration of SARS-CoV-2 antigen vaccine
or	medication	NLM:CVX:213	SARS-CoV-2 (COVID-19) Vaccine
or	procedure	UMLS:CPT:1036660	Immunization administration by intramuscular injection of severe acute respiratory syndrome coronavirus 2 (SARS-CoV-2) (coronavirus disease [COVID-19]) vaccine, mRNA-LNP, spike protein, preservative free, 30 mcg/0.3 mL dosage, diluent reconstituted
or	procedure	UMLS:CPT:1036663	Immunization administration by intramuscular injection of severe acute respiratory

			syndrome coronavirus 2 (SARS-CoV-2) (coronavirus disease [COVID-19]) vaccine, mRNA-LNP, spike protein, preservative free, 100 mcg/0.5 mL dosage
or	procedure	UMLS:CPT:0124A	Immunization administration by intramuscular injection of severe acute respiratory syndrome coronavirus 2 (SARS-CoV-2) (coronavirus disease [COVID-19]) vaccine, mRNA-LNP, bivalent spike protein, preservative free, 30 mcg/0.3 mL dosage, tris-sucrose formulation, booster dose
or	procedure	UMLS:CPT:0004A	Immunization administration by intramuscular injection of severe acute respiratory syndrome coronavirus 2 (SARS-CoV-2) (coronavirus disease [COVID-19]) vaccine, mRNA-LNP, spike protein, preservative free, 30 mcg/0.3 mL dosage, diluent reconstituted; booster dose
or	procedure	UMLS:CPT:0003A	Immunization administration by intramuscular injection of severe acute respiratory syndrome coronavirus 2 (SARS-CoV-2) (coronavirus disease [COVID-19]) vaccine, mRNA-LNP, spike protein, preservative free, 30 mcg/0.3 mL dosage, diluent reconstituted; third dose
or	procedure	UMLS:CPT:1037166	Immunization administration by intramuscular injection of severe acute respiratory syndrome coronavirus 2 (SARS-

			CoV-2) (coronavirus disease [COVID-19]) vaccine, mRNA-LNP, spike protein, preservative free, 30 mcg/0.3 mL dosage, tris-sucrose formulation
or	procedure	UMLS:CPT:0054A	Immunization administration by intramuscular injection of severe acute respiratory syndrome coronavirus 2 (SARS-CoV-2) (coronavirus disease [COVID-19]) vaccine, mRNA-LNP, spike protein, preservative free, 30 mcg/0.3 mL dosage, tris-sucrose formulation; booster dose
or	procedure	UMLS:CPT:0064A	Immunization administration by intramuscular injection of severe acute respiratory syndrome coronavirus 2 (SARS-CoV-2) (coronavirus disease [COVID-19]) vaccine, mRNA-LNP, spike protein, preservative free, 50 mcg/0.25 mL dosage, booster dose
or	procedure	UMLS:CPT:90480	Immunization administration by intramuscular injection of severe acute respiratory syndrome coronavirus 2 (SARS-CoV-2) (coronavirus disease [COVID-19]) vaccine, single dose
or	procedure	UMLS:CPT:1037171	Immunization administration by intramuscular injection of severe acute respiratory syndrome coronavirus 2 (SARS-CoV-2) (coronavirus disease [COVID-19]) vaccine, mRNA-LNP, spike protein, preservative

			free, 10 mcg/0.2 mL dosage, diluent reconstituted, tris-sucrose formulation
or	procedure	UMLS:CPT:0071A	Immunization administration by intramuscular injection of severe acute respiratory syndrome coronavirus 2 (SARS-CoV-2) (coronavirus disease [COVID-19]) vaccine, mRNA-LNP, spike protein, preservative free, 10 mcg/0.2 mL dosage, diluent reconstituted, tris-sucrose formulation; first dose
or	procedure	UMLS:CPT:0072A	Immunization administration by intramuscular injection of severe acute respiratory syndrome coronavirus 2 (SARS-CoV-2) (coronavirus disease [COVID-19]) vaccine, mRNA-LNP, spike protein, preservative free, 10 mcg/0.2 mL dosage, diluent reconstituted, tris-sucrose formulation; second dose
or	medication	NLM:RXNORM:2610319	SARS-CoV-2 (COVID-19) vaccine, mRNA-BNT162b2 0.05 MG/ML / SARS-CoV-2 (COVID-19) vaccine, mRNA-BNT162b2 OMICRON (BA.4/BA.5) 0.05 MG/ML Injectable Suspension
or	procedure	UMLS:CPT:91313	Severe acute respiratory syndrome coronavirus 2 (SARS-CoV-2) (coronavirus disease [COVID-19]) vaccine, mRNA-LNP, spike protein, bivalent, preservative free, 50 mcg/0.5 mL dosage, for intramuscular use

or	procedure	UMLS:CPT:0134A	Immunization administration by intramuscular injection of severe acute respiratory syndrome coronavirus 2 (SARS-CoV-2) (coronavirus disease [COVID-19]) vaccine, mRNA-LNP, spike protein, bivalent, preservative free, 50 mcg/0.5 mL dosage, booster dose
or	procedure	UMLS:CPT:1037175	Immunization administration by intramuscular injection of severe acute respiratory syndrome coronavirus 2 (SARS-CoV-2) (coronavirus disease [COVID-19]) vaccine, DNA, spike protein, adenovirus type 26 (Ad26) vector, preservative free, 5x1010 viral particles/0.5 mL dosage
or	medication	NLM:RXNORM:2610347	0.3 ML SARS-CoV-2 (COVID-19) vaccine, mRNA-BNT162b2 0.05 MG/ML / SARS-CoV-2 (COVID-19) vaccine, mRNA-BNT162b2 OMICRON (BA.4/BA.5) -1 MG/ML Injection
or	procedure	UMLS:CPT:1037228	Immunization administration by intramuscular injection of severe acute respiratory syndrome coronavirus 2 (SARS-CoV-2) (coronavirus disease [COVID-19]) vaccine, mRNA-LNP, spike protein, preservative free, 3 mcg/0.2 mL dosage, diluent reconstituted, tris-sucrose formulation
or	procedure	UMLS:CPT:0013A	Immunization administration by intramuscular injection of severe acute respiratory

			syndrome coronavirus 2 (SARS-CoV-2) (coronavirus disease [COVID-19]) vaccine, mRNA-LNP, spike protein, preservative free, 100 mcg/0.5 mL dosage; third dose
or	procedure	UMLS:CPT:0081A	Immunization administration by intramuscular injection of severe acute respiratory syndrome coronavirus 2 (SARS-CoV-2) (coronavirus disease [COVID-19]) vaccine, mRNA-LNP, spike protein, preservative free, 3 mcg/0.2 mL dosage, diluent reconstituted, tris-sucrose formulation; first dose
or	procedure	UMLS:CPT:0082A	Immunization administration by intramuscular injection of severe acute respiratory syndrome coronavirus 2 (SARS-CoV-2) (coronavirus disease [COVID-19]) vaccine, mRNA-LNP, spike protein, preservative free, 3 mcg/0.2 mL dosage, diluent reconstituted, tris-sucrose formulation; second dose
or	medication	NLM:RXNORM:2610328	SARS-CoV-2 (COVID-19) vaccine, mRNA-1273 0.05 MG/ML / SARS-CoV-2 (COVID-19) vaccine, mRNA-1273 OMICRON (BA.4/BA.5) 0.05 MG/ML Injectable Suspension
or	procedure	UMLS:CPT:0154A	Immunization administration by intramuscular injection of severe acute respiratory syndrome coronavirus 2 (SARS-CoV-2) (coronavirus disease

			[COVID-19]) vaccine, mRNA-LNP, bivalent spike protein, preservative free, 10 mcg/0.2 mL dosage, diluent reconstituted, tris-sucrose formulation, booster dose
or	procedure	UMLS:CPT:0053A	Immunization administration by intramuscular injection of severe acute respiratory syndrome coronavirus 2 (SARS-CoV-2) (coronavirus disease [COVID-19]) vaccine, mRNA-LNP, spike protein, preservative free, 30 mcg/0.3 mL dosage, tris-sucrose formulation; third dose
or	procedure	UMLS:CPT:1037332	Immunization administration by intramuscular injection of severe acute respiratory syndrome coronavirus 2 (SARS-CoV-2) (coronavirus disease [COVID-19]) vaccine, mRNA-LNP, spike protein, preservative free, 25 mcg/0.25 mL dosage
or	procedure	UMLS:CPT:0052A	Immunization administration by intramuscular injection of severe acute respiratory syndrome coronavirus 2 (SARS-CoV-2) (coronavirus disease [COVID-19]) vaccine, mRNA-LNP, spike protein, preservative free, 30 mcg/0.3 mL dosage, tris-sucrose formulation; second dose
or	procedure	UMLS:CPT:0111A	Immunization administration by intramuscular injection of severe acute respiratory syndrome coronavirus 2 (SARS-

			CoV-2) (coronavirus disease [COVID-19]) vaccine, mRNA-LNP, spike protein, preservative free, 25 mcg/0.25 mL dosage; first dose
or	procedure	UMLS:CPT:0051A	Immunization administration by intramuscular injection of severe acute respiratory syndrome coronavirus 2 (SARS-CoV-2) (coronavirus disease [COVID-19]) vaccine, mRNA-LNP, spike protein, preservative free, 30 mcg/0.3 mL dosage, tris-sucrose formulation; first dose
or	procedure	UMLS:CPT:91311	Severe acute respiratory syndrome coronavirus 2 (SARS-CoV-2) (coronavirus disease [COVID-19]) vaccine, mRNA-LNP, spike protein, preservative free, 25 mcg/0.25 mL dosage, for intramuscular use
or	procedure	UMLS:CPT:0074A	Immunization administration by intramuscular injection of severe acute respiratory syndrome coronavirus 2 (SARS-CoV-2) (coronavirus disease [COVID-19]) vaccine, mRNA-LNP, spike protein, preservative free, 10 mcg/0.2 mL dosage, diluent reconstituted, tris-sucrose formulation; booster dose
or	procedure	UMLS:CPT:0112A	Immunization administration by intramuscular injection of severe acute respiratory syndrome coronavirus 2 (SARS-CoV-2) (coronavirus disease

			[COVID-19]) vaccine, mRNA-LNP, spike protein, preservative free, 25 mcg/0.25 mL dosage; second dose
or	procedure	UMLS:CPT:0083A	Immunization administration by intramuscular injection of severe acute respiratory syndrome coronavirus 2 (SARS-CoV-2) (coronavirus disease [COVID-19]) vaccine, mRNA-LNP, spike protein, preservative free, 3 mcg/0.2 mL dosage, diluent reconstituted, tris-sucrose formulation; third dose
or	procedure	UMLS:CPT:0073A	Immunization administration by intramuscular injection of severe acute respiratory syndrome coronavirus 2 (SARS-CoV-2) (coronavirus disease [COVID-19]) vaccine, mRNA-LNP, spike protein, preservative free, 10 mcg/0.2 mL dosage, diluent reconstituted, tris-sucrose formulation; third dose
or	procedure	UMLS:CPT:0173A	Immunization administration by intramuscular injection of severe acute respiratory syndrome coronavirus 2 (SARS-CoV-2) (coronavirus disease [COVID-19]) vaccine, mRNA-LNP, bivalent spike protein, preservative free, 3 mcg/0.2 mL dosage, diluent reconstituted, tris-sucrose formulation, third dose
or	procedure	UMLS:CPT:0164A	Immunization administration by intramuscular injection of severe acute respiratory

			syndrome coronavirus 2 (SARS-CoV-2) (coronavirus disease [COVID-19]) vaccine, mRNA-LNP, spike protein, bivalent, preservative free, 10 mcg/0.2 mL dosage, booster dose
or	procedure	UMLS:CPT:1037838	Immunization administration by intramuscular injection of severe acute respiratory syndrome coronavirus 2 (SARS-CoV-2) (coronavirus disease [COVID-19]) vaccine, mRNA-LNP, spike protein, preservative free, 50 mcg/0.5 mL dosage
or	procedure	UMLS:CPT:0094A	Immunization administration by intramuscular injection of severe acute respiratory syndrome coronavirus 2 (SARS-CoV-2) (coronavirus disease [COVID-19]) vaccine, mRNA-LNP, spike protein, preservative free, 50 mcg/0.5 mL dosage; booster dose, when administered to individuals 18 years and over
or	procedure	UMLS:CPT:0034A	Immunization administration by intramuscular injection of severe acute respiratory syndrome coronavirus 2 (SARS-CoV-2) (coronavirus disease [COVID-19]) vaccine, DNA, spike protein, adenovirus type 26 (Ad26) vector, preservative free, 5x1010 viral particles/0.5 mL dosage; booster dose
or	procedure	UMLS:CPT:0144A	Immunization administration by intramuscular injection of severe acute respiratory

			syndrome coronavirus 2 (SARS-CoV-2) (coronavirus disease [COVID-19]) vaccine, mRNA-LNP, spike protein, bivalent, preservative free, 25 mcg/0.25 mL dosage, booster dose
or	procedure	UMLS:CPT:0091A	Immunization administration by intramuscular injection of severe acute respiratory syndrome coronavirus 2 (SARS-CoV-2) (coronavirus disease [COVID-19]) vaccine, mRNA-LNP, spike protein, preservative free, 50 mcg/0.5 mL dosage; first dose, when administered to individuals 6 through 11 years
or	procedure	UMLS:CPT:0174A	Immunization administration by intramuscular injection of severe acute respiratory syndrome coronavirus 2 (SARS-CoV-2) (coronavirus disease [COVID-19]) vaccine, mRNA-LNP, bivalent spike protein, preservative free, 3 mcg/0.2 mL dosage, diluent reconstituted, tris-sucrose formulation, booster
or	procedure	UMLS:CPT:0092A	Immunization administration by intramuscular injection of severe acute respiratory syndrome coronavirus 2 (SARS-CoV-2) (coronavirus disease [COVID-19]) vaccine, mRNA-LNP, spike protein, preservative free, 50 mcg/0.5 mL dosage; second dose, when administered to individuals 6 through 11 years

or	procedure	UMLS:CPT:1036682	Immunization administration by intramuscular injection of severe acute respiratory syndrome coronavirus 2 (SARS-CoV-2) (coronavirus disease [COVID-19]) vaccine, recombinant spike protein nanoparticle, saponin-based adjuvant, preservative free, 5 mcg/0.5 mL dosage
or	procedure	UMLS:CPT:0041A	Immunization administration by intramuscular injection of severe acute respiratory syndrome coronavirus 2 (SARS-CoV-2) (coronavirus disease [COVID-19]) vaccine, recombinant spike protein nanoparticle, saponin-based adjuvant, preservative free, 5 mcg/0.5 mL dosage; first dose
or	procedure	UMLS:CPT:0113A	Immunization administration by intramuscular injection of severe acute respiratory syndrome coronavirus 2 (SARS-CoV-2) (coronavirus disease [COVID-19]) vaccine, mRNA-LNP, spike protein, preservative free, 25 mcg/0.25 mL dosage; third dose
or	procedure	UMLS:CPT:0042A	Immunization administration by intramuscular injection of severe acute respiratory syndrome coronavirus 2 (SARS-CoV-2) (coronavirus disease [COVID-19]) vaccine, recombinant spike protein nanoparticle, saponin-based adjuvant, preservative free, 5

				mcg/0.5 mL dosage; second dose
	or	procedure	UMLS:CPT:0093A	Immunization administration by intramuscular injection of severe acute respiratory syndrome coronavirus 2 (SARS-CoV-2) (coronavirus disease [COVID-19]) vaccine, mRNA-LNP, spike protein, preservative free, 50 mcg/0.5 mL dosage; third dose, when administered to individuals 6 through 11 years
	or	procedure	UMLS:CPT:1036666	Immunization administration by intramuscular injection of severe acute respiratory syndrome coronavirus 2 (SARS-CoV-2) (coronavirus disease [COVID-19]) vaccine, DNA, spike protein, chimpanzee adenovirus Oxford 1 (ChAdOx1) vector, preservative free, 5x1010 viral particles/0.5 mL dosage
	or	procedure	UMLS:CPT:0044A	Immunization administration by intramuscular injection of severe acute respiratory syndrome coronavirus 2 (SARS-CoV-2) (coronavirus disease [COVID-19]) vaccine, recombinant spike protein nanoparticle, saponin-based adjuvant, preservative free, 5 mcg/0.5mL dosage; booster
date constraint	The terms in this group occurred between Dec 1, 2019 and Dec 31, 2023			
event relationship	Any instance of paxlovid occurred within 7 days on or after any instance of COVID-19, no vaccination			
**Group 1B paxlovid**				
must have		medication	NLM:RXNORM:2587892	nirmatrelvir

	and	medication	NLM:RXNORM:85762	ritonavir
**Group 2**				
**Group 2A COVID-19**				
must have	any of	diagnosis	UMLS:ICD10CM:U07.1	COVID-19
		diagnosis	UMLS:ICD10CM:U07.2	COVID-19, virus not identified (WHO)
		diagnosis	UMLS:ICD10CM:J12.82	Pneumonia due to coronavirus disease 2019
		laboratory	UMLS:LNC:94500-6	SARS-CoV-2 (COVID-19) RNA [Presence] in Respiratory specimen by NAA with probe detection (labResult: Positive)
		laboratory	UMLS:LNC:94309-2	SARS-CoV-2 (COVID-19) RNA [Presence] in Specimen by NAA with probe detection (labResult: Positive)
		laboratory	UMLS:LNC:94565-9	SARS-CoV-2 (COVID-19) RNA [Presence] in Nasopharynx by NAA with non-probe detection (labResult: Positive)
		laboratory	UMLS:LNC:94759-8	SARS-CoV-2 (COVID-19) RNA [Presence] in Nasopharynx by NAA with probe detection (labResult: Positive)
		laboratory	UMLS:LNC:95608-6	SARS-CoV-2 (COVID-19) RNA [Presence] in Respiratory specimen by NAA with non-probe detection (labResult: Positive)
		laboratory	UMLS:LNC:94845-5	SARS-CoV-2 (COVID-19) RNA [Presence] in Saliva (oral fluid) by NAA with probe detection (labResult: Positive)
		laboratory	UMLS:LNC:95406-5	SARS-CoV-2 (COVID-19) RNA [Presence] in Nose by NAA with probe detection (labResult: Positive)

	and	visit	TNX:Visit	Visit (Data Source: TriNetX)
date constraint		The terms in this group occurred at any time		
event relationship		Any instance of thyroid cancer history occurred at least 1 day before the first instance of COVID-19		
**Group 2B thyroid cancer history**				
cannot have		diagnosis	UMLS:ICD10CM:C73	Malignant neoplasm of thyroid gland
	or	diagnosis	UMLS:ICD10CM:Z85.850	Personal history of malignant neoplasm of thyroid

Query Criteria for Cohort post-COVID s/p Molnupiravir

This query was run on the network Global Collaborative Network with 148 HCO(s) queried and 148 HCO(s) responded. A total of 53 provider(s) responded with patients. The final cohort included 24,535 patients who matched the query criteria listed in the table below.

**Ungrouped terms**				
must have		demographics	Age	Age (at least 18 years (most recent occurrence))
	and any of	demographics	UMLS:HL7V3.0:Gender:M	Male
		demographics	UMLS:HL7V3.0:Gender:F	Female
**Group 1**				
**Group 1A COVID-19, no vaccination**				
must have	any of	diagnosis	UMLS:ICD10CM:U07.1	COVID-19
		diagnosis	UMLS:ICD10CM:U07.2	COVID-19, virus not identified (WHO)
		diagnosis	UMLS:ICD10CM:J12.82	Pneumonia due to coronavirus disease 2019
		laboratory	UMLS:LNC:94500-6	SARS-CoV-2 (COVID-19) RNA [Presence] in Respiratory specimen by NAA with probe detection (labResult: Positive)
		laboratory	UMLS:LNC:94309-2	SARS-CoV-2 (COVID-19) RNA [Presence] in Specimen by NAA with probe detection (labResult: Positive)

		laboratory	UMLS:LNC:94565-9	SARS-CoV-2 (COVID-19) RNA [Presence] in Nasopharynx by NAA with non-probe detection (labResult: Positive)
		laboratory	UMLS:LNC:94759-8	SARS-CoV-2 (COVID-19) RNA [Presence] in Nasopharynx by NAA with probe detection (labResult: Positive)
		laboratory	UMLS:LNC:95608-6	SARS-CoV-2 (COVID-19) RNA [Presence] in Respiratory specimen by NAA with non-probe detection (labResult: Positive)
		laboratory	UMLS:LNC:94845-5	SARS-CoV-2 (COVID-19) RNA [Presence] in Saliva (oral fluid) by NAA with probe detection (labResult: Positive)
		laboratory	UMLS:LNC:95406-5	SARS-CoV-2 (COVID-19) RNA [Presence] in Nose by NAA with probe detection (labResult: Positive)
cannot have		medication	NLM:CVX:208	COVID-19, mRNA, LNP-S, PF, 30 mcg/0.3 mL dose
	or	medication	NLM:CVX:207	COVID-19, mRNA, LNP-S, PF, 100 mcg/0.5mL dose or 50 mcg/0.25mL dose
	or	medication	NLM:CVX:212	COVID-19 vaccine, vector-nr, rS-Ad26, PF, 0.5 mL
	or	medication	NLM:RXNORM:OMOP 5042939	COVID-19 vaccine
	or	medication	NLM:CVX:300	COVID-19, mRNA, LNP-S, bivalent, PF, 30 mcg/0.3 mL dose
	or	medication	NLM:CVX:217	COVID-19, mRNA, LNP-S, PF, 30 mcg/0.3 mL dose, tris-sucrose
	or	medication	NLM:CVX:229	COVID-19, mRNA, LNP-S, bivalent, PF, 50 mcg/0.5 mL or 25mcg/0.25 mL dose

or	medication	NLM:CVX:218	COVID-19, mRNA, LNP-S, PF, 10 mcg/0.2 mL dose, tris-sucrose
or	medication	NLM:CVX:520	COVID-19 mRNA, bivalent, original/Omicron BA.1, Non-US Vaccine Product, Pfizer-BioNTech
or	medication	NLM:CVX:519	COVID-19 mRNA, bivalent, original/Omicron BA.1, Non-US Vaccine (Spikevax Bivalent), Moderna
or	medication	NLM:CVX:301	COVID-19, mRNA, LNP-S, bivalent, PF, 10 mcg/0.2 mL dose
or	medication	NLM:CVX:219	COVID-19, mRNA, LNP-S, PF, 3 mcg/0.2 mL dose, tris-sucrose
or	medication	NLM:CVX:228	COVID-19, mRNA, LNP-S, PF, pediatric 25 mcg/0.25 mL dose
or	medication	NLM:CVX:230	COVID-19, mRNA, LNP-S, bivalent booster, PF, 10 mcg/0.2 mL
or	medication	NLM:CVX:221	COVID-19, mRNA, LNP-S, PF, 50 mcg/0.5 mL dose
or	medication	NLM:CVX:210	COVID-19 vaccine, vector-nr, rS-ChAdOx1, PF, 0.5 mL
or	medication	NLM:CVX:302	COVID-19, mRNA, LNP-S, bivalent, PF, 3 mcg/0.2 mL dose
or	medication	NLM:CVX:511	COVID-19 IV Non-US Vaccine (CoronaVac, Sinovac)
or	medication	NLM:RXNORM:2468231	SARS-CoV-2 (COVID-19) vaccine, mRNA spike protein
or	procedure	UMLS:CPT:91300	Severe acute respiratory syndrome coronavirus 2 (SARS-CoV-2) (coronavirus disease [COVID-19]) vaccine, mRNA-LNP, spike protein, preservative free, 30 mcg/0.3 mL dosage, diluent reconstituted, for intramuscular use

or	procedure	UMLS:CPT:0001A	Immunization administration by intramuscular injection of severe acute respiratory syndrome coronavirus 2 (SARS-CoV-2) (coronavirus disease [COVID-19]) vaccine, mRNA-LNP, spike protein, preservative free, 30 mcg/0.3 mL dosage, diluent reconstituted; first dose
or	procedure	UMLS:CPT:0002A	Immunization administration by intramuscular injection of severe acute respiratory syndrome coronavirus 2 (SARS-CoV-2) (coronavirus disease [COVID-19]) vaccine, mRNA-LNP, spike protein, preservative free, 30 mcg/0.3 mL dosage, diluent reconstituted; second dose
or	procedure	UMLS:CPT:91301	Severe acute respiratory syndrome coronavirus 2 (SARS-CoV-2) (coronavirus disease [COVID-19]) vaccine, mRNA-LNP, spike protein, preservative free, 100 mcg/0.5 mL dosage, for intramuscular use
or	procedure	UMLS:CPT:0011A	Immunization administration by intramuscular injection of severe acute respiratory syndrome coronavirus 2 (SARS-CoV-2) (coronavirus disease [COVID-19]) vaccine, mRNA-LNP, spike protein, preservative free, 100 mcg/0.5 mL dosage; first dose
or	procedure	UMLS:CPT:0012A	Immunization administration by intramuscular injection of severe acute respiratory

			syndrome coronavirus 2 (SARS-CoV-2) (coronavirus disease [COVID-19]) vaccine, mRNA-LNP, spike protein, preservative free, 100 mcg/0.5 mL dosage; second dose
or	procedure	UMLS:SNOMED:840534001	Administration of SARS-CoV-2 antigen vaccine
or	medication	NLM:CVX:213	SARS-CoV-2 (COVID-19) Vaccine
or	procedure	UMLS:CPT:1036660	Immunization administration by intramuscular injection of severe acute respiratory syndrome coronavirus 2 (SARS-CoV-2) (coronavirus disease [COVID-19]) vaccine, mRNA-LNP, spike protein, preservative free, 30 mcg/0.3 mL dosage, diluent reconstituted
or	procedure	UMLS:CPT:1036663	Immunization administration by intramuscular injection of severe acute respiratory syndrome coronavirus 2 (SARS-CoV-2) (coronavirus disease [COVID-19]) vaccine, mRNA-LNP, spike protein, preservative free, 100 mcg/0.5 mL dosage
or	procedure	UMLS:CPT:0124A	Immunization administration by intramuscular injection of severe acute respiratory syndrome coronavirus 2 (SARS-CoV-2) (coronavirus disease [COVID-19]) vaccine, mRNA-LNP, bivalent spike protein, preservative free, 30 mcg/0.3 mL dosage, tris-sucrose formulation, booster dose

or	procedure	UMLS:CPT:0004A	Immunization administration by intramuscular injection of severe acute respiratory syndrome coronavirus 2 (SARS-CoV-2) (coronavirus disease [COVID-19]) vaccine, mRNA-LNP, spike protein, preservative free, 30 mcg/0.3 mL dosage, diluent reconstituted; booster dose
or	procedure	UMLS:CPT:0003A	Immunization administration by intramuscular injection of severe acute respiratory syndrome coronavirus 2 (SARS-CoV-2) (coronavirus disease [COVID-19]) vaccine, mRNA-LNP, spike protein, preservative free, 30 mcg/0.3 mL dosage, diluent reconstituted; third dose
or	procedure	UMLS:CPT:1037166	Immunization administration by intramuscular injection of severe acute respiratory syndrome coronavirus 2 (SARS-CoV-2) (coronavirus disease [COVID-19]) vaccine, mRNA-LNP, spike protein, preservative free, 30 mcg/0.3 mL dosage, tris-sucrose formulation
or	procedure	UMLS:CPT:0054A	Immunization administration by intramuscular injection of severe acute respiratory syndrome coronavirus 2 (SARS-CoV-2) (coronavirus disease [COVID-19]) vaccine, mRNA-LNP, spike protein, preservative free, 30 mcg/0.3 mL dosage, tris-sucrose formulation; booster dose

or	procedure	UMLS:CPT:0064A	Immunization administration by intramuscular injection of severe acute respiratory syndrome coronavirus 2 (SARS-CoV-2) (coronavirus disease [COVID-19]) vaccine, mRNA-LNP, spike protein, preservative free, 50 mcg/0.25 mL dosage, booster dose
or	procedure	UMLS:CPT:90480	Immunization administration by intramuscular injection of severe acute respiratory syndrome coronavirus 2 (SARS-CoV-2) (coronavirus disease [COVID-19]) vaccine, single dose
or	procedure	UMLS:CPT:1037171	Immunization administration by intramuscular injection of severe acute respiratory syndrome coronavirus 2 (SARS-CoV-2) (coronavirus disease [COVID-19]) vaccine, mRNA-LNP, spike protein, preservative free, 10 mcg/0.2 mL dosage, diluent reconstituted, tris-sucrose formulation
or	procedure	UMLS:CPT:0071A	Immunization administration by intramuscular injection of severe acute respiratory syndrome coronavirus 2 (SARS-CoV-2) (coronavirus disease [COVID-19]) vaccine, mRNA-LNP, spike protein, preservative free, 10 mcg/0.2 mL dosage, diluent reconstituted, tris-sucrose formulation; first dose
or	procedure	UMLS:CPT:0072A	Immunization administration by intramuscular injection of

			severe acute respiratory syndrome coronavirus 2 (SARS-CoV-2) (coronavirus disease [COVID-19]) vaccine, mRNA-LNP, spike protein, preservative free, 10 mcg/0.2 mL dosage, diluent reconstituted, tris-sucrose formulation; second dose
or	medication	NLM:RXNORM:2610319	SARS-CoV-2 (COVID-19) vaccine, mRNA-BNT162b2 0.05 MG/ML / SARS-CoV-2 (COVID-19) vaccine, mRNA-BNT162b2 OMICRON (BA.4/BA.5) 0.05 MG/ML Injectable Suspension
or	procedure	UMLS:CPT:91313	Severe acute respiratory syndrome coronavirus 2 (SARS-CoV-2) (coronavirus disease [COVID-19]) vaccine, mRNA-LNP, spike protein, bivalent, preservative free, 50 mcg/0.5 mL dosage, for intramuscular use
or	procedure	UMLS:CPT:0134A	Immunization administration by intramuscular injection of severe acute respiratory syndrome coronavirus 2 (SARS-CoV-2) (coronavirus disease [COVID-19]) vaccine, mRNA-LNP, spike protein, bivalent, preservative free, 50 mcg/0.5 mL dosage, booster dose
or	procedure	UMLS:CPT:1037175	Immunization administration by intramuscular injection of severe acute respiratory syndrome coronavirus 2 (SARS-CoV-2) (coronavirus disease [COVID-19]) vaccine, DNA, spike protein, adenovirus type 26

			(Ad26) vector, preservative free, 5x1010 viral particles/0.5 mL dosage
or	medication	NLM:RXNORM:2610347	0.3 ML SARS-CoV-2 (COVID-19) vaccine, mRNA-BNT162b2 0.05 MG/ML / SARS-CoV-2 (COVID-19) vaccine, mRNA-BNT162b2 OMICRON (BA.4/BA.5) -1 MG/ML Injection
or	procedure	UMLS:CPT:1037228	Immunization administration by intramuscular injection of severe acute respiratory syndrome coronavirus 2 (SARS-CoV-2) (coronavirus disease [COVID-19]) vaccine, mRNA-LNP, spike protein, preservative free, 3 mcg/0.2 mL dosage, diluent reconstituted, tris-sucrose formulation
or	procedure	UMLS:CPT:0013A	Immunization administration by intramuscular injection of severe acute respiratory syndrome coronavirus 2 (SARS-CoV-2) (coronavirus disease [COVID-19]) vaccine, mRNA-LNP, spike protein, preservative free, 100 mcg/0.5 mL dosage; third dose
or	procedure	UMLS:CPT:0081A	Immunization administration by intramuscular injection of severe acute respiratory syndrome coronavirus 2 (SARS-CoV-2) (coronavirus disease [COVID-19]) vaccine, mRNA-LNP, spike protein, preservative free, 3 mcg/0.2 mL dosage, diluent reconstituted, tris-sucrose formulation; first dose

or	procedure	UMLS:CPT:0082A	Immunization administration by intramuscular injection of severe acute respiratory syndrome coronavirus 2 (SARS-CoV-2) (coronavirus disease [COVID-19]) vaccine, mRNA-LNP, spike protein, preservative free, 3 mcg/0.2 mL dosage, diluent reconstituted, tris-sucrose formulation; second dose
or	medication	NLM:RXNORM:2610328	SARS-CoV-2 (COVID-19) vaccine, mRNA-1273 0.05 MG/ML / SARS-CoV-2 (COVID-19) vaccine, mRNA-1273 OMICRON (BA.4/BA.5) 0.05 MG/ML Injectable Suspension
or	procedure	UMLS:CPT:0154A	Immunization administration by intramuscular injection of severe acute respiratory syndrome coronavirus 2 (SARS-CoV-2) (coronavirus disease [COVID-19]) vaccine, mRNA-LNP, bivalent spike protein, preservative free, 10 mcg/0.2 mL dosage, diluent reconstituted, tris-sucrose formulation, booster dose
or	procedure	UMLS:CPT:0053A	Immunization administration by intramuscular injection of severe acute respiratory syndrome coronavirus 2 (SARS-CoV-2) (coronavirus disease [COVID-19]) vaccine, mRNA-LNP, spike protein, preservative free, 30 mcg/0.3 mL dosage, tris-sucrose formulation; third dose

or	procedure	UMLS:CPT:1037332	Immunization administration by intramuscular injection of severe acute respiratory syndrome coronavirus 2 (SARS-CoV-2) (coronavirus disease [COVID-19]) vaccine, mRNA-LNP, spike protein, preservative free, 25 mcg/0.25 mL dosage
or	procedure	UMLS:CPT:0052A	Immunization administration by intramuscular injection of severe acute respiratory syndrome coronavirus 2 (SARS-CoV-2) (coronavirus disease [COVID-19]) vaccine, mRNA-LNP, spike protein, preservative free, 30 mcg/0.3 mL dosage, tris-sucrose formulation; second dose
or	procedure	UMLS:CPT:0111A	Immunization administration by intramuscular injection of severe acute respiratory syndrome coronavirus 2 (SARS-CoV-2) (coronavirus disease [COVID-19]) vaccine, mRNA-LNP, spike protein, preservative free, 25 mcg/0.25 mL dosage; first dose
or	procedure	UMLS:CPT:0051A	Immunization administration by intramuscular injection of severe acute respiratory syndrome coronavirus 2 (SARS-CoV-2) (coronavirus disease [COVID-19]) vaccine, mRNA-LNP, spike protein, preservative free, 30 mcg/0.3 mL dosage, tris-sucrose formulation; first dose

or	procedure	UMLS:CPT:91311	Severe acute respiratory syndrome coronavirus 2 (SARS-CoV-2) (coronavirus disease [COVID-19]) vaccine, mRNA-LNP, spike protein, preservative free, 25 mcg/0.25 mL dosage, for intramuscular use
or	procedure	UMLS:CPT:0074A	Immunization administration by intramuscular injection of severe acute respiratory syndrome coronavirus 2 (SARS-CoV-2) (coronavirus disease [COVID-19]) vaccine, mRNA-LNP, spike protein, preservative free, 10 mcg/0.2 mL dosage, diluent reconstituted, tris-sucrose formulation; booster dose
or	procedure	UMLS:CPT:0112A	Immunization administration by intramuscular injection of severe acute respiratory syndrome coronavirus 2 (SARS-CoV-2) (coronavirus disease [COVID-19]) vaccine, mRNA-LNP, spike protein, preservative free, 25 mcg/0.25 mL dosage; second dose
or	procedure	UMLS:CPT:0083A	Immunization administration by intramuscular injection of severe acute respiratory syndrome coronavirus 2 (SARS-CoV-2) (coronavirus disease [COVID-19]) vaccine, mRNA-LNP, spike protein, preservative free, 3 mcg/0.2 mL dosage, diluent reconstituted, tris-sucrose formulation; third dose

or	procedure	UMLS:CPT:0073A	Immunization administration by intramuscular injection of severe acute respiratory syndrome coronavirus 2 (SARS-CoV-2) (coronavirus disease [COVID-19]) vaccine, mRNA-LNP, spike protein, preservative free, 10 mcg/0.2 mL dosage, diluent reconstituted, tris-sucrose formulation; third dose
or	procedure	UMLS:CPT:0173A	Immunization administration by intramuscular injection of severe acute respiratory syndrome coronavirus 2 (SARS-CoV-2) (coronavirus disease [COVID-19]) vaccine, mRNA-LNP, bivalent spike protein, preservative free, 3 mcg/0.2 mL dosage, diluent reconstituted, tris-sucrose formulation, third dose
or	procedure	UMLS:CPT:0164A	Immunization administration by intramuscular injection of severe acute respiratory syndrome coronavirus 2 (SARS-CoV-2) (coronavirus disease [COVID-19]) vaccine, mRNA-LNP, spike protein, bivalent, preservative free, 10 mcg/0.2 mL dosage, booster dose
or	procedure	UMLS:CPT:1037838	Immunization administration by intramuscular injection of severe acute respiratory syndrome coronavirus 2 (SARS-CoV-2) (coronavirus disease [COVID-19]) vaccine, mRNA-LNP, spike protein, preservative free, 50 mcg/0.5 mL dosage

or	procedure	UMLS:CPT:0094A	Immunization administration by intramuscular injection of severe acute respiratory syndrome coronavirus 2 (SARS-CoV-2) (coronavirus disease [COVID-19]) vaccine, mRNA-LNP, spike protein, preservative free, 50 mcg/0.5 mL dosage; booster dose, when administered to individuals 18 years and over
or	procedure	UMLS:CPT:0034A	Immunization administration by intramuscular injection of severe acute respiratory syndrome coronavirus 2 (SARS-CoV-2) (coronavirus disease [COVID-19]) vaccine, DNA, spike protein, adenovirus type 26 (Ad26) vector, preservative free, 5x1010 viral particles/0.5 mL dosage; booster dose
or	procedure	UMLS:CPT:0144A	Immunization administration by intramuscular injection of severe acute respiratory syndrome coronavirus 2 (SARS-CoV-2) (coronavirus disease [COVID-19]) vaccine, mRNA-LNP, spike protein, bivalent, preservative free, 25 mcg/0.25 mL dosage, booster dose
or	procedure	UMLS:CPT:0091A	Immunization administration by intramuscular injection of severe acute respiratory syndrome coronavirus 2 (SARS-CoV-2) (coronavirus disease [COVID-19]) vaccine, mRNA-LNP, spike protein, preservative free, 50 mcg/0.5 mL dosage;

			first dose, when administered to individuals 6 through 11 years
or	procedure	UMLS:CPT:0174A	Immunization administration by intramuscular injection of severe acute respiratory syndrome coronavirus 2 (SARS-CoV-2) (coronavirus disease [COVID-19]) vaccine, mRNA-LNP, bivalent spike protein, preservative free, 3 mcg/0.2 mL dosage, diluent reconstituted, tris-sucrose formulation, booster
or	procedure	UMLS:CPT:0092A	Immunization administration by intramuscular injection of severe acute respiratory syndrome coronavirus 2 (SARS-CoV-2) (coronavirus disease [COVID-19]) vaccine, mRNA-LNP, spike protein, preservative free, 50 mcg/0.5 mL dosage; second dose, when administered to individuals 6 through 11 years
or	procedure	UMLS:CPT:1036682	Immunization administration by intramuscular injection of severe acute respiratory syndrome coronavirus 2 (SARS-CoV-2) (coronavirus disease [COVID-19]) vaccine, recombinant spike protein nanoparticle, saponin-based adjuvant, preservative free, 5 mcg/0.5 mL dosage
or	procedure	UMLS:CPT:0041A	Immunization administration by intramuscular injection of severe acute respiratory syndrome coronavirus 2 (SARS-

			CoV-2) (coronavirus disease [COVID-19]) vaccine, recombinant spike protein nanoparticle, saponin-based adjuvant, preservative free, 5 mcg/0.5 mL dosage; first dose
or	procedure	UMLS:CPT:0113A	Immunization administration by intramuscular injection of severe acute respiratory syndrome coronavirus 2 (SARS-CoV-2) (coronavirus disease [COVID-19]) vaccine, mRNA-LNP, spike protein, preservative free, 25 mcg/0.25 mL dosage; third dose
or	procedure	UMLS:CPT:0042A	Immunization administration by intramuscular injection of severe acute respiratory syndrome coronavirus 2 (SARS-CoV-2) (coronavirus disease [COVID-19]) vaccine, recombinant spike protein nanoparticle, saponin-based adjuvant, preservative free, 5 mcg/0.5 mL dosage; second dose
or	procedure	UMLS:CPT:0093A	Immunization administration by intramuscular injection of severe acute respiratory syndrome coronavirus 2 (SARS-CoV-2) (coronavirus disease [COVID-19]) vaccine, mRNA-LNP, spike protein, preservative free, 50 mcg/0.5 mL dosage; third dose, when administered to individuals 6 through 11 years
or	procedure	UMLS:CPT:1036666	Immunization administration by intramuscular injection of

				severe acute respiratory syndrome coronavirus 2 (SARS-CoV-2) (coronavirus disease [COVID-19]) vaccine, DNA, spike protein, chimpanzee adenovirus Oxford 1 (ChAdOx1) vector, preservative free, 5x1010 viral particles/0.5 mL dosage
	or	procedure	UMLS:CPT:0044A	Immunization administration by intramuscular injection of severe acute respiratory syndrome coronavirus 2 (SARS-CoV-2) (coronavirus disease [COVID-19]) vaccine, recombinant spike protein nanoparticle, saponin-based adjuvant, preservative free, 5 mcg/0.5mL dosage; booster
date constraint		The terms in this group occurred between Dec 1, 2019 and Dec 31, 2023		
event relationship		Any instance of Molnupiravir occurred within 7 days on or after any instance of COVID-19, no vaccination		
**Group 1B Molnupiravir**				
must have		medication	NLM:RXNORM:2587901	molnupiravir
**Group 2**				
**Group 2A COVID-19**				
must have	any of	diagnosis	UMLS:ICD10CM:U07.1	COVID-19
		diagnosis	UMLS:ICD10CM:U07.2	COVID-19, virus not identified (WHO)
		diagnosis	UMLS:ICD10CM:J12.82	Pneumonia due to coronavirus disease 2019
		laboratory	UMLS:LNC:94500-6	SARS-CoV-2 (COVID-19) RNA [Presence] in Respiratory specimen by NAA with probe detection (labResult: Positive)
		laboratory	UMLS:LNC:94309-2	SARS-CoV-2 (COVID-19) RNA [Presence] in Specimen by NAA

				with probe detection (labResult: Positive)
		laboratory	UMLS:LNC:94565-9	SARS-CoV-2 (COVID-19) RNA [Presence] in Nasopharynx by NAA with non-probe detection (labResult: Positive)
		laboratory	UMLS:LNC:94759-8	SARS-CoV-2 (COVID-19) RNA [Presence] in Nasopharynx by NAA with probe detection (labResult: Positive)
		laboratory	UMLS:LNC:95608-6	SARS-CoV-2 (COVID-19) RNA [Presence] in Respiratory specimen by NAA with non-probe detection (labResult: Positive)
		laboratory	UMLS:LNC:94845-5	SARS-CoV-2 (COVID-19) RNA [Presence] in Saliva (oral fluid) by NAA with probe detection (labResult: Positive)
		laboratory	UMLS:LNC:95406-5	SARS-CoV-2 (COVID-19) RNA [Presence] in Nose by NAA with probe detection (labResult: Positive)
	and	visit	TNX:Visit	Visit (Data Source: TriNetX)
date constraint		The terms in this group occurred at any time		
event relationship		Any instance of thyroid cancer history occurred at least 1 day before the first instance of COVID-19		
**Group 2B thyroid cancer history**				
cannot have		diagnosis	UMLS:ICD10CM:C73	Malignant neoplasm of thyroid gland
	or	diagnosis	UMLS:ICD10CM:Z85.850	Personal history of malignant neoplasm of thyroid

Query Criteria for Cohort post-COVID s/p Remdesivir

This query was run on the network Global Collaborative Network with 148 HCO(s) queried and 148 HCO(s) responded. A total of 83 provider(s) responded with patients. The final cohort included 92,299 patients who matched the query criteria listed in the table below.

**Ungrouped terms**				
must have		demographics	Age	Age (at least 18 years (most recent occurrence))
	and any of	demographics	UMLS:HL7V3.0:Gender:M	Male
		demographics	UMLS:HL7V3.0:Gender:F	Female
**Group 1**				
**Group 1A COVID-19, no vaccination**				
must have	any of	diagnosis	UMLS:ICD10CM:U07.1	COVID-19
		diagnosis	UMLS:ICD10CM:U07.2	COVID-19, virus not identified (WHO)
		diagnosis	UMLS:ICD10CM:J12.82	Pneumonia due to coronavirus disease 2019
		laboratory	UMLS:LNC:94500-6	SARS-CoV-2 (COVID-19) RNA [Presence] in Respiratory specimen by NAA with probe detection (labResult: Positive)
		laboratory	UMLS:LNC:94309-2	SARS-CoV-2 (COVID-19) RNA [Presence] in Specimen by NAA with probe detection (labResult: Positive)
		laboratory	UMLS:LNC:94565-9	SARS-CoV-2 (COVID-19) RNA [Presence] in Nasopharynx by NAA with non-probe detection (labResult: Positive)
		laboratory	UMLS:LNC:94759-8	SARS-CoV-2 (COVID-19) RNA [Presence] in Nasopharynx by NAA with probe detection (labResult: Positive)
		laboratory	UMLS:LNC:95608-6	SARS-CoV-2 (COVID-19) RNA [Presence] in Respiratory specimen by NAA with non-probe detection (labResult: Positive)
		laboratory	UMLS:LNC:94845-5	SARS-CoV-2 (COVID-19) RNA [Presence] in Saliva (oral fluid)

				by NAA with probe detection (labResult: Positive)
		laboratory	UMLS:LNC:95406-5	SARS-CoV-2 (COVID-19) RNA [Presence] in Nose by NAA with probe detection (labResult: Positive)
cannot have		medication	NLM:CVX:208	COVID-19, mRNA, LNP-S, PF, 30 mcg/0.3 mL dose
	or	medication	NLM:CVX:207	COVID-19, mRNA, LNP-S, PF, 100 mcg/0.5mL dose or 50 mcg/0.25mL dose
	or	medication	NLM:CVX:212	COVID-19 vaccine, vector-nr, rS-Ad26, PF, 0.5 mL
	or	medication	NLM:RXNORM:OMOP 5042939	COVID-19 vaccine
	or	medication	NLM:CVX:300	COVID-19, mRNA, LNP-S, bivalent, PF, 30 mcg/0.3 mL dose
	or	medication	NLM:CVX:217	COVID-19, mRNA, LNP-S, PF, 30 mcg/0.3 mL dose, tris-sucrose
	or	medication	NLM:CVX:229	COVID-19, mRNA, LNP-S, bivalent, PF, 50 mcg/0.5 mL or 25mcg/0.25 mL dose
	or	medication	NLM:CVX:218	COVID-19, mRNA, LNP-S, PF, 10 mcg/0.2 mL dose, tris-sucrose
	or	medication	NLM:CVX:520	COVID-19 mRNA, bivalent, original/Omicron BA.1, Non-US Vaccine Product, Pfizer-BioNTech
	or	medication	NLM:CVX:519	COVID-19 mRNA, bivalent, original/Omicron BA.1, Non-US Vaccine (Spikevax Bivalent), Moderna
	or	medication	NLM:CVX:301	COVID-19, mRNA, LNP-S, bivalent, PF, 10 mcg/0.2 mL dose
	or	medication	NLM:CVX:219	COVID-19, mRNA, LNP-S, PF, 3 mcg/0.2 mL dose, tris-sucrose

or	medication	NLM:CVX:228	COVID-19, mRNA, LNP-S, PF, pediatric 25 mcg/0.25 mL dose
or	medication	NLM:CVX:230	COVID-19, mRNA, LNP-S, bivalent booster, PF, 10 mcg/0.2 mL
or	medication	NLM:CVX:221	COVID-19, mRNA, LNP-S, PF, 50 mcg/0.5 mL dose
or	medication	NLM:CVX:210	COVID-19 vaccine, vector-nr, rS-ChAdOx1, PF, 0.5 mL
or	medication	NLM:CVX:302	COVID-19, mRNA, LNP-S, bivalent, PF, 3 mcg/0.2 mL dose
or	medication	NLM:CVX:511	COVID-19 IV Non-US Vaccine (CoronaVac, Sinovac)
or	medication	NLM:RXNORM:2468231	SARS-CoV-2 (COVID-19) vaccine, mRNA spike protein
or	procedure	UMLS:CPT:91300	Severe acute respiratory syndrome coronavirus 2 (SARS-CoV-2) (coronavirus disease [COVID-19]) vaccine, mRNA-LNP, spike protein, preservative free, 30 mcg/0.3 mL dosage, diluent reconstituted, for intramuscular use
or	procedure	UMLS:CPT:0001A	Immunization administration by intramuscular injection of severe acute respiratory syndrome coronavirus 2 (SARS-CoV-2) (coronavirus disease [COVID-19]) vaccine, mRNA-LNP, spike protein, preservative free, 30 mcg/0.3 mL dosage, diluent reconstituted; first dose
or	procedure	UMLS:CPT:0002A	Immunization administration by intramuscular injection of severe acute respiratory syndrome coronavirus 2 (SARS-CoV-2) (coronavirus disease [COVID-19]) vaccine, mRNA-

			LNP, spike protein, preservative free, 30 mcg/0.3 mL dosage, diluent reconstituted; second dose
or	procedure	UMLS:CPT:91301	Severe acute respiratory syndrome coronavirus 2 (SARS-CoV-2) (coronavirus disease [COVID-19]) vaccine, mRNA-LNP, spike protein, preservative free, 100 mcg/0.5 mL dosage, for intramuscular use
or	procedure	UMLS:CPT:0011A	Immunization administration by intramuscular injection of severe acute respiratory syndrome coronavirus 2 (SARS-CoV-2) (coronavirus disease [COVID-19]) vaccine, mRNA-LNP, spike protein, preservative free, 100 mcg/0.5 mL dosage; first dose
or	procedure	UMLS:CPT:0012A	Immunization administration by intramuscular injection of severe acute respiratory syndrome coronavirus 2 (SARS-CoV-2) (coronavirus disease [COVID-19]) vaccine, mRNA-LNP, spike protein, preservative free, 100 mcg/0.5 mL dosage; second dose
or	procedure	UMLS:SNOMED:840534001	Administration of SARS-CoV-2 antigen vaccine
or	medication	NLM:CVX:213	SARS-CoV-2 (COVID-19) Vaccine
or	procedure	UMLS:CPT:1036660	Immunization administration by intramuscular injection of severe acute respiratory syndrome coronavirus 2 (SARS-CoV-2) (coronavirus disease

			[COVID-19]) vaccine, mRNA-LNP, spike protein, preservative free, 30 mcg/0.3 mL dosage, diluent reconstituted
or	procedure	UMLS:CPT:1036663	Immunization administration by intramuscular injection of severe acute respiratory syndrome coronavirus 2 (SARS-CoV-2) (coronavirus disease [COVID-19]) vaccine, mRNA-LNP, spike protein, preservative free, 100 mcg/0.5 mL dosage
or	procedure	UMLS:CPT:0124A	Immunization administration by intramuscular injection of severe acute respiratory syndrome coronavirus 2 (SARS-CoV-2) (coronavirus disease [COVID-19]) vaccine, mRNA-LNP, bivalent spike protein, preservative free, 30 mcg/0.3 mL dosage, tris-sucrose formulation, booster dose
or	procedure	UMLS:CPT:0004A	Immunization administration by intramuscular injection of severe acute respiratory syndrome coronavirus 2 (SARS-CoV-2) (coronavirus disease [COVID-19]) vaccine, mRNA-LNP, spike protein, preservative free, 30 mcg/0.3 mL dosage, diluent reconstituted; booster dose
or	procedure	UMLS:CPT:0003A	Immunization administration by intramuscular injection of severe acute respiratory syndrome coronavirus 2 (SARS-CoV-2) (coronavirus disease [COVID-19]) vaccine, mRNA-

			LNP, spike protein, preservative free, 30 mcg/0.3 mL dosage, diluent reconstituted; third dose
or	procedure	UMLS:CPT:1037166	Immunization administration by intramuscular injection of severe acute respiratory syndrome coronavirus 2 (SARS-CoV-2) (coronavirus disease [COVID-19]) vaccine, mRNA-LNP, spike protein, preservative free, 30 mcg/0.3 mL dosage, tris-sucrose formulation
or	procedure	UMLS:CPT:0054A	Immunization administration by intramuscular injection of severe acute respiratory syndrome coronavirus 2 (SARS-CoV-2) (coronavirus disease [COVID-19]) vaccine, mRNA-LNP, spike protein, preservative free, 30 mcg/0.3 mL dosage, tris-sucrose formulation; booster dose
or	procedure	UMLS:CPT:0064A	Immunization administration by intramuscular injection of severe acute respiratory syndrome coronavirus 2 (SARS-CoV-2) (coronavirus disease [COVID-19]) vaccine, mRNA-LNP, spike protein, preservative free, 50 mcg/0.25 mL dosage, booster dose
or	procedure	UMLS:CPT:90480	Immunization administration by intramuscular injection of severe acute respiratory syndrome coronavirus 2 (SARS-CoV-2) (coronavirus disease [COVID-19]) vaccine, single dose

or	procedure	UMLS:CPT:1037171	Immunization administration by intramuscular injection of severe acute respiratory syndrome coronavirus 2 (SARS-CoV-2) (coronavirus disease [COVID-19]) vaccine, mRNA-LNP, spike protein, preservative free, 10 mcg/0.2 mL dosage, diluent reconstituted, tris-sucrose formulation
or	procedure	UMLS:CPT:0071A	Immunization administration by intramuscular injection of severe acute respiratory syndrome coronavirus 2 (SARS-CoV-2) (coronavirus disease [COVID-19]) vaccine, mRNA-LNP, spike protein, preservative free, 10 mcg/0.2 mL dosage, diluent reconstituted, tris-sucrose formulation; first dose
or	procedure	UMLS:CPT:0072A	Immunization administration by intramuscular injection of severe acute respiratory syndrome coronavirus 2 (SARS-CoV-2) (coronavirus disease [COVID-19]) vaccine, mRNA-LNP, spike protein, preservative free, 10 mcg/0.2 mL dosage, diluent reconstituted, tris-sucrose formulation; second dose
or	medication	NLM:RXNORM:2610319	SARS-CoV-2 (COVID-19) vaccine, mRNA-BNT162b2 0.05 MG/ML / SARS-CoV-2 (COVID-19) vaccine, mRNA-BNT162b2 OMICRON (BA.4/BA.5) 0.05 MG/ML Injectable Suspension

or	procedure	UMLS:CPT:91313	Severe acute respiratory syndrome coronavirus 2 (SARS-CoV-2) (coronavirus disease [COVID-19]) vaccine, mRNA-LNP, spike protein, bivalent, preservative free, 50 mcg/0.5 mL dosage, for intramuscular use
or	procedure	UMLS:CPT:0134A	Immunization administration by intramuscular injection of severe acute respiratory syndrome coronavirus 2 (SARS-CoV-2) (coronavirus disease [COVID-19]) vaccine, mRNA-LNP, spike protein, bivalent, preservative free, 50 mcg/0.5 mL dosage, booster dose
or	procedure	UMLS:CPT:1037175	Immunization administration by intramuscular injection of severe acute respiratory syndrome coronavirus 2 (SARS-CoV-2) (coronavirus disease [COVID-19]) vaccine, DNA, spike protein, adenovirus type 26 (Ad26) vector, preservative free, 5x1010 viral particles/0.5 mL dosage
or	medication	NLM:RXNORM:2610347	0.3 ML SARS-CoV-2 (COVID-19) vaccine, mRNA-BNT162b2 0.05 MG/ML / SARS-CoV-2 (COVID-19) vaccine, mRNA-BNT162b2 OMICRON (BA.4/BA.5) -1 MG/ML Injection
or	procedure	UMLS:CPT:1037228	Immunization administration by intramuscular injection of severe acute respiratory syndrome coronavirus 2 (SARS-CoV-2) (coronavirus disease [COVID-19]) vaccine, mRNA-

			LNP, spike protein, preservative free, 3 mcg/0.2 mL dosage, diluent reconstituted, tris-sucrose formulation
or	procedure	UMLS:CPT:0013A	Immunization administration by intramuscular injection of severe acute respiratory syndrome coronavirus 2 (SARS-CoV-2) (coronavirus disease [COVID-19]) vaccine, mRNA-LNP, spike protein, preservative free, 100 mcg/0.5 mL dosage; third dose
or	procedure	UMLS:CPT:0081A	Immunization administration by intramuscular injection of severe acute respiratory syndrome coronavirus 2 (SARS-CoV-2) (coronavirus disease [COVID-19]) vaccine, mRNA-LNP, spike protein, preservative free, 3 mcg/0.2 mL dosage, diluent reconstituted, tris-sucrose formulation; first dose
or	procedure	UMLS:CPT:0082A	Immunization administration by intramuscular injection of severe acute respiratory syndrome coronavirus 2 (SARS-CoV-2) (coronavirus disease [COVID-19]) vaccine, mRNA-LNP, spike protein, preservative free, 3 mcg/0.2 mL dosage, diluent reconstituted, tris-sucrose formulation; second dose
or	medication	NLM:RXNORM:2610328	SARS-CoV-2 (COVID-19) vaccine, mRNA-1273 0.05 MG/ML / SARS-CoV-2 (COVID-19) vaccine, mRNA-1273

			OMICRON (BA.4/BA.5) 0.05 MG/ML Injectable Suspension
or	procedure	UMLS:CPT:0154A	Immunization administration by intramuscular injection of severe acute respiratory syndrome coronavirus 2 (SARS-CoV-2) (coronavirus disease [COVID-19]) vaccine, mRNA-LNP, bivalent spike protein, preservative free, 10 mcg/0.2 mL dosage, diluent reconstituted, tris-sucrose formulation, booster dose
or	procedure	UMLS:CPT:0053A	Immunization administration by intramuscular injection of severe acute respiratory syndrome coronavirus 2 (SARS-CoV-2) (coronavirus disease [COVID-19]) vaccine, mRNA-LNP, spike protein, preservative free, 30 mcg/0.3 mL dosage, tris-sucrose formulation; third dose
or	procedure	UMLS:CPT:1037332	Immunization administration by intramuscular injection of severe acute respiratory syndrome coronavirus 2 (SARS-CoV-2) (coronavirus disease [COVID-19]) vaccine, mRNA-LNP, spike protein, preservative free, 25 mcg/0.25 mL dosage
or	procedure	UMLS:CPT:0052A	Immunization administration by intramuscular injection of severe acute respiratory syndrome coronavirus 2 (SARS-CoV-2) (coronavirus disease [COVID-19]) vaccine, mRNA-LNP, spike protein, preservative

			free, 30 mcg/0.3 mL dosage, tris-sucrose formulation; second dose
or	procedure	UMLS:CPT:0111A	Immunization administration by intramuscular injection of severe acute respiratory syndrome coronavirus 2 (SARS-CoV-2) (coronavirus disease [COVID-19]) vaccine, mRNA-LNP, spike protein, preservative free, 25 mcg/0.25 mL dosage; first dose
or	procedure	UMLS:CPT:0051A	Immunization administration by intramuscular injection of severe acute respiratory syndrome coronavirus 2 (SARS-CoV-2) (coronavirus disease [COVID-19]) vaccine, mRNA-LNP, spike protein, preservative free, 30 mcg/0.3 mL dosage, tris-sucrose formulation; first dose
or	procedure	UMLS:CPT:91311	Severe acute respiratory syndrome coronavirus 2 (SARS-CoV-2) (coronavirus disease [COVID-19]) vaccine, mRNA-LNP, spike protein, preservative free, 25 mcg/0.25 mL dosage, for intramuscular use
or	procedure	UMLS:CPT:0074A	Immunization administration by intramuscular injection of severe acute respiratory syndrome coronavirus 2 (SARS-CoV-2) (coronavirus disease [COVID-19]) vaccine, mRNA-LNP, spike protein, preservative free, 10 mcg/0.2 mL dosage, diluent reconstituted, tris-

			sucrose formulation; booster dose
or	procedure	UMLS:CPT:0112A	Immunization administration by intramuscular injection of severe acute respiratory syndrome coronavirus 2 (SARS-CoV-2) (coronavirus disease [COVID-19]) vaccine, mRNA-LNP, spike protein, preservative free, 25 mcg/0.25 mL dosage; second dose
or	procedure	UMLS:CPT:0083A	Immunization administration by intramuscular injection of severe acute respiratory syndrome coronavirus 2 (SARS-CoV-2) (coronavirus disease [COVID-19]) vaccine, mRNA-LNP, spike protein, preservative free, 3 mcg/0.2 mL dosage, diluent reconstituted, tris-sucrose formulation; third dose
or	procedure	UMLS:CPT:0073A	Immunization administration by intramuscular injection of severe acute respiratory syndrome coronavirus 2 (SARS-CoV-2) (coronavirus disease [COVID-19]) vaccine, mRNA-LNP, spike protein, preservative free, 10 mcg/0.2 mL dosage, diluent reconstituted, tris-sucrose formulation; third dose
or	procedure	UMLS:CPT:0173A	Immunization administration by intramuscular injection of severe acute respiratory syndrome coronavirus 2 (SARS-CoV-2) (coronavirus disease [COVID-19]) vaccine, mRNA-LNP, bivalent spike protein,

			preservative free, 3 mcg/0.2 mL dosage, diluent reconstituted, tris-sucrose formulation, third dose
or	procedure	UMLS:CPT:0164A	Immunization administration by intramuscular injection of severe acute respiratory syndrome coronavirus 2 (SARS-CoV-2) (coronavirus disease [COVID-19]) vaccine, mRNA-LNP, spike protein, bivalent, preservative free, 10 mcg/0.2 mL dosage, booster dose
or	procedure	UMLS:CPT:1037838	Immunization administration by intramuscular injection of severe acute respiratory syndrome coronavirus 2 (SARS-CoV-2) (coronavirus disease [COVID-19]) vaccine, mRNA-LNP, spike protein, preservative free, 50 mcg/0.5 mL dosage
or	procedure	UMLS:CPT:0094A	Immunization administration by intramuscular injection of severe acute respiratory syndrome coronavirus 2 (SARS-CoV-2) (coronavirus disease [COVID-19]) vaccine, mRNA-LNP, spike protein, preservative free, 50 mcg/0.5 mL dosage; booster dose, when administered to individuals 18 years and over
or	procedure	UMLS:CPT:0034A	Immunization administration by intramuscular injection of severe acute respiratory syndrome coronavirus 2 (SARS-CoV-2) (coronavirus disease [COVID-19]) vaccine, DNA, spike

			protein, adenovirus type 26 (Ad26) vector, preservative free, 5x1010 viral particles/0.5 mL dosage; booster dose
or	procedure	UMLS:CPT:0144A	Immunization administration by intramuscular injection of severe acute respiratory syndrome coronavirus 2 (SARS-CoV-2) (coronavirus disease [COVID-19]) vaccine, mRNA-LNP, spike protein, bivalent, preservative free, 25 mcg/0.25 mL dosage, booster dose
or	procedure	UMLS:CPT:0091A	Immunization administration by intramuscular injection of severe acute respiratory syndrome coronavirus 2 (SARS-CoV-2) (coronavirus disease [COVID-19]) vaccine, mRNA-LNP, spike protein, preservative free, 50 mcg/0.5 mL dosage; first dose, when administered to individuals 6 through 11 years
or	procedure	UMLS:CPT:0174A	Immunization administration by intramuscular injection of severe acute respiratory syndrome coronavirus 2 (SARS-CoV-2) (coronavirus disease [COVID-19]) vaccine, mRNA-LNP, bivalent spike protein, preservative free, 3 mcg/0.2 mL dosage, diluent reconstituted, tris-sucrose formulation, booster
or	procedure	UMLS:CPT:0092A	Immunization administration by intramuscular injection of severe acute respiratory syndrome coronavirus 2 (SARS-

			CoV-2) (coronavirus disease [COVID-19]) vaccine, mRNA-LNP, spike protein, preservative free, 50 mcg/0.5 mL dosage; second dose, when administered to individuals 6 through 11 years
or	procedure	UMLS:CPT:1036682	Immunization administration by intramuscular injection of severe acute respiratory syndrome coronavirus 2 (SARS-CoV-2) (coronavirus disease [COVID-19]) vaccine, recombinant spike protein nanoparticle, saponin-based adjuvant, preservative free, 5 mcg/0.5 mL dosage
or	procedure	UMLS:CPT:0041A	Immunization administration by intramuscular injection of severe acute respiratory syndrome coronavirus 2 (SARS-CoV-2) (coronavirus disease [COVID-19]) vaccine, recombinant spike protein nanoparticle, saponin-based adjuvant, preservative free, 5 mcg/0.5 mL dosage; first dose
or	procedure	UMLS:CPT:0113A	Immunization administration by intramuscular injection of severe acute respiratory syndrome coronavirus 2 (SARS-CoV-2) (coronavirus disease [COVID-19]) vaccine, mRNA-LNP, spike protein, preservative free, 25 mcg/0.25 mL dosage; third dose
or	procedure	UMLS:CPT:0042A	Immunization administration by intramuscular injection of

			severe acute respiratory syndrome coronavirus 2 (SARS-CoV-2) (coronavirus disease [COVID-19]) vaccine, recombinant spike protein nanoparticle, saponin-based adjuvant, preservative free, 5 mcg/0.5 mL dosage; second dose
or	procedure	UMLS:CPT:0093A	Immunization administration by intramuscular injection of severe acute respiratory syndrome coronavirus 2 (SARS-CoV-2) (coronavirus disease [COVID-19]) vaccine, mRNA-LNP, spike protein, preservative free, 50 mcg/0.5 mL dosage; third dose, when administered to individuals 6 through 11 years
or	procedure	UMLS:CPT:1036666	Immunization administration by intramuscular injection of severe acute respiratory syndrome coronavirus 2 (SARS-CoV-2) (coronavirus disease [COVID-19]) vaccine, DNA, spike protein, chimpanzee adenovirus Oxford 1 (ChAdOx1) vector, preservative free, 5x1010 viral particles/0.5 mL dosage
or	procedure	UMLS:CPT:0044A	Immunization administration by intramuscular injection of severe acute respiratory syndrome coronavirus 2 (SARS-CoV-2) (coronavirus disease [COVID-19]) vaccine, recombinant spike protein nanoparticle, saponin-based

				adjuvant, preservative free, 5 mcg/0.5mL dosage; booster
date constraint		The terms in this group occurred between Dec 1, 2019 and Dec 31, 2023		
event relationship		Any instance of Molnupiravir occurred within 7 days on or after any instance of COVID-19, no vaccination		
**Group 1B Molnupiravir**				
must have		medication	NLM:RXNORM:2284718	remdesivir
**Group 2**				
**Group 2A COVID-19**				
must have	any of	diagnosis	UMLS:ICD10CM:U07.1	COVID-19
		diagnosis	UMLS:ICD10CM:U07.2	COVID-19, virus not identified (WHO)
		diagnosis	UMLS:ICD10CM:J12.82	Pneumonia due to coronavirus disease 2019
		laboratory	UMLS:LNC:94500-6	SARS-CoV-2 (COVID-19) RNA [Presence] in Respiratory specimen by NAA with probe detection (labResult: Positive)
		laboratory	UMLS:LNC:94309-2	SARS-CoV-2 (COVID-19) RNA [Presence] in Specimen by NAA with probe detection (labResult: Positive)
		laboratory	UMLS:LNC:94565-9	SARS-CoV-2 (COVID-19) RNA [Presence] in Nasopharynx by NAA with non-probe detection (labResult: Positive)
		laboratory	UMLS:LNC:94759-8	SARS-CoV-2 (COVID-19) RNA [Presence] in Nasopharynx by NAA with probe detection (labResult: Positive)
		laboratory	UMLS:LNC:95608-6	SARS-CoV-2 (COVID-19) RNA [Presence] in Respiratory specimen by NAA with non-probe detection (labResult: Positive)

		laboratory	UMLS:LNC:94845-5	SARS-CoV-2 (COVID-19) RNA [Presence] in Saliva (oral fluid) by NAA with probe detection (labResult: Positive)
		laboratory	UMLS:LNC:95406-5	SARS-CoV-2 (COVID-19) RNA [Presence] in Nose by NAA with probe detection (labResult: Positive)
	and	visit	TNX:Visit	Visit (Data Source: TriNetX)
date constraint		The terms in this group occurred at any time		
event relationship		Any instance of thyroid cancer history occurred at least 1 day before the first instance of COVID-19		
**Group 2B thyroid cancer history**				
cannot have		diagnosis	UMLS:ICD10CM:C73	Malignant neoplasm of thyroid gland
	or	diagnosis	UMLS:ICD10CM:Z85.850	Personal history of malignant neoplasm of thyroid
