# Supplementary material for: Association Between COVID-19 Infection and Thyroid Cancer Development: A Retrospective Cohort Study Using the TriNetX Database
Source: Biomedicines. 2025 Aug 8;13(8):1933. doi: 10.3390/biomedicines13081933 (PMC12383963; doi:10.3390/biomedicines13081933)
Supplement: Supplementary file 1 [file biomedicines-13-01933-s001.zip › Supplementary File S3.pdf]

Supplementary File S3: Detailed query criteria of subgroup of population.

Query Criteria for post-COVID, >41

This query was run on the network Global Collaborative Network with 151 HCO(s) queried and 151 HCO(s) responded. A total of 125 provider(s) responded with patients. The final cohort included 1,457,295 patients who matched the query criteria listed in the table below.

**Ungrouped terms**				
must have		demographics	Age	Age (at least 41 years (most recent occurrence))
	and any of	demographics	UMLS:HL7V3.0:Gender:M	Male
		demographics	UMLS:HL7V3.0:Gender:F	Female
**Group 1**				
**COVID-19, no vaccination**				
must have	any of	diagnosis	UMLS:ICD10CM:U07.1	COVID-19
		diagnosis	UMLS:ICD10CM:U07.2	COVID-19, virus not identified (WHO)
		diagnosis	UMLS:ICD10CM:J12.82	Pneumonia due to coronavirus disease 2019
		laboratory	UMLS:LNC:94500-6	SARS-CoV-2 (COVID-19) RNA [Presence] in Respiratory specimen by NAA with probe detection (labResult: Positive)

	laboratory	UMLS:LNC:94309-2	SARS-CoV-2 (COVID-19) RNA [Presence] in Specimen by NAA with probe detection (labResult: Positive)
	laboratory	UMLS:LNC:94565-9	SARS-CoV-2 (COVID-19) RNA [Presence] in Nasopharynx by NAA with non-probe detection (labResult: Positive)
	laboratory	UMLS:LNC:94759-8	SARS-CoV-2 (COVID-19) RNA [Presence] in Nasopharynx by NAA with probe detection (labResult: Positive)
	laboratory	UMLS:LNC:95608-6	SARS-CoV-2 (COVID-19) RNA [Presence] in Respiratory specimen by NAA with non-probe detection (labResult: Positive)
	laboratory	UMLS:LNC:94845-5	SARS-CoV-2 (COVID-19) RNA [Presence] in Saliva (oral fluid) by NAA with probe detection (labResult: Positive)
	laboratory	UMLS:LNC:95406-5	SARS-CoV-2 (COVID-19) RNA [Presence] in Nose by NAA with probe detection (labResult: Positive)
cannot	medicatio	NLM:CVX:208	COVID-19, mRNA, LNP-S,

have		n		PF, 30 mcg/0.3 mL dose
	or	medicatio n	NLM:CVX:207	COVID-19, mRNA, LNP-S, PF, 100 mcg/0.5mL dose or 50 mcg/0.25mL dose
	or	medicatio n	NLM:CVX:212	COVID-19 vaccine, vector-nr, rS-Ad26, PF, 0.5 mL
	or	medicatio n	NLM:RXNORM:OM OP5042939	COVID-19 vaccine
	or	medicatio n	NLM:CVX:300	COVID-19, mRNA, LNP-S, bivalent, PF, 30 mcg/0.3 mL dose
	or	medicatio n	NLM:CVX:217	COVID-19, mRNA, LNP-S, PF, 30 mcg/0.3 mL dose, tris-sucrose
	or	medicatio n	NLM:CVX:229	COVID-19, mRNA, LNP-S, bivalent, PF, 50 mcg/0.5 mL or 25mcg/0.25 mL dose
	or	medicatio n	NLM:CVX:218	COVID-19, mRNA, LNP-S, PF, 10 mcg/0.2 mL dose, tris-sucrose
	or	medicatio n	NLM:CVX:520	COVID-19 mRNA, bivalent, original/Omicron BA.1, Non-US Vaccine Product, Pfizer-BioNTech
	or	medicatio n	NLM:CVX:519	COVID-19 mRNA, bivalent, original/Omicron BA.1, Non-US Vaccine (Spikevax Bivalent), Moderna

or	medication	NLM:CVX:301	COVID-19, mRNA, LNP-S, bivalent, PF, 10 mcg/0.2 mL dose
or	medication	NLM:CVX:219	COVID-19, mRNA, LNP-S, PF, 3 mcg/0.2 mL dose, tris-sucrose
or	medication	NLM:CVX:228	COVID-19, mRNA, LNP-S, PF, pediatric 25 mcg/0.25 mL dose
or	medication	NLM:CVX:230	COVID-19, mRNA, LNP-S, bivalent booster, PF, 10 mcg/0.2 mL
or	medication	NLM:CVX:221	COVID-19, mRNA, LNP-S, PF, 50 mcg/0.5 mL dose
or	medication	NLM:CVX:210	COVID-19 vaccine, vector-nr, rS-ChAdOx1, PF, 0.5 mL
or	medication	NLM:CVX:302	COVID-19, mRNA, LNP-S, bivalent, PF, 3 mcg/0.2 mL dose
or	medication	NLM:CVX:511	COVID-19 IV Non-US Vaccine (CoronaVac, Sinovac)
or	medication	NLM:RXNORM:2468231	SARS-CoV-2 (COVID-19) vaccine, mRNA spike protein
or	procedure	UMLS:CPT:91300	Severe acute respiratory syndrome coronavirus 2 (SARS-CoV-2) (coronavirus disease [COVID-19])

			vaccine, mRNA-LNP, spike protein, preservative free, 30 mcg/0.3 mL dosage, diluent reconstituted, for intramuscular use
or	procedure	UMLS:CPT:0001A	Immunization administration by intramuscular injection of severe acute respiratory syndrome coronavirus 2 (SARS-CoV-2) (coronavirus disease [COVID-19]) vaccine, mRNA-LNP, spike protein, preservative free, 30 mcg/0.3 mL dosage, diluent reconstituted; first dose
or	procedure	UMLS:CPT:0002A	Immunization administration by intramuscular injection of severe acute respiratory syndrome coronavirus 2 (SARS-CoV-2) (coronavirus disease [COVID-19]) vaccine, mRNA-LNP, spike protein, preservative free, 30 mcg/0.3 mL dosage, diluent reconstituted; second dose
or	procedure	UMLS:CPT:91301	Severe acute respiratory syndrome coronavirus 2 (SARS-CoV-2) (coronavirus disease [COVID-19]) vaccine, mRNA-LNP, spike

			protein, preservative free, 100 mcg/0.5 mL dosage, for intramuscular use
or	procedure	UMLS:CPT:0011A	Immunization administration by intramuscular injection of severe acute respiratory syndrome coronavirus 2 (SARS-CoV-2) (coronavirus disease [COVID-19]) vaccine, mRNA-LNP, spike protein, preservative free, 100 mcg/0.5 mL dosage; first dose
or	procedure	UMLS:CPT:0012A	Immunization administration by intramuscular injection of severe acute respiratory syndrome coronavirus 2 (SARS-CoV-2) (coronavirus disease [COVID-19]) vaccine, mRNA-LNP, spike protein, preservative free, 100 mcg/0.5 mL dosage; second dose
or	procedure	UMLS:SNOMED:840534001	Administration of SARS-CoV-2 antigen vaccine
or	medication	NLM:CVX:213	SARS-CoV-2 (COVID-19) Vaccine
or	procedure	UMLS:CPT:1036660	Immunization administration by

			intramuscular injection of severe acute respiratory syndrome coronavirus 2 (SARS-CoV-2) (coronavirus disease [COVID-19]) vaccine, mRNA-LNP, spike protein, preservative free, 30 mcg/0.3 mL dosage, diluent reconstituted
or	procedure	UMLS:CPT:1036663	Immunization administration by intramuscular injection of severe acute respiratory syndrome coronavirus 2 (SARS-CoV-2) (coronavirus disease [COVID-19]) vaccine, mRNA-LNP, spike protein, preservative free, 100 mcg/0.5 mL dosage
or	procedure	UMLS:CPT:0124A	Immunization administration by intramuscular injection of severe acute respiratory syndrome coronavirus 2 (SARS-CoV-2) (coronavirus disease [COVID-19]) vaccine, mRNA-LNP, bivalent spike protein, preservative free, 30 mcg/0.3 mL dosage, tris-sucrose formulation, booster dose

or	procedure	UMLS:CPT:0004A	Immunization administration by intramuscular injection of severe acute respiratory syndrome coronavirus 2 (SARS-CoV-2) (coronavirus disease [COVID-19]) vaccine, mRNA-LNP, spike protein, preservative free, 30 mcg/0.3 mL dosage, diluent reconstituted; booster dose
or	procedure	UMLS:CPT:0003A	Immunization administration by intramuscular injection of severe acute respiratory syndrome coronavirus 2 (SARS-CoV-2) (coronavirus disease [COVID-19]) vaccine, mRNA-LNP, spike protein, preservative free, 30 mcg/0.3 mL dosage, diluent reconstituted; third dose
or	procedure	UMLS:CPT:1037166	Immunization administration by intramuscular injection of severe acute respiratory syndrome coronavirus 2 (SARS-CoV-2) (coronavirus disease [COVID-19]) vaccine, mRNA-LNP, spike protein, preservative free, 30 mcg/0.3 mL dosage, tris-

			sucrose formulation
or	procedure	UMLS:CPT:0054A	Immunization administration by intramuscular injection of severe acute respiratory syndrome coronavirus 2 (SARS-CoV-2) (coronavirus disease [COVID-19]) vaccine, mRNA-LNP, spike protein, preservative free, 30 mcg/0.3 mL dosage, tris-sucrose formulation; booster dose
or	procedure	UMLS:CPT:0064A	Immunization administration by intramuscular injection of severe acute respiratory syndrome coronavirus 2 (SARS-CoV-2) (coronavirus disease [COVID-19]) vaccine, mRNA-LNP, spike protein, preservative free, 50 mcg/0.25 mL dosage, booster dose
or	procedure	UMLS:CPT:90480	Immunization administration by intramuscular injection of severe acute respiratory syndrome coronavirus 2 (SARS-CoV-2) (coronavirus disease [COVID-19])

			vaccine, single dose
or	procedure	UMLS:CPT:1037171	Immunization administration by intramuscular injection of severe acute respiratory syndrome coronavirus 2 (SARS-CoV-2) (coronavirus disease [COVID-19]) vaccine, mRNA-LNP, spike protein, preservative free, 10 mcg/0.2 mL dosage, diluent reconstituted, tris-sucrose formulation
or	procedure	UMLS:CPT:0071A	Immunization administration by intramuscular injection of severe acute respiratory syndrome coronavirus 2 (SARS-CoV-2) (coronavirus disease [COVID-19]) vaccine, mRNA-LNP, spike protein, preservative free, 10 mcg/0.2 mL dosage, diluent reconstituted, tris-sucrose formulation; first dose
or	procedure	UMLS:CPT:0072A	Immunization administration by intramuscular injection of severe acute respiratory syndrome coronavirus 2 (SARS-CoV-2) (coronavirus disease [COVID-19])

			vaccine, mRNA-LNP, spike protein, preservative free, 10 mcg/0.2 mL dosage, diluent reconstituted, tris-sucrose formulation; second dose
or	medication	NLM:RXNORM:2610319	SARS-CoV-2 (COVID-19) vaccine, mRNA-BNT162b2 0.05 MG/ML / SARS-CoV-2 (COVID-19) vaccine, mRNA-BNT162b2 OMICRON (BA.4/BA.5) 0.05 MG/ML Injectable Suspension
or	procedure	UMLS:CPT:91313	Severe acute respiratory syndrome coronavirus 2 (SARS-CoV-2) (coronavirus disease [COVID-19]) vaccine, mRNA-LNP, spike protein, bivalent, preservative free, 50 mcg/0.5 mL dosage, for intramuscular use
or	procedure	UMLS:CPT:0134A	Immunization administration by intramuscular injection of severe acute respiratory syndrome coronavirus 2 (SARS-CoV-2) (coronavirus disease [COVID-19]) vaccine, mRNA-LNP, spike protein, bivalent, preservative free, 50 mcg/0.5 mL dosage, booster

			dose
or	procedure	UMLS:CPT:1037175	Immunization administration by intramuscular injection of severe acute respiratory syndrome coronavirus 2 (SARS-CoV-2) (coronavirus disease [COVID-19]) vaccine, DNA, spike protein, adenovirus type 26 (Ad26) vector, preservative free, 5x1010 viral particles/0.5 mL dosage
or	medication	NLM:RXNORM:2610347	0.3 ML SARS-CoV-2 (COVID-19) vaccine, mRNA-BNT162b2 0.05 MG/ML / SARS-CoV-2 (COVID-19) vaccine, mRNA-BNT162b2 OMICRON (BA.4/BA.5) -1 MG/ML Injection
or	procedure	UMLS:CPT:1037228	Immunization administration by intramuscular injection of severe acute respiratory syndrome coronavirus 2 (SARS-CoV-2) (coronavirus disease [COVID-19]) vaccine, mRNA-LNP, spike protein, preservative free, 3 mcg/0.2 mL dosage, diluent reconstituted, tris-sucrose

			formulation
or	procedure	UMLS:CPT:0013A	Immunization administration by intramuscular injection of severe acute respiratory syndrome coronavirus 2 (SARS-CoV-2) (coronavirus disease [COVID-19]) vaccine, mRNA-LNP, spike protein, preservative free, 100 mcg/0.5 mL dosage; third dose
or	procedure	UMLS:CPT:0081A	Immunization administration by intramuscular injection of severe acute respiratory syndrome coronavirus 2 (SARS-CoV-2) (coronavirus disease [COVID-19]) vaccine, mRNA-LNP, spike protein, preservative free, 3 mcg/0.2 mL dosage, diluent reconstituted, tris-sucrose formulation; first dose
or	procedure	UMLS:CPT:0082A	Immunization administration by intramuscular injection of severe acute respiratory syndrome coronavirus 2 (SARS-CoV-2) (coronavirus disease [COVID-19]) vaccine, mRNA-LNP, spike

			protein, preservative free, 3 mcg/0.2 mL dosage, diluent reconstituted, tris-sucrose formulation; second dose
or	medicatio n	NLM:RXNORM:261 0328	SARS-CoV-2 (COVID-19) vaccine, mRNA-1273 0.05 MG/ML / SARS-CoV-2 (COVID-19) vaccine, mRNA-1273 OMICRON (BA.4/BA.5) 0.05 MG/ML Injectable Suspension
or	procedure	UMLS:CPT:0154A	Immunization administration by intramuscular injection of severe acute respiratory syndrome coronavirus 2 (SARS-CoV-2) (coronavirus disease [COVID-19]) vaccine, mRNA-LNP, bivalent spike protein, preservative free, 10 mcg/0.2 mL dosage, diluent reconstituted, tris-sucrose formulation, booster dose
or	procedure	UMLS:CPT:0053A	Immunization administration by intramuscular injection of severe acute respiratory syndrome coronavirus 2 (SARS-CoV-2) (coronavirus disease [COVID-19]) vaccine, mRNA-LNP, spike

			protein, preservative free, 30 mcg/0.3 mL dosage, tris-sucrose formulation; third dose
or	procedure	UMLS:CPT:103733 2	Immunization administration by intramuscular injection of severe acute respiratory syndrome coronavirus 2 (SARS-CoV-2) (coronavirus disease [COVID-19]) vaccine, mRNA-LNP, spike protein, preservative free, 25 mcg/0.25 mL dosage
or	procedure	UMLS:CPT:0052A	Immunization administration by intramuscular injection of severe acute respiratory syndrome coronavirus 2 (SARS-CoV-2) (coronavirus disease [COVID-19]) vaccine, mRNA-LNP, spike protein, preservative free, 30 mcg/0.3 mL dosage, tris-sucrose formulation; second dose
or	procedure	UMLS:CPT:0111A	Immunization administration by intramuscular injection of severe acute respiratory syndrome coronavirus 2 (SARS-CoV-2) (coronavirus

			disease [COVID-19]) vaccine, mRNA-LNP, spike protein, preservative free, 25 mcg/0.25 mL dosage; first dose
or	procedure	UMLS:CPT:0051A	Immunization administration by intramuscular injection of severe acute respiratory syndrome coronavirus 2 (SARS-CoV-2) (coronavirus disease [COVID-19]) vaccine, mRNA-LNP, spike protein, preservative free, 30 mcg/0.3 mL dosage, tris-sucrose formulation; first dose
or	procedure	UMLS:CPT:91311	Severe acute respiratory syndrome coronavirus 2 (SARS-CoV-2) (coronavirus disease [COVID-19]) vaccine, mRNA-LNP, spike protein, preservative free, 25 mcg/0.25 mL dosage, for intramuscular use
or	procedure	UMLS:CPT:0074A	Immunization administration by intramuscular injection of severe acute respiratory syndrome coronavirus 2 (SARS-CoV-2) (coronavirus disease [COVID-19])

			vaccine, mRNA-LNP, spike protein, preservative free, 10 mcg/0.2 mL dosage, diluent reconstituted, tris-sucrose formulation; booster dose
or	procedure	UMLS:CPT:0112A	Immunization administration by intramuscular injection of severe acute respiratory syndrome coronavirus 2 (SARS-CoV-2) (coronavirus disease [COVID-19]) vaccine, mRNA-LNP, spike protein, preservative free, 25 mcg/0.25 mL dosage; second dose
or	procedure	UMLS:CPT:0083A	Immunization administration by intramuscular injection of severe acute respiratory syndrome coronavirus 2 (SARS-CoV-2) (coronavirus disease [COVID-19]) vaccine, mRNA-LNP, spike protein, preservative free, 3 mcg/0.2 mL dosage, diluent reconstituted, tris-sucrose formulation; third dose
or	procedure	UMLS:CPT:0073A	Immunization administration by intramuscular injection of severe acute respiratory

			syndrome coronavirus 2 (SARS-CoV-2) (coronavirus disease [COVID-19]) vaccine, mRNA-LNP, spike protein, preservative free, 10 mcg/0.2 mL dosage, diluent reconstituted, tris-sucrose formulation; third dose
or	procedure	UMLS:CPT:0173A	Immunization administration by intramuscular injection of severe acute respiratory syndrome coronavirus 2 (SARS-CoV-2) (coronavirus disease [COVID-19]) vaccine, mRNA-LNP, bivalent spike protein, preservative free, 3 mcg/0.2 mL dosage, diluent reconstituted, tris-sucrose formulation, third dose
or	procedure	UMLS:CPT:0164A	Immunization administration by intramuscular injection of severe acute respiratory syndrome coronavirus 2 (SARS-CoV-2) (coronavirus disease [COVID-19]) vaccine, mRNA-LNP, spike protein, bivalent, preservative free, 10 mcg/0.2 mL dosage, booster

			dose
or	procedure	UMLS:CPT:1037838	Immunization administration by intramuscular injection of severe acute respiratory syndrome coronavirus 2 (SARS-CoV-2) (coronavirus disease [COVID-19]) vaccine, mRNA-LNP, spike protein, preservative free, 50 mcg/0.5 mL dosage
or	procedure	UMLS:CPT:0094A	Immunization administration by intramuscular injection of severe acute respiratory syndrome coronavirus 2 (SARS-CoV-2) (coronavirus disease [COVID-19]) vaccine, mRNA-LNP, spike protein, preservative free, 50 mcg/0.5 mL dosage; booster dose, when administered to individuals 18 years and over
or	procedure	UMLS:CPT:0034A	Immunization administration by intramuscular injection of severe acute respiratory syndrome coronavirus 2 (SARS-CoV-2) (coronavirus disease [COVID-19]) vaccine, DNA, spike protein,

			adenovirus type 26 (Ad26) vector, preservative free, 5x1010 viral particles/0.5 mL dosage; booster dose
or	procedure	UMLS:CPT:0144A	Immunization administration by intramuscular injection of severe acute respiratory syndrome coronavirus 2 (SARS-CoV-2) (coronavirus disease [COVID-19]) vaccine, mRNA-LNP, spike protein, bivalent, preservative free, 25 mcg/0.25 mL dosage, booster dose
or	procedure	UMLS:CPT:0091A	Immunization administration by intramuscular injection of severe acute respiratory syndrome coronavirus 2 (SARS-CoV-2) (coronavirus disease [COVID-19]) vaccine, mRNA-LNP, spike protein, preservative free, 50 mcg/0.5 mL dosage; first dose, when administered to individuals 6 through 11 years
or	procedure	UMLS:CPT:0174A	Immunization administration by intramuscular injection of

			severe acute respiratory syndrome coronavirus 2 (SARS-CoV-2) (coronavirus disease [COVID-19]) vaccine, mRNA-LNP, bivalent spike protein, preservative free, 3 mcg/0.2 mL dosage, diluent reconstituted, tris-sucrose formulation, booster
or	procedure	UMLS:CPT:0092A	Immunization administration by intramuscular injection of severe acute respiratory syndrome coronavirus 2 (SARS-CoV-2) (coronavirus disease [COVID-19]) vaccine, mRNA-LNP, spike protein, preservative free, 50 mcg/0.5 mL dosage; second dose, when administered to individuals 6 through 11 years
or	procedure	UMLS:CPT:1036682	Immunization administration by intramuscular injection of severe acute respiratory syndrome coronavirus 2 (SARS-CoV-2) (coronavirus disease [COVID-19]) vaccine, recombinant spike protein nanoparticle,

			saponin-based adjuvant, preservative free, 5 mcg/0.5 mL dosage
or	procedure	UMLS:CPT:0041A	Immunization administration by intramuscular injection of severe acute respiratory syndrome coronavirus 2 (SARS-CoV-2) (coronavirus disease [COVID-19]) vaccine, recombinant spike protein nanoparticle, saponin-based adjuvant, preservative free, 5 mcg/0.5 mL dosage; first dose
or	procedure	UMLS:CPT:0113A	Immunization administration by intramuscular injection of severe acute respiratory syndrome coronavirus 2 (SARS-CoV-2) (coronavirus disease [COVID-19]) vaccine, mRNA-LNP, spike protein, preservative free, 25 mcg/0.25 mL dosage; third dose
or	procedure	UMLS:CPT:0042A	Immunization administration by intramuscular injection of severe acute respiratory syndrome coronavirus 2 (SARS-CoV-2) (coronavirus

			disease [COVID-19]) vaccine, recombinant spike protein nanoparticle, saponin-based adjuvant, preservative free, 5 mcg/0.5 mL dosage; second dose
or	procedure	UMLS:CPT:0093A	Immunization administration by intramuscular injection of severe acute respiratory syndrome coronavirus 2 (SARS-CoV-2) (coronavirus disease [COVID-19]) vaccine, mRNA-LNP, spike protein, preservative free, 50 mcg/0.5 mL dosage; third dose, when administered to individuals 6 through 11 years
or	procedure	UMLS:CPT:1036666	Immunization administration by intramuscular injection of severe acute respiratory syndrome coronavirus 2 (SARS-CoV-2) (coronavirus disease [COVID-19]) vaccine, DNA, spike protein, chimpanzee adenovirus Oxford 1 (ChAdOx1) vector, preservative free, 5x1010 viral particles/0.5 mL dosage

	or	procedure	UMLS:CPT:0044A	Immunization administration by intramuscular injection of severe acute respiratory syndrome coronavirus 2 (SARS-CoV-2) (coronavirus disease [COVID-19]) vaccine, recombinant spike protein nanoparticle, saponin-based adjuvant, preservative free, 5 mcg/0.5mL dosage; booster
date constraint		The terms in this group occurred between Dec 1, 2019 and Dec 31, 2023		
**Group 2**				
**Group 2A COVID-19**				
must have	any of	diagnosis	UMLS:ICD10CM:U07.1	COVID-19
		diagnosis	UMLS:ICD10CM:U07.2	COVID-19, virus not identified (WHO)
		diagnosis	UMLS:ICD10CM:J12.82	Pneumonia due to coronavirus disease 2019
		laboratory	UMLS:LNC:94500-6	SARS-CoV-2 (COVID-19) RNA [Presence] in Respiratory specimen by NAA with probe detection (labResult: Positive)
		laboratory	UMLS:LNC:94309-2	SARS-CoV-2 (COVID-19) RNA [Presence] in Specimen by NAA with probe detection

				(labResult: Positive)
		laboratory	UMLS:LNC:94565-9	SARS-CoV-2 (COVID-19) RNA [Presence] in Nasopharynx by NAA with non-probe detection (labResult: Positive)
		laboratory	UMLS:LNC:94759-8	SARS-CoV-2 (COVID-19) RNA [Presence] in Nasopharynx by NAA with probe detection (labResult: Positive)
		laboratory	UMLS:LNC:95608-6	SARS-CoV-2 (COVID-19) RNA [Presence] in Respiratory specimen by NAA with non-probe detection (labResult: Positive)
		laboratory	UMLS:LNC:94845-5	SARS-CoV-2 (COVID-19) RNA [Presence] in Saliva (oral fluid) by NAA with probe detection (labResult: Positive)
		laboratory	UMLS:LNC:95406-5	SARS-CoV-2 (COVID-19) RNA [Presence] in Nose by NAA with probe detection (labResult: Positive)
	and	visit	TNX:Visit	Visit (Data Source: TriNetX)
date constraint	The terms in this group occurred at any time			
event relationship	Any instance of thyroid cancer history occurred at least 1 day			

		before the first instance of COVID-19		
**Group 2B thyroid cancer history**				
cannot have		diagnosis	UMLS:ICD10CM:C73	Malignant neoplasm of thyroid gland
	or	diagnosis	UMLS:ICD10CM:Z85.850	Personal history of malignant neoplasm of thyroid

Query Criteria for Cohort post-COVID, 18-40

This query was run on the network Global Collaborative Network with 151 HCO(s) queried and 151 HCO(s) responded. A total of 124 provider(s) responded with patients. The final cohort included 719,968 patients who matched the query criteria listed in the table below.

**Ungrouped terms**				
must have		demographics	Age	Age (between 18 and 40 years (most recent occurrence))
	and any of	demographics	UMLS:HL7V3.0:Gender:M	Male
		demographics	UMLS:HL7V3.0:Gender:F	Female
**Group 1**				
**COVID-19, no vaccination**				
must have	any of	diagnosis	UMLS:ICD10CM:U07.1	COVID-19
		diagnosis	UMLS:ICD10CM:U	COVID-19, virus not

	07.2	identified (WHO)
diagnosis	UMLS:ICD10CM:J12.82	Pneumonia due to coronavirus disease 2019
laboratory	UMLS:LNC:94500-6	SARS-CoV-2 (COVID-19) RNA [Presence] in Respiratory specimen by NAA with probe detection (labResult: Positive)
laboratory	UMLS:LNC:94309-2	SARS-CoV-2 (COVID-19) RNA [Presence] in Specimen by NAA with probe detection (labResult: Positive)
laboratory	UMLS:LNC:94565-9	SARS-CoV-2 (COVID-19) RNA [Presence] in Nasopharynx by NAA with non-probe detection (labResult: Positive)
laboratory	UMLS:LNC:94759-8	SARS-CoV-2 (COVID-19) RNA [Presence] in Nasopharynx by NAA with probe detection (labResult: Positive)
laboratory	UMLS:LNC:95608-6	SARS-CoV-2 (COVID-19) RNA [Presence] in Respiratory specimen by NAA with non-probe detection (labResult: Positive)
laboratory	UMLS:LNC:94845-5	SARS-CoV-2 (COVID-19) RNA [Presence] in Saliva

				(oral fluid) by NAA with probe detection (labResult: Positive)
		laboratory	UMLS:LNC:95406-5	SARS-CoV-2 (COVID-19) RNA [Presence] in Nose by NAA with probe detection (labResult: Positive)
cannot have		medication	NLM:CVX:208	COVID-19, mRNA, LNP-S, PF, 30 mcg/0.3 mL dose
	or	medication	NLM:CVX:207	COVID-19, mRNA, LNP-S, PF, 100 mcg/0.5mL dose or 50 mcg/0.25mL dose
	or	medication	NLM:CVX:212	COVID-19 vaccine, vector-nr, rS-Ad26, PF, 0.5 mL
	or	medication	NLM:RXNORM:OMOP5042939	COVID-19 vaccine
	or	medication	NLM:CVX:300	COVID-19, mRNA, LNP-S, bivalent, PF, 30 mcg/0.3 mL dose
	or	medication	NLM:CVX:217	COVID-19, mRNA, LNP-S, PF, 30 mcg/0.3 mL dose, tris-sucrose
	or	medication	NLM:CVX:229	COVID-19, mRNA, LNP-S, bivalent, PF, 50 mcg/0.5 mL or 25mcg/0.25 mL dose
	or	medication	NLM:CVX:218	COVID-19, mRNA, LNP-S, PF, 10 mcg/0.2 mL dose, tris-sucrose
	or	medicatio	NLM:CVX:520	COVID-19 mRNA, bivalent,

	n		original/Omicron BA.1, Non-US Vaccine Product, Pfizer-BioNTech
or	medication	NLM:CVX:519	COVID-19 mRNA, bivalent, original/Omicron BA.1, Non-US Vaccine (Spikevax Bivalent), Moderna
or	medication	NLM:CVX:301	COVID-19, mRNA, LNP-S, bivalent, PF, 10 mcg/0.2 mL dose
or	medication	NLM:CVX:219	COVID-19, mRNA, LNP-S, PF, 3 mcg/0.2 mL dose, tris-sucrose
or	medication	NLM:CVX:228	COVID-19, mRNA, LNP-S, PF, pediatric 25 mcg/0.25 mL dose
or	medication	NLM:CVX:230	COVID-19, mRNA, LNP-S, bivalent booster, PF, 10 mcg/0.2 mL
or	medication	NLM:CVX:221	COVID-19, mRNA, LNP-S, PF, 50 mcg/0.5 mL dose
or	medication	NLM:CVX:210	COVID-19 vaccine, vector-nr, rS-ChAdOx1, PF, 0.5 mL
or	medication	NLM:CVX:302	COVID-19, mRNA, LNP-S, bivalent, PF, 3 mcg/0.2 mL dose
or	medication	NLM:CVX:511	COVID-19 IV Non-US Vaccine (CoronaVac, Sinovac)

or	medicatio n	NLM:RXNORM:246 8231	SARS-CoV-2 (COVID-19) vaccine, mRNA spike protein
or	procedure	UMLS:CPT:91300	Severe acute respiratory syndrome coronavirus 2 (SARS-CoV-2) (coronavirus disease [COVID-19]) vaccine, mRNA-LNP, spike protein, preservative free, 30 mcg/0.3 mL dosage, diluent reconstituted, for intramuscular use
or	procedure	UMLS:CPT:0001A	Immunization administration by intramuscular injection of severe acute respiratory syndrome coronavirus 2 (SARS-CoV-2) (coronavirus disease [COVID-19]) vaccine, mRNA-LNP, spike protein, preservative free, 30 mcg/0.3 mL dosage, diluent reconstituted; first dose
or	procedure	UMLS:CPT:0002A	Immunization administration by intramuscular injection of severe acute respiratory syndrome coronavirus 2 (SARS-CoV-2) (coronavirus disease [COVID-19]) vaccine, mRNA-LNP, spike protein, preservative free, 30

			mcg/0.3 mL dosage, diluent reconstituted; second dose
or	procedure	UMLS:CPT:91301	Severe acute respiratory syndrome coronavirus 2 (SARS-CoV-2) (coronavirus disease [COVID-19]) vaccine, mRNA-LNP, spike protein, preservative free, 100 mcg/0.5 mL dosage, for intramuscular use
or	procedure	UMLS:CPT:0011A	Immunization administration by intramuscular injection of severe acute respiratory syndrome coronavirus 2 (SARS-CoV-2) (coronavirus disease [COVID-19]) vaccine, mRNA-LNP, spike protein, preservative free, 100 mcg/0.5 mL dosage; first dose
or	procedure	UMLS:CPT:0012A	Immunization administration by intramuscular injection of severe acute respiratory syndrome coronavirus 2 (SARS-CoV-2) (coronavirus disease [COVID-19]) vaccine, mRNA-LNP, spike protein, preservative free, 100 mcg/0.5 mL dosage;

			second dose
or	procedure	UMLS:SNOMED:840534001	Administration of SARS-CoV-2 antigen vaccine
or	medication	NLM:CVX:213	SARS-CoV-2 (COVID-19) Vaccine
or	procedure	UMLS:CPT:1036660	Immunization administration by intramuscular injection of severe acute respiratory syndrome coronavirus 2 (SARS-CoV-2) (coronavirus disease [COVID-19]) vaccine, mRNA-LNP, spike protein, preservative free, 30 mcg/0.3 mL dosage, diluent reconstituted
or	procedure	UMLS:CPT:1036663	Immunization administration by intramuscular injection of severe acute respiratory syndrome coronavirus 2 (SARS-CoV-2) (coronavirus disease [COVID-19]) vaccine, mRNA-LNP, spike protein, preservative free, 100 mcg/0.5 mL dosage
or	procedure	UMLS:CPT:0124A	Immunization administration by intramuscular injection of severe acute respiratory syndrome coronavirus 2

			(SARS-CoV-2) (coronavirus disease [COVID-19]) vaccine, mRNA-LNP, bivalent spike protein, preservative free, 30 mcg/0.3 mL dosage, tris-sucrose formulation, booster dose
or	procedure	UMLS:CPT:0004A	Immunization administration by intramuscular injection of severe acute respiratory syndrome coronavirus 2 (SARS-CoV-2) (coronavirus disease [COVID-19]) vaccine, mRNA-LNP, spike protein, preservative free, 30 mcg/0.3 mL dosage, diluent reconstituted; booster dose
or	procedure	UMLS:CPT:0003A	Immunization administration by intramuscular injection of severe acute respiratory syndrome coronavirus 2 (SARS-CoV-2) (coronavirus disease [COVID-19]) vaccine, mRNA-LNP, spike protein, preservative free, 30 mcg/0.3 mL dosage, diluent reconstituted; third dose
or	procedure	UMLS:CPT:103716	Immunization administration by

		6	intramuscular injection of severe acute respiratory syndrome coronavirus 2 (SARS-CoV-2) (coronavirus disease [COVID-19]) vaccine, mRNA-LNP, spike protein, preservative free, 30 mcg/0.3 mL dosage, tris-sucrose formulation
or	procedure	UMLS:CPT:0054A	Immunization administration by intramuscular injection of severe acute respiratory syndrome coronavirus 2 (SARS-CoV-2) (coronavirus disease [COVID-19]) vaccine, mRNA-LNP, spike protein, preservative free, 30 mcg/0.3 mL dosage, tris-sucrose formulation; booster dose
or	procedure	UMLS:CPT:0064A	Immunization administration by intramuscular injection of severe acute respiratory syndrome coronavirus 2 (SARS-CoV-2) (coronavirus disease [COVID-19]) vaccine, mRNA-LNP, spike protein, preservative free, 50 mcg/0.25 mL dosage, booster dose

or	procedure	UMLS:CPT:90480	Immunization administration by intramuscular injection of severe acute respiratory syndrome coronavirus 2 (SARS-CoV-2) (coronavirus disease [COVID-19]) vaccine, single dose
or	procedure	UMLS:CPT:1037171	Immunization administration by intramuscular injection of severe acute respiratory syndrome coronavirus 2 (SARS-CoV-2) (coronavirus disease [COVID-19]) vaccine, mRNA-LNP, spike protein, preservative free, 10 mcg/0.2 mL dosage, diluent reconstituted, tris-sucrose formulation
or	procedure	UMLS:CPT:0071A	Immunization administration by intramuscular injection of severe acute respiratory syndrome coronavirus 2 (SARS-CoV-2) (coronavirus disease [COVID-19]) vaccine, mRNA-LNP, spike protein, preservative free, 10 mcg/0.2 mL dosage, diluent reconstituted, tris-sucrose formulation; first dose

or	procedure	UMLS:CPT:0072A	Immunization administration by intramuscular injection of severe acute respiratory syndrome coronavirus 2 (SARS-CoV-2) (coronavirus disease [COVID-19]) vaccine, mRNA-LNP, spike protein, preservative free, 10 mcg/0.2 mL dosage, diluent reconstituted, tris-sucrose formulation; second dose
or	medicatio n	NLM:RXNORM:261 0319	SARS-CoV-2 (COVID-19) vaccine, mRNA-BNT162b2 0.05 MG/ML / SARS-CoV-2 (COVID-19) vaccine, mRNA-BNT162b2 OMICRON (BA.4/BA.5) 0.05 MG/ML Injectable Suspension
or	procedure	UMLS:CPT:91313	Severe acute respiratory syndrome coronavirus 2 (SARS-CoV-2) (coronavirus disease [COVID-19]) vaccine, mRNA-LNP, spike protein, bivalent, preservative free, 50 mcg/0.5 mL dosage, for intramuscular use
or	procedure	UMLS:CPT:0134A	Immunization administration by intramuscular injection of severe acute respiratory

			syndrome coronavirus 2 (SARS-CoV-2) (coronavirus disease [COVID-19]) vaccine, mRNA-LNP, spike protein, bivalent, preservative free, 50 mcg/0.5 mL dosage, booster dose
or	procedure	UMLS:CPT:1037175	Immunization administration by intramuscular injection of severe acute respiratory syndrome coronavirus 2 (SARS-CoV-2) (coronavirus disease [COVID-19]) vaccine, DNA, spike protein, adenovirus type 26 (Ad26) vector, preservative free, 5x1010 viral particles/0.5 mL dosage
or	medication	NLM:RXNORM:2610347	0.3 ML SARS-CoV-2 (COVID-19) vaccine, mRNA-BNT162b2 0.05 MG/ML / SARS-CoV-2 (COVID-19) vaccine, mRNA-BNT162b2 OMICRON (BA.4/BA.5) -1 MG/ML Injection
or	procedure	UMLS:CPT:1037228	Immunization administration by intramuscular injection of severe acute respiratory syndrome coronavirus 2

			(SARS-CoV-2) (coronavirus disease [COVID-19]) vaccine, mRNA-LNP, spike protein, preservative free, 3 mcg/0.2 mL dosage, diluent reconstituted, tris-sucrose formulation
or	procedure	UMLS:CPT:0013A	Immunization administration by intramuscular injection of severe acute respiratory syndrome coronavirus 2 (SARS-CoV-2) (coronavirus disease [COVID-19]) vaccine, mRNA-LNP, spike protein, preservative free, 100 mcg/0.5 mL dosage; third dose
or	procedure	UMLS:CPT:0081A	Immunization administration by intramuscular injection of severe acute respiratory syndrome coronavirus 2 (SARS-CoV-2) (coronavirus disease [COVID-19]) vaccine, mRNA-LNP, spike protein, preservative free, 3 mcg/0.2 mL dosage, diluent reconstituted, tris-sucrose formulation; first dose
or	procedure	UMLS:CPT:0082A	Immunization administration by

			intramuscular injection of severe acute respiratory syndrome coronavirus 2 (SARS-CoV-2) (coronavirus disease [COVID-19]) vaccine, mRNA-LNP, spike protein, preservative free, 3 mcg/0.2 mL dosage, diluent reconstituted, tris-sucrose formulation; second dose
or	medication	NLM:RXNORM:2610328	SARS-CoV-2 (COVID-19) vaccine, mRNA-1273 0.05 MG/ML / SARS-CoV-2 (COVID-19) vaccine, mRNA-1273 OMICRON (BA.4/BA.5) 0.05 MG/ML Injectable Suspension
or	procedure	UMLS:CPT:0154A	Immunization administration by intramuscular injection of severe acute respiratory syndrome coronavirus 2 (SARS-CoV-2) (coronavirus disease [COVID-19]) vaccine, mRNA-LNP, bivalent spike protein, preservative free, 10 mcg/0.2 mL dosage, diluent reconstituted, tris-sucrose formulation, booster dose
or	procedure	UMLS:CPT:0053A	Immunization administration by

			intramuscular injection of severe acute respiratory syndrome coronavirus 2 (SARS-CoV-2) (coronavirus disease [COVID-19]) vaccine, mRNA-LNP, spike protein, preservative free, 30 mcg/0.3 mL dosage, tris-sucrose formulation; third dose
or	procedure	UMLS:CPT:1037332	Immunization administration by intramuscular injection of severe acute respiratory syndrome coronavirus 2 (SARS-CoV-2) (coronavirus disease [COVID-19]) vaccine, mRNA-LNP, spike protein, preservative free, 25 mcg/0.25 mL dosage
or	procedure	UMLS:CPT:0052A	Immunization administration by intramuscular injection of severe acute respiratory syndrome coronavirus 2 (SARS-CoV-2) (coronavirus disease [COVID-19]) vaccine, mRNA-LNP, spike protein, preservative free, 30 mcg/0.3 mL dosage, tris-sucrose formulation; second dose

or	procedure	UMLS:CPT:0111A	Immunization administration by intramuscular injection of severe acute respiratory syndrome coronavirus 2 (SARS-CoV-2) (coronavirus disease [COVID-19]) vaccine, mRNA-LNP, spike protein, preservative free, 25 mcg/0.25 mL dosage; first dose
or	procedure	UMLS:CPT:0051A	Immunization administration by intramuscular injection of severe acute respiratory syndrome coronavirus 2 (SARS-CoV-2) (coronavirus disease [COVID-19]) vaccine, mRNA-LNP, spike protein, preservative free, 30 mcg/0.3 mL dosage, tris-sucrose formulation; first dose
or	procedure	UMLS:CPT:91311	Severe acute respiratory syndrome coronavirus 2 (SARS-CoV-2) (coronavirus disease [COVID-19]) vaccine, mRNA-LNP, spike protein, preservative free, 25 mcg/0.25 mL dosage, for intramuscular use

or	procedure	UMLS:CPT:0074A	Immunization administration by intramuscular injection of severe acute respiratory syndrome coronavirus 2 (SARS-CoV-2) (coronavirus disease [COVID-19]) vaccine, mRNA-LNP, spike protein, preservative free, 10 mcg/0.2 mL dosage, diluent reconstituted, tris-sucrose formulation; booster dose
or	procedure	UMLS:CPT:0112A	Immunization administration by intramuscular injection of severe acute respiratory syndrome coronavirus 2 (SARS-CoV-2) (coronavirus disease [COVID-19]) vaccine, mRNA-LNP, spike protein, preservative free, 25 mcg/0.25 mL dosage; second dose
or	procedure	UMLS:CPT:0083A	Immunization administration by intramuscular injection of severe acute respiratory syndrome coronavirus 2 (SARS-CoV-2) (coronavirus disease [COVID-19]) vaccine, mRNA-LNP, spike protein, preservative free, 3

			mcg/0.2 mL dosage, diluent reconstituted, tris-sucrose formulation; third dose
or	procedure	UMLS:CPT:0073A	Immunization administration by intramuscular injection of severe acute respiratory syndrome coronavirus 2 (SARS-CoV-2) (coronavirus disease [COVID-19]) vaccine, mRNA-LNP, spike protein, preservative free, 10 mcg/0.2 mL dosage, diluent reconstituted, tris-sucrose formulation; third dose
or	procedure	UMLS:CPT:0173A	Immunization administration by intramuscular injection of severe acute respiratory syndrome coronavirus 2 (SARS-CoV-2) (coronavirus disease [COVID-19]) vaccine, mRNA-LNP, bivalent spike protein, preservative free, 3 mcg/0.2 mL dosage, diluent reconstituted, tris-sucrose formulation, third dose
or	procedure	UMLS:CPT:0164A	Immunization administration by intramuscular injection of severe acute respiratory

			syndrome coronavirus 2 (SARS-CoV-2) (coronavirus disease [COVID-19]) vaccine, mRNA-LNP, spike protein, bivalent, preservative free, 10 mcg/0.2 mL dosage, booster dose
or	procedure	UMLS:CPT:1037838	Immunization administration by intramuscular injection of severe acute respiratory syndrome coronavirus 2 (SARS-CoV-2) (coronavirus disease [COVID-19]) vaccine, mRNA-LNP, spike protein, preservative free, 50 mcg/0.5 mL dosage
or	procedure	UMLS:CPT:0094A	Immunization administration by intramuscular injection of severe acute respiratory syndrome coronavirus 2 (SARS-CoV-2) (coronavirus disease [COVID-19]) vaccine, mRNA-LNP, spike protein, preservative free, 50 mcg/0.5 mL dosage; booster dose, when administered to individuals 18 years and over

or	procedure	UMLS:CPT:0034A	Immunization administration by intramuscular injection of severe acute respiratory syndrome coronavirus 2 (SARS-CoV-2) (coronavirus disease [COVID-19]) vaccine, DNA, spike protein, adenovirus type 26 (Ad26) vector, preservative free, 5x1010 viral particles/0.5 mL dosage; booster dose
or	procedure	UMLS:CPT:0144A	Immunization administration by intramuscular injection of severe acute respiratory syndrome coronavirus 2 (SARS-CoV-2) (coronavirus disease [COVID-19]) vaccine, mRNA-LNP, spike protein, bivalent, preservative free, 25 mcg/0.25 mL dosage, booster dose
or	procedure	UMLS:CPT:0091A	Immunization administration by intramuscular injection of severe acute respiratory syndrome coronavirus 2 (SARS-CoV-2) (coronavirus disease [COVID-19]) vaccine, mRNA-LNP, spike

			protein, preservative free, 50 mcg/0.5 mL dosage; first dose, when administered to individuals 6 through 11 years
or	procedure	UMLS:CPT:0174A	Immunization administration by intramuscular injection of severe acute respiratory syndrome coronavirus 2 (SARS-CoV-2) (coronavirus disease [COVID-19]) vaccine, mRNA-LNP, bivalent spike protein, preservative free, 3 mcg/0.2 mL dosage, diluent reconstituted, tris-sucrose formulation, booster
or	procedure	UMLS:CPT:0092A	Immunization administration by intramuscular injection of severe acute respiratory syndrome coronavirus 2 (SARS-CoV-2) (coronavirus disease [COVID-19]) vaccine, mRNA-LNP, spike protein, preservative free, 50 mcg/0.5 mL dosage; second dose, when administered to individuals 6 through 11 years

or	procedure	UMLS:CPT:1036682	Immunization administration by intramuscular injection of severe acute respiratory syndrome coronavirus 2 (SARS-CoV-2) (coronavirus disease [COVID-19]) vaccine, recombinant spike protein nanoparticle, saponin-based adjuvant, preservative free, 5 mcg/0.5 mL dosage
or	procedure	UMLS:CPT:0041A	Immunization administration by intramuscular injection of severe acute respiratory syndrome coronavirus 2 (SARS-CoV-2) (coronavirus disease [COVID-19]) vaccine, recombinant spike protein nanoparticle, saponin-based adjuvant, preservative free, 5 mcg/0.5 mL dosage; first dose
or	procedure	UMLS:CPT:0113A	Immunization administration by intramuscular injection of severe acute respiratory syndrome coronavirus 2 (SARS-CoV-2) (coronavirus disease [COVID-19]) vaccine, mRNA-LNP, spike

			protein, preservative free, 25 mcg/0.25 mL dosage; third dose
or	procedure	UMLS:CPT:0042A	Immunization administration by intramuscular injection of severe acute respiratory syndrome coronavirus 2 (SARS-CoV-2) (coronavirus disease [COVID-19]) vaccine, recombinant spike protein nanoparticle, saponin-based adjuvant, preservative free, 5 mcg/0.5 mL dosage; second dose
or	procedure	UMLS:CPT:0093A	Immunization administration by intramuscular injection of severe acute respiratory syndrome coronavirus 2 (SARS-CoV-2) (coronavirus disease [COVID-19]) vaccine, mRNA-LNP, spike protein, preservative free, 50 mcg/0.5 mL dosage; third dose, when administered to individuals 6 through 11 years
or	procedure	UMLS:CPT:1036666	Immunization administration by intramuscular injection of severe acute respiratory

				syndrome coronavirus 2 (SARS-CoV-2) (coronavirus disease [COVID-19]) vaccine, DNA, spike protein, chimpanzee adenovirus Oxford 1 (ChAdOx1) vector, preservative free, 5x1010 viral particles/0.5 mL dosage
	or	procedure	UMLS:CPT:0044A	Immunization administration by intramuscular injection of severe acute respiratory syndrome coronavirus 2 (SARS-CoV-2) (coronavirus disease [COVID-19]) vaccine, recombinant spike protein nanoparticle, saponin-based adjuvant, preservative free, 5 mcg/0.5mL dosage; booster
date constraint		The terms in this group occurred between Dec 1, 2019 and Dec 31, 2023		
**Group 2**				
**Group 2A COVID-19**				
must have	any of	diagnosis	UMLS:ICD10CM:U07.1	COVID-19
		diagnosis	UMLS:ICD10CM:U07.2	COVID-19, virus not identified (WHO)
		diagnosis	UMLS:ICD10CM:J1	Pneumonia due to

	2.82	coronavirus disease 2019
laboratory	UMLS:LNC:94500-6	SARS-CoV-2 (COVID-19) RNA [Presence] in Respiratory specimen by NAA with probe detection (labResult: Positive)
laboratory	UMLS:LNC:94309-2	SARS-CoV-2 (COVID-19) RNA [Presence] in Specimen by NAA with probe detection (labResult: Positive)
laboratory	UMLS:LNC:94565-9	SARS-CoV-2 (COVID-19) RNA [Presence] in Nasopharynx by NAA with non-probe detection (labResult: Positive)
laboratory	UMLS:LNC:94759-8	SARS-CoV-2 (COVID-19) RNA [Presence] in Nasopharynx by NAA with probe detection (labResult: Positive)
laboratory	UMLS:LNC:95608-6	SARS-CoV-2 (COVID-19) RNA [Presence] in Respiratory specimen by NAA with non-probe detection (labResult: Positive)
laboratory	UMLS:LNC:94845-5	SARS-CoV-2 (COVID-19) RNA [Presence] in Saliva (oral fluid) by NAA with probe detection (labResult:

				Positive)
		laboratory	UMLS:LNC:95406-5	SARS-CoV-2 (COVID-19) RNA [Presence] in Nose by NAA with probe detection (labResult: Positive)
	and	visit	TNX:Visit	Visit (Data Source: TriNetX)
date constraint		The terms in this group occurred at any time		
event relationship		Any instance of thyroid cancer history occurred at least 1 day before the first instance of COVID-19		
**Group 2B thyroid cancer history**				
cannot have		diagnosis	UMLS:ICD10CM:C73	Malignant neoplasm of thyroid gland
	or	diagnosis	UMLS:ICD10CM:Z85.850	Personal history of malignant neoplasm of thyroid

Query Criteria for Cohort post-COVID, >60

This query was run on the network Global Collaborative Network with 148 HCO(s) queried and 148 HCO(s) responded. A total of 122 provider(s) responded with patients. The final cohort included 897,532 patients who matched the query criteria listed in the table below.

**Ungrouped terms**				
must have		demographics	Age	Age (at least 61 years (most recent occurrence))
	and any of	demographics	UMLS:HL7V3.0:Gender:M	Male
		demograp	UMLS:HL7V3.0:Ge	Female

		hics	nder:F	
**Group 1**				
**COVID-19, no vaccination**				
must have	any of	diagnosis	UMLS:ICD10CM:U07.1	COVID-19
		diagnosis	UMLS:ICD10CM:U07.2	COVID-19, virus not identified (WHO)
		diagnosis	UMLS:ICD10CM:J12.82	Pneumonia due to coronavirus disease 2019
		laboratory	UMLS:LNC:94500-6	SARS-CoV-2 (COVID-19) RNA [Presence] in Respiratory specimen by NAA with probe detection (labResult: Positive)
		laboratory	UMLS:LNC:94309-2	SARS-CoV-2 (COVID-19) RNA [Presence] in Specimen by NAA with probe detection (labResult: Positive)
		laboratory	UMLS:LNC:94565-9	SARS-CoV-2 (COVID-19) RNA [Presence] in Nasopharynx by NAA with non-probe detection (labResult: Positive)
		laboratory	UMLS:LNC:94759-8	SARS-CoV-2 (COVID-19) RNA [Presence] in Nasopharynx by NAA with probe detection (labResult: Positive)
		laboratory	UMLS:LNC:95608-	SARS-CoV-2 (COVID-19) RNA [Presence] in

			6	Respiratory specimen by NAA with non-probe detection (labResult: Positive)
		laboratory	UMLS:LNC:94845-5	SARS-CoV-2 (COVID-19) RNA [Presence] in Saliva (oral fluid) by NAA with probe detection (labResult: Positive)
		laboratory	UMLS:LNC:95406-5	SARS-CoV-2 (COVID-19) RNA [Presence] in Nose by NAA with probe detection (labResult: Positive)
cannot have		medication	NLM:CVX:208	COVID-19, mRNA, LNP-S, PF, 30 mcg/0.3 mL dose
	or	medication	NLM:CVX:207	COVID-19, mRNA, LNP-S, PF, 100 mcg/0.5mL dose or 50 mcg/0.25mL dose
	or	medication	NLM:CVX:212	COVID-19 vaccine, vector-nr, rS-Ad26, PF, 0.5 mL
	or	medication	NLM:RXNORM:OMOP5042939	COVID-19 vaccine
	or	medication	NLM:CVX:300	COVID-19, mRNA, LNP-S, bivalent, PF, 30 mcg/0.3 mL dose
	or	medication	NLM:CVX:217	COVID-19, mRNA, LNP-S, PF, 30 mcg/0.3 mL dose, tris-sucrose
	or	medicatio	NLM:CVX:229	COVID-19, mRNA, LNP-S,

	n		bivalent, PF, 50 mcg/0.5 mL or 25mcg/0.25 mL dose
or	medication	NLM:CVX:218	COVID-19, mRNA, LNP-S, PF, 10 mcg/0.2 mL dose, tris-sucrose
or	medication	NLM:CVX:520	COVID-19 mRNA, bivalent, original/Omicron BA.1, Non-US Vaccine Product, Pfizer-BioNTech
or	medication	NLM:CVX:519	COVID-19 mRNA, bivalent, original/Omicron BA.1, Non-US Vaccine (Spikevax Bivalent), Moderna
or	medication	NLM:CVX:301	COVID-19, mRNA, LNP-S, bivalent, PF, 10 mcg/0.2 mL dose
or	medication	NLM:CVX:219	COVID-19, mRNA, LNP-S, PF, 3 mcg/0.2 mL dose, tris-sucrose
or	medication	NLM:CVX:228	COVID-19, mRNA, LNP-S, PF, pediatric 25 mcg/0.25 mL dose
or	medication	NLM:CVX:230	COVID-19, mRNA, LNP-S, bivalent booster, PF, 10 mcg/0.2 mL
or	medication	NLM:CVX:221	COVID-19, mRNA, LNP-S, PF, 50 mcg/0.5 mL dose
or	medication	NLM:CVX:210	COVID-19 vaccine, vector-nr, rS-ChAdOx1, PF, 0.5 mL

or	medication	NLM:CVX:302	COVID-19, mRNA, LNP-S, bivalent, PF, 3 mcg/0.2 mL dose
or	medication	NLM:CVX:511	COVID-19 IV Non-US Vaccine (CoronaVac, Sinovac)
or	medication	NLM:RXNORM:2468231	SARS-CoV-2 (COVID-19) vaccine, mRNA spike protein
or	procedure	UMLS:CPT:91300	Severe acute respiratory syndrome coronavirus 2 (SARS-CoV-2) (coronavirus disease [COVID-19]) vaccine, mRNA-LNP, spike protein, preservative free, 30 mcg/0.3 mL dosage, diluent reconstituted, for intramuscular use
or	procedure	UMLS:CPT:0001A	Immunization administration by intramuscular injection of severe acute respiratory syndrome coronavirus 2 (SARS-CoV-2) (coronavirus disease [COVID-19]) vaccine, mRNA-LNP, spike protein, preservative free, 30 mcg/0.3 mL dosage, diluent reconstituted; first dose
or	procedure	UMLS:CPT:0002A	Immunization administration by

			intramuscular injection of severe acute respiratory syndrome coronavirus 2 (SARS-CoV-2) (coronavirus disease [COVID-19]) vaccine, mRNA-LNP, spike protein, preservative free, 30 mcg/0.3 mL dosage, diluent reconstituted; second dose
or	procedure	UMLS:CPT:91301	Severe acute respiratory syndrome coronavirus 2 (SARS-CoV-2) (coronavirus disease [COVID-19]) vaccine, mRNA-LNP, spike protein, preservative free, 100 mcg/0.5 mL dosage, for intramuscular use
or	procedure	UMLS:CPT:0011A	Immunization administration by intramuscular injection of severe acute respiratory syndrome coronavirus 2 (SARS-CoV-2) (coronavirus disease [COVID-19]) vaccine, mRNA-LNP, spike protein, preservative free, 100 mcg/0.5 mL dosage; first dose
or	procedure	UMLS:CPT:0012A	Immunization administration by intramuscular injection of severe acute respiratory

			syndrome coronavirus 2 (SARS-CoV-2) (coronavirus disease [COVID-19]) vaccine, mRNA-LNP, spike protein, preservative free, 100 mcg/0.5 mL dosage; second dose
or	procedure	UMLS:SNOMED:840534001	Administration of SARS-CoV-2 antigen vaccine
or	medication	NLM:CVX:213	SARS-CoV-2 (COVID-19) Vaccine
or	procedure	UMLS:CPT:1036660	Immunization administration by intramuscular injection of severe acute respiratory syndrome coronavirus 2 (SARS-CoV-2) (coronavirus disease [COVID-19]) vaccine, mRNA-LNP, spike protein, preservative free, 30 mcg/0.3 mL dosage, diluent reconstituted
or	procedure	UMLS:CPT:1036663	Immunization administration by intramuscular injection of severe acute respiratory syndrome coronavirus 2 (SARS-CoV-2) (coronavirus disease [COVID-19]) vaccine, mRNA-LNP, spike protein, preservative free,

			100 mcg/0.5 mL dosage
or	procedure	UMLS:CPT:0124A	Immunization administration by intramuscular injection of severe acute respiratory syndrome coronavirus 2 (SARS-CoV-2) (coronavirus disease [COVID-19]) vaccine, mRNA-LNP, bivalent spike protein, preservative free, 30 mcg/0.3 mL dosage, tris-sucrose formulation, booster dose
or	procedure	UMLS:CPT:0004A	Immunization administration by intramuscular injection of severe acute respiratory syndrome coronavirus 2 (SARS-CoV-2) (coronavirus disease [COVID-19]) vaccine, mRNA-LNP, spike protein, preservative free, 30 mcg/0.3 mL dosage, diluent reconstituted; booster dose
or	procedure	UMLS:CPT:0003A	Immunization administration by intramuscular injection of severe acute respiratory syndrome coronavirus 2 (SARS-CoV-2) (coronavirus disease [COVID-19])

			vaccine, mRNA-LNP, spike protein, preservative free, 30 mcg/0.3 mL dosage, diluent reconstituted; third dose
or	procedure	UMLS:CPT:1037166	Immunization administration by intramuscular injection of severe acute respiratory syndrome coronavirus 2 (SARS-CoV-2) (coronavirus disease [COVID-19]) vaccine, mRNA-LNP, spike protein, preservative free, 30 mcg/0.3 mL dosage, tris-sucrose formulation
or	procedure	UMLS:CPT:0054A	Immunization administration by intramuscular injection of severe acute respiratory syndrome coronavirus 2 (SARS-CoV-2) (coronavirus disease [COVID-19]) vaccine, mRNA-LNP, spike protein, preservative free, 30 mcg/0.3 mL dosage, tris-sucrose formulation; booster dose
or	procedure	UMLS:CPT:0064A	Immunization administration by intramuscular injection of severe acute respiratory syndrome coronavirus 2

			(SARS-CoV-2) (coronavirus disease [COVID-19]) vaccine, mRNA-LNP, spike protein, preservative free, 50 mcg/0.25 mL dosage, booster dose
or	procedure	UMLS:CPT:90480	Immunization administration by intramuscular injection of severe acute respiratory syndrome coronavirus 2 (SARS-CoV-2) (coronavirus disease [COVID-19]) vaccine, single dose
or	procedure	UMLS:CPT:1037171	Immunization administration by intramuscular injection of severe acute respiratory syndrome coronavirus 2 (SARS-CoV-2) (coronavirus disease [COVID-19]) vaccine, mRNA-LNP, spike protein, preservative free, 10 mcg/0.2 mL dosage, diluent reconstituted, tris-sucrose formulation
or	procedure	UMLS:CPT:0071A	Immunization administration by intramuscular injection of severe acute respiratory syndrome coronavirus 2 (SARS-CoV-2) (coronavirus

			disease [COVID-19]) vaccine, mRNA-LNP, spike protein, preservative free, 10 mcg/0.2 mL dosage, diluent reconstituted, tris-sucrose formulation; first dose
or	procedure	UMLS:CPT:0072A	Immunization administration by intramuscular injection of severe acute respiratory syndrome coronavirus 2 (SARS-CoV-2) (coronavirus disease [COVID-19]) vaccine, mRNA-LNP, spike protein, preservative free, 10 mcg/0.2 mL dosage, diluent reconstituted, tris-sucrose formulation; second dose
or	medicatio n	NLM:RXNORM:261 0319	SARS-CoV-2 (COVID-19) vaccine, mRNA-BNT162b2 0.05 MG/ML / SARS-CoV-2 (COVID-19) vaccine, mRNA-BNT162b2 OMICRON (BA.4/BA.5) 0.05 MG/ML Injectable Suspension
or	procedure	UMLS:CPT:91313	Severe acute respiratory syndrome coronavirus 2 (SARS-CoV-2) (coronavirus disease [COVID-19]) vaccine, mRNA-LNP, spike protein, bivalent, preservative free, 50

			mcg/0.5 mL dosage, for intramuscular use
or	procedure	UMLS:CPT:0134A	Immunization administration by intramuscular injection of severe acute respiratory syndrome coronavirus 2 (SARS-CoV-2) (coronavirus disease [COVID-19]) vaccine, mRNA-LNP, spike protein, bivalent, preservative free, 50 mcg/0.5 mL dosage, booster dose
or	procedure	UMLS:CPT:1037175	Immunization administration by intramuscular injection of severe acute respiratory syndrome coronavirus 2 (SARS-CoV-2) (coronavirus disease [COVID-19]) vaccine, DNA, spike protein, adenovirus type 26 (Ad26) vector, preservative free, 5x1010 viral particles/0.5 mL dosage
or	medication	NLM:RXNORM:2610347	0.3 ML SARS-CoV-2 (COVID-19) vaccine, mRNA-BNT162b2 0.05 MG/ML / SARS-CoV-2 (COVID-19) vaccine, mRNA-BNT162b2 OMICRON (BA.4/BA.5) -1

			MG/ML Injection
or	procedure	UMLS:CPT:1037228	Immunization administration by intramuscular injection of severe acute respiratory syndrome coronavirus 2 (SARS-CoV-2) (coronavirus disease [COVID-19]) vaccine, mRNA-LNP, spike protein, preservative free, 3 mcg/0.2 mL dosage, diluent reconstituted, tris-sucrose formulation
or	procedure	UMLS:CPT:0013A	Immunization administration by intramuscular injection of severe acute respiratory syndrome coronavirus 2 (SARS-CoV-2) (coronavirus disease [COVID-19]) vaccine, mRNA-LNP, spike protein, preservative free, 100 mcg/0.5 mL dosage; third dose
or	procedure	UMLS:CPT:0081A	Immunization administration by intramuscular injection of severe acute respiratory syndrome coronavirus 2 (SARS-CoV-2) (coronavirus disease [COVID-19]) vaccine, mRNA-LNP, spike

			protein, preservative free, 3 mcg/0.2 mL dosage, diluent reconstituted, tris-sucrose formulation; first dose
or	procedure	UMLS:CPT:0082A	Immunization administration by intramuscular injection of severe acute respiratory syndrome coronavirus 2 (SARS-CoV-2) (coronavirus disease [COVID-19]) vaccine, mRNA-LNP, spike protein, preservative free, 3 mcg/0.2 mL dosage, diluent reconstituted, tris-sucrose formulation; second dose
or	medicatio n	NLM:RXNORM:261 0328	SARS-CoV-2 (COVID-19) vaccine, mRNA-1273 0.05 MG/ML / SARS-CoV-2 (COVID-19) vaccine, mRNA-1273 OMICRON (BA.4/BA.5) 0.05 MG/ML Injectable Suspension
or	procedure	UMLS:CPT:0154A	Immunization administration by intramuscular injection of severe acute respiratory syndrome coronavirus 2 (SARS-CoV-2) (coronavirus disease [COVID-19]) vaccine, mRNA-LNP, bivalent spike protein,

			preservative free, 10 mcg/0.2 mL dosage, diluent reconstituted, tris-sucrose formulation, booster dose
or	procedure	UMLS:CPT:0053A	Immunization administration by intramuscular injection of severe acute respiratory syndrome coronavirus 2 (SARS-CoV-2) (coronavirus disease [COVID-19]) vaccine, mRNA-LNP, spike protein, preservative free, 30 mcg/0.3 mL dosage, tris-sucrose formulation; third dose
or	procedure	UMLS:CPT:1037332	Immunization administration by intramuscular injection of severe acute respiratory syndrome coronavirus 2 (SARS-CoV-2) (coronavirus disease [COVID-19]) vaccine, mRNA-LNP, spike protein, preservative free, 25 mcg/0.25 mL dosage
or	procedure	UMLS:CPT:0052A	Immunization administration by intramuscular injection of severe acute respiratory syndrome coronavirus 2 (SARS-CoV-2) (coronavirus

			disease [COVID-19]) vaccine, mRNA-LNP, spike protein, preservative free, 30 mcg/0.3 mL dosage, tris-sucrose formulation; second dose
or	procedure	UMLS:CPT:0111A	Immunization administration by intramuscular injection of severe acute respiratory syndrome coronavirus 2 (SARS-CoV-2) (coronavirus disease [COVID-19]) vaccine, mRNA-LNP, spike protein, preservative free, 25 mcg/0.25 mL dosage; first dose
or	procedure	UMLS:CPT:0051A	Immunization administration by intramuscular injection of severe acute respiratory syndrome coronavirus 2 (SARS-CoV-2) (coronavirus disease [COVID-19]) vaccine, mRNA-LNP, spike protein, preservative free, 30 mcg/0.3 mL dosage, tris-sucrose formulation; first dose
or	procedure	UMLS:CPT:91311	Severe acute respiratory syndrome coronavirus 2 (SARS-CoV-2) (coronavirus

			disease [COVID-19]) vaccine, mRNA-LNP, spike protein, preservative free, 25 mcg/0.25 mL dosage, for intramuscular use
or	procedure	UMLS:CPT:0074A	Immunization administration by intramuscular injection of severe acute respiratory syndrome coronavirus 2 (SARS-CoV-2) (coronavirus disease [COVID-19]) vaccine, mRNA-LNP, spike protein, preservative free, 10 mcg/0.2 mL dosage, diluent reconstituted, tris-sucrose formulation; booster dose
or	procedure	UMLS:CPT:0112A	Immunization administration by intramuscular injection of severe acute respiratory syndrome coronavirus 2 (SARS-CoV-2) (coronavirus disease [COVID-19]) vaccine, mRNA-LNP, spike protein, preservative free, 25 mcg/0.25 mL dosage; second dose
or	procedure	UMLS:CPT:0083A	Immunization administration by intramuscular injection of severe acute respiratory

			syndrome coronavirus 2 (SARS-CoV-2) (coronavirus disease [COVID-19]) vaccine, mRNA-LNP, spike protein, preservative free, 3 mcg/0.2 mL dosage, diluent reconstituted, tris-sucrose formulation; third dose
or	procedure	UMLS:CPT:0073A	Immunization administration by intramuscular injection of severe acute respiratory syndrome coronavirus 2 (SARS-CoV-2) (coronavirus disease [COVID-19]) vaccine, mRNA-LNP, spike protein, preservative free, 10 mcg/0.2 mL dosage, diluent reconstituted, tris-sucrose formulation; third dose
or	procedure	UMLS:CPT:0173A	Immunization administration by intramuscular injection of severe acute respiratory syndrome coronavirus 2 (SARS-CoV-2) (coronavirus disease [COVID-19]) vaccine, mRNA-LNP, bivalent spike protein, preservative free, 3 mcg/0.2 mL dosage, diluent reconstituted, tris-sucrose

			formulation, third dose
or	procedure	UMLS:CPT:0164A	Immunization administration by intramuscular injection of severe acute respiratory syndrome coronavirus 2 (SARS-CoV-2) (coronavirus disease [COVID-19]) vaccine, mRNA-LNP, spike protein, bivalent, preservative free, 10 mcg/0.2 mL dosage, booster dose
or	procedure	UMLS:CPT:1037838	Immunization administration by intramuscular injection of severe acute respiratory syndrome coronavirus 2 (SARS-CoV-2) (coronavirus disease [COVID-19]) vaccine, mRNA-LNP, spike protein, preservative free, 50 mcg/0.5 mL dosage
or	procedure	UMLS:CPT:0094A	Immunization administration by intramuscular injection of severe acute respiratory syndrome coronavirus 2 (SARS-CoV-2) (coronavirus disease [COVID-19]) vaccine, mRNA-LNP, spike protein, preservative free, 50

			mcg/0.5 mL dosage; booster dose, when administered to individuals 18 years and over
or	procedure	UMLS:CPT:0034A	Immunization administration by intramuscular injection of severe acute respiratory syndrome coronavirus 2 (SARS-CoV-2) (coronavirus disease [COVID-19]) vaccine, DNA, spike protein, adenovirus type 26 (Ad26) vector, preservative free, 5x1010 viral particles/0.5 mL dosage; booster dose
or	procedure	UMLS:CPT:0144A	Immunization administration by intramuscular injection of severe acute respiratory syndrome coronavirus 2 (SARS-CoV-2) (coronavirus disease [COVID-19]) vaccine, mRNA-LNP, spike protein, bivalent, preservative free, 25 mcg/0.25 mL dosage, booster dose
or	procedure	UMLS:CPT:0091A	Immunization administration by intramuscular injection of severe acute respiratory

			syndrome coronavirus 2 (SARS-CoV-2) (coronavirus disease [COVID-19]) vaccine, mRNA-LNP, spike protein, preservative free, 50 mcg/0.5 mL dosage; first dose, when administered to individuals 6 through 11 years
or	procedure	UMLS:CPT:0174A	Immunization administration by intramuscular injection of severe acute respiratory syndrome coronavirus 2 (SARS-CoV-2) (coronavirus disease [COVID-19]) vaccine, mRNA-LNP, bivalent spike protein, preservative free, 3 mcg/0.2 mL dosage, diluent reconstituted, tris-sucrose formulation, booster
or	procedure	UMLS:CPT:0092A	Immunization administration by intramuscular injection of severe acute respiratory syndrome coronavirus 2 (SARS-CoV-2) (coronavirus disease [COVID-19]) vaccine, mRNA-LNP, spike protein, preservative free, 50 mcg/0.5 mL dosage; second

			dose, when administered to individuals 6 through 11 years
or	procedure	UMLS:CPT:1036682	Immunization administration by intramuscular injection of severe acute respiratory syndrome coronavirus 2 (SARS-CoV-2) (coronavirus disease [COVID-19]) vaccine, recombinant spike protein nanoparticle, saponin-based adjuvant, preservative free, 5 mcg/0.5 mL dosage
or	procedure	UMLS:CPT:0041A	Immunization administration by intramuscular injection of severe acute respiratory syndrome coronavirus 2 (SARS-CoV-2) (coronavirus disease [COVID-19]) vaccine, recombinant spike protein nanoparticle, saponin-based adjuvant, preservative free, 5 mcg/0.5 mL dosage; first dose
or	procedure	UMLS:CPT:0113A	Immunization administration by intramuscular injection of severe acute respiratory syndrome coronavirus 2

			(SARS-CoV-2) (coronavirus disease [COVID-19]) vaccine, mRNA-LNP, spike protein, preservative free, 25 mcg/0.25 mL dosage; third dose
or	procedure	UMLS:CPT:0042A	Immunization administration by intramuscular injection of severe acute respiratory syndrome coronavirus 2 (SARS-CoV-2) (coronavirus disease [COVID-19]) vaccine, recombinant spike protein nanoparticle, saponin-based adjuvant, preservative free, 5 mcg/0.5 mL dosage; second dose
or	procedure	UMLS:CPT:0093A	Immunization administration by intramuscular injection of severe acute respiratory syndrome coronavirus 2 (SARS-CoV-2) (coronavirus disease [COVID-19]) vaccine, mRNA-LNP, spike protein, preservative free, 50 mcg/0.5 mL dosage; third dose, when administered to individuals 6 through 11 years

	or	procedure	UMLS:CPT:1036666	Immunization administration by intramuscular injection of severe acute respiratory syndrome coronavirus 2 (SARS-CoV-2) (coronavirus disease [COVID-19]) vaccine, DNA, spike protein, chimpanzee adenovirus Oxford 1 (ChAdOx1) vector, preservative free, 5x1010 viral particles/0.5 mL dosage
	or	procedure	UMLS:CPT:0044A	Immunization administration by intramuscular injection of severe acute respiratory syndrome coronavirus 2 (SARS-CoV-2) (coronavirus disease [COVID-19]) vaccine, recombinant spike protein nanoparticle, saponin-based adjuvant, preservative free, 5 mcg/0.5mL dosage; booster
date constraint		The terms in this group occurred between Dec 1, 2019 and Dec 31, 2023		

Group 2

Group 2A COVID-19

must have	any of	diagnosis	UMLS:ICD10CM:U07.1	COVID-19

diagnosis	UMLS:ICD10CM:U07.2	COVID-19, virus not identified (WHO)
diagnosis	UMLS:ICD10CM:J12.82	Pneumonia due to coronavirus disease 2019
laboratory	UMLS:LNC:94500-6	SARS-CoV-2 (COVID-19) RNA [Presence] in Respiratory specimen by NAA with probe detection (labResult: Positive)
laboratory	UMLS:LNC:94309-2	SARS-CoV-2 (COVID-19) RNA [Presence] in Specimen by NAA with probe detection (labResult: Positive)
laboratory	UMLS:LNC:94565-9	SARS-CoV-2 (COVID-19) RNA [Presence] in Nasopharynx by NAA with non-probe detection (labResult: Positive)
laboratory	UMLS:LNC:94759-8	SARS-CoV-2 (COVID-19) RNA [Presence] in Nasopharynx by NAA with probe detection (labResult: Positive)
laboratory	UMLS:LNC:95608-6	SARS-CoV-2 (COVID-19) RNA [Presence] in Respiratory specimen by NAA with non-probe detection (labResult: Positive)
laboratory	UMLS:LNC:94845-	SARS-CoV-2 (COVID-19)

			5	RNA [Presence] in Saliva (oral fluid) by NAA with probe detection (labResult: Positive)
		laboratory	UMLS:LNC:95406-5	SARS-CoV-2 (COVID-19) RNA [Presence] in Nose by NAA with probe detection (labResult: Positive)
	and	visit	TNX:Visit	Visit (Data Source: TriNetX)
date constraint		The terms in this group occurred at any time		
event relationship		Any instance of thyroid cancer history occurred at least 1 day before the first instance of COVID-19		
**Group 2B thyroid cancer history**				
cannot have		diagnosis	UMLS:ICD10CM:C73	Malignant neoplasm of thyroid gland
	or	diagnosis	UMLS:ICD10CM:Z85.850	Personal history of malignant neoplasm of thyroid

Query Criteria for Cohort post-COVID, 18-60

This query was run on the network Global Collaborative Network with 148 HCO(s) queried and 148 HCO(s) responded. A total of 122 provider(s) responded with patients. The final cohort included 1,548,783 patients who matched the query criteria listed in the table below.

**Ungrouped terms**				
must		demograp	Age	Age (between 18 and 60 years (most recent

have		hics		occurrence))
	and any of	demographics	UMLS:HL7V3.0:Gender:M	Male
		demographics	UMLS:HL7V3.0:Gender:F	Female
**Group 1**				
**COVID-19, no vaccination**				
must have	any of	diagnosis	UMLS:ICD10CM:U07.1	COVID-19
		diagnosis	UMLS:ICD10CM:U07.2	COVID-19, virus not identified (WHO)
		diagnosis	UMLS:ICD10CM:J12.82	Pneumonia due to coronavirus disease 2019
		laboratory	UMLS:LNC:94500-6	SARS-CoV-2 (COVID-19) RNA [Presence] in Respiratory specimen by NAA with probe detection (labResult: Positive)
		laboratory	UMLS:LNC:94309-2	SARS-CoV-2 (COVID-19) RNA [Presence] in Specimen by NAA with probe detection (labResult: Positive)
		laboratory	UMLS:LNC:94565-9	SARS-CoV-2 (COVID-19) RNA [Presence] in Nasopharynx by NAA with non-probe detection (labResult: Positive)
		laboratory	UMLS:LNC:94759-8	SARS-CoV-2 (COVID-19) RNA [Presence] in Nasopharynx by NAA with

				probe detection (labResult: Positive)
		laboratory	UMLS:LNC:95608-6	SARS-CoV-2 (COVID-19) RNA [Presence] in Respiratory specimen by NAA with non-probe detection (labResult: Positive)
		laboratory	UMLS:LNC:94845-5	SARS-CoV-2 (COVID-19) RNA [Presence] in Saliva (oral fluid) by NAA with probe detection (labResult: Positive)
		laboratory	UMLS:LNC:95406-5	SARS-CoV-2 (COVID-19) RNA [Presence] in Nose by NAA with probe detection (labResult: Positive)
cannot have		medication	NLM:CVX:208	COVID-19, mRNA, LNP-S, PF, 30 mcg/0.3 mL dose
	or	medication	NLM:CVX:207	COVID-19, mRNA, LNP-S, PF, 100 mcg/0.5mL dose or 50 mcg/0.25mL dose
	or	medication	NLM:CVX:212	COVID-19 vaccine, vector-nr, rS-Ad26, PF, 0.5 mL
	or	medication	NLM:RXNORM:OMOP5042939	COVID-19 vaccine
	or	medication	NLM:CVX:300	COVID-19, mRNA, LNP-S, bivalent, PF, 30 mcg/0.3 mL dose

or	medication	NLM:CVX:217	COVID-19, mRNA, LNP-S, PF, 30 mcg/0.3 mL dose, tris-sucrose
or	medication	NLM:CVX:229	COVID-19, mRNA, LNP-S, bivalent, PF, 50 mcg/0.5 mL or 25mcg/0.25 mL dose
or	medication	NLM:CVX:218	COVID-19, mRNA, LNP-S, PF, 10 mcg/0.2 mL dose, tris-sucrose
or	medication	NLM:CVX:520	COVID-19 mRNA, bivalent, original/Omicron BA.1, Non-US Vaccine Product, Pfizer-BioNTech
or	medication	NLM:CVX:519	COVID-19 mRNA, bivalent, original/Omicron BA.1, Non-US Vaccine (Spikevax Bivalent), Moderna
or	medication	NLM:CVX:301	COVID-19, mRNA, LNP-S, bivalent, PF, 10 mcg/0.2 mL dose
or	medication	NLM:CVX:219	COVID-19, mRNA, LNP-S, PF, 3 mcg/0.2 mL dose, tris-sucrose
or	medication	NLM:CVX:228	COVID-19, mRNA, LNP-S, PF, pediatric 25 mcg/0.25 mL dose
or	medication	NLM:CVX:230	COVID-19, mRNA, LNP-S, bivalent booster, PF, 10 mcg/0.2 mL

or	medication	NLM:CVX:221	COVID-19, mRNA, LNP-S, PF, 50 mcg/0.5 mL dose
or	medication	NLM:CVX:210	COVID-19 vaccine, vector-nr, rS-ChAdOx1, PF, 0.5 mL
or	medication	NLM:CVX:302	COVID-19, mRNA, LNP-S, bivalent, PF, 3 mcg/0.2 mL dose
or	medication	NLM:CVX:511	COVID-19 IV Non-US Vaccine (CoronaVac, Sinovac)
or	medication	NLM:RXNORM:2468231	SARS-CoV-2 (COVID-19) vaccine, mRNA spike protein
or	procedure	UMLS:CPT:91300	Severe acute respiratory syndrome coronavirus 2 (SARS-CoV-2) (coronavirus disease [COVID-19]) vaccine, mRNA-LNP, spike protein, preservative free, 30 mcg/0.3 mL dosage, diluent reconstituted, for intramuscular use
or	procedure	UMLS:CPT:0001A	Immunization administration by intramuscular injection of severe acute respiratory syndrome coronavirus 2 (SARS-CoV-2) (coronavirus disease [COVID-19]) vaccine, mRNA-LNP, spike protein, preservative free, 30

			mcg/0.3 mL dosage, diluent reconstituted; first dose
or	procedure	UMLS:CPT:0002A	Immunization administration by intramuscular injection of severe acute respiratory syndrome coronavirus 2 (SARS-CoV-2) (coronavirus disease [COVID-19]) vaccine, mRNA-LNP, spike protein, preservative free, 30 mcg/0.3 mL dosage, diluent reconstituted; second dose
or	procedure	UMLS:CPT:91301	Severe acute respiratory syndrome coronavirus 2 (SARS-CoV-2) (coronavirus disease [COVID-19]) vaccine, mRNA-LNP, spike protein, preservative free, 100 mcg/0.5 mL dosage, for intramuscular use
or	procedure	UMLS:CPT:0011A	Immunization administration by intramuscular injection of severe acute respiratory syndrome coronavirus 2 (SARS-CoV-2) (coronavirus disease [COVID-19]) vaccine, mRNA-LNP, spike protein, preservative free, 100 mcg/0.5 mL dosage;

			first dose
or	procedure	UMLS:CPT:0012A	Immunization administration by intramuscular injection of severe acute respiratory syndrome coronavirus 2 (SARS-CoV-2) (coronavirus disease [COVID-19]) vaccine, mRNA-LNP, spike protein, preservative free, 100 mcg/0.5 mL dosage; second dose
or	procedure	UMLS:SNOMED:840534001	Administration of SARS-CoV-2 antigen vaccine
or	medication	NLM:CVX:213	SARS-CoV-2 (COVID-19) Vaccine
or	procedure	UMLS:CPT:1036660	Immunization administration by intramuscular injection of severe acute respiratory syndrome coronavirus 2 (SARS-CoV-2) (coronavirus disease [COVID-19]) vaccine, mRNA-LNP, spike protein, preservative free, 30 mcg/0.3 mL dosage, diluent reconstituted
or	procedure	UMLS:CPT:1036663	Immunization administration by intramuscular injection of severe acute respiratory

			syndrome coronavirus 2 (SARS-CoV-2) (coronavirus disease [COVID-19]) vaccine, mRNA-LNP, spike protein, preservative free, 100 mcg/0.5 mL dosage
or	procedure	UMLS:CPT:0124A	Immunization administration by intramuscular injection of severe acute respiratory syndrome coronavirus 2 (SARS-CoV-2) (coronavirus disease [COVID-19]) vaccine, mRNA-LNP, bivalent spike protein, preservative free, 30 mcg/0.3 mL dosage, tris-sucrose formulation, booster dose
or	procedure	UMLS:CPT:0004A	Immunization administration by intramuscular injection of severe acute respiratory syndrome coronavirus 2 (SARS-CoV-2) (coronavirus disease [COVID-19]) vaccine, mRNA-LNP, spike protein, preservative free, 30 mcg/0.3 mL dosage, diluent reconstituted; booster dose
or	procedure	UMLS:CPT:0003A	Immunization administration by

			intramuscular injection of severe acute respiratory syndrome coronavirus 2 (SARS-CoV-2) (coronavirus disease [COVID-19]) vaccine, mRNA-LNP, spike protein, preservative free, 30 mcg/0.3 mL dosage, diluent reconstituted; third dose
or	procedure	UMLS:CPT:1037166	Immunization administration by intramuscular injection of severe acute respiratory syndrome coronavirus 2 (SARS-CoV-2) (coronavirus disease [COVID-19]) vaccine, mRNA-LNP, spike protein, preservative free, 30 mcg/0.3 mL dosage, tris-sucrose formulation
or	procedure	UMLS:CPT:0054A	Immunization administration by intramuscular injection of severe acute respiratory syndrome coronavirus 2 (SARS-CoV-2) (coronavirus disease [COVID-19]) vaccine, mRNA-LNP, spike protein, preservative free, 30 mcg/0.3 mL dosage, tris-sucrose formulation; booster dose

or	procedure	UMLS:CPT:0064A	Immunization administration by intramuscular injection of severe acute respiratory syndrome coronavirus 2 (SARS-CoV-2) (coronavirus disease [COVID-19]) vaccine, mRNA-LNP, spike protein, preservative free, 50 mcg/0.25 mL dosage, booster dose
or	procedure	UMLS:CPT:90480	Immunization administration by intramuscular injection of severe acute respiratory syndrome coronavirus 2 (SARS-CoV-2) (coronavirus disease [COVID-19]) vaccine, single dose
or	procedure	UMLS:CPT:1037171	Immunization administration by intramuscular injection of severe acute respiratory syndrome coronavirus 2 (SARS-CoV-2) (coronavirus disease [COVID-19]) vaccine, mRNA-LNP, spike protein, preservative free, 10 mcg/0.2 mL dosage, diluent reconstituted, tris-sucrose formulation

or	procedure	UMLS:CPT:0071A	Immunization administration by intramuscular injection of severe acute respiratory syndrome coronavirus 2 (SARS-CoV-2) (coronavirus disease [COVID-19]) vaccine, mRNA-LNP, spike protein, preservative free, 10 mcg/0.2 mL dosage, diluent reconstituted, tris-sucrose formulation; first dose
or	procedure	UMLS:CPT:0072A	Immunization administration by intramuscular injection of severe acute respiratory syndrome coronavirus 2 (SARS-CoV-2) (coronavirus disease [COVID-19]) vaccine, mRNA-LNP, spike protein, preservative free, 10 mcg/0.2 mL dosage, diluent reconstituted, tris-sucrose formulation; second dose
or	medication	NLM:RXNORM:2610319	SARS-CoV-2 (COVID-19) vaccine, mRNA-BNT162b2 0.05 MG/ML / SARS-CoV-2 (COVID-19) vaccine, mRNA-BNT162b2 OMICRON (BA.4/BA.5) 0.05 MG/ML Injectable Suspension

or	procedure	UMLS:CPT:91313	Severe acute respiratory syndrome coronavirus 2 (SARS-CoV-2) (coronavirus disease [COVID-19]) vaccine, mRNA-LNP, spike protein, bivalent, preservative free, 50 mcg/0.5 mL dosage, for intramuscular use
or	procedure	UMLS:CPT:0134A	Immunization administration by intramuscular injection of severe acute respiratory syndrome coronavirus 2 (SARS-CoV-2) (coronavirus disease [COVID-19]) vaccine, mRNA-LNP, spike protein, bivalent, preservative free, 50 mcg/0.5 mL dosage, booster dose
or	procedure	UMLS:CPT:1037175	Immunization administration by intramuscular injection of severe acute respiratory syndrome coronavirus 2 (SARS-CoV-2) (coronavirus disease [COVID-19]) vaccine, DNA, spike protein, adenovirus type 26 (Ad26) vector, preservative free, 5x1010 viral particles/0.5

			mL dosage
or	medication	NLM:RXNORM:2610347	0.3 ML SARS-CoV-2 (COVID-19) vaccine, mRNA-BNT162b2 0.05 MG/ML / SARS-CoV-2 (COVID-19) vaccine, mRNA-BNT162b2 OMICRON (BA.4/BA.5) -1 MG/ML Injection
or	procedure	UMLS:CPT:1037228	Immunization administration by intramuscular injection of severe acute respiratory syndrome coronavirus 2 (SARS-CoV-2) (coronavirus disease [COVID-19]) vaccine, mRNA-LNP, spike protein, preservative free, 3 mcg/0.2 mL dosage, diluent reconstituted, tris-sucrose formulation
or	procedure	UMLS:CPT:0013A	Immunization administration by intramuscular injection of severe acute respiratory syndrome coronavirus 2 (SARS-CoV-2) (coronavirus disease [COVID-19]) vaccine, mRNA-LNP, spike protein, preservative free, 100 mcg/0.5 mL dosage; third dose

or	procedure	UMLS:CPT:0081A	Immunization administration by intramuscular injection of severe acute respiratory syndrome coronavirus 2 (SARS-CoV-2) (coronavirus disease [COVID-19]) vaccine, mRNA-LNP, spike protein, preservative free, 3 mcg/0.2 mL dosage, diluent reconstituted, tris-sucrose formulation; first dose
or	procedure	UMLS:CPT:0082A	Immunization administration by intramuscular injection of severe acute respiratory syndrome coronavirus 2 (SARS-CoV-2) (coronavirus disease [COVID-19]) vaccine, mRNA-LNP, spike protein, preservative free, 3 mcg/0.2 mL dosage, diluent reconstituted, tris-sucrose formulation; second dose
or	medication	NLM:RXNORM:2610328	SARS-CoV-2 (COVID-19) vaccine, mRNA-1273 0.05 MG/ML / SARS-CoV-2 (COVID-19) vaccine, mRNA-1273 OMICRON (BA.4/BA.5) 0.05 MG/ML Injectable Suspension

or	procedure	UMLS:CPT:0154A	Immunization administration by intramuscular injection of severe acute respiratory syndrome coronavirus 2 (SARS-CoV-2) (coronavirus disease [COVID-19]) vaccine, mRNA-LNP, bivalent spike protein, preservative free, 10 mcg/0.2 mL dosage, diluent reconstituted, tris-sucrose formulation, booster dose
or	procedure	UMLS:CPT:0053A	Immunization administration by intramuscular injection of severe acute respiratory syndrome coronavirus 2 (SARS-CoV-2) (coronavirus disease [COVID-19]) vaccine, mRNA-LNP, spike protein, preservative free, 30 mcg/0.3 mL dosage, tris-sucrose formulation; third dose
or	procedure	UMLS:CPT:1037332	Immunization administration by intramuscular injection of severe acute respiratory syndrome coronavirus 2 (SARS-CoV-2) (coronavirus disease [COVID-19])

			vaccine, mRNA-LNP, spike protein, preservative free, 25 mcg/0.25 mL dosage
or	procedure	UMLS:CPT:0052A	Immunization administration by intramuscular injection of severe acute respiratory syndrome coronavirus 2 (SARS-CoV-2) (coronavirus disease [COVID-19]) vaccine, mRNA-LNP, spike protein, preservative free, 30 mcg/0.3 mL dosage, tris-sucrose formulation; second dose
or	procedure	UMLS:CPT:0111A	Immunization administration by intramuscular injection of severe acute respiratory syndrome coronavirus 2 (SARS-CoV-2) (coronavirus disease [COVID-19]) vaccine, mRNA-LNP, spike protein, preservative free, 25 mcg/0.25 mL dosage; first dose
or	procedure	UMLS:CPT:0051A	Immunization administration by intramuscular injection of severe acute respiratory syndrome coronavirus 2 (SARS-CoV-2) (coronavirus

			disease [COVID-19]) vaccine, mRNA-LNP, spike protein, preservative free, 30 mcg/0.3 mL dosage, tris-sucrose formulation; first dose
or	procedure	UMLS:CPT:91311	Severe acute respiratory syndrome coronavirus 2 (SARS-CoV-2) (coronavirus disease [COVID-19]) vaccine, mRNA-LNP, spike protein, preservative free, 25 mcg/0.25 mL dosage, for intramuscular use
or	procedure	UMLS:CPT:0074A	Immunization administration by intramuscular injection of severe acute respiratory syndrome coronavirus 2 (SARS-CoV-2) (coronavirus disease [COVID-19]) vaccine, mRNA-LNP, spike protein, preservative free, 10 mcg/0.2 mL dosage, diluent reconstituted, tris-sucrose formulation; booster dose
or	procedure	UMLS:CPT:0112A	Immunization administration by intramuscular injection of severe acute respiratory syndrome coronavirus 2 (SARS-CoV-2) (coronavirus

			disease [COVID-19]) vaccine, mRNA-LNP, spike protein, preservative free, 25 mcg/0.25 mL dosage; second dose
or	procedure	UMLS:CPT:0083A	Immunization administration by intramuscular injection of severe acute respiratory syndrome coronavirus 2 (SARS-CoV-2) (coronavirus disease [COVID-19]) vaccine, mRNA-LNP, spike protein, preservative free, 3 mcg/0.2 mL dosage, diluent reconstituted, tris-sucrose formulation; third dose
or	procedure	UMLS:CPT:0073A	Immunization administration by intramuscular injection of severe acute respiratory syndrome coronavirus 2 (SARS-CoV-2) (coronavirus disease [COVID-19]) vaccine, mRNA-LNP, spike protein, preservative free, 10 mcg/0.2 mL dosage, diluent reconstituted, tris-sucrose formulation; third dose
or	procedure	UMLS:CPT:0173A	Immunization administration by intramuscular injection of

			severe acute respiratory syndrome coronavirus 2 (SARS-CoV-2) (coronavirus disease [COVID-19]) vaccine, mRNA-LNP, bivalent spike protein, preservative free, 3 mcg/0.2 mL dosage, diluent reconstituted, tris-sucrose formulation, third dose
or	procedure	UMLS:CPT:0164A	Immunization administration by intramuscular injection of severe acute respiratory syndrome coronavirus 2 (SARS-CoV-2) (coronavirus disease [COVID-19]) vaccine, mRNA-LNP, spike protein, bivalent, preservative free, 10 mcg/0.2 mL dosage, booster dose
or	procedure	UMLS:CPT:1037838	Immunization administration by intramuscular injection of severe acute respiratory syndrome coronavirus 2 (SARS-CoV-2) (coronavirus disease [COVID-19]) vaccine, mRNA-LNP, spike protein, preservative free, 50 mcg/0.5 mL dosage

or	procedure	UMLS:CPT:0094A	Immunization administration by intramuscular injection of severe acute respiratory syndrome coronavirus 2 (SARS-CoV-2) (coronavirus disease [COVID-19]) vaccine, mRNA-LNP, spike protein, preservative free, 50 mcg/0.5 mL dosage; booster dose, when administered to individuals 18 years and over
or	procedure	UMLS:CPT:0034A	Immunization administration by intramuscular injection of severe acute respiratory syndrome coronavirus 2 (SARS-CoV-2) (coronavirus disease [COVID-19]) vaccine, DNA, spike protein, adenovirus type 26 (Ad26) vector, preservative free, 5x1010 viral particles/0.5 mL dosage; booster dose
or	procedure	UMLS:CPT:0144A	Immunization administration by intramuscular injection of severe acute respiratory syndrome coronavirus 2 (SARS-CoV-2) (coronavirus disease [COVID-19])

			vaccine, mRNA-LNP, spike protein, bivalent, preservative free, 25 mcg/0.25 mL dosage, booster dose
or	procedure	UMLS:CPT:0091A	Immunization administration by intramuscular injection of severe acute respiratory syndrome coronavirus 2 (SARS-CoV-2) (coronavirus disease [COVID-19]) vaccine, mRNA-LNP, spike protein, preservative free, 50 mcg/0.5 mL dosage; first dose, when administered to individuals 6 through 11 years
or	procedure	UMLS:CPT:0174A	Immunization administration by intramuscular injection of severe acute respiratory syndrome coronavirus 2 (SARS-CoV-2) (coronavirus disease [COVID-19]) vaccine, mRNA-LNP, bivalent spike protein, preservative free, 3 mcg/0.2 mL dosage, diluent reconstituted, tris-sucrose formulation, booster

or	procedure	UMLS:CPT:0092A	Immunization administration by intramuscular injection of severe acute respiratory syndrome coronavirus 2 (SARS-CoV-2) (coronavirus disease [COVID-19]) vaccine, mRNA-LNP, spike protein, preservative free, 50 mcg/0.5 mL dosage; second dose, when administered to individuals 6 through 11 years
or	procedure	UMLS:CPT:1036682	Immunization administration by intramuscular injection of severe acute respiratory syndrome coronavirus 2 (SARS-CoV-2) (coronavirus disease [COVID-19]) vaccine, recombinant spike protein nanoparticle, saponin-based adjuvant, preservative free, 5 mcg/0.5 mL dosage
or	procedure	UMLS:CPT:0041A	Immunization administration by intramuscular injection of severe acute respiratory syndrome coronavirus 2 (SARS-CoV-2) (coronavirus disease [COVID-19])

			vaccine, recombinant spike protein nanoparticle, saponin-based adjuvant, preservative free, 5 mcg/0.5 mL dosage; first dose
or	procedure	UMLS:CPT:0113A	Immunization administration by intramuscular injection of severe acute respiratory syndrome coronavirus 2 (SARS-CoV-2) (coronavirus disease [COVID-19]) vaccine, mRNA-LNP, spike protein, preservative free, 25 mcg/0.25 mL dosage; third dose
or	procedure	UMLS:CPT:0042A	Immunization administration by intramuscular injection of severe acute respiratory syndrome coronavirus 2 (SARS-CoV-2) (coronavirus disease [COVID-19]) vaccine, recombinant spike protein nanoparticle, saponin-based adjuvant, preservative free, 5 mcg/0.5 mL dosage; second dose
or	procedure	UMLS:CPT:0093A	Immunization administration by intramuscular injection of severe acute respiratory

			syndrome coronavirus 2 (SARS-CoV-2) (coronavirus disease [COVID-19]) vaccine, mRNA-LNP, spike protein, preservative free, 50 mcg/0.5 mL dosage; third dose, when administered to individuals 6 through 11 years
or	procedure	UMLS:CPT:1036666	Immunization administration by intramuscular injection of severe acute respiratory syndrome coronavirus 2 (SARS-CoV-2) (coronavirus disease [COVID-19]) vaccine, DNA, spike protein, chimpanzee adenovirus Oxford 1 (ChAdOx1) vector, preservative free, 5x1010 viral particles/0.5 mL dosage
or	procedure	UMLS:CPT:0044A	Immunization administration by intramuscular injection of severe acute respiratory syndrome coronavirus 2 (SARS-CoV-2) (coronavirus disease [COVID-19]) vaccine, recombinant spike protein nanoparticle, saponin-based adjuvant,

				preservative free, 5 mcg/0.5mL dosage; booster
date constraint		The terms in this group occurred between Dec 1, 2019 and Dec 31, 2023		
**Group 2**				
**Group 2A COVID-19**				
must have	any of	diagnosis	UMLS:ICD10CM:U07.1	COVID-19
		diagnosis	UMLS:ICD10CM:U07.2	COVID-19, virus not identified (WHO)
		diagnosis	UMLS:ICD10CM:J12.82	Pneumonia due to coronavirus disease 2019
		laboratory	UMLS:LNC:94500-6	SARS-CoV-2 (COVID-19) RNA [Presence] in Respiratory specimen by NAA with probe detection (labResult: Positive)
		laboratory	UMLS:LNC:94309-2	SARS-CoV-2 (COVID-19) RNA [Presence] in Specimen by NAA with probe detection (labResult: Positive)
		laboratory	UMLS:LNC:94565-9	SARS-CoV-2 (COVID-19) RNA [Presence] in Nasopharynx by NAA with non-probe detection (labResult: Positive)
		laboratory	UMLS:LNC:94759-8	SARS-CoV-2 (COVID-19) RNA [Presence] in Nasopharynx by NAA with

				probe detection (labResult: Positive)
		laboratory	UMLS:LNC:95608-6	SARS-CoV-2 (COVID-19) RNA [Presence] in Respiratory specimen by NAA with non-probe detection (labResult: Positive)
		laboratory	UMLS:LNC:94845-5	SARS-CoV-2 (COVID-19) RNA [Presence] in Saliva (oral fluid) by NAA with probe detection (labResult: Positive)
		laboratory	UMLS:LNC:95406-5	SARS-CoV-2 (COVID-19) RNA [Presence] in Nose by NAA with probe detection (labResult: Positive)
	and	visit	TNX:Visit	Visit (Data Source: TriNetX)
date constraint		The terms in this group occurred at any time		
event relationship		Any instance of thyroid cancer history occurred at least 1 day before the first instance of COVID-19		
**Group 2B thyroid cancer history**				
cannot have		diagnosis	UMLS:ICD10CM:C73	Malignant neoplasm of thyroid gland
	or	diagnosis	UMLS:ICD10CM:Z85.850	Personal history of malignant neoplasm of thyroid

Query Criteria for Cohort post-COVID, normal thyroid function

This query was run on the network Global Collaborative Network with 146 HCO(s) queried and 146 HCO(s) responded. A total of 120 provider(s) responded with patients. The final cohort included 2,102,969 patients who matched the query criteria listed in the table below.

**Ungrouped terms**				
must have		demographics	Age	Age (at least 18 years (most recent occurrence))
	and any of	demographics	UMLS:HL7V3.0:Gender:M	Male
		demographics	UMLS:HL7V3.0:Gender:F	Female
**Group 1**				
**Group 1A COVID-19, no vaccination**				
must have	any of	diagnosis	UMLS:ICD10CM:U07.1	COVID-19
		diagnosis	UMLS:ICD10CM:U07.2	COVID-19, virus not identified (WHO)
		diagnosis	UMLS:ICD10CM:J12.82	Pneumonia due to coronavirus disease 2019
		laboratory	UMLS:LNC:94500-6	SARS-CoV-2 (COVID-19) RNA [Presence] in Respiratory specimen by NAA with probe detection (labResult: Positive)

	laboratory	UMLS:LNC:94309-2	SARS-CoV-2 (COVID-19) RNA [Presence] in Specimen by NAA with probe detection (labResult: Positive)
	laboratory	UMLS:LNC:94565-9	SARS-CoV-2 (COVID-19) RNA [Presence] in Nasopharynx by NAA with non-probe detection (labResult: Positive)
	laboratory	UMLS:LNC:94759-8	SARS-CoV-2 (COVID-19) RNA [Presence] in Nasopharynx by NAA with probe detection (labResult: Positive)
	laboratory	UMLS:LNC:95608-6	SARS-CoV-2 (COVID-19) RNA [Presence] in Respiratory specimen by NAA with non-probe detection (labResult: Positive)
	laboratory	UMLS:LNC:94845-5	SARS-CoV-2 (COVID-19) RNA [Presence] in Saliva (oral fluid) by NAA with probe detection (labResult: Positive)
	laboratory	UMLS:LNC:95406-5	SARS-CoV-2 (COVID-19) RNA [Presence] in Nose by NAA with probe detection (labResult: Positive)
cannot	medicatio	NLM:CVX:208	COVID-19, mRNA, LNP-S,

have		n		PF, 30 mcg/0.3 mL dose
	or	medication	NLM:CVX:207	COVID-19, mRNA, LNP-S, PF, 100 mcg/0.5mL dose or 50 mcg/0.25mL dose
	or	medication	NLM:CVX:212	COVID-19 vaccine, vector-nr, rS-Ad26, PF, 0.5 mL
	or	medication	NLM:RXNORM:OMOP5042939	COVID-19 vaccine
	or	medication	NLM:CVX:300	COVID-19, mRNA, LNP-S, bivalent, PF, 30 mcg/0.3 mL dose
	or	medication	NLM:CVX:217	COVID-19, mRNA, LNP-S, PF, 30 mcg/0.3 mL dose, tris-sucrose
	or	medication	NLM:CVX:229	COVID-19, mRNA, LNP-S, bivalent, PF, 50 mcg/0.5 mL or 25mcg/0.25 mL dose
	or	medication	NLM:CVX:218	COVID-19, mRNA, LNP-S, PF, 10 mcg/0.2 mL dose, tris-sucrose
	or	medication	NLM:CVX:520	COVID-19 mRNA, bivalent, original/Omicron BA.1, Non-US Vaccine Product, Pfizer-BioNTech
	or	medication	NLM:CVX:519	COVID-19 mRNA, bivalent, original/Omicron BA.1, Non-US Vaccine (Spikevax Bivalent), Moderna

or	medicatio n	NLM:CVX:301	COVID-19, mRNA, LNP-S, bivalent, PF, 10 mcg/0.2 mL dose
or	medicatio n	NLM:CVX:219	COVID-19, mRNA, LNP-S, PF, 3 mcg/0.2 mL dose, tris-sucrose
or	medicatio n	NLM:CVX:228	COVID-19, mRNA, LNP-S, PF, pediatric 25 mcg/0.25 mL dose
or	medicatio n	NLM:CVX:230	COVID-19, mRNA, LNP-S, bivalent booster, PF, 10 mcg/0.2 mL
or	medicatio n	NLM:CVX:221	COVID-19, mRNA, LNP-S, PF, 50 mcg/0.5 mL dose
or	medicatio n	NLM:CVX:210	COVID-19 vaccine, vector-nr, rS-ChAdOx1, PF, 0.5 mL
or	medicatio n	NLM:CVX:302	COVID-19, mRNA, LNP-S, bivalent, PF, 3 mcg/0.2 mL dose
or	medicatio n	NLM:CVX:511	COVID-19 IV Non-US Vaccine (CoronaVac, Sinovac)
or	medicatio n	NLM:RXNORM:246 8231	SARS-CoV-2 (COVID-19) vaccine, mRNA spike protein
or	procedure	UMLS:CPT:91300	Severe acute respiratory syndrome coronavirus 2 (SARS-CoV-2) (coronavirus disease [COVID-19])

			vaccine, mRNA-LNP, spike protein, preservative free, 30 mcg/0.3 mL dosage, diluent reconstituted, for intramuscular use
or	procedure	UMLS:CPT:0001A	Immunization administration by intramuscular injection of severe acute respiratory syndrome coronavirus 2 (SARS-CoV-2) (coronavirus disease [COVID-19]) vaccine, mRNA-LNP, spike protein, preservative free, 30 mcg/0.3 mL dosage, diluent reconstituted; first dose
or	procedure	UMLS:CPT:0002A	Immunization administration by intramuscular injection of severe acute respiratory syndrome coronavirus 2 (SARS-CoV-2) (coronavirus disease [COVID-19]) vaccine, mRNA-LNP, spike protein, preservative free, 30 mcg/0.3 mL dosage, diluent reconstituted; second dose
or	procedure	UMLS:CPT:91301	Severe acute respiratory syndrome coronavirus 2 (SARS-CoV-2) (coronavirus disease [COVID-19]) vaccine, mRNA-LNP, spike

			protein, preservative free, 100 mcg/0.5 mL dosage, for intramuscular use
or	procedure	UMLS:CPT:0011A	Immunization administration by intramuscular injection of severe acute respiratory syndrome coronavirus 2 (SARS-CoV-2) (coronavirus disease [COVID-19]) vaccine, mRNA-LNP, spike protein, preservative free, 100 mcg/0.5 mL dosage; first dose
or	procedure	UMLS:CPT:0012A	Immunization administration by intramuscular injection of severe acute respiratory syndrome coronavirus 2 (SARS-CoV-2) (coronavirus disease [COVID-19]) vaccine, mRNA-LNP, spike protein, preservative free, 100 mcg/0.5 mL dosage; second dose
or	procedure	UMLS:SNOMED:840534001	Administration of SARS-CoV-2 antigen vaccine
or	medication	NLM:CVX:213	SARS-CoV-2 (COVID-19) Vaccine
or	procedure	UMLS:CPT:1036660	Immunization administration by

			intramuscular injection of severe acute respiratory syndrome coronavirus 2 (SARS-CoV-2) (coronavirus disease [COVID-19]) vaccine, mRNA-LNP, spike protein, preservative free, 30 mcg/0.3 mL dosage, diluent reconstituted
or	procedure	UMLS:CPT:1036663	Immunization administration by intramuscular injection of severe acute respiratory syndrome coronavirus 2 (SARS-CoV-2) (coronavirus disease [COVID-19]) vaccine, mRNA-LNP, spike protein, preservative free, 100 mcg/0.5 mL dosage
or	procedure	UMLS:CPT:0124A	Immunization administration by intramuscular injection of severe acute respiratory syndrome coronavirus 2 (SARS-CoV-2) (coronavirus disease [COVID-19]) vaccine, mRNA-LNP, bivalent spike protein, preservative free, 30 mcg/0.3 mL dosage, tris-sucrose formulation, booster dose

or	procedure	UMLS:CPT:0004A	Immunization administration by intramuscular injection of severe acute respiratory syndrome coronavirus 2 (SARS-CoV-2) (coronavirus disease [COVID-19]) vaccine, mRNA-LNP, spike protein, preservative free, 30 mcg/0.3 mL dosage, diluent reconstituted; booster dose
or	procedure	UMLS:CPT:0003A	Immunization administration by intramuscular injection of severe acute respiratory syndrome coronavirus 2 (SARS-CoV-2) (coronavirus disease [COVID-19]) vaccine, mRNA-LNP, spike protein, preservative free, 30 mcg/0.3 mL dosage, diluent reconstituted; third dose
or	procedure	UMLS:CPT:1037166	Immunization administration by intramuscular injection of severe acute respiratory syndrome coronavirus 2 (SARS-CoV-2) (coronavirus disease [COVID-19]) vaccine, mRNA-LNP, spike protein, preservative free, 30 mcg/0.3 mL dosage, tris-

			sucrose formulation
or	procedure	UMLS:CPT:0054A	Immunization administration by intramuscular injection of severe acute respiratory syndrome coronavirus 2 (SARS-CoV-2) (coronavirus disease [COVID-19]) vaccine, mRNA-LNP, spike protein, preservative free, 30 mcg/0.3 mL dosage, tris-sucrose formulation; booster dose
or	procedure	UMLS:CPT:0064A	Immunization administration by intramuscular injection of severe acute respiratory syndrome coronavirus 2 (SARS-CoV-2) (coronavirus disease [COVID-19]) vaccine, mRNA-LNP, spike protein, preservative free, 50 mcg/0.25 mL dosage, booster dose
or	procedure	UMLS:CPT:90480	Immunization administration by intramuscular injection of severe acute respiratory syndrome coronavirus 2 (SARS-CoV-2) (coronavirus disease [COVID-19])

			vaccine, single dose
or	procedure	UMLS:CPT:1037171	Immunization administration by intramuscular injection of severe acute respiratory syndrome coronavirus 2 (SARS-CoV-2) (coronavirus disease [COVID-19]) vaccine, mRNA-LNP, spike protein, preservative free, 10 mcg/0.2 mL dosage, diluent reconstituted, tris-sucrose formulation
or	procedure	UMLS:CPT:0071A	Immunization administration by intramuscular injection of severe acute respiratory syndrome coronavirus 2 (SARS-CoV-2) (coronavirus disease [COVID-19]) vaccine, mRNA-LNP, spike protein, preservative free, 10 mcg/0.2 mL dosage, diluent reconstituted, tris-sucrose formulation; first dose
or	procedure	UMLS:CPT:0072A	Immunization administration by intramuscular injection of severe acute respiratory syndrome coronavirus 2 (SARS-CoV-2) (coronavirus disease [COVID-19])

			vaccine, mRNA-LNP, spike protein, preservative free, 10 mcg/0.2 mL dosage, diluent reconstituted, tris-sucrose formulation; second dose
or	medication	NLM:RXNORM:2610319	SARS-CoV-2 (COVID-19) vaccine, mRNA-BNT162b2 0.05 MG/ML / SARS-CoV-2 (COVID-19) vaccine, mRNA-BNT162b2 OMICRON (BA.4/BA.5) 0.05 MG/ML Injectable Suspension
or	procedure	UMLS:CPT:91313	Severe acute respiratory syndrome coronavirus 2 (SARS-CoV-2) (coronavirus disease [COVID-19]) vaccine, mRNA-LNP, spike protein, bivalent, preservative free, 50 mcg/0.5 mL dosage, for intramuscular use
or	procedure	UMLS:CPT:0134A	Immunization administration by intramuscular injection of severe acute respiratory syndrome coronavirus 2 (SARS-CoV-2) (coronavirus disease [COVID-19]) vaccine, mRNA-LNP, spike protein, bivalent, preservative free, 50 mcg/0.5 mL dosage, booster

			dose
or	procedure	UMLS:CPT:1037175	Immunization administration by intramuscular injection of severe acute respiratory syndrome coronavirus 2 (SARS-CoV-2) (coronavirus disease [COVID-19]) vaccine, DNA, spike protein, adenovirus type 26 (Ad26) vector, preservative free, 5x1010 viral particles/0.5 mL dosage
or	medication	NLM:RXNORM:2610347	0.3 ML SARS-CoV-2 (COVID-19) vaccine, mRNA-BNT162b2 0.05 MG/ML / SARS-CoV-2 (COVID-19) vaccine, mRNA-BNT162b2 OMICRON (BA.4/BA.5) -1 MG/ML Injection
or	procedure	UMLS:CPT:1037228	Immunization administration by intramuscular injection of severe acute respiratory syndrome coronavirus 2 (SARS-CoV-2) (coronavirus disease [COVID-19]) vaccine, mRNA-LNP, spike protein, preservative free, 3 mcg/0.2 mL dosage, diluent reconstituted, tris-sucrose

			formulation
or	procedure	UMLS:CPT:0013A	Immunization administration by intramuscular injection of severe acute respiratory syndrome coronavirus 2 (SARS-CoV-2) (coronavirus disease [COVID-19]) vaccine, mRNA-LNP, spike protein, preservative free, 100 mcg/0.5 mL dosage; third dose
or	procedure	UMLS:CPT:0081A	Immunization administration by intramuscular injection of severe acute respiratory syndrome coronavirus 2 (SARS-CoV-2) (coronavirus disease [COVID-19]) vaccine, mRNA-LNP, spike protein, preservative free, 3 mcg/0.2 mL dosage, diluent reconstituted, tris-sucrose formulation; first dose
or	procedure	UMLS:CPT:0082A	Immunization administration by intramuscular injection of severe acute respiratory syndrome coronavirus 2 (SARS-CoV-2) (coronavirus disease [COVID-19]) vaccine, mRNA-LNP, spike

			protein, preservative free, 3 mcg/0.2 mL dosage, diluent reconstituted, tris-sucrose formulation; second dose
or	medication	NLM:RXNORM:2610328	SARS-CoV-2 (COVID-19) vaccine, mRNA-1273 0.05 MG/ML / SARS-CoV-2 (COVID-19) vaccine, mRNA-1273 OMICRON (BA.4/BA.5) 0.05 MG/ML Injectable Suspension
or	procedure	UMLS:CPT:0154A	Immunization administration by intramuscular injection of severe acute respiratory syndrome coronavirus 2 (SARS-CoV-2) (coronavirus disease [COVID-19]) vaccine, mRNA-LNP, bivalent spike protein, preservative free, 10 mcg/0.2 mL dosage, diluent reconstituted, tris-sucrose formulation, booster dose
or	procedure	UMLS:CPT:0053A	Immunization administration by intramuscular injection of severe acute respiratory syndrome coronavirus 2 (SARS-CoV-2) (coronavirus disease [COVID-19]) vaccine, mRNA-LNP, spike

			protein, preservative free, 30 mcg/0.3 mL dosage, tris-sucrose formulation; third dose
or	procedure	UMLS:CPT:1037332	Immunization administration by intramuscular injection of severe acute respiratory syndrome coronavirus 2 (SARS-CoV-2) (coronavirus disease [COVID-19]) vaccine, mRNA-LNP, spike protein, preservative free, 25 mcg/0.25 mL dosage
or	procedure	UMLS:CPT:0052A	Immunization administration by intramuscular injection of severe acute respiratory syndrome coronavirus 2 (SARS-CoV-2) (coronavirus disease [COVID-19]) vaccine, mRNA-LNP, spike protein, preservative free, 30 mcg/0.3 mL dosage, tris-sucrose formulation; second dose
or	procedure	UMLS:CPT:0111A	Immunization administration by intramuscular injection of severe acute respiratory syndrome coronavirus 2 (SARS-CoV-2) (coronavirus

			disease [COVID-19]) vaccine, mRNA-LNP, spike protein, preservative free, 25 mcg/0.25 mL dosage; first dose
or	procedure	UMLS:CPT:0051A	Immunization administration by intramuscular injection of severe acute respiratory syndrome coronavirus 2 (SARS-CoV-2) (coronavirus disease [COVID-19]) vaccine, mRNA-LNP, spike protein, preservative free, 30 mcg/0.3 mL dosage, tris-sucrose formulation; first dose
or	procedure	UMLS:CPT:91311	Severe acute respiratory syndrome coronavirus 2 (SARS-CoV-2) (coronavirus disease [COVID-19]) vaccine, mRNA-LNP, spike protein, preservative free, 25 mcg/0.25 mL dosage, for intramuscular use
or	procedure	UMLS:CPT:0074A	Immunization administration by intramuscular injection of severe acute respiratory syndrome coronavirus 2 (SARS-CoV-2) (coronavirus disease [COVID-19])

			vaccine, mRNA-LNP, spike protein, preservative free, 10 mcg/0.2 mL dosage, diluent reconstituted, tris-sucrose formulation; booster dose
or	procedure	UMLS:CPT:0112A	Immunization administration by intramuscular injection of severe acute respiratory syndrome coronavirus 2 (SARS-CoV-2) (coronavirus disease [COVID-19]) vaccine, mRNA-LNP, spike protein, preservative free, 25 mcg/0.25 mL dosage; second dose
or	procedure	UMLS:CPT:0083A	Immunization administration by intramuscular injection of severe acute respiratory syndrome coronavirus 2 (SARS-CoV-2) (coronavirus disease [COVID-19]) vaccine, mRNA-LNP, spike protein, preservative free, 3 mcg/0.2 mL dosage, diluent reconstituted, tris-sucrose formulation; third dose
or	procedure	UMLS:CPT:0073A	Immunization administration by intramuscular injection of severe acute respiratory

			syndrome coronavirus 2 (SARS-CoV-2) (coronavirus disease [COVID-19]) vaccine, mRNA-LNP, spike protein, preservative free, 10 mcg/0.2 mL dosage, diluent reconstituted, tris-sucrose formulation; third dose
or	procedure	UMLS:CPT:0173A	Immunization administration by intramuscular injection of severe acute respiratory syndrome coronavirus 2 (SARS-CoV-2) (coronavirus disease [COVID-19]) vaccine, mRNA-LNP, bivalent spike protein, preservative free, 3 mcg/0.2 mL dosage, diluent reconstituted, tris-sucrose formulation, third dose
or	procedure	UMLS:CPT:0164A	Immunization administration by intramuscular injection of severe acute respiratory syndrome coronavirus 2 (SARS-CoV-2) (coronavirus disease [COVID-19]) vaccine, mRNA-LNP, spike protein, bivalent, preservative free, 10 mcg/0.2 mL dosage, booster

			dose
or	procedure	UMLS:CPT:1037838	Immunization administration by intramuscular injection of severe acute respiratory syndrome coronavirus 2 (SARS-CoV-2) (coronavirus disease [COVID-19]) vaccine, mRNA-LNP, spike protein, preservative free, 50 mcg/0.5 mL dosage
or	procedure	UMLS:CPT:0094A	Immunization administration by intramuscular injection of severe acute respiratory syndrome coronavirus 2 (SARS-CoV-2) (coronavirus disease [COVID-19]) vaccine, mRNA-LNP, spike protein, preservative free, 50 mcg/0.5 mL dosage; booster dose, when administered to individuals 18 years and over
or	procedure	UMLS:CPT:0034A	Immunization administration by intramuscular injection of severe acute respiratory syndrome coronavirus 2 (SARS-CoV-2) (coronavirus disease [COVID-19]) vaccine, DNA, spike protein,

			adenovirus type 26 (Ad26) vector, preservative free, 5x1010 viral particles/0.5 mL dosage; booster dose
or	procedure	UMLS:CPT:0144A	Immunization administration by intramuscular injection of severe acute respiratory syndrome coronavirus 2 (SARS-CoV-2) (coronavirus disease [COVID-19]) vaccine, mRNA-LNP, spike protein, bivalent, preservative free, 25 mcg/0.25 mL dosage, booster dose
or	procedure	UMLS:CPT:0091A	Immunization administration by intramuscular injection of severe acute respiratory syndrome coronavirus 2 (SARS-CoV-2) (coronavirus disease [COVID-19]) vaccine, mRNA-LNP, spike protein, preservative free, 50 mcg/0.5 mL dosage; first dose, when administered to individuals 6 through 11 years
or	procedure	UMLS:CPT:0174A	Immunization administration by intramuscular injection of

			severe acute respiratory syndrome coronavirus 2 (SARS-CoV-2) (coronavirus disease [COVID-19]) vaccine, mRNA-LNP, bivalent spike protein, preservative free, 3 mcg/0.2 mL dosage, diluent reconstituted, tris-sucrose formulation, booster
or	procedure	UMLS:CPT:0092A	Immunization administration by intramuscular injection of severe acute respiratory syndrome coronavirus 2 (SARS-CoV-2) (coronavirus disease [COVID-19]) vaccine, mRNA-LNP, spike protein, preservative free, 50 mcg/0.5 mL dosage; second dose, when administered to individuals 6 through 11 years
or	procedure	UMLS:CPT:1036682	Immunization administration by intramuscular injection of severe acute respiratory syndrome coronavirus 2 (SARS-CoV-2) (coronavirus disease [COVID-19]) vaccine, recombinant spike protein nanoparticle,

			saponin-based adjuvant, preservative free, 5 mcg/0.5 mL dosage
or	procedure	UMLS:CPT:0041A	Immunization administration by intramuscular injection of severe acute respiratory syndrome coronavirus 2 (SARS-CoV-2) (coronavirus disease [COVID-19]) vaccine, recombinant spike protein nanoparticle, saponin-based adjuvant, preservative free, 5 mcg/0.5 mL dosage; first dose
or	procedure	UMLS:CPT:0113A	Immunization administration by intramuscular injection of severe acute respiratory syndrome coronavirus 2 (SARS-CoV-2) (coronavirus disease [COVID-19]) vaccine, mRNA-LNP, spike protein, preservative free, 25 mcg/0.25 mL dosage; third dose
or	procedure	UMLS:CPT:0042A	Immunization administration by intramuscular injection of severe acute respiratory syndrome coronavirus 2 (SARS-CoV-2) (coronavirus

			disease [COVID-19]) vaccine, recombinant spike protein nanoparticle, saponin-based adjuvant, preservative free, 5 mcg/0.5 mL dosage; second dose
or	procedure	UMLS:CPT:0093A	Immunization administration by intramuscular injection of severe acute respiratory syndrome coronavirus 2 (SARS-CoV-2) (coronavirus disease [COVID-19]) vaccine, mRNA-LNP, spike protein, preservative free, 50 mcg/0.5 mL dosage; third dose, when administered to individuals 6 through 11 years
or	procedure	UMLS:CPT:1036666	Immunization administration by intramuscular injection of severe acute respiratory syndrome coronavirus 2 (SARS-CoV-2) (coronavirus disease [COVID-19]) vaccine, DNA, spike protein, chimpanzee adenovirus Oxford 1 (ChAdOx1) vector, preservative free, 5x1010 viral particles/0.5 mL dosage

	or	procedure	UMLS:CPT:0044A	Immunization administration by intramuscular injection of severe acute respiratory syndrome coronavirus 2 (SARS-CoV-2) (coronavirus disease [COVID-19]) vaccine, recombinant spike protein nanoparticle, saponin-based adjuvant, preservative free, 5 mcg/0.5mL dosage; booster
date constraint	The terms in this group occurred between Dec 1, 2019 and Dec 31, 2023			
event relationship	Any instance of no hyperthyroidism or hypothyroidism occurred at least 1 day after any instance of COVID-19, no vaccination			
**Group 1B no hyperthyroidism or hypothyroidism**				
cannot have		diagnosis	UMLS:ICD10CM:E03	Other hypothyroidism
	or	diagnosis	UMLS:ICD10CM:E03.9	Hypothyroidism, unspecified
	or	diagnosis	UMLS:ICD10CM:E06	Thyroiditis
	or	diagnosis	UMLS:ICD10CM:E03.3	Postinfectious hypothyroidism
	or	diagnosis	UMLS:ICD10CM:E03.8	Other specified hypothyroidism
	or	diagnosis	UMLS:ICD10CM:E05	Thyrotoxicosis [hyperthyroidism]

	or	diagnosis	UMLS:ICD10CM:E06	Thyroiditis
	or	diagnosis	UMLS:ICD10CM:E05.80	Other thyrotoxicosis without thyrotoxic crisis or storm
**Group 2**				
**Group 2A COVID-19**				
must have	any of	diagnosis	UMLS:ICD10CM:U07.1	COVID-19
		diagnosis	UMLS:ICD10CM:U07.2	COVID-19, virus not identified (WHO)
		diagnosis	UMLS:ICD10CM:J12.82	Pneumonia due to coronavirus disease 2019
		laboratory	UMLS:LNC:94500-6	SARS-CoV-2 (COVID-19) RNA [Presence] in Respiratory specimen by NAA with probe detection (labResult: Positive)
		laboratory	UMLS:LNC:94309-2	SARS-CoV-2 (COVID-19) RNA [Presence] in Specimen by NAA with probe detection (labResult: Positive)
		laboratory	UMLS:LNC:94565-9	SARS-CoV-2 (COVID-19) RNA [Presence] in Nasopharynx by NAA with non-probe detection (labResult: Positive)
		laboratory	UMLS:LNC:94759-8	SARS-CoV-2 (COVID-19) RNA [Presence] in Nasopharynx by NAA with

				probe detection (labResult: Positive)
		laboratory	UMLS:LNC:95608-6	SARS-CoV-2 (COVID-19) RNA [Presence] in Respiratory specimen by NAA with non-probe detection (labResult: Positive)
		laboratory	UMLS:LNC:94845-5	SARS-CoV-2 (COVID-19) RNA [Presence] in Saliva (oral fluid) by NAA with probe detection (labResult: Positive)
		laboratory	UMLS:LNC:95406-5	SARS-CoV-2 (COVID-19) RNA [Presence] in Nose by NAA with probe detection (labResult: Positive)
	and	visit	TNX:Visit	Visit (Data Source: TriNetX)
date constraint		The terms in this group occurred at any time		
event relationship		Any instance of History of thyroidism abnormality or thyroid ca occurred on or before any instance of COVID-19		
**Group 2B History of thyroidism abnormality or thyroid ca**				
cannot have		diagnosis	UMLS:ICD10CM:E03	Other hypothyroidism
	or	diagnosis	UMLS:ICD10CM:E03.9	Hypothyroidism, unspecified
	or	diagnosis	UMLS:ICD10CM:E06	Thyroiditis

or	diagnosis	UMLS:ICD10CM:E03.3	Postinfectious hypothyroidism
or	diagnosis	UMLS:ICD10CM:E03.8	Other specified hypothyroidism
or	diagnosis	UMLS:ICD10CM:E05	Thyrotoxicosis [hyperthyroidism]
or	diagnosis	UMLS:ICD10CM:E06	Thyroiditis
or	diagnosis	UMLS:ICD10CM:E05.80	Other thyrotoxicosis without thyrotoxic crisis or storm
or	diagnosis	UMLS:ICD10CM:E05.40	Thyrotoxicosis factitia without thyrotoxic crisis or storm
or	diagnosis	UMLS:ICD10CM:E05.30	Thyrotoxicosis from ectopic thyroid tissue without thyrotoxic crisis or storm
or	diagnosis	UMLS:ICD10CM:E05.31	Thyrotoxicosis from ectopic thyroid tissue with thyrotoxic crisis or storm
or	diagnosis	UMLS:ICD10CM:Z85.850	Personal history of malignant neoplasm of thyroid
or	diagnosis	UMLS:ICD10CM:C73	Malignant neoplasm of thyroid gland

Query Criteria for Cohort post-COVID with hypothyroidism

This query was run on the network Global Collaborative Network with 147

HCO(s) queried and 147 HCO(s) responded. A total of 103 provider(s) responded with patients. The final cohort included 41,883 patients who matched the query criteria listed in the table below.

**Ungrouped terms**				
must have		demographics	Age	Age (at least 18 years (most recent occurrence))
	and any of	demographics	UMLS:HL7V3.0:Gender:M	Male
		demographics	UMLS:HL7V3.0:Gender:F	Female
**Group 1**				
**Group 1A COVID-19, no vaccination**				
must have	any of	diagnosis	UMLS:ICD10CM:U07.1	COVID-19
		diagnosis	UMLS:ICD10CM:U07.2	COVID-19, virus not identified (WHO)
		diagnosis	UMLS:ICD10CM:J12.82	Pneumonia due to coronavirus disease 2019
		laboratory	UMLS:LNC:94500-6	SARS-CoV-2 (COVID-19) RNA [Presence] in Respiratory specimen by NAA with probe detection (labResult: Positive)
		laboratory	UMLS:LNC:94309-2	SARS-CoV-2 (COVID-19) RNA [Presence] in Specimen by NAA with probe detection (labResult: Positive)
		laboratory	UMLS:LNC:94565-	SARS-CoV-2 (COVID-19)

			9	RNA [Presence] in Nasopharynx by NAA with non-probe detection (labResult: Positive)
		laboratory	UMLS:LNC:94759-8	SARS-CoV-2 (COVID-19) RNA [Presence] in Nasopharynx by NAA with probe detection (labResult: Positive)
		laboratory	UMLS:LNC:95608-6	SARS-CoV-2 (COVID-19) RNA [Presence] in Respiratory specimen by NAA with non-probe detection (labResult: Positive)
		laboratory	UMLS:LNC:94845-5	SARS-CoV-2 (COVID-19) RNA [Presence] in Saliva (oral fluid) by NAA with probe detection (labResult: Positive)
		laboratory	UMLS:LNC:95406-5	SARS-CoV-2 (COVID-19) RNA [Presence] in Nose by NAA with probe detection (labResult: Positive)
cannot have		medication	NLM:CVX:208	COVID-19, mRNA, LNP-S, PF, 30 mcg/0.3 mL dose
	or	medication	NLM:CVX:207	COVID-19, mRNA, LNP-S, PF, 100 mcg/0.5mL dose or 50 mcg/0.25mL dose
	or	medicatio	NLM:CVX:212	COVID-19 vaccine, vector-

	n		nr, rS-Ad26, PF, 0.5 mL
or	medication	NLM:RXNORM:OMOP5042939	COVID-19 vaccine
or	medication	NLM:CVX:300	COVID-19, mRNA, LNP-S, bivalent, PF, 30 mcg/0.3 mL dose
or	medication	NLM:CVX:217	COVID-19, mRNA, LNP-S, PF, 30 mcg/0.3 mL dose, tris-sucrose
or	medication	NLM:CVX:229	COVID-19, mRNA, LNP-S, bivalent, PF, 50 mcg/0.5 mL or 25mcg/0.25 mL dose
or	medication	NLM:CVX:218	COVID-19, mRNA, LNP-S, PF, 10 mcg/0.2 mL dose, tris-sucrose
or	medication	NLM:CVX:520	COVID-19 mRNA, bivalent, original/Omicron BA.1, Non-US Vaccine Product, Pfizer-BioNTech
or	medication	NLM:CVX:519	COVID-19 mRNA, bivalent, original/Omicron BA.1, Non-US Vaccine (Spikevax Bivalent), Moderna
or	medication	NLM:CVX:301	COVID-19, mRNA, LNP-S, bivalent, PF, 10 mcg/0.2 mL dose
or	medication	NLM:CVX:219	COVID-19, mRNA, LNP-S, PF, 3 mcg/0.2 mL dose, tris-sucrose

or	medicatio n	NLM:CVX:228	COVID-19, mRNA, LNP-S, PF, pediatric 25 mcg/0.25 mL dose
or	medicatio n	NLM:CVX:230	COVID-19, mRNA, LNP-S, bivalent booster, PF, 10 mcg/0.2 mL
or	medicatio n	NLM:CVX:221	COVID-19, mRNA, LNP-S, PF, 50 mcg/0.5 mL dose
or	medicatio n	NLM:CVX:210	COVID-19 vaccine, vector-nr, rS-ChAdOx1, PF, 0.5 mL
or	medicatio n	NLM:CVX:302	COVID-19, mRNA, LNP-S, bivalent, PF, 3 mcg/0.2 mL dose
or	medicatio n	NLM:CVX:511	COVID-19 IV Non-US Vaccine (CoronaVac, Sinovac)
or	medicatio n	NLM:RXNORM:246 8231	SARS-CoV-2 (COVID-19) vaccine, mRNA spike protein
or	procedure	UMLS:CPT:91300	Severe acute respiratory syndrome coronavirus 2 (SARS-CoV-2) (coronavirus disease [COVID-19]) vaccine, mRNA-LNP, spike protein, preservative free, 30 mcg/0.3 mL dosage, diluent reconstituted, for intramuscular use
or	procedure	UMLS:CPT:0001A	Immunization administration by

			intramuscular injection of severe acute respiratory syndrome coronavirus 2 (SARS-CoV-2) (coronavirus disease [COVID-19]) vaccine, mRNA-LNP, spike protein, preservative free, 30 mcg/0.3 mL dosage, diluent reconstituted; first dose
or	procedure	UMLS:CPT:0002A	Immunization administration by intramuscular injection of severe acute respiratory syndrome coronavirus 2 (SARS-CoV-2) (coronavirus disease [COVID-19]) vaccine, mRNA-LNP, spike protein, preservative free, 30 mcg/0.3 mL dosage, diluent reconstituted; second dose
or	procedure	UMLS:CPT:91301	Severe acute respiratory syndrome coronavirus 2 (SARS-CoV-2) (coronavirus disease [COVID-19]) vaccine, mRNA-LNP, spike protein, preservative free, 100 mcg/0.5 mL dosage, for intramuscular use
or	procedure	UMLS:CPT:0011A	Immunization administration by intramuscular injection of severe acute respiratory

			syndrome coronavirus 2 (SARS-CoV-2) (coronavirus disease [COVID-19]) vaccine, mRNA-LNP, spike protein, preservative free, 100 mcg/0.5 mL dosage; first dose
or	procedure	UMLS:CPT:0012A	Immunization administration by intramuscular injection of severe acute respiratory syndrome coronavirus 2 (SARS-CoV-2) (coronavirus disease [COVID-19]) vaccine, mRNA-LNP, spike protein, preservative free, 100 mcg/0.5 mL dosage; second dose
or	procedure	UMLS:SNOMED:840534001	Administration of SARS-CoV-2 antigen vaccine
or	medication	NLM:CVX:213	SARS-CoV-2 (COVID-19) Vaccine
or	procedure	UMLS:CPT:1036660	Immunization administration by intramuscular injection of severe acute respiratory syndrome coronavirus 2 (SARS-CoV-2) (coronavirus disease [COVID-19]) vaccine, mRNA-LNP, spike protein, preservative free, 30

			mcg/0.3 mL dosage, diluent reconstituted
or	procedure	UMLS:CPT:1036663	Immunization administration by intramuscular injection of severe acute respiratory syndrome coronavirus 2 (SARS-CoV-2) (coronavirus disease [COVID-19]) vaccine, mRNA-LNP, spike protein, preservative free, 100 mcg/0.5 mL dosage
or	procedure	UMLS:CPT:0124A	Immunization administration by intramuscular injection of severe acute respiratory syndrome coronavirus 2 (SARS-CoV-2) (coronavirus disease [COVID-19]) vaccine, mRNA-LNP, bivalent spike protein, preservative free, 30 mcg/0.3 mL dosage, tris-sucrose formulation, booster dose
or	procedure	UMLS:CPT:0004A	Immunization administration by intramuscular injection of severe acute respiratory syndrome coronavirus 2 (SARS-CoV-2) (coronavirus disease [COVID-19])

			vaccine, mRNA-LNP, spike protein, preservative free, 30 mcg/0.3 mL dosage, diluent reconstituted; booster dose
or	procedure	UMLS:CPT:0003A	Immunization administration by intramuscular injection of severe acute respiratory syndrome coronavirus 2 (SARS-CoV-2) (coronavirus disease [COVID-19]) vaccine, mRNA-LNP, spike protein, preservative free, 30 mcg/0.3 mL dosage, diluent reconstituted; third dose
or	procedure	UMLS:CPT:1037166	Immunization administration by intramuscular injection of severe acute respiratory syndrome coronavirus 2 (SARS-CoV-2) (coronavirus disease [COVID-19]) vaccine, mRNA-LNP, spike protein, preservative free, 30 mcg/0.3 mL dosage, tris-sucrose formulation
or	procedure	UMLS:CPT:0054A	Immunization administration by intramuscular injection of severe acute respiratory syndrome coronavirus 2 (SARS-CoV-2) (coronavirus

			disease [COVID-19]) vaccine, mRNA-LNP, spike protein, preservative free, 30 mcg/0.3 mL dosage, tris-sucrose formulation; booster dose
or	procedure	UMLS:CPT:0064A	Immunization administration by intramuscular injection of severe acute respiratory syndrome coronavirus 2 (SARS-CoV-2) (coronavirus disease [COVID-19]) vaccine, mRNA-LNP, spike protein, preservative free, 50 mcg/0.25 mL dosage, booster dose
or	procedure	UMLS:CPT:90480	Immunization administration by intramuscular injection of severe acute respiratory syndrome coronavirus 2 (SARS-CoV-2) (coronavirus disease [COVID-19]) vaccine, single dose
or	procedure	UMLS:CPT:1037171	Immunization administration by intramuscular injection of severe acute respiratory syndrome coronavirus 2 (SARS-CoV-2) (coronavirus disease [COVID-19])

			vaccine, mRNA-LNP, spike protein, preservative free, 10 mcg/0.2 mL dosage, diluent reconstituted, tris-sucrose formulation
or	procedure	UMLS:CPT:0071A	Immunization administration by intramuscular injection of severe acute respiratory syndrome coronavirus 2 (SARS-CoV-2) (coronavirus disease [COVID-19]) vaccine, mRNA-LNP, spike protein, preservative free, 10 mcg/0.2 mL dosage, diluent reconstituted, tris-sucrose formulation; first dose
or	procedure	UMLS:CPT:0072A	Immunization administration by intramuscular injection of severe acute respiratory syndrome coronavirus 2 (SARS-CoV-2) (coronavirus disease [COVID-19]) vaccine, mRNA-LNP, spike protein, preservative free, 10 mcg/0.2 mL dosage, diluent reconstituted, tris-sucrose formulation; second dose
or	medicatio n	NLM:RXNORM:261 0319	SARS-CoV-2 (COVID-19) vaccine, mRNA-BNT162b2 0.05 MG/ML / SARS-CoV-2

			(COVID-19) vaccine, mRNA-BNT162b2 OMICRON (BA.4/BA.5) 0.05 MG/ML Injectable Suspension
or	procedure	UMLS:CPT:91313	Severe acute respiratory syndrome coronavirus 2 (SARS-CoV-2) (coronavirus disease [COVID-19]) vaccine, mRNA-LNP, spike protein, bivalent, preservative free, 50 mcg/0.5 mL dosage, for intramuscular use
or	procedure	UMLS:CPT:0134A	Immunization administration by intramuscular injection of severe acute respiratory syndrome coronavirus 2 (SARS-CoV-2) (coronavirus disease [COVID-19]) vaccine, mRNA-LNP, spike protein, bivalent, preservative free, 50 mcg/0.5 mL dosage, booster dose
or	procedure	UMLS:CPT:1037175	Immunization administration by intramuscular injection of severe acute respiratory syndrome coronavirus 2 (SARS-CoV-2) (coronavirus disease [COVID-19])

			vaccine, DNA, spike protein, adenovirus type 26 (Ad26) vector, preservative free, 5x1010 viral particles/0.5 mL dosage
or	medicatio n	NLM:RXNORM:261 0347	0.3 ML SARS-CoV-2 (COVID-19) vaccine, mRNA-BNT162b2 0.05 MG/ML / SARS-CoV-2 (COVID-19) vaccine, mRNA-BNT162b2 OMICRON (BA.4/BA.5) -1 MG/ML Injection
or	procedure	UMLS:CPT:103722 8	Immunization administration by intramuscular injection of severe acute respiratory syndrome coronavirus 2 (SARS-CoV-2) (coronavirus disease [COVID-19]) vaccine, mRNA-LNP, spike protein, preservative free, 3 mcg/0.2 mL dosage, diluent reconstituted, tris-sucrose formulation
or	procedure	UMLS:CPT:0013A	Immunization administration by intramuscular injection of severe acute respiratory syndrome coronavirus 2 (SARS-CoV-2) (coronavirus disease [COVID-19]) vaccine, mRNA-LNP, spike

			protein, preservative free, 100 mcg/0.5 mL dosage; third dose
or	procedure	UMLS:CPT:0081A	Immunization administration by intramuscular injection of severe acute respiratory syndrome coronavirus 2 (SARS-CoV-2) (coronavirus disease [COVID-19]) vaccine, mRNA-LNP, spike protein, preservative free, 3 mcg/0.2 mL dosage, diluent reconstituted, tris-sucrose formulation; first dose
or	procedure	UMLS:CPT:0082A	Immunization administration by intramuscular injection of severe acute respiratory syndrome coronavirus 2 (SARS-CoV-2) (coronavirus disease [COVID-19]) vaccine, mRNA-LNP, spike protein, preservative free, 3 mcg/0.2 mL dosage, diluent reconstituted, tris-sucrose formulation; second dose
or	medication	NLM:RXNORM:2610328	SARS-CoV-2 (COVID-19) vaccine, mRNA-1273 0.05 MG/ML / SARS-CoV-2 (COVID-19) vaccine, mRNA-1273 OMICRON (BA.4/BA.5)

			0.05 MG/ML Injectable Suspension
or	procedure	UMLS:CPT:0154A	Immunization administration by intramuscular injection of severe acute respiratory syndrome coronavirus 2 (SARS-CoV-2) (coronavirus disease [COVID-19]) vaccine, mRNA-LNP, bivalent spike protein, preservative free, 10 mcg/0.2 mL dosage, diluent reconstituted, tris-sucrose formulation, booster dose
or	procedure	UMLS:CPT:0053A	Immunization administration by intramuscular injection of severe acute respiratory syndrome coronavirus 2 (SARS-CoV-2) (coronavirus disease [COVID-19]) vaccine, mRNA-LNP, spike protein, preservative free, 30 mcg/0.3 mL dosage, tris-sucrose formulation; third dose
or	procedure	UMLS:CPT:1037332	Immunization administration by intramuscular injection of severe acute respiratory syndrome coronavirus 2

			(SARS-CoV-2) (coronavirus disease [COVID-19]) vaccine, mRNA-LNP, spike protein, preservative free, 25 mcg/0.25 mL dosage
or	procedure	UMLS:CPT:0052A	Immunization administration by intramuscular injection of severe acute respiratory syndrome coronavirus 2 (SARS-CoV-2) (coronavirus disease [COVID-19]) vaccine, mRNA-LNP, spike protein, preservative free, 30 mcg/0.3 mL dosage, tris-sucrose formulation; second dose
or	procedure	UMLS:CPT:0111A	Immunization administration by intramuscular injection of severe acute respiratory syndrome coronavirus 2 (SARS-CoV-2) (coronavirus disease [COVID-19]) vaccine, mRNA-LNP, spike protein, preservative free, 25 mcg/0.25 mL dosage; first dose
or	procedure	UMLS:CPT:0051A	Immunization administration by intramuscular injection of severe acute respiratory

			syndrome coronavirus 2 (SARS-CoV-2) (coronavirus disease [COVID-19]) vaccine, mRNA-LNP, spike protein, preservative free, 30 mcg/0.3 mL dosage, tris-sucrose formulation; first dose
or	procedure	UMLS:CPT:91311	Severe acute respiratory syndrome coronavirus 2 (SARS-CoV-2) (coronavirus disease [COVID-19]) vaccine, mRNA-LNP, spike protein, preservative free, 25 mcg/0.25 mL dosage, for intramuscular use
or	procedure	UMLS:CPT:0074A	Immunization administration by intramuscular injection of severe acute respiratory syndrome coronavirus 2 (SARS-CoV-2) (coronavirus disease [COVID-19]) vaccine, mRNA-LNP, spike protein, preservative free, 10 mcg/0.2 mL dosage, diluent reconstituted, tris-sucrose formulation; booster dose
or	procedure	UMLS:CPT:0112A	Immunization administration by intramuscular injection of severe acute respiratory

			syndrome coronavirus 2 (SARS-CoV-2) (coronavirus disease [COVID-19]) vaccine, mRNA-LNP, spike protein, preservative free, 25 mcg/0.25 mL dosage; second dose
or	procedure	UMLS:CPT:0083A	Immunization administration by intramuscular injection of severe acute respiratory syndrome coronavirus 2 (SARS-CoV-2) (coronavirus disease [COVID-19]) vaccine, mRNA-LNP, spike protein, preservative free, 3 mcg/0.2 mL dosage, diluent reconstituted, tris-sucrose formulation; third dose
or	procedure	UMLS:CPT:0073A	Immunization administration by intramuscular injection of severe acute respiratory syndrome coronavirus 2 (SARS-CoV-2) (coronavirus disease [COVID-19]) vaccine, mRNA-LNP, spike protein, preservative free, 10 mcg/0.2 mL dosage, diluent reconstituted, tris-sucrose formulation; third dose

or	procedure	UMLS:CPT:0173A	Immunization administration by intramuscular injection of severe acute respiratory syndrome coronavirus 2 (SARS-CoV-2) (coronavirus disease [COVID-19]) vaccine, mRNA-LNP, bivalent spike protein, preservative free, 3 mcg/0.2 mL dosage, diluent reconstituted, tris-sucrose formulation, third dose
or	procedure	UMLS:CPT:0164A	Immunization administration by intramuscular injection of severe acute respiratory syndrome coronavirus 2 (SARS-CoV-2) (coronavirus disease [COVID-19]) vaccine, mRNA-LNP, spike protein, bivalent, preservative free, 10 mcg/0.2 mL dosage, booster dose
or	procedure	UMLS:CPT:1037838	Immunization administration by intramuscular injection of severe acute respiratory syndrome coronavirus 2 (SARS-CoV-2) (coronavirus disease [COVID-19])

			vaccine, mRNA-LNP, spike protein, preservative free, 50 mcg/0.5 mL dosage
or	procedure	UMLS:CPT:0094A	Immunization administration by intramuscular injection of severe acute respiratory syndrome coronavirus 2 (SARS-CoV-2) (coronavirus disease [COVID-19]) vaccine, mRNA-LNP, spike protein, preservative free, 50 mcg/0.5 mL dosage; booster dose, when administered to individuals 18 years and over
or	procedure	UMLS:CPT:0034A	Immunization administration by intramuscular injection of severe acute respiratory syndrome coronavirus 2 (SARS-CoV-2) (coronavirus disease [COVID-19]) vaccine, DNA, spike protein, adenovirus type 26 (Ad26) vector, preservative free, 5x1010 viral particles/0.5 mL dosage; booster dose
or	procedure	UMLS:CPT:0144A	Immunization administration by intramuscular injection of severe acute respiratory

			syndrome coronavirus 2 (SARS-CoV-2) (coronavirus disease [COVID-19]) vaccine, mRNA-LNP, spike protein, bivalent, preservative free, 25 mcg/0.25 mL dosage, booster dose
or	procedure	UMLS:CPT:0091A	Immunization administration by intramuscular injection of severe acute respiratory syndrome coronavirus 2 (SARS-CoV-2) (coronavirus disease [COVID-19]) vaccine, mRNA-LNP, spike protein, preservative free, 50 mcg/0.5 mL dosage; first dose, when administered to individuals 6 through 11 years
or	procedure	UMLS:CPT:0174A	Immunization administration by intramuscular injection of severe acute respiratory syndrome coronavirus 2 (SARS-CoV-2) (coronavirus disease [COVID-19]) vaccine, mRNA-LNP, bivalent spike protein, preservative free, 3 mcg/0.2 mL dosage, diluent

			reconstituted, tris-sucrose formulation, booster
or	procedure	UMLS:CPT:0092A	Immunization administration by intramuscular injection of severe acute respiratory syndrome coronavirus 2 (SARS-CoV-2) (coronavirus disease [COVID-19]) vaccine, mRNA-LNP, spike protein, preservative free, 50 mcg/0.5 mL dosage; second dose, when administered to individuals 6 through 11 years
or	procedure	UMLS:CPT:1036682	Immunization administration by intramuscular injection of severe acute respiratory syndrome coronavirus 2 (SARS-CoV-2) (coronavirus disease [COVID-19]) vaccine, recombinant spike protein nanoparticle, saponin-based adjuvant, preservative free, 5 mcg/0.5 mL dosage
or	procedure	UMLS:CPT:0041A	Immunization administration by intramuscular injection of severe acute respiratory syndrome coronavirus 2

			(SARS-CoV-2) (coronavirus disease [COVID-19]) vaccine, recombinant spike protein nanoparticle, saponin-based adjuvant, preservative free, 5 mcg/0.5 mL dosage; first dose
or	procedure	UMLS:CPT:0113A	Immunization administration by intramuscular injection of severe acute respiratory syndrome coronavirus 2 (SARS-CoV-2) (coronavirus disease [COVID-19]) vaccine, mRNA-LNP, spike protein, preservative free, 25 mcg/0.25 mL dosage; third dose
or	procedure	UMLS:CPT:0042A	Immunization administration by intramuscular injection of severe acute respiratory syndrome coronavirus 2 (SARS-CoV-2) (coronavirus disease [COVID-19]) vaccine, recombinant spike protein nanoparticle, saponin-based adjuvant, preservative free, 5 mcg/0.5 mL dosage; second dose
or	procedure	UMLS:CPT:0093A	Immunization administration by

			intramuscular injection of severe acute respiratory syndrome coronavirus 2 (SARS-CoV-2) (coronavirus disease [COVID-19]) vaccine, mRNA-LNP, spike protein, preservative free, 50 mcg/0.5 mL dosage; third dose, when administered to individuals 6 through 11 years
or	procedure	UMLS:CPT:1036666	Immunization administration by intramuscular injection of severe acute respiratory syndrome coronavirus 2 (SARS-CoV-2) (coronavirus disease [COVID-19]) vaccine, DNA, spike protein, chimpanzee adenovirus Oxford 1 (ChAdOx1) vector, preservative free, 5x1010 viral particles/0.5 mL dosage
or	procedure	UMLS:CPT:0044A	Immunization administration by intramuscular injection of severe acute respiratory syndrome coronavirus 2 (SARS-CoV-2) (coronavirus disease [COVID-19]) vaccine, recombinant spike

				protein nanoparticle, saponin-based adjuvant, preservative free, 5 mcg/0.5mL dosage; booster
date constraint	The terms in this group occurred between Dec 1, 2019 and Dec 31, 2023			
event relationship	Any instance of hyperthyroidism occurred at least 1 day after any instance of COVID-19, no vaccination			
**Group 1B hyperthyroidism**				
must have	any of	diagnosis	UMLS:ICD10CM:E03	Other hypothyroidism
		diagnosis	UMLS:ICD10CM:E03.9	Hypothyroidism, unspecified
		diagnosis	UMLS:ICD10CM:E03.8	Other specified hypothyroidism
		diagnosis	UMLS:ICD10CM:E06	Thyroiditis
		diagnosis	UMLS:ICD10CM:E03.3	Postinfectious hypothyroidism
**Group 2**				
**Group 2A COVID-19**				
must have	any of	diagnosis	UMLS:ICD10CM:U07.1	COVID-19
		diagnosis	UMLS:ICD10CM:U07.2	COVID-19, virus not identified (WHO)
		diagnosis	UMLS:ICD10CM:J12.82	Pneumonia due to coronavirus disease 2019

laboratory	UMLS:LNC:94500-6	SARS-CoV-2 (COVID-19) RNA [Presence] in Respiratory specimen by NAA with probe detection (labResult: Positive)
laboratory	UMLS:LNC:94309-2	SARS-CoV-2 (COVID-19) RNA [Presence] in Specimen by NAA with probe detection (labResult: Positive)
laboratory	UMLS:LNC:94565-9	SARS-CoV-2 (COVID-19) RNA [Presence] in Nasopharynx by NAA with non-probe detection (labResult: Positive)
laboratory	UMLS:LNC:94759-8	SARS-CoV-2 (COVID-19) RNA [Presence] in Nasopharynx by NAA with probe detection (labResult: Positive)
laboratory	UMLS:LNC:95608-6	SARS-CoV-2 (COVID-19) RNA [Presence] in Respiratory specimen by NAA with non-probe detection (labResult: Positive)
laboratory	UMLS:LNC:94845-5	SARS-CoV-2 (COVID-19) RNA [Presence] in Saliva (oral fluid) by NAA with probe detection (labResult: Positive)

		laboratory	UMLS:LNC:95406-5	SARS-CoV-2 (COVID-19) RNA [Presence] in Nose by NAA with probe detection (labResult: Positive)
	and	visit	TNX:Visit	Visit (Data Source: TriNetX)
date constraint		The terms in this group occurred at any time		
event relationship		Any instance of Hx of thyroid abnormality and thyroid ca occurred on or before any instance of COVID-19		
**Group 2B Hx of thyroid abnormality and thyroid ca**				
cannot have		diagnosis	UMLS:ICD10CM:E03	Other hypothyroidism
	or	diagnosis	UMLS:ICD10CM:E03.9	Hypothyroidism, unspecified
	or	diagnosis	UMLS:ICD10CM:E03.8	Other specified hypothyroidism
	or	diagnosis	UMLS:ICD10CM:E03.3	Postinfectious hypothyroidism
	or	diagnosis	UMLS:ICD10CM:E06	Thyroiditis
	or	diagnosis	UMLS:ICD10CM:C73	Malignant neoplasm of thyroid gland
	or	diagnosis	UMLS:ICD10CM:Z85.850	Personal history of malignant neoplasm of thyroid
	or	diagnosis	UMLS:ICD10CM:E05	Thyrotoxicosis [hyperthyroidism]
	or	diagnosis	UMLS:ICD10CM:E	Other thyrotoxicosis without

		05.80	thyrotoxic crisis or storm
or	diagnosis	UMLS:ICD10CM:E05.30	Thyrotoxicosis from ectopic thyroid tissue without thyrotoxic crisis or storm
or	diagnosis	UMLS:ICD10CM:E05.31	Thyrotoxicosis from ectopic thyroid tissue with thyrotoxic crisis or storm

Query Criteria for Cohort post-COVID with hyperthyroidism

This query was run on the network Global Collaborative Network with 148 HCO(s) queried and 148 HCO(s) responded. A total of 103 provider(s) responded with patients. The final cohort included 38,236 patients who matched the query criteria listed in the table below.

**Ungrouped terms**				
must have		demographics	Age	Age (at least 18 years (most recent occurrence))
	and any of	demographics	UMLS:HL7V3.0:Gender:M	Male
		demographics	UMLS:HL7V3.0:Gender:F	Female
**Group 1**				
**Group 1A COVID-19, no vaccination**				
must have	any of	diagnosis	UMLS:ICD10CM:U07.1	COVID-19
		diagnosis	UMLS:ICD10CM:U07.2	COVID-19, virus not identified (WHO)

diagnosis	UMLS:ICD10CM:J12.82	Pneumonia due to coronavirus disease 2019
laboratory	UMLS:LNC:94500-6	SARS-CoV-2 (COVID-19) RNA [Presence] in Respiratory specimen by NAA with probe detection (labResult: Positive)
laboratory	UMLS:LNC:94309-2	SARS-CoV-2 (COVID-19) RNA [Presence] in Specimen by NAA with probe detection (labResult: Positive)
laboratory	UMLS:LNC:94565-9	SARS-CoV-2 (COVID-19) RNA [Presence] in Nasopharynx by NAA with non-probe detection (labResult: Positive)
laboratory	UMLS:LNC:94759-8	SARS-CoV-2 (COVID-19) RNA [Presence] in Nasopharynx by NAA with probe detection (labResult: Positive)
laboratory	UMLS:LNC:95608-6	SARS-CoV-2 (COVID-19) RNA [Presence] in Respiratory specimen by NAA with non-probe detection (labResult: Positive)
laboratory	UMLS:LNC:94845-5	SARS-CoV-2 (COVID-19) RNA [Presence] in Saliva (oral fluid) by NAA with probe detection (labResult:

				Positive)
		laboratory	UMLS:LNC:95406-5	SARS-CoV-2 (COVID-19) RNA [Presence] in Nose by NAA with probe detection (labResult: Positive)
cannot have		medication	NLM:CVX:208	COVID-19, mRNA, LNP-S, PF, 30 mcg/0.3 mL dose
	or	medication	NLM:CVX:207	COVID-19, mRNA, LNP-S, PF, 100 mcg/0.5mL dose or 50 mcg/0.25mL dose
	or	medication	NLM:CVX:212	COVID-19 vaccine, vector-nr, rS-Ad26, PF, 0.5 mL
	or	medication	NLM:RXNORM:OMOP5042939	COVID-19 vaccine
	or	medication	NLM:CVX:300	COVID-19, mRNA, LNP-S, bivalent, PF, 30 mcg/0.3 mL dose
	or	medication	NLM:CVX:217	COVID-19, mRNA, LNP-S, PF, 30 mcg/0.3 mL dose, tris-sucrose
	or	medication	NLM:CVX:229	COVID-19, mRNA, LNP-S, bivalent, PF, 50 mcg/0.5 mL or 25mcg/0.25 mL dose
	or	medication	NLM:CVX:218	COVID-19, mRNA, LNP-S, PF, 10 mcg/0.2 mL dose, tris-sucrose
	or	medication	NLM:CVX:520	COVID-19 mRNA, bivalent, original/Omicron BA.1, Non-US Vaccine Product, Pfizer-

			BioNTech
or	medicatio n	NLM:CVX:519	COVID-19 mRNA, bivalent, original/Omicron BA.1, Non-US Vaccine (Spikevax Bivalent), Moderna
or	medicatio n	NLM:CVX:301	COVID-19, mRNA, LNP-S, bivalent, PF, 10 mcg/0.2 mL dose
or	medicatio n	NLM:CVX:219	COVID-19, mRNA, LNP-S, PF, 3 mcg/0.2 mL dose, tris-sucrose
or	medicatio n	NLM:CVX:228	COVID-19, mRNA, LNP-S, PF, pediatric 25 mcg/0.25 mL dose
or	medicatio n	NLM:CVX:230	COVID-19, mRNA, LNP-S, bivalent booster, PF, 10 mcg/0.2 mL
or	medicatio n	NLM:CVX:221	COVID-19, mRNA, LNP-S, PF, 50 mcg/0.5 mL dose
or	medicatio n	NLM:CVX:210	COVID-19 vaccine, vector-nr, rS-ChAdOx1, PF, 0.5 mL
or	medicatio n	NLM:CVX:302	COVID-19, mRNA, LNP-S, bivalent, PF, 3 mcg/0.2 mL dose
or	medicatio n	NLM:CVX:511	COVID-19 IV Non-US Vaccine (CoronaVac, Sinovac)
or	medicatio	NLM:RXNORM:246	SARS-CoV-2 (COVID-19) vaccine, mRNA spike

	n	8231	protein
or	procedure	UMLS:CPT:91300	Severe acute respiratory syndrome coronavirus 2 (SARS-CoV-2) (coronavirus disease [COVID-19]) vaccine, mRNA-LNP, spike protein, preservative free, 30 mcg/0.3 mL dosage, diluent reconstituted, for intramuscular use
or	procedure	UMLS:CPT:0001A	Immunization administration by intramuscular injection of severe acute respiratory syndrome coronavirus 2 (SARS-CoV-2) (coronavirus disease [COVID-19]) vaccine, mRNA-LNP, spike protein, preservative free, 30 mcg/0.3 mL dosage, diluent reconstituted; first dose
or	procedure	UMLS:CPT:0002A	Immunization administration by intramuscular injection of severe acute respiratory syndrome coronavirus 2 (SARS-CoV-2) (coronavirus disease [COVID-19]) vaccine, mRNA-LNP, spike protein, preservative free, 30 mcg/0.3 mL dosage, diluent

			reconstituted; second dose
or	procedure	UMLS:CPT:91301	Severe acute respiratory syndrome coronavirus 2 (SARS-CoV-2) (coronavirus disease [COVID-19]) vaccine, mRNA-LNP, spike protein, preservative free, 100 mcg/0.5 mL dosage, for intramuscular use
or	procedure	UMLS:CPT:0011A	Immunization administration by intramuscular injection of severe acute respiratory syndrome coronavirus 2 (SARS-CoV-2) (coronavirus disease [COVID-19]) vaccine, mRNA-LNP, spike protein, preservative free, 100 mcg/0.5 mL dosage; first dose
or	procedure	UMLS:CPT:0012A	Immunization administration by intramuscular injection of severe acute respiratory syndrome coronavirus 2 (SARS-CoV-2) (coronavirus disease [COVID-19]) vaccine, mRNA-LNP, spike protein, preservative free, 100 mcg/0.5 mL dosage; second dose

or	procedure	UMLS:SNOMED:840534001	Administration of SARS-CoV-2 antigen vaccine
or	medication	NLM:CVX:213	SARS-CoV-2 (COVID-19) Vaccine
or	procedure	UMLS:CPT:1036660	Immunization administration by intramuscular injection of severe acute respiratory syndrome coronavirus 2 (SARS-CoV-2) (coronavirus disease [COVID-19]) vaccine, mRNA-LNP, spike protein, preservative free, 30 mcg/0.3 mL dosage, diluent reconstituted
or	procedure	UMLS:CPT:1036663	Immunization administration by intramuscular injection of severe acute respiratory syndrome coronavirus 2 (SARS-CoV-2) (coronavirus disease [COVID-19]) vaccine, mRNA-LNP, spike protein, preservative free, 100 mcg/0.5 mL dosage
or	procedure	UMLS:CPT:0124A	Immunization administration by intramuscular injection of severe acute respiratory syndrome coronavirus 2 (SARS-CoV-2) (coronavirus

			disease [COVID-19]) vaccine, mRNA-LNP, bivalent spike protein, preservative free, 30 mcg/0.3 mL dosage, tris-sucrose formulation, booster dose
or	procedure	UMLS:CPT:0004A	Immunization administration by intramuscular injection of severe acute respiratory syndrome coronavirus 2 (SARS-CoV-2) (coronavirus disease [COVID-19]) vaccine, mRNA-LNP, spike protein, preservative free, 30 mcg/0.3 mL dosage, diluent reconstituted; booster dose
or	procedure	UMLS:CPT:0003A	Immunization administration by intramuscular injection of severe acute respiratory syndrome coronavirus 2 (SARS-CoV-2) (coronavirus disease [COVID-19]) vaccine, mRNA-LNP, spike protein, preservative free, 30 mcg/0.3 mL dosage, diluent reconstituted; third dose
or	procedure	UMLS:CPT:1037166	Immunization administration by intramuscular injection of

			severe acute respiratory syndrome coronavirus 2 (SARS-CoV-2) (coronavirus disease [COVID-19]) vaccine, mRNA-LNP, spike protein, preservative free, 30 mcg/0.3 mL dosage, tris-sucrose formulation
or	procedure	UMLS:CPT:0054A	Immunization administration by intramuscular injection of severe acute respiratory syndrome coronavirus 2 (SARS-CoV-2) (coronavirus disease [COVID-19]) vaccine, mRNA-LNP, spike protein, preservative free, 30 mcg/0.3 mL dosage, tris-sucrose formulation; booster dose
or	procedure	UMLS:CPT:0064A	Immunization administration by intramuscular injection of severe acute respiratory syndrome coronavirus 2 (SARS-CoV-2) (coronavirus disease [COVID-19]) vaccine, mRNA-LNP, spike protein, preservative free, 50 mcg/0.25 mL dosage, booster dose

or	procedure	UMLS:CPT:90480	Immunization administration by intramuscular injection of severe acute respiratory syndrome coronavirus 2 (SARS-CoV-2) (coronavirus disease [COVID-19]) vaccine, single dose
or	procedure	UMLS:CPT:1037171	Immunization administration by intramuscular injection of severe acute respiratory syndrome coronavirus 2 (SARS-CoV-2) (coronavirus disease [COVID-19]) vaccine, mRNA-LNP, spike protein, preservative free, 10 mcg/0.2 mL dosage, diluent reconstituted, tris-sucrose formulation
or	procedure	UMLS:CPT:0071A	Immunization administration by intramuscular injection of severe acute respiratory syndrome coronavirus 2 (SARS-CoV-2) (coronavirus disease [COVID-19]) vaccine, mRNA-LNP, spike protein, preservative free, 10 mcg/0.2 mL dosage, diluent reconstituted, tris-sucrose formulation; first dose

or	procedure	UMLS:CPT:0072A	Immunization administration by intramuscular injection of severe acute respiratory syndrome coronavirus 2 (SARS-CoV-2) (coronavirus disease [COVID-19]) vaccine, mRNA-LNP, spike protein, preservative free, 10 mcg/0.2 mL dosage, diluent reconstituted, tris-sucrose formulation; second dose
or	medication	NLM:RXNORM:2610319	SARS-CoV-2 (COVID-19) vaccine, mRNA-BNT162b2 0.05 MG/ML / SARS-CoV-2 (COVID-19) vaccine, mRNA-BNT162b2 OMICRON (BA.4/BA.5) 0.05 MG/ML Injectable Suspension
or	procedure	UMLS:CPT:91313	Severe acute respiratory syndrome coronavirus 2 (SARS-CoV-2) (coronavirus disease [COVID-19]) vaccine, mRNA-LNP, spike protein, bivalent, preservative free, 50 mcg/0.5 mL dosage, for intramuscular use
or	procedure	UMLS:CPT:0134A	Immunization administration by intramuscular injection of severe acute respiratory

			syndrome coronavirus 2 (SARS-CoV-2) (coronavirus disease [COVID-19]) vaccine, mRNA-LNP, spike protein, bivalent, preservative free, 50 mcg/0.5 mL dosage, booster dose
or	procedure	UMLS:CPT:1037175	Immunization administration by intramuscular injection of severe acute respiratory syndrome coronavirus 2 (SARS-CoV-2) (coronavirus disease [COVID-19]) vaccine, DNA, spike protein, adenovirus type 26 (Ad26) vector, preservative free, 5x1010 viral particles/0.5 mL dosage
or	medication	NLM:RXNORM:2610347	0.3 ML SARS-CoV-2 (COVID-19) vaccine, mRNA-BNT162b2 0.05 MG/ML / SARS-CoV-2 (COVID-19) vaccine, mRNA-BNT162b2 OMICRON (BA.4/BA.5) -1 MG/ML Injection
or	procedure	UMLS:CPT:1037228	Immunization administration by intramuscular injection of severe acute respiratory syndrome coronavirus 2

			(SARS-CoV-2) (coronavirus disease [COVID-19]) vaccine, mRNA-LNP, spike protein, preservative free, 3 mcg/0.2 mL dosage, diluent reconstituted, tris-sucrose formulation
or	procedure	UMLS:CPT:0013A	Immunization administration by intramuscular injection of severe acute respiratory syndrome coronavirus 2 (SARS-CoV-2) (coronavirus disease [COVID-19]) vaccine, mRNA-LNP, spike protein, preservative free, 100 mcg/0.5 mL dosage; third dose
or	procedure	UMLS:CPT:0081A	Immunization administration by intramuscular injection of severe acute respiratory syndrome coronavirus 2 (SARS-CoV-2) (coronavirus disease [COVID-19]) vaccine, mRNA-LNP, spike protein, preservative free, 3 mcg/0.2 mL dosage, diluent reconstituted, tris-sucrose formulation; first dose
or	procedure	UMLS:CPT:0082A	Immunization administration by

			intramuscular injection of severe acute respiratory syndrome coronavirus 2 (SARS-CoV-2) (coronavirus disease [COVID-19]) vaccine, mRNA-LNP, spike protein, preservative free, 3 mcg/0.2 mL dosage, diluent reconstituted, tris-sucrose formulation; second dose
or	medication	NLM:RXNORM:2610328	SARS-CoV-2 (COVID-19) vaccine, mRNA-1273 0.05 MG/ML / SARS-CoV-2 (COVID-19) vaccine, mRNA-1273 OMICRON (BA.4/BA.5) 0.05 MG/ML Injectable Suspension
or	procedure	UMLS:CPT:0154A	Immunization administration by intramuscular injection of severe acute respiratory syndrome coronavirus 2 (SARS-CoV-2) (coronavirus disease [COVID-19]) vaccine, mRNA-LNP, bivalent spike protein, preservative free, 10 mcg/0.2 mL dosage, diluent reconstituted, tris-sucrose formulation, booster dose
or	procedure	UMLS:CPT:0053A	Immunization administration by

			intramuscular injection of severe acute respiratory syndrome coronavirus 2 (SARS-CoV-2) (coronavirus disease [COVID-19]) vaccine, mRNA-LNP, spike protein, preservative free, 30 mcg/0.3 mL dosage, tris-sucrose formulation; third dose
or	procedure	UMLS:CPT:1037332	Immunization administration by intramuscular injection of severe acute respiratory syndrome coronavirus 2 (SARS-CoV-2) (coronavirus disease [COVID-19]) vaccine, mRNA-LNP, spike protein, preservative free, 25 mcg/0.25 mL dosage
or	procedure	UMLS:CPT:0052A	Immunization administration by intramuscular injection of severe acute respiratory syndrome coronavirus 2 (SARS-CoV-2) (coronavirus disease [COVID-19]) vaccine, mRNA-LNP, spike protein, preservative free, 30 mcg/0.3 mL dosage, tris-sucrose formulation; second dose

or	procedure	UMLS:CPT:0111A	Immunization administration by intramuscular injection of severe acute respiratory syndrome coronavirus 2 (SARS-CoV-2) (coronavirus disease [COVID-19]) vaccine, mRNA-LNP, spike protein, preservative free, 25 mcg/0.25 mL dosage; first dose
or	procedure	UMLS:CPT:0051A	Immunization administration by intramuscular injection of severe acute respiratory syndrome coronavirus 2 (SARS-CoV-2) (coronavirus disease [COVID-19]) vaccine, mRNA-LNP, spike protein, preservative free, 30 mcg/0.3 mL dosage, tris-sucrose formulation; first dose
or	procedure	UMLS:CPT:91311	Severe acute respiratory syndrome coronavirus 2 (SARS-CoV-2) (coronavirus disease [COVID-19]) vaccine, mRNA-LNP, spike protein, preservative free, 25 mcg/0.25 mL dosage, for intramuscular use

or	procedure	UMLS:CPT:0074A	Immunization administration by intramuscular injection of severe acute respiratory syndrome coronavirus 2 (SARS-CoV-2) (coronavirus disease [COVID-19]) vaccine, mRNA-LNP, spike protein, preservative free, 10 mcg/0.2 mL dosage, diluent reconstituted, tris-sucrose formulation; booster dose
or	procedure	UMLS:CPT:0112A	Immunization administration by intramuscular injection of severe acute respiratory syndrome coronavirus 2 (SARS-CoV-2) (coronavirus disease [COVID-19]) vaccine, mRNA-LNP, spike protein, preservative free, 25 mcg/0.25 mL dosage; second dose
or	procedure	UMLS:CPT:0083A	Immunization administration by intramuscular injection of severe acute respiratory syndrome coronavirus 2 (SARS-CoV-2) (coronavirus disease [COVID-19]) vaccine, mRNA-LNP, spike protein, preservative free, 3

			mcg/0.2 mL dosage, diluent reconstituted, tris-sucrose formulation; third dose
or	procedure	UMLS:CPT:0073A	Immunization administration by intramuscular injection of severe acute respiratory syndrome coronavirus 2 (SARS-CoV-2) (coronavirus disease [COVID-19]) vaccine, mRNA-LNP, spike protein, preservative free, 10 mcg/0.2 mL dosage, diluent reconstituted, tris-sucrose formulation; third dose
or	procedure	UMLS:CPT:0173A	Immunization administration by intramuscular injection of severe acute respiratory syndrome coronavirus 2 (SARS-CoV-2) (coronavirus disease [COVID-19]) vaccine, mRNA-LNP, bivalent spike protein, preservative free, 3 mcg/0.2 mL dosage, diluent reconstituted, tris-sucrose formulation, third dose
or	procedure	UMLS:CPT:0164A	Immunization administration by intramuscular injection of severe acute respiratory

			syndrome coronavirus 2 (SARS-CoV-2) (coronavirus disease [COVID-19]) vaccine, mRNA-LNP, spike protein, bivalent, preservative free, 10 mcg/0.2 mL dosage, booster dose
or	procedure	UMLS:CPT:1037838	Immunization administration by intramuscular injection of severe acute respiratory syndrome coronavirus 2 (SARS-CoV-2) (coronavirus disease [COVID-19]) vaccine, mRNA-LNP, spike protein, preservative free, 50 mcg/0.5 mL dosage
or	procedure	UMLS:CPT:0094A	Immunization administration by intramuscular injection of severe acute respiratory syndrome coronavirus 2 (SARS-CoV-2) (coronavirus disease [COVID-19]) vaccine, mRNA-LNP, spike protein, preservative free, 50 mcg/0.5 mL dosage; booster dose, when administered to individuals 18 years and over

or	procedure	UMLS:CPT:0034A	Immunization administration by intramuscular injection of severe acute respiratory syndrome coronavirus 2 (SARS-CoV-2) (coronavirus disease [COVID-19]) vaccine, DNA, spike protein, adenovirus type 26 (Ad26) vector, preservative free, 5x1010 viral particles/0.5 mL dosage; booster dose
or	procedure	UMLS:CPT:0144A	Immunization administration by intramuscular injection of severe acute respiratory syndrome coronavirus 2 (SARS-CoV-2) (coronavirus disease [COVID-19]) vaccine, mRNA-LNP, spike protein, bivalent, preservative free, 25 mcg/0.25 mL dosage, booster dose
or	procedure	UMLS:CPT:0091A	Immunization administration by intramuscular injection of severe acute respiratory syndrome coronavirus 2 (SARS-CoV-2) (coronavirus disease [COVID-19]) vaccine, mRNA-LNP, spike

			protein, preservative free, 50 mcg/0.5 mL dosage; first dose, when administered to individuals 6 through 11 years
or	procedure	UMLS:CPT:0174A	Immunization administration by intramuscular injection of severe acute respiratory syndrome coronavirus 2 (SARS-CoV-2) (coronavirus disease [COVID-19]) vaccine, mRNA-LNP, bivalent spike protein, preservative free, 3 mcg/0.2 mL dosage, diluent reconstituted, tris-sucrose formulation, booster
or	procedure	UMLS:CPT:0092A	Immunization administration by intramuscular injection of severe acute respiratory syndrome coronavirus 2 (SARS-CoV-2) (coronavirus disease [COVID-19]) vaccine, mRNA-LNP, spike protein, preservative free, 50 mcg/0.5 mL dosage; second dose, when administered to individuals 6 through 11 years

or	procedure	UMLS:CPT:1036682	Immunization administration by intramuscular injection of severe acute respiratory syndrome coronavirus 2 (SARS-CoV-2) (coronavirus disease [COVID-19]) vaccine, recombinant spike protein nanoparticle, saponin-based adjuvant, preservative free, 5 mcg/0.5 mL dosage
or	procedure	UMLS:CPT:0041A	Immunization administration by intramuscular injection of severe acute respiratory syndrome coronavirus 2 (SARS-CoV-2) (coronavirus disease [COVID-19]) vaccine, recombinant spike protein nanoparticle, saponin-based adjuvant, preservative free, 5 mcg/0.5 mL dosage; first dose
or	procedure	UMLS:CPT:0113A	Immunization administration by intramuscular injection of severe acute respiratory syndrome coronavirus 2 (SARS-CoV-2) (coronavirus disease [COVID-19]) vaccine, mRNA-LNP, spike

			protein, preservative free, 25 mcg/0.25 mL dosage; third dose
or	procedure	UMLS:CPT:0042A	Immunization administration by intramuscular injection of severe acute respiratory syndrome coronavirus 2 (SARS-CoV-2) (coronavirus disease [COVID-19]) vaccine, recombinant spike protein nanoparticle, saponin-based adjuvant, preservative free, 5 mcg/0.5 mL dosage; second dose
or	procedure	UMLS:CPT:0093A	Immunization administration by intramuscular injection of severe acute respiratory syndrome coronavirus 2 (SARS-CoV-2) (coronavirus disease [COVID-19]) vaccine, mRNA-LNP, spike protein, preservative free, 50 mcg/0.5 mL dosage; third dose, when administered to individuals 6 through 11 years
or	procedure	UMLS:CPT:1036666	Immunization administration by intramuscular injection of severe acute respiratory

				syndrome coronavirus 2 (SARS-CoV-2) (coronavirus disease [COVID-19]) vaccine, DNA, spike protein, chimpanzee adenovirus Oxford 1 (ChAdOx1) vector, preservative free, 5x1010 viral particles/0.5 mL dosage
	or	procedure	UMLS:CPT:0044A	Immunization administration by intramuscular injection of severe acute respiratory syndrome coronavirus 2 (SARS-CoV-2) (coronavirus disease [COVID-19]) vaccine, recombinant spike protein nanoparticle, saponin-based adjuvant, preservative free, 5 mcg/0.5mL dosage; booster
date constraint		The terms in this group occurred between Dec 1, 2019 and Dec 31, 2023		
event relationship		Any instance of hyperthyroidism occurred at least 1 day after any instance of COVID-19, no vaccination		
**Group 1B hyperthyroidism**				
must have	any of	diagnosis	UMLS:ICD10CM:E05	Thyrotoxicosis [hyperthyroidism]
		diagnosis	UMLS:ICD10CM:E06	Thyroiditis

Group 2				
**Group 2A COVID-19**				
must have	any of	diagnosis	UMLS:ICD10CM:U07.1	COVID-19
		diagnosis	UMLS:ICD10CM:U07.2	COVID-19, virus not identified (WHO)
		diagnosis	UMLS:ICD10CM:J12.82	Pneumonia due to coronavirus disease 2019
		laboratory	UMLS:LNC:94500-6	SARS-CoV-2 (COVID-19) RNA [Presence] in Respiratory specimen by NAA with probe detection (labResult: Positive)
		laboratory	UMLS:LNC:94309-2	SARS-CoV-2 (COVID-19) RNA [Presence] in Specimen by NAA with probe detection (labResult: Positive)
		laboratory	UMLS:LNC:94565-9	SARS-CoV-2 (COVID-19) RNA [Presence] in Nasopharynx by NAA with non-probe detection (labResult: Positive)
		laboratory	UMLS:LNC:94759-8	SARS-CoV-2 (COVID-19) RNA [Presence] in Nasopharynx by NAA with probe detection (labResult: Positive)
		laboratory	UMLS:LNC:95608-6	SARS-CoV-2 (COVID-19) RNA [Presence] in

				Respiratory specimen by NAA with non-probe detection (labResult: Positive)
		laboratory	UMLS:LNC:94845-5	SARS-CoV-2 (COVID-19) RNA [Presence] in Saliva (oral fluid) by NAA with probe detection (labResult: Positive)
		laboratory	UMLS:LNC:95406-5	SARS-CoV-2 (COVID-19) RNA [Presence] in Nose by NAA with probe detection (labResult: Positive)
	and	visit	TNX:Visit	Visit (Data Source: TriNetX)
date constraint		The terms in this group occurred at any time		
event relationship		Any instance of thyroid cancer history occurred at least 1 day before the first instance of COVID-19		
**Group 2B thyroid cancer history**				
cannot have		diagnosis	UMLS:ICD10CM:C73	Malignant neoplasm of thyroid gland
	or	diagnosis	UMLS:ICD10CM:Z85.850	Personal history of malignant neoplasm of thyroid

Query Criteria for Cohort non-COVID, with hyperthyroidism

This query was run on the network Global Collaborative Network with 146 HCO(s) queried and 146 HCO(s) responded. A total of 125 provider(s) responded with patients. The final cohort included 260,468 patients who matched the query criteria listed in the table below.

**Ungrouped terms**				
must have		demographics	Age	Age (at least 18 years (most recent occurrence))
	and any of	demographics	UMLS:HL7V3.0:Gender:M	Male
		demographics	UMLS:HL7V3.0:Gender:F	Female
**Group 1**				
**COVID-19 negative, no vaccination**				
must have		visit	TNX:Visit	Visit
cannot have		medication	NLM:CVX:208	COVID-19, mRNA, LNP-S, PF, 30 mcg/0.3 mL dose
	or	medication	NLM:CVX:207	COVID-19, mRNA, LNP-S, PF, 100 mcg/0.5mL dose or 50 mcg/0.25mL dose
	or	medication	NLM:CVX:212	COVID-19 vaccine, vector-nr, rS-Ad26, PF, 0.5 mL
	or	medication	NLM:RXNORM:OMOP5042939	COVID-19 vaccine
	or	medication	NLM:CVX:300	COVID-19, mRNA, LNP-S, bivalent, PF, 30 mcg/0.3 mL dose
	or	medication	NLM:CVX:217	COVID-19, mRNA, LNP-S, PF, 30 mcg/0.3 mL dose, tris-sucrose
	or	medicatio	NLM:CVX:229	COVID-19, mRNA, LNP-S, bivalent, PF, 50 mcg/0.5 mL

	n		or 25mcg/0.25 mL dose
or	medication	NLM:CVX:218	COVID-19, mRNA, LNP-S, PF, 10 mcg/0.2 mL dose, tris-sucrose
or	medication	NLM:CVX:520	COVID-19 mRNA, bivalent, original/Omicron BA.1, Non-US Vaccine Product, Pfizer-BioNTech
or	medication	NLM:CVX:519	COVID-19 mRNA, bivalent, original/Omicron BA.1, Non-US Vaccine (Spikevax Bivalent), Moderna
or	medication	NLM:CVX:301	COVID-19, mRNA, LNP-S, bivalent, PF, 10 mcg/0.2 mL dose
or	medication	NLM:CVX:219	COVID-19, mRNA, LNP-S, PF, 3 mcg/0.2 mL dose, tris-sucrose
or	medication	NLM:CVX:228	COVID-19, mRNA, LNP-S, PF, pediatric 25 mcg/0.25 mL dose
or	medication	NLM:CVX:230	COVID-19, mRNA, LNP-S, bivalent booster, PF, 10 mcg/0.2 mL
or	medication	NLM:CVX:221	COVID-19, mRNA, LNP-S, PF, 50 mcg/0.5 mL dose
or	medication	NLM:CVX:210	COVID-19 vaccine, vector-nr, rS-ChAdOx1, PF, 0.5 mL

or	medicatio n	NLM:CVX:302	COVID-19, mRNA, LNP-S, bivalent, PF, 3 mcg/0.2 mL dose
or	medicatio n	NLM:CVX:511	COVID-19 IV Non-US Vaccine (CoronaVac, Sinovac)
or	medicatio n	NLM:RXNORM:246 8231	SARS-CoV-2 (COVID-19) vaccine, mRNA spike protein
or	procedure	UMLS:CPT:91300	Severe acute respiratory syndrome coronavirus 2 (SARS-CoV-2) (coronavirus disease [COVID-19]) vaccine, mRNA-LNP, spike protein, preservative free, 30 mcg/0.3 mL dosage, diluent reconstituted, for intramuscular use
or	procedure	UMLS:CPT:0001A	Immunization administration by intramuscular injection of severe acute respiratory syndrome coronavirus 2 (SARS-CoV-2) (coronavirus disease [COVID-19]) vaccine, mRNA-LNP, spike protein, preservative free, 30 mcg/0.3 mL dosage, diluent reconstituted; first dose
or	procedure	UMLS:CPT:0002A	Immunization administration by

			intramuscular injection of severe acute respiratory syndrome coronavirus 2 (SARS-CoV-2) (coronavirus disease [COVID-19]) vaccine, mRNA-LNP, spike protein, preservative free, 30 mcg/0.3 mL dosage, diluent reconstituted; second dose
or	procedure	UMLS:CPT:91301	Severe acute respiratory syndrome coronavirus 2 (SARS-CoV-2) (coronavirus disease [COVID-19]) vaccine, mRNA-LNP, spike protein, preservative free, 100 mcg/0.5 mL dosage, for intramuscular use
or	procedure	UMLS:CPT:0011A	Immunization administration by intramuscular injection of severe acute respiratory syndrome coronavirus 2 (SARS-CoV-2) (coronavirus disease [COVID-19]) vaccine, mRNA-LNP, spike protein, preservative free, 100 mcg/0.5 mL dosage; first dose
or	procedure	UMLS:CPT:0012A	Immunization administration by intramuscular injection of severe acute respiratory

			syndrome coronavirus 2 (SARS-CoV-2) (coronavirus disease [COVID-19]) vaccine, mRNA-LNP, spike protein, preservative free, 100 mcg/0.5 mL dosage; second dose
or	procedure	UMLS:SNOMED:840534001	Administration of SARS-CoV-2 antigen vaccine
or	medication	NLM:CVX:213	SARS-CoV-2 (COVID-19) Vaccine
or	procedure	UMLS:CPT:1036660	Immunization administration by intramuscular injection of severe acute respiratory syndrome coronavirus 2 (SARS-CoV-2) (coronavirus disease [COVID-19]) vaccine, mRNA-LNP, spike protein, preservative free, 30 mcg/0.3 mL dosage, diluent reconstituted
or	procedure	UMLS:CPT:1036663	Immunization administration by intramuscular injection of severe acute respiratory syndrome coronavirus 2 (SARS-CoV-2) (coronavirus disease [COVID-19]) vaccine, mRNA-LNP, spike protein, preservative free,

			100 mcg/0.5 mL dosage
or	procedure	UMLS:CPT:0124A	Immunization administration by intramuscular injection of severe acute respiratory syndrome coronavirus 2 (SARS-CoV-2) (coronavirus disease [COVID-19]) vaccine, mRNA-LNP, bivalent spike protein, preservative free, 30 mcg/0.3 mL dosage, tris-sucrose formulation, booster dose
or	procedure	UMLS:CPT:0004A	Immunization administration by intramuscular injection of severe acute respiratory syndrome coronavirus 2 (SARS-CoV-2) (coronavirus disease [COVID-19]) vaccine, mRNA-LNP, spike protein, preservative free, 30 mcg/0.3 mL dosage, diluent reconstituted; booster dose
or	procedure	UMLS:CPT:0003A	Immunization administration by intramuscular injection of severe acute respiratory syndrome coronavirus 2 (SARS-CoV-2) (coronavirus disease [COVID-19])

			vaccine, mRNA-LNP, spike protein, preservative free, 30 mcg/0.3 mL dosage, diluent reconstituted; third dose
or	procedure	UMLS:CPT:1037166	Immunization administration by intramuscular injection of severe acute respiratory syndrome coronavirus 2 (SARS-CoV-2) (coronavirus disease [COVID-19]) vaccine, mRNA-LNP, spike protein, preservative free, 30 mcg/0.3 mL dosage, tris-sucrose formulation
or	procedure	UMLS:CPT:0054A	Immunization administration by intramuscular injection of severe acute respiratory syndrome coronavirus 2 (SARS-CoV-2) (coronavirus disease [COVID-19]) vaccine, mRNA-LNP, spike protein, preservative free, 30 mcg/0.3 mL dosage, tris-sucrose formulation; booster dose
or	procedure	UMLS:CPT:0064A	Immunization administration by intramuscular injection of severe acute respiratory syndrome coronavirus 2

			(SARS-CoV-2) (coronavirus disease [COVID-19]) vaccine, mRNA-LNP, spike protein, preservative free, 50 mcg/0.25 mL dosage, booster dose
or	procedure	UMLS:CPT:90480	Immunization administration by intramuscular injection of severe acute respiratory syndrome coronavirus 2 (SARS-CoV-2) (coronavirus disease [COVID-19]) vaccine, single dose
or	procedure	UMLS:CPT:1037171	Immunization administration by intramuscular injection of severe acute respiratory syndrome coronavirus 2 (SARS-CoV-2) (coronavirus disease [COVID-19]) vaccine, mRNA-LNP, spike protein, preservative free, 10 mcg/0.2 mL dosage, diluent reconstituted, tris-sucrose formulation
or	procedure	UMLS:CPT:0071A	Immunization administration by intramuscular injection of severe acute respiratory syndrome coronavirus 2 (SARS-CoV-2) (coronavirus

			disease [COVID-19]) vaccine, mRNA-LNP, spike protein, preservative free, 10 mcg/0.2 mL dosage, diluent reconstituted, tris-sucrose formulation; first dose
or	procedure	UMLS:CPT:0072A	Immunization administration by intramuscular injection of severe acute respiratory syndrome coronavirus 2 (SARS-CoV-2) (coronavirus disease [COVID-19]) vaccine, mRNA-LNP, spike protein, preservative free, 10 mcg/0.2 mL dosage, diluent reconstituted, tris-sucrose formulation; second dose
or	medication	NLM:RXNORM:2610319	SARS-CoV-2 (COVID-19) vaccine, mRNA-BNT162b2 0.05 MG/ML / SARS-CoV-2 (COVID-19) vaccine, mRNA-BNT162b2 OMICRON (BA.4/BA.5) 0.05 MG/ML Injectable Suspension
or	procedure	UMLS:CPT:91313	Severe acute respiratory syndrome coronavirus 2 (SARS-CoV-2) (coronavirus disease [COVID-19]) vaccine, mRNA-LNP, spike protein, bivalent, preservative free, 50

			mcg/0.5 mL dosage, for intramuscular use
or	procedure	UMLS:CPT:0134A	Immunization administration by intramuscular injection of severe acute respiratory syndrome coronavirus 2 (SARS-CoV-2) (coronavirus disease [COVID-19]) vaccine, mRNA-LNP, spike protein, bivalent, preservative free, 50 mcg/0.5 mL dosage, booster dose
or	procedure	UMLS:CPT:1037175	Immunization administration by intramuscular injection of severe acute respiratory syndrome coronavirus 2 (SARS-CoV-2) (coronavirus disease [COVID-19]) vaccine, DNA, spike protein, adenovirus type 26 (Ad26) vector, preservative free, 5x1010 viral particles/0.5 mL dosage
or	medication	NLM:RXNORM:2610347	0.3 ML SARS-CoV-2 (COVID-19) vaccine, mRNA-BNT162b2 0.05 MG/ML / SARS-CoV-2 (COVID-19) vaccine, mRNA-BNT162b2 OMICRON (BA.4/BA.5) -1

			MG/ML Injection
or	procedure	UMLS:CPT:1037228	Immunization administration by intramuscular injection of severe acute respiratory syndrome coronavirus 2 (SARS-CoV-2) (coronavirus disease [COVID-19]) vaccine, mRNA-LNP, spike protein, preservative free, 3 mcg/0.2 mL dosage, diluent reconstituted, tris-sucrose formulation
or	procedure	UMLS:CPT:0013A	Immunization administration by intramuscular injection of severe acute respiratory syndrome coronavirus 2 (SARS-CoV-2) (coronavirus disease [COVID-19]) vaccine, mRNA-LNP, spike protein, preservative free, 100 mcg/0.5 mL dosage; third dose
or	procedure	UMLS:CPT:0081A	Immunization administration by intramuscular injection of severe acute respiratory syndrome coronavirus 2 (SARS-CoV-2) (coronavirus disease [COVID-19]) vaccine, mRNA-LNP, spike

			protein, preservative free, 3 mcg/0.2 mL dosage, diluent reconstituted, tris-sucrose formulation; first dose
or	procedure	UMLS:CPT:0082A	Immunization administration by intramuscular injection of severe acute respiratory syndrome coronavirus 2 (SARS-CoV-2) (coronavirus disease [COVID-19]) vaccine, mRNA-LNP, spike protein, preservative free, 3 mcg/0.2 mL dosage, diluent reconstituted, tris-sucrose formulation; second dose
or	medicatio n	NLM:RXNORM:261 0328	SARS-CoV-2 (COVID-19) vaccine, mRNA-1273 0.05 MG/ML / SARS-CoV-2 (COVID-19) vaccine, mRNA-1273 OMICRON (BA.4/BA.5) 0.05 MG/ML Injectable Suspension
or	procedure	UMLS:CPT:0154A	Immunization administration by intramuscular injection of severe acute respiratory syndrome coronavirus 2 (SARS-CoV-2) (coronavirus disease [COVID-19]) vaccine, mRNA-LNP, bivalent spike protein,

			preservative free, 10 mcg/0.2 mL dosage, diluent reconstituted, tris-sucrose formulation, booster dose
or	procedure	UMLS:CPT:0053A	Immunization administration by intramuscular injection of severe acute respiratory syndrome coronavirus 2 (SARS-CoV-2) (coronavirus disease [COVID-19]) vaccine, mRNA-LNP, spike protein, preservative free, 30 mcg/0.3 mL dosage, tris-sucrose formulation; third dose
or	procedure	UMLS:CPT:1037332	Immunization administration by intramuscular injection of severe acute respiratory syndrome coronavirus 2 (SARS-CoV-2) (coronavirus disease [COVID-19]) vaccine, mRNA-LNP, spike protein, preservative free, 25 mcg/0.25 mL dosage
or	procedure	UMLS:CPT:0052A	Immunization administration by intramuscular injection of severe acute respiratory syndrome coronavirus 2 (SARS-CoV-2) (coronavirus

			disease [COVID-19]) vaccine, mRNA-LNP, spike protein, preservative free, 30 mcg/0.3 mL dosage, tris-sucrose formulation; second dose
or	procedure	UMLS:CPT:0111A	Immunization administration by intramuscular injection of severe acute respiratory syndrome coronavirus 2 (SARS-CoV-2) (coronavirus disease [COVID-19]) vaccine, mRNA-LNP, spike protein, preservative free, 25 mcg/0.25 mL dosage; first dose
or	procedure	UMLS:CPT:0051A	Immunization administration by intramuscular injection of severe acute respiratory syndrome coronavirus 2 (SARS-CoV-2) (coronavirus disease [COVID-19]) vaccine, mRNA-LNP, spike protein, preservative free, 30 mcg/0.3 mL dosage, tris-sucrose formulation; first dose
or	procedure	UMLS:CPT:91311	Severe acute respiratory syndrome coronavirus 2 (SARS-CoV-2) (coronavirus

			disease [COVID-19]) vaccine, mRNA-LNP, spike protein, preservative free, 25 mcg/0.25 mL dosage, for intramuscular use
or	procedure	UMLS:CPT:0074A	Immunization administration by intramuscular injection of severe acute respiratory syndrome coronavirus 2 (SARS-CoV-2) (coronavirus disease [COVID-19]) vaccine, mRNA-LNP, spike protein, preservative free, 10 mcg/0.2 mL dosage, diluent reconstituted, tris-sucrose formulation; booster dose
or	procedure	UMLS:CPT:0112A	Immunization administration by intramuscular injection of severe acute respiratory syndrome coronavirus 2 (SARS-CoV-2) (coronavirus disease [COVID-19]) vaccine, mRNA-LNP, spike protein, preservative free, 25 mcg/0.25 mL dosage; second dose
or	procedure	UMLS:CPT:0083A	Immunization administration by intramuscular injection of severe acute respiratory

			syndrome coronavirus 2 (SARS-CoV-2) (coronavirus disease [COVID-19]) vaccine, mRNA-LNP, spike protein, preservative free, 3 mcg/0.2 mL dosage, diluent reconstituted, tris-sucrose formulation; third dose
or	procedure	UMLS:CPT:0073A	Immunization administration by intramuscular injection of severe acute respiratory syndrome coronavirus 2 (SARS-CoV-2) (coronavirus disease [COVID-19]) vaccine, mRNA-LNP, spike protein, preservative free, 10 mcg/0.2 mL dosage, diluent reconstituted, tris-sucrose formulation; third dose
or	procedure	UMLS:CPT:0173A	Immunization administration by intramuscular injection of severe acute respiratory syndrome coronavirus 2 (SARS-CoV-2) (coronavirus disease [COVID-19]) vaccine, mRNA-LNP, bivalent spike protein, preservative free, 3 mcg/0.2 mL dosage, diluent reconstituted, tris-sucrose

			formulation, third dose
or	procedure	UMLS:CPT:0164A	Immunization administration by intramuscular injection of severe acute respiratory syndrome coronavirus 2 (SARS-CoV-2) (coronavirus disease [COVID-19]) vaccine, mRNA-LNP, spike protein, bivalent, preservative free, 10 mcg/0.2 mL dosage, booster dose
or	procedure	UMLS:CPT:1037838	Immunization administration by intramuscular injection of severe acute respiratory syndrome coronavirus 2 (SARS-CoV-2) (coronavirus disease [COVID-19]) vaccine, mRNA-LNP, spike protein, preservative free, 50 mcg/0.5 mL dosage
or	procedure	UMLS:CPT:0094A	Immunization administration by intramuscular injection of severe acute respiratory syndrome coronavirus 2 (SARS-CoV-2) (coronavirus disease [COVID-19]) vaccine, mRNA-LNP, spike protein, preservative free, 50

			mcg/0.5 mL dosage; booster dose, when administered to individuals 18 years and over
or	procedure	UMLS:CPT:0034A	Immunization administration by intramuscular injection of severe acute respiratory syndrome coronavirus 2 (SARS-CoV-2) (coronavirus disease [COVID-19]) vaccine, DNA, spike protein, adenovirus type 26 (Ad26) vector, preservative free, 5x1010 viral particles/0.5 mL dosage; booster dose
or	procedure	UMLS:CPT:0144A	Immunization administration by intramuscular injection of severe acute respiratory syndrome coronavirus 2 (SARS-CoV-2) (coronavirus disease [COVID-19]) vaccine, mRNA-LNP, spike protein, bivalent, preservative free, 25 mcg/0.25 mL dosage, booster dose
or	procedure	UMLS:CPT:0091A	Immunization administration by intramuscular injection of severe acute respiratory

			syndrome coronavirus 2 (SARS-CoV-2) (coronavirus disease [COVID-19]) vaccine, mRNA-LNP, spike protein, preservative free, 50 mcg/0.5 mL dosage; first dose, when administered to individuals 6 through 11 years
or	procedure	UMLS:CPT:0174A	Immunization administration by intramuscular injection of severe acute respiratory syndrome coronavirus 2 (SARS-CoV-2) (coronavirus disease [COVID-19]) vaccine, mRNA-LNP, bivalent spike protein, preservative free, 3 mcg/0.2 mL dosage, diluent reconstituted, tris-sucrose formulation, booster
or	procedure	UMLS:CPT:0092A	Immunization administration by intramuscular injection of severe acute respiratory syndrome coronavirus 2 (SARS-CoV-2) (coronavirus disease [COVID-19]) vaccine, mRNA-LNP, spike protein, preservative free, 50 mcg/0.5 mL dosage; second

			dose, when administered to individuals 6 through 11 years
or	procedure	UMLS:CPT:1036682	Immunization administration by intramuscular injection of severe acute respiratory syndrome coronavirus 2 (SARS-CoV-2) (coronavirus disease [COVID-19]) vaccine, recombinant spike protein nanoparticle, saponin-based adjuvant, preservative free, 5 mcg/0.5 mL dosage
or	procedure	UMLS:CPT:0041A	Immunization administration by intramuscular injection of severe acute respiratory syndrome coronavirus 2 (SARS-CoV-2) (coronavirus disease [COVID-19]) vaccine, recombinant spike protein nanoparticle, saponin-based adjuvant, preservative free, 5 mcg/0.5 mL dosage; first dose
or	procedure	UMLS:CPT:0113A	Immunization administration by intramuscular injection of severe acute respiratory syndrome coronavirus 2

			(SARS-CoV-2) (coronavirus disease [COVID-19]) vaccine, mRNA-LNP, spike protein, preservative free, 25 mcg/0.25 mL dosage; third dose
or	procedure	UMLS:CPT:0042A	Immunization administration by intramuscular injection of severe acute respiratory syndrome coronavirus 2 (SARS-CoV-2) (coronavirus disease [COVID-19]) vaccine, recombinant spike protein nanoparticle, saponin-based adjuvant, preservative free, 5 mcg/0.5 mL dosage; second dose
or	procedure	UMLS:CPT:0093A	Immunization administration by intramuscular injection of severe acute respiratory syndrome coronavirus 2 (SARS-CoV-2) (coronavirus disease [COVID-19]) vaccine, mRNA-LNP, spike protein, preservative free, 50 mcg/0.5 mL dosage; third dose, when administered to individuals 6 through 11 years

	or	procedure	UMLS:CPT:1036666	Immunization administration by intramuscular injection of severe acute respiratory syndrome coronavirus 2 (SARS-CoV-2) (coronavirus disease [COVID-19]) vaccine, DNA, spike protein, chimpanzee adenovirus Oxford 1 (ChAdOx1) vector, preservative free, 5x1010 viral particles/0.5 mL dosage
	or	procedure	UMLS:CPT:0044A	Immunization administration by intramuscular injection of severe acute respiratory syndrome coronavirus 2 (SARS-CoV-2) (coronavirus disease [COVID-19]) vaccine, recombinant spike protein nanoparticle, saponin-based adjuvant, preservative free, 5 mcg/0.5mL dosage; booster
date constraint		The terms in this group occurred between Dec 1, 2019 and Dec 31, 2023		
**Group 2**				
**Group 2A COVID-19 negative**				
must have		visit	TNX:Visit	Visit (Data Source: TriNetX)

cannot have		diagnosis	UMLS:ICD10CM:U07.1	COVID-19
	or	laboratory	UMLS:LNC:95406-5	SARS-CoV-2 (COVID-19) RNA [Presence] in Nose by NAA with probe detection (labResult: Positive)
	or	laboratory	UMLS:LNC:94845-5	SARS-CoV-2 (COVID-19) RNA [Presence] in Saliva (oral fluid) by NAA with probe detection (labResult: Positive)
	or	laboratory	UMLS:LNC:95608-6	SARS-CoV-2 (COVID-19) RNA [Presence] in Respiratory specimen by NAA with non-probe detection (labResult: Positive)
	or	laboratory	UMLS:LNC:94759-8	SARS-CoV-2 (COVID-19) RNA [Presence] in Nasopharynx by NAA with probe detection (labResult: Positive)
	or	laboratory	UMLS:LNC:94565-9	SARS-CoV-2 (COVID-19) RNA [Presence] in Nasopharynx by NAA with non-probe detection (labResult: Positive)
	or	laboratory	UMLS:LNC:94309-2	SARS-CoV-2 (COVID-19) RNA [Presence] in Specimen by NAA with probe detection

			(labResult: Positive)
or	laboratory	UMLS:LNC:94500-6	SARS-CoV-2 (COVID-19) RNA [Presence] in Respiratory specimen by NAA with probe detection (labResult: Positive)
or	diagnosis	UMLS:ICD10CM:U07.2	COVID-19, virus not identified (WHO)
or	diagnosis	UMLS:ICD10CM:J12.82	Pneumonia due to coronavirus disease 2019
date constraint	The terms in this group occurred at any time		
event relationship	Any instance of Hx of hypothyroidism and thyroid ca occurred on or before any instance of COVID-19 negative		
**Group 2B Hx of hypothyroidism and thyroid ca**			
cannot have	diagnosis	UMLS:ICD10CM:E03	Other hypothyroidism
or	diagnosis	UMLS:ICD10CM:E03.9	Hypothyroidism, unspecified
or	diagnosis	UMLS:ICD10CM:E03.8	Other specified hypothyroidism
or	diagnosis	UMLS:ICD10CM:E03.3	Postinfectious hypothyroidism
or	diagnosis	UMLS:ICD10CM:C73	Malignant neoplasm of thyroid gland
or	diagnosis	UMLS:ICD10CM:Z85.850	Personal history of malignant neoplasm of thyroid

Group 3				
**hypothyroidism**				
must have	any of	diagnosis	UMLS:ICD10CM:E05	Thyrotoxicosis [hyperthyroidism]
		diagnosis	UMLS:ICD10CM:E06	Thyroiditis
date constraint		The terms in this group occurred at any time		

Query Criteria for Cohort non-COVID with hypothyroidism

This query was run on the network Global Collaborative Network with 147 HCO(s) queried and 147 HCO(s) responded. A total of 127 provider(s) responded with patients. The final cohort included 1,560,968 patients who matched the query criteria listed in the table below.

Ungrouped terms				
must have		demographics	Age	Age (at least 18 years (most recent occurrence))
	and any of	demographics	UMLS:HL7V3.0:Gender:M	Male
		demographics	UMLS:HL7V3.0:Gender:F	Female
**Group 1**				
**COVID-19 negative, no vaccination**				
must have		visit	TNX:Visit	Visit
cannot		medicatio	NLM:CVX:208	COVID-19, mRNA, LNP-S,

have		n		PF, 30 mcg/0.3 mL dose
	or	medicatio n	NLM:CVX:207	COVID-19, mRNA, LNP-S, PF, 100 mcg/0.5mL dose or 50 mcg/0.25mL dose
	or	medicatio n	NLM:CVX:212	COVID-19 vaccine, vector-nr, rS-Ad26, PF, 0.5 mL
	or	medicatio n	NLM:RXNORM:OM OP5042939	COVID-19 vaccine
	or	medicatio n	NLM:CVX:300	COVID-19, mRNA, LNP-S, bivalent, PF, 30 mcg/0.3 mL dose
	or	medicatio n	NLM:CVX:217	COVID-19, mRNA, LNP-S, PF, 30 mcg/0.3 mL dose, tris-sucrose
	or	medicatio n	NLM:CVX:229	COVID-19, mRNA, LNP-S, bivalent, PF, 50 mcg/0.5 mL or 25mcg/0.25 mL dose
	or	medicatio n	NLM:CVX:218	COVID-19, mRNA, LNP-S, PF, 10 mcg/0.2 mL dose, tris-sucrose
	or	medicatio n	NLM:CVX:520	COVID-19 mRNA, bivalent, original/Omicron BA.1, Non-US Vaccine Product, Pfizer-BioNTech
	or	medicatio n	NLM:CVX:519	COVID-19 mRNA, bivalent, original/Omicron BA.1, Non-US Vaccine (Spikevax Bivalent), Moderna

or	medicatio n	NLM:CVX:301	COVID-19, mRNA, LNP-S, bivalent, PF, 10 mcg/0.2 mL dose
or	medicatio n	NLM:CVX:219	COVID-19, mRNA, LNP-S, PF, 3 mcg/0.2 mL dose, tris-sucrose
or	medicatio n	NLM:CVX:228	COVID-19, mRNA, LNP-S, PF, pediatric 25 mcg/0.25 mL dose
or	medicatio n	NLM:CVX:230	COVID-19, mRNA, LNP-S, bivalent booster, PF, 10 mcg/0.2 mL
or	medicatio n	NLM:CVX:221	COVID-19, mRNA, LNP-S, PF, 50 mcg/0.5 mL dose
or	medicatio n	NLM:CVX:210	COVID-19 vaccine, vector-nr, rS-ChAdOx1, PF, 0.5 mL
or	medicatio n	NLM:CVX:302	COVID-19, mRNA, LNP-S, bivalent, PF, 3 mcg/0.2 mL dose
or	medicatio n	NLM:CVX:511	COVID-19 IV Non-US Vaccine (CoronaVac, Sinovac)
or	medicatio n	NLM:RXNORM:2468231	SARS-CoV-2 (COVID-19) vaccine, mRNA spike protein
or	procedure	UMLS:CPT:91300	Severe acute respiratory syndrome coronavirus 2 (SARS-CoV-2) (coronavirus disease [COVID-19])

			vaccine, mRNA-LNP, spike protein, preservative free, 30 mcg/0.3 mL dosage, diluent reconstituted, for intramuscular use
or	procedure	UMLS:CPT:0001A	Immunization administration by intramuscular injection of severe acute respiratory syndrome coronavirus 2 (SARS-CoV-2) (coronavirus disease [COVID-19]) vaccine, mRNA-LNP, spike protein, preservative free, 30 mcg/0.3 mL dosage, diluent reconstituted; first dose
or	procedure	UMLS:CPT:0002A	Immunization administration by intramuscular injection of severe acute respiratory syndrome coronavirus 2 (SARS-CoV-2) (coronavirus disease [COVID-19]) vaccine, mRNA-LNP, spike protein, preservative free, 30 mcg/0.3 mL dosage, diluent reconstituted; second dose
or	procedure	UMLS:CPT:91301	Severe acute respiratory syndrome coronavirus 2 (SARS-CoV-2) (coronavirus disease [COVID-19]) vaccine, mRNA-LNP, spike

			protein, preservative free, 100 mcg/0.5 mL dosage, for intramuscular use
or	procedure	UMLS:CPT:0011A	Immunization administration by intramuscular injection of severe acute respiratory syndrome coronavirus 2 (SARS-CoV-2) (coronavirus disease [COVID-19]) vaccine, mRNA-LNP, spike protein, preservative free, 100 mcg/0.5 mL dosage; first dose
or	procedure	UMLS:CPT:0012A	Immunization administration by intramuscular injection of severe acute respiratory syndrome coronavirus 2 (SARS-CoV-2) (coronavirus disease [COVID-19]) vaccine, mRNA-LNP, spike protein, preservative free, 100 mcg/0.5 mL dosage; second dose
or	procedure	UMLS:SNOMED:840534001	Administration of SARS-CoV-2 antigen vaccine
or	medication	NLM:CVX:213	SARS-CoV-2 (COVID-19) Vaccine
or	procedure	UMLS:CPT:1036660	Immunization administration by

			intramuscular injection of severe acute respiratory syndrome coronavirus 2 (SARS-CoV-2) (coronavirus disease [COVID-19]) vaccine, mRNA-LNP, spike protein, preservative free, 30 mcg/0.3 mL dosage, diluent reconstituted
or	procedure	UMLS:CPT:1036663	Immunization administration by intramuscular injection of severe acute respiratory syndrome coronavirus 2 (SARS-CoV-2) (coronavirus disease [COVID-19]) vaccine, mRNA-LNP, spike protein, preservative free, 100 mcg/0.5 mL dosage
or	procedure	UMLS:CPT:0124A	Immunization administration by intramuscular injection of severe acute respiratory syndrome coronavirus 2 (SARS-CoV-2) (coronavirus disease [COVID-19]) vaccine, mRNA-LNP, bivalent spike protein, preservative free, 30 mcg/0.3 mL dosage, tris-sucrose formulation, booster dose

or	procedure	UMLS:CPT:0004A	Immunization administration by intramuscular injection of severe acute respiratory syndrome coronavirus 2 (SARS-CoV-2) (coronavirus disease [COVID-19]) vaccine, mRNA-LNP, spike protein, preservative free, 30 mcg/0.3 mL dosage, diluent reconstituted; booster dose
or	procedure	UMLS:CPT:0003A	Immunization administration by intramuscular injection of severe acute respiratory syndrome coronavirus 2 (SARS-CoV-2) (coronavirus disease [COVID-19]) vaccine, mRNA-LNP, spike protein, preservative free, 30 mcg/0.3 mL dosage, diluent reconstituted; third dose
or	procedure	UMLS:CPT:1037166	Immunization administration by intramuscular injection of severe acute respiratory syndrome coronavirus 2 (SARS-CoV-2) (coronavirus disease [COVID-19]) vaccine, mRNA-LNP, spike protein, preservative free, 30 mcg/0.3 mL dosage, tris-

			sucrose formulation
or	procedure	UMLS:CPT:0054A	Immunization administration by intramuscular injection of severe acute respiratory syndrome coronavirus 2 (SARS-CoV-2) (coronavirus disease [COVID-19]) vaccine, mRNA-LNP, spike protein, preservative free, 30 mcg/0.3 mL dosage, tris-sucrose formulation; booster dose
or	procedure	UMLS:CPT:0064A	Immunization administration by intramuscular injection of severe acute respiratory syndrome coronavirus 2 (SARS-CoV-2) (coronavirus disease [COVID-19]) vaccine, mRNA-LNP, spike protein, preservative free, 50 mcg/0.25 mL dosage, booster dose
or	procedure	UMLS:CPT:90480	Immunization administration by intramuscular injection of severe acute respiratory syndrome coronavirus 2 (SARS-CoV-2) (coronavirus disease [COVID-19])

			vaccine, single dose
or	procedure	UMLS:CPT:1037171	Immunization administration by intramuscular injection of severe acute respiratory syndrome coronavirus 2 (SARS-CoV-2) (coronavirus disease [COVID-19]) vaccine, mRNA-LNP, spike protein, preservative free, 10 mcg/0.2 mL dosage, diluent reconstituted, tris-sucrose formulation
or	procedure	UMLS:CPT:0071A	Immunization administration by intramuscular injection of severe acute respiratory syndrome coronavirus 2 (SARS-CoV-2) (coronavirus disease [COVID-19]) vaccine, mRNA-LNP, spike protein, preservative free, 10 mcg/0.2 mL dosage, diluent reconstituted, tris-sucrose formulation; first dose
or	procedure	UMLS:CPT:0072A	Immunization administration by intramuscular injection of severe acute respiratory syndrome coronavirus 2 (SARS-CoV-2) (coronavirus disease [COVID-19])

			vaccine, mRNA-LNP, spike protein, preservative free, 10 mcg/0.2 mL dosage, diluent reconstituted, tris-sucrose formulation; second dose
or	medication	NLM:RXNORM:2610319	SARS-CoV-2 (COVID-19) vaccine, mRNA-BNT162b2 0.05 MG/ML / SARS-CoV-2 (COVID-19) vaccine, mRNA-BNT162b2 OMICRON (BA.4/BA.5) 0.05 MG/ML Injectable Suspension
or	procedure	UMLS:CPT:91313	Severe acute respiratory syndrome coronavirus 2 (SARS-CoV-2) (coronavirus disease [COVID-19]) vaccine, mRNA-LNP, spike protein, bivalent, preservative free, 50 mcg/0.5 mL dosage, for intramuscular use
or	procedure	UMLS:CPT:0134A	Immunization administration by intramuscular injection of severe acute respiratory syndrome coronavirus 2 (SARS-CoV-2) (coronavirus disease [COVID-19]) vaccine, mRNA-LNP, spike protein, bivalent, preservative free, 50 mcg/0.5 mL dosage, booster

			dose
or	procedure	UMLS:CPT:1037175	Immunization administration by intramuscular injection of severe acute respiratory syndrome coronavirus 2 (SARS-CoV-2) (coronavirus disease [COVID-19]) vaccine, DNA, spike protein, adenovirus type 26 (Ad26) vector, preservative free, 5x1010 viral particles/0.5 mL dosage
or	medication	NLM:RXNORM:2610347	0.3 ML SARS-CoV-2 (COVID-19) vaccine, mRNA-BNT162b2 0.05 MG/ML / SARS-CoV-2 (COVID-19) vaccine, mRNA-BNT162b2 OMICRON (BA.4/BA.5) -1 MG/ML Injection
or	procedure	UMLS:CPT:1037228	Immunization administration by intramuscular injection of severe acute respiratory syndrome coronavirus 2 (SARS-CoV-2) (coronavirus disease [COVID-19]) vaccine, mRNA-LNP, spike protein, preservative free, 3 mcg/0.2 mL dosage, diluent reconstituted, tris-sucrose

			formulation
or	procedure	UMLS:CPT:0013A	Immunization administration by intramuscular injection of severe acute respiratory syndrome coronavirus 2 (SARS-CoV-2) (coronavirus disease [COVID-19]) vaccine, mRNA-LNP, spike protein, preservative free, 100 mcg/0.5 mL dosage; third dose
or	procedure	UMLS:CPT:0081A	Immunization administration by intramuscular injection of severe acute respiratory syndrome coronavirus 2 (SARS-CoV-2) (coronavirus disease [COVID-19]) vaccine, mRNA-LNP, spike protein, preservative free, 3 mcg/0.2 mL dosage, diluent reconstituted, tris-sucrose formulation; first dose
or	procedure	UMLS:CPT:0082A	Immunization administration by intramuscular injection of severe acute respiratory syndrome coronavirus 2 (SARS-CoV-2) (coronavirus disease [COVID-19]) vaccine, mRNA-LNP, spike

			protein, preservative free, 3 mcg/0.2 mL dosage, diluent reconstituted, tris-sucrose formulation; second dose
or	medicatio n	NLM:RXNORM:261 0328	SARS-CoV-2 (COVID-19) vaccine, mRNA-1273 0.05 MG/ML / SARS-CoV-2 (COVID-19) vaccine, mRNA-1273 OMICRON (BA.4/BA.5) 0.05 MG/ML Injectable Suspension
or	procedure	UMLS:CPT:0154A	Immunization administration by intramuscular injection of severe acute respiratory syndrome coronavirus 2 (SARS-CoV-2) (coronavirus disease [COVID-19]) vaccine, mRNA-LNP, bivalent spike protein, preservative free, 10 mcg/0.2 mL dosage, diluent reconstituted, tris-sucrose formulation, booster dose
or	procedure	UMLS:CPT:0053A	Immunization administration by intramuscular injection of severe acute respiratory syndrome coronavirus 2 (SARS-CoV-2) (coronavirus disease [COVID-19]) vaccine, mRNA-LNP, spike

			protein, preservative free, 30 mcg/0.3 mL dosage, tris-sucrose formulation; third dose
or	procedure	UMLS:CPT:1037332	Immunization administration by intramuscular injection of severe acute respiratory syndrome coronavirus 2 (SARS-CoV-2) (coronavirus disease [COVID-19]) vaccine, mRNA-LNP, spike protein, preservative free, 25 mcg/0.25 mL dosage
or	procedure	UMLS:CPT:0052A	Immunization administration by intramuscular injection of severe acute respiratory syndrome coronavirus 2 (SARS-CoV-2) (coronavirus disease [COVID-19]) vaccine, mRNA-LNP, spike protein, preservative free, 30 mcg/0.3 mL dosage, tris-sucrose formulation; second dose
or	procedure	UMLS:CPT:0111A	Immunization administration by intramuscular injection of severe acute respiratory syndrome coronavirus 2 (SARS-CoV-2) (coronavirus

			disease [COVID-19]) vaccine, mRNA-LNP, spike protein, preservative free, 25 mcg/0.25 mL dosage; first dose
or	procedure	UMLS:CPT:0051A	Immunization administration by intramuscular injection of severe acute respiratory syndrome coronavirus 2 (SARS-CoV-2) (coronavirus disease [COVID-19]) vaccine, mRNA-LNP, spike protein, preservative free, 30 mcg/0.3 mL dosage, tris-sucrose formulation; first dose
or	procedure	UMLS:CPT:91311	Severe acute respiratory syndrome coronavirus 2 (SARS-CoV-2) (coronavirus disease [COVID-19]) vaccine, mRNA-LNP, spike protein, preservative free, 25 mcg/0.25 mL dosage, for intramuscular use
or	procedure	UMLS:CPT:0074A	Immunization administration by intramuscular injection of severe acute respiratory syndrome coronavirus 2 (SARS-CoV-2) (coronavirus disease [COVID-19])

			vaccine, mRNA-LNP, spike protein, preservative free, 10 mcg/0.2 mL dosage, diluent reconstituted, tris-sucrose formulation; booster dose
or	procedure	UMLS:CPT:0112A	Immunization administration by intramuscular injection of severe acute respiratory syndrome coronavirus 2 (SARS-CoV-2) (coronavirus disease [COVID-19]) vaccine, mRNA-LNP, spike protein, preservative free, 25 mcg/0.25 mL dosage; second dose
or	procedure	UMLS:CPT:0083A	Immunization administration by intramuscular injection of severe acute respiratory syndrome coronavirus 2 (SARS-CoV-2) (coronavirus disease [COVID-19]) vaccine, mRNA-LNP, spike protein, preservative free, 3 mcg/0.2 mL dosage, diluent reconstituted, tris-sucrose formulation; third dose
or	procedure	UMLS:CPT:0073A	Immunization administration by intramuscular injection of severe acute respiratory

			syndrome coronavirus 2 (SARS-CoV-2) (coronavirus disease [COVID-19]) vaccine, mRNA-LNP, spike protein, preservative free, 10 mcg/0.2 mL dosage, diluent reconstituted, tris-sucrose formulation; third dose
or	procedure	UMLS:CPT:0173A	Immunization administration by intramuscular injection of severe acute respiratory syndrome coronavirus 2 (SARS-CoV-2) (coronavirus disease [COVID-19]) vaccine, mRNA-LNP, bivalent spike protein, preservative free, 3 mcg/0.2 mL dosage, diluent reconstituted, tris-sucrose formulation, third dose
or	procedure	UMLS:CPT:0164A	Immunization administration by intramuscular injection of severe acute respiratory syndrome coronavirus 2 (SARS-CoV-2) (coronavirus disease [COVID-19]) vaccine, mRNA-LNP, spike protein, bivalent, preservative free, 10 mcg/0.2 mL dosage, booster

			dose
or	procedure	UMLS:CPT:1037838	Immunization administration by intramuscular injection of severe acute respiratory syndrome coronavirus 2 (SARS-CoV-2) (coronavirus disease [COVID-19]) vaccine, mRNA-LNP, spike protein, preservative free, 50 mcg/0.5 mL dosage
or	procedure	UMLS:CPT:0094A	Immunization administration by intramuscular injection of severe acute respiratory syndrome coronavirus 2 (SARS-CoV-2) (coronavirus disease [COVID-19]) vaccine, mRNA-LNP, spike protein, preservative free, 50 mcg/0.5 mL dosage; booster dose, when administered to individuals 18 years and over
or	procedure	UMLS:CPT:0034A	Immunization administration by intramuscular injection of severe acute respiratory syndrome coronavirus 2 (SARS-CoV-2) (coronavirus disease [COVID-19]) vaccine, DNA, spike protein,

			adenovirus type 26 (Ad26) vector, preservative free, 5x1010 viral particles/0.5 mL dosage; booster dose
or	procedure	UMLS:CPT:0144A	Immunization administration by intramuscular injection of severe acute respiratory syndrome coronavirus 2 (SARS-CoV-2) (coronavirus disease [COVID-19]) vaccine, mRNA-LNP, spike protein, bivalent, preservative free, 25 mcg/0.25 mL dosage, booster dose
or	procedure	UMLS:CPT:0091A	Immunization administration by intramuscular injection of severe acute respiratory syndrome coronavirus 2 (SARS-CoV-2) (coronavirus disease [COVID-19]) vaccine, mRNA-LNP, spike protein, preservative free, 50 mcg/0.5 mL dosage; first dose, when administered to individuals 6 through 11 years
or	procedure	UMLS:CPT:0174A	Immunization administration by intramuscular injection of

			severe acute respiratory syndrome coronavirus 2 (SARS-CoV-2) (coronavirus disease [COVID-19]) vaccine, mRNA-LNP, bivalent spike protein, preservative free, 3 mcg/0.2 mL dosage, diluent reconstituted, tris-sucrose formulation, booster
or	procedure	UMLS:CPT:0092A	Immunization administration by intramuscular injection of severe acute respiratory syndrome coronavirus 2 (SARS-CoV-2) (coronavirus disease [COVID-19]) vaccine, mRNA-LNP, spike protein, preservative free, 50 mcg/0.5 mL dosage; second dose, when administered to individuals 6 through 11 years
or	procedure	UMLS:CPT:1036682	Immunization administration by intramuscular injection of severe acute respiratory syndrome coronavirus 2 (SARS-CoV-2) (coronavirus disease [COVID-19]) vaccine, recombinant spike protein nanoparticle,

			saponin-based adjuvant, preservative free, 5 mcg/0.5 mL dosage
or	procedure	UMLS:CPT:0041A	Immunization administration by intramuscular injection of severe acute respiratory syndrome coronavirus 2 (SARS-CoV-2) (coronavirus disease [COVID-19]) vaccine, recombinant spike protein nanoparticle, saponin-based adjuvant, preservative free, 5 mcg/0.5 mL dosage; first dose
or	procedure	UMLS:CPT:0113A	Immunization administration by intramuscular injection of severe acute respiratory syndrome coronavirus 2 (SARS-CoV-2) (coronavirus disease [COVID-19]) vaccine, mRNA-LNP, spike protein, preservative free, 25 mcg/0.25 mL dosage; third dose
or	procedure	UMLS:CPT:0042A	Immunization administration by intramuscular injection of severe acute respiratory syndrome coronavirus 2 (SARS-CoV-2) (coronavirus

			disease [COVID-19]) vaccine, recombinant spike protein nanoparticle, saponin-based adjuvant, preservative free, 5 mcg/0.5 mL dosage; second dose
or	procedure	UMLS:CPT:0093A	Immunization administration by intramuscular injection of severe acute respiratory syndrome coronavirus 2 (SARS-CoV-2) (coronavirus disease [COVID-19]) vaccine, mRNA-LNP, spike protein, preservative free, 50 mcg/0.5 mL dosage; third dose, when administered to individuals 6 through 11 years
or	procedure	UMLS:CPT:1036666	Immunization administration by intramuscular injection of severe acute respiratory syndrome coronavirus 2 (SARS-CoV-2) (coronavirus disease [COVID-19]) vaccine, DNA, spike protein, chimpanzee adenovirus Oxford 1 (ChAdOx1) vector, preservative free, 5x1010 viral particles/0.5 mL dosage

	or	procedure	UMLS:CPT:0044A	Immunization administration by intramuscular injection of severe acute respiratory syndrome coronavirus 2 (SARS-CoV-2) (coronavirus disease [COVID-19]) vaccine, recombinant spike protein nanoparticle, saponin-based adjuvant, preservative free, 5 mcg/0.5mL dosage; booster
date constraint		The terms in this group occurred between Dec 1, 2019 and Dec 31, 2023		
**Group 2**				
**Group 2A COVID-19 negative**				
must have		visit	TNX:Visit	Visit (Data Source: TriNetX)
cannot have		diagnosis	UMLS:ICD10CM:U07.1	COVID-19
	or	laboratory	UMLS:LNC:95406-5	SARS-CoV-2 (COVID-19) RNA [Presence] in Nose by NAA with probe detection (labResult: Positive)
	or	laboratory	UMLS:LNC:94845-5	SARS-CoV-2 (COVID-19) RNA [Presence] in Saliva (oral fluid) by NAA with probe detection (labResult: Positive)

	or	laboratory	UMLS:LNC:95608-6	SARS-CoV-2 (COVID-19) RNA [Presence] in Respiratory specimen by NAA with non-probe detection (labResult: Positive)
	or	laboratory	UMLS:LNC:94759-8	SARS-CoV-2 (COVID-19) RNA [Presence] in Nasopharynx by NAA with probe detection (labResult: Positive)
	or	laboratory	UMLS:LNC:94565-9	SARS-CoV-2 (COVID-19) RNA [Presence] in Nasopharynx by NAA with non-probe detection (labResult: Positive)
	or	laboratory	UMLS:LNC:94309-2	SARS-CoV-2 (COVID-19) RNA [Presence] in Specimen by NAA with probe detection (labResult: Positive)
	or	laboratory	UMLS:LNC:94500-6	SARS-CoV-2 (COVID-19) RNA [Presence] in Respiratory specimen by NAA with probe detection (labResult: Positive)
	or	diagnosis	UMLS:ICD10CM:U07.2	COVID-19, virus not identified (WHO)
	or	diagnosis	UMLS:ICD10CM:J12.82	Pneumonia due to coronavirus disease 2019
date constraint	The terms in this group occurred at any time			

event relationship		Any instance of Hx of hyperthyroidism and thyroid ca occurred on or before any instance of COVID-19 negative		
**Group 2B Hx of hyperthyroidism and thyroid ca**				
cannot have		diagnosis	UMLS:ICD10CM:C73	Malignant neoplasm of thyroid gland
	or	diagnosis	UMLS:ICD10CM:Z85.850	Personal history of malignant neoplasm of thyroid
	or	diagnosis	UMLS:ICD10CM:E05	Thyrotoxicosis [hyperthyroidism]
	or	diagnosis	UMLS:ICD10CM:E05.40	Thyrotoxicosis factitia without thyrotoxic crisis or storm
	or	diagnosis	UMLS:ICD10CM:E05.80	Other thyrotoxicosis without thyrotoxic crisis or storm
	or	diagnosis	UMLS:ICD10CM:E05.30	Thyrotoxicosis from ectopic thyroid tissue without thyrotoxic crisis or storm
	or	diagnosis	UMLS:ICD10CM:E05.31	Thyrotoxicosis from ectopic thyroid tissue with thyrotoxic crisis or storm
	or	diagnosis	UMLS:ICD10CM:E05.1	Thyrotoxicosis with toxic single thyroid nodule
**Group 3**				
**hypothyroidism**				
must have	any of	diagnosis	UMLS:ICD10CM:E03.9	Hypothyroidism, unspecified

	diagnosis	UMLS:ICD10CM:E03	Other hypothyroidism
	diagnosis	UMLS:ICD10CM:E03.9	Hypothyroidism, unspecified
	diagnosis	UMLS:ICD10CM:E03.8	Other specified hypothyroidism
	diagnosis	UMLS:ICD10CM:E03.3	Postinfectious hypothyroidism
	diagnosis	UMLS:ICD10CM:E89.0	Postprocedural hypothyroidism
	diagnosis	UMLS:ICD10CM:E03.1	Congenital hypothyroidism without goiter
	diagnosis	UMLS:ICD10CM:E00	Congenital iodine-deficiency syndrome
	diagnosis	UMLS:ICD10CM:D89.89	Other specified disorders involving the immune mechanism, not elsewhere classified
date constraint	The terms in this group occurred at any time		
