# Supplementary material for: Association Between COVID-19 Infection and Thyroid Cancer Development: A Retrospective Cohort Study Using the TriNetX Database
Source: Biomedicines. 2025 Aug 8;13(8):1933. doi: 10.3390/biomedicines13081933 (PMC12383963; doi:10.3390/biomedicines13081933)
Supplement: Supplementary file 1 [file biomedicines-13-01933-s001.zip › Supplementary File S4.pdf]

Supplementary File S4. Kaplan-Meier graph of thyroid cancer risk in different subgroups of COVID-19 patients.

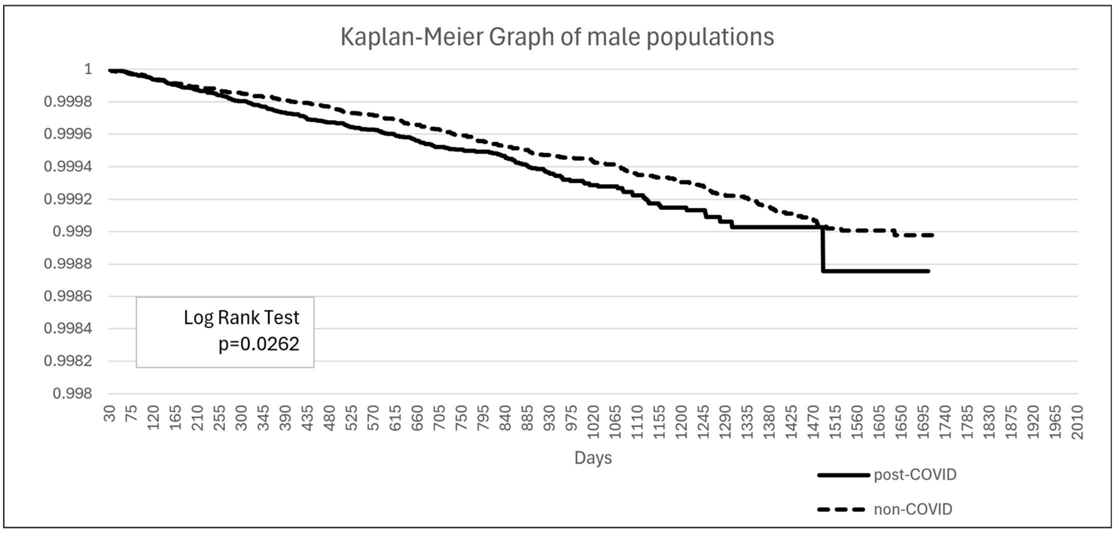

Supplementary Figure S4.1. Kaplan - Meier survival curve of thyroid cancer risk in male populations.

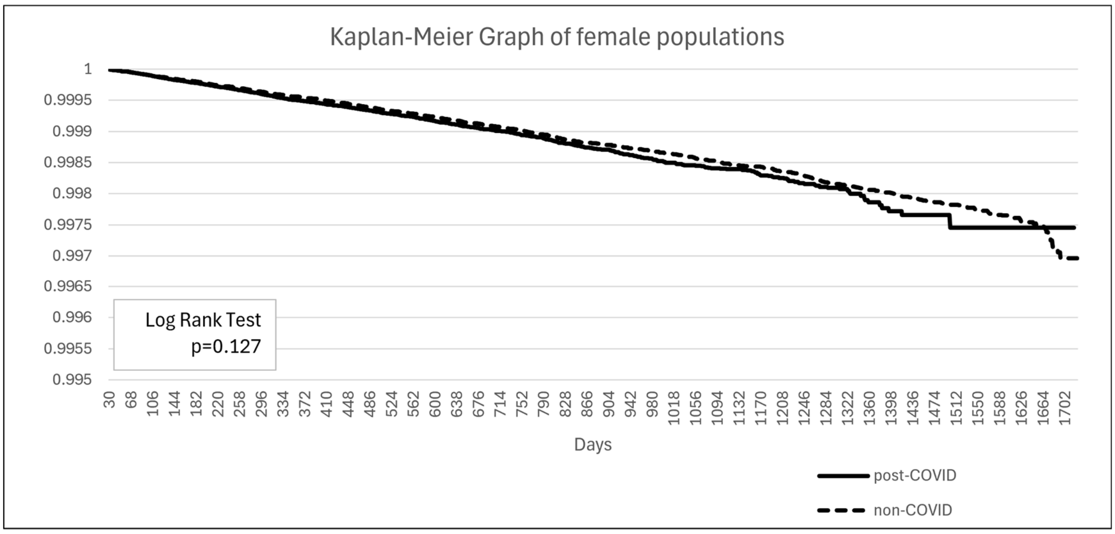

Supplementary Figure S4.2. Kaplan - Meier survival curve of thyroid cancer risk in female populations.

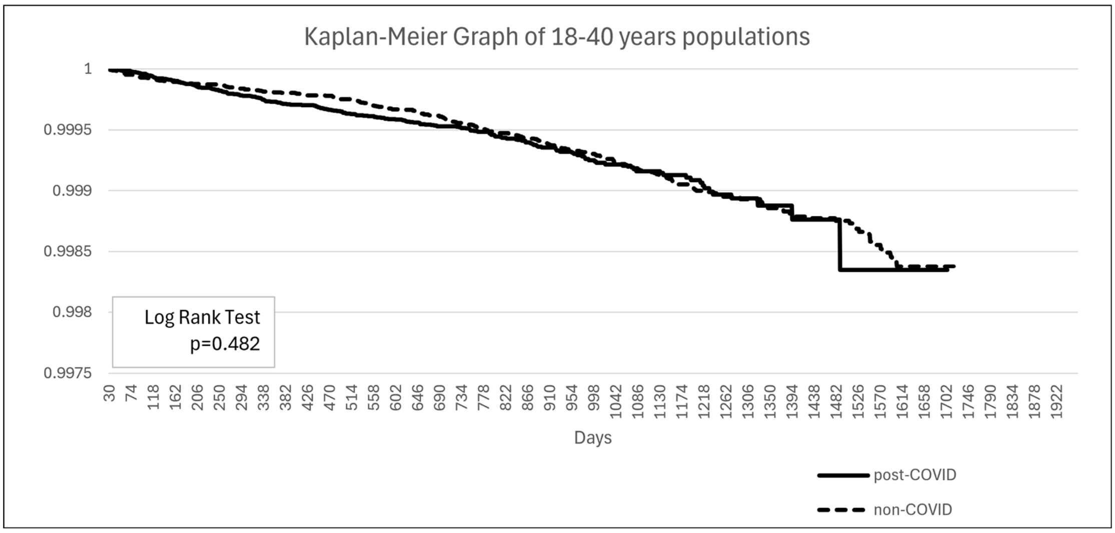

Supplementary Figure S4.3. Kaplan - Meier survival curve of thyroid cancer risk in populations aged between 18 and 40 years.

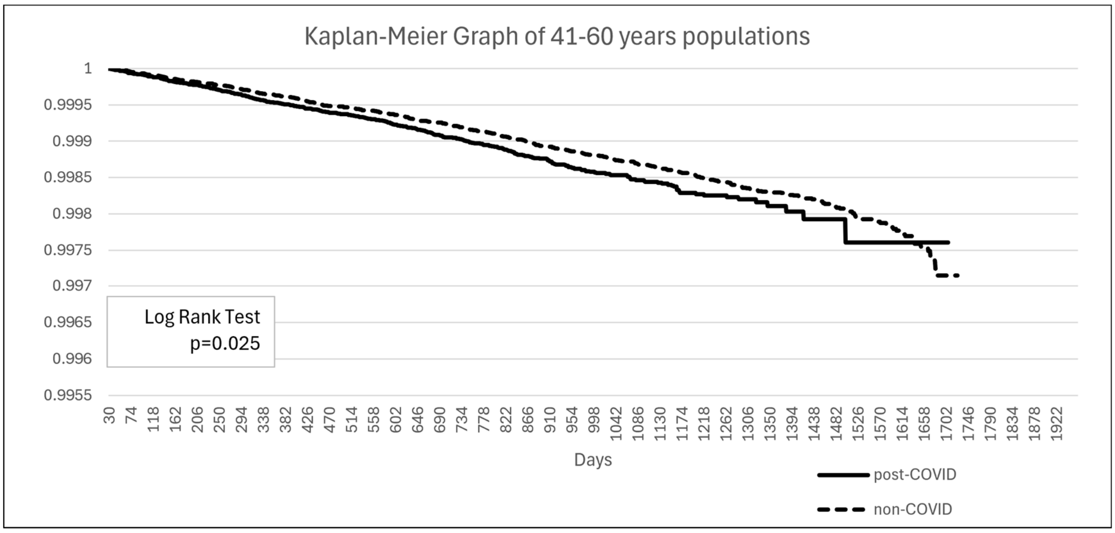

Supplementary Figure S4.4. Kaplan - Meier survival curve of thyroid cancer risk in populations aged between 41 and 60 years.

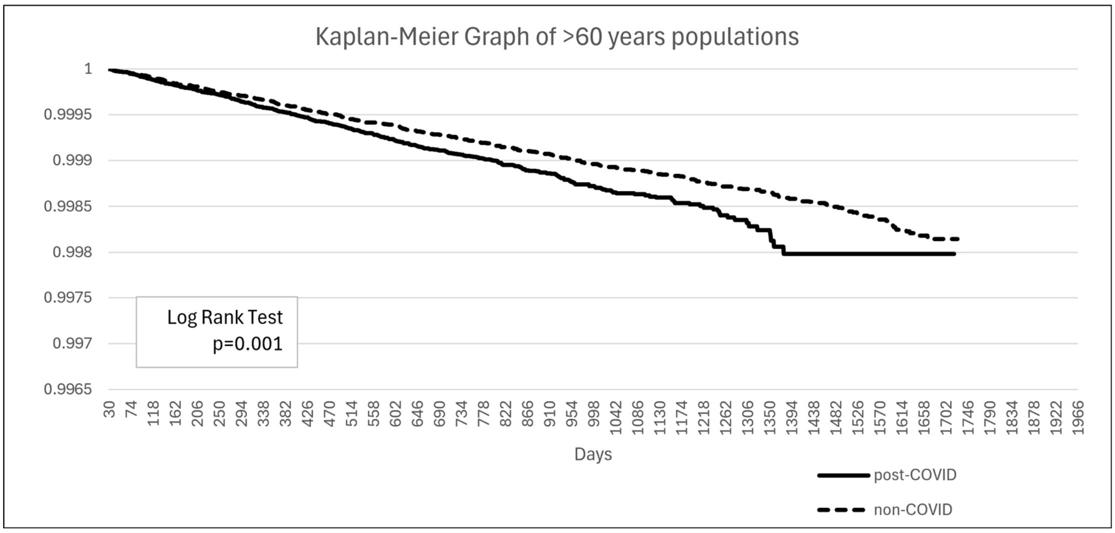

Supplementary Figure S4.5. Kaplan - Meier survival curve of thyroid cancer risk in populations aged over 60 years.

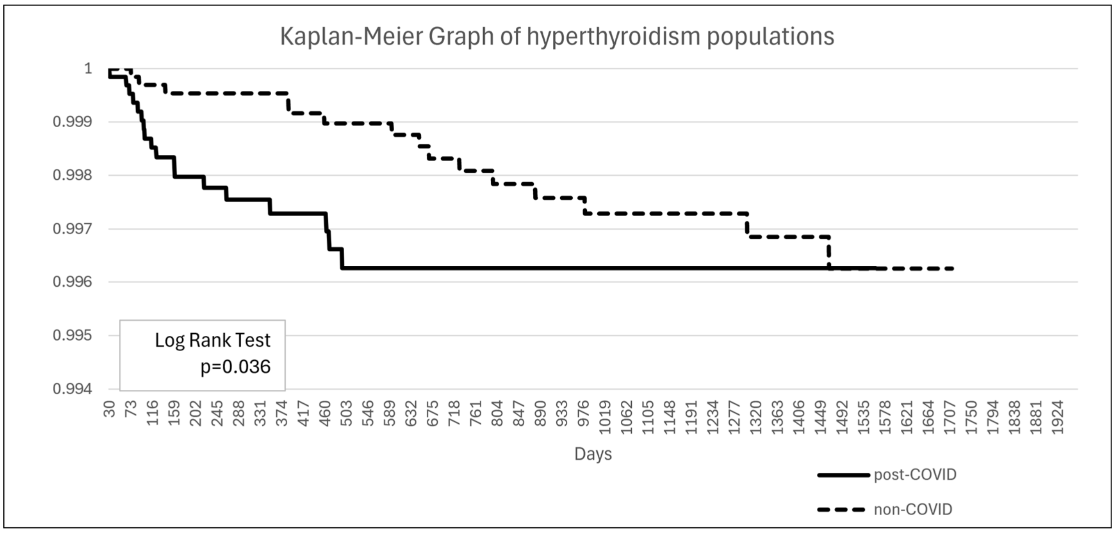

Supplementary Figure S4.6. Kaplan - Meier survival curve of thyroid cancer risk in post-COVID populations who developed hyperthyroidism vs non-COVID populations.

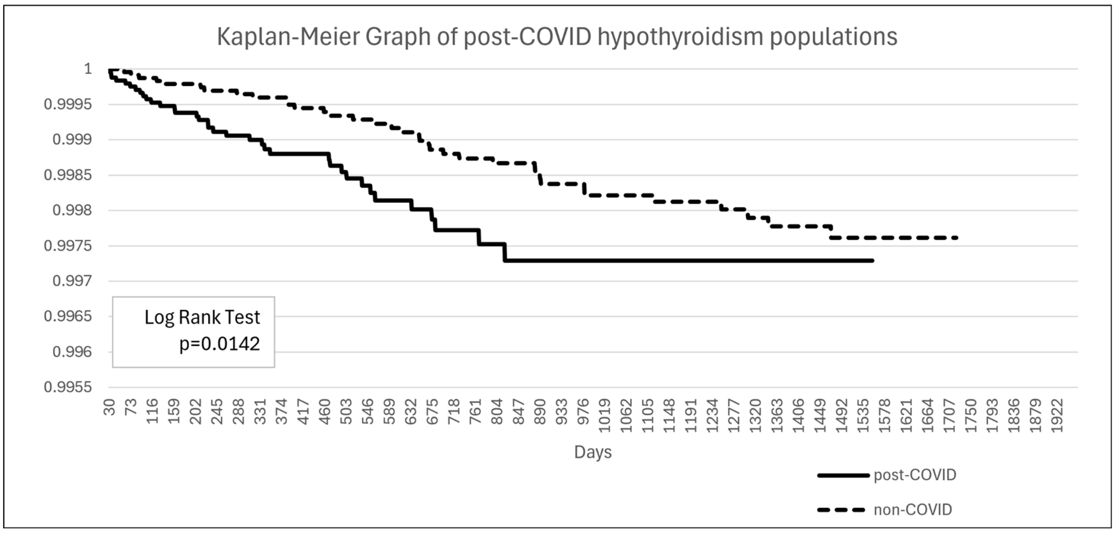

Supplementary Figure S4.7. Kaplan - Meier survival curve of thyroid cancer risk in post-COVID populations who developed hypothyroidism vs non-COVID populations.

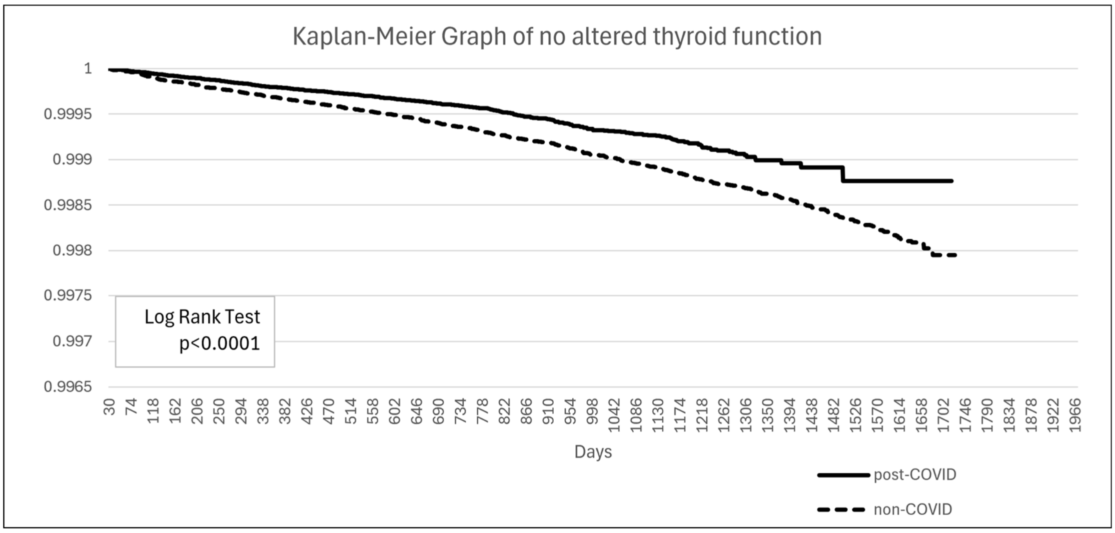

Supplementary Figure S4.8. Kaplan - Meier survival curve of thyroid cancer risk in post-COVID populations who had no thyroid function alteration vs non-COVID populations.

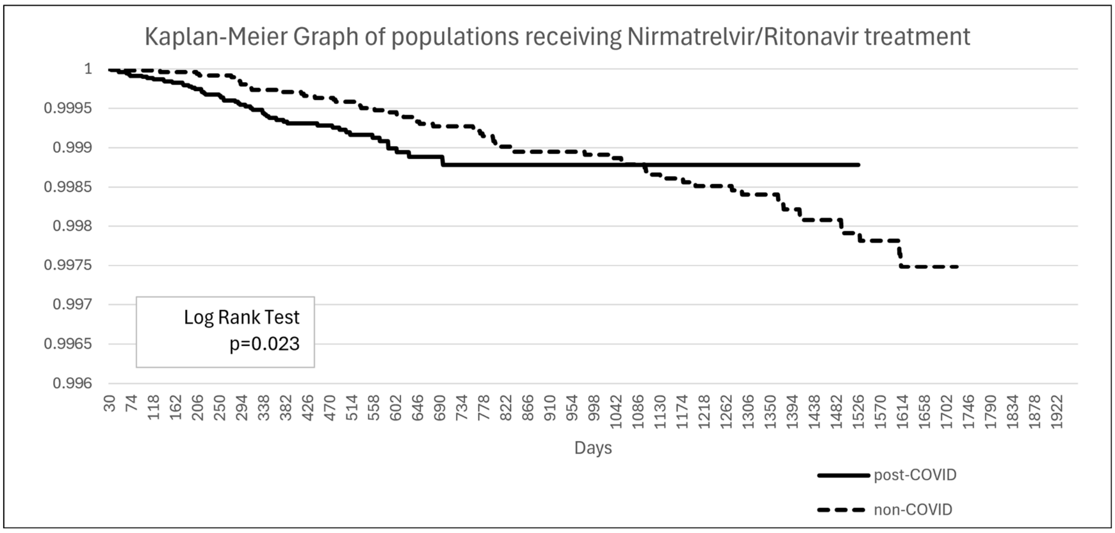

Supplementary Figure S4.9. Kaplan - Meier survival curve of thyroid cancer risk in post-COVID populations who received Nirmatrelvir/Ritonavir treatment vs non-COVID populations.

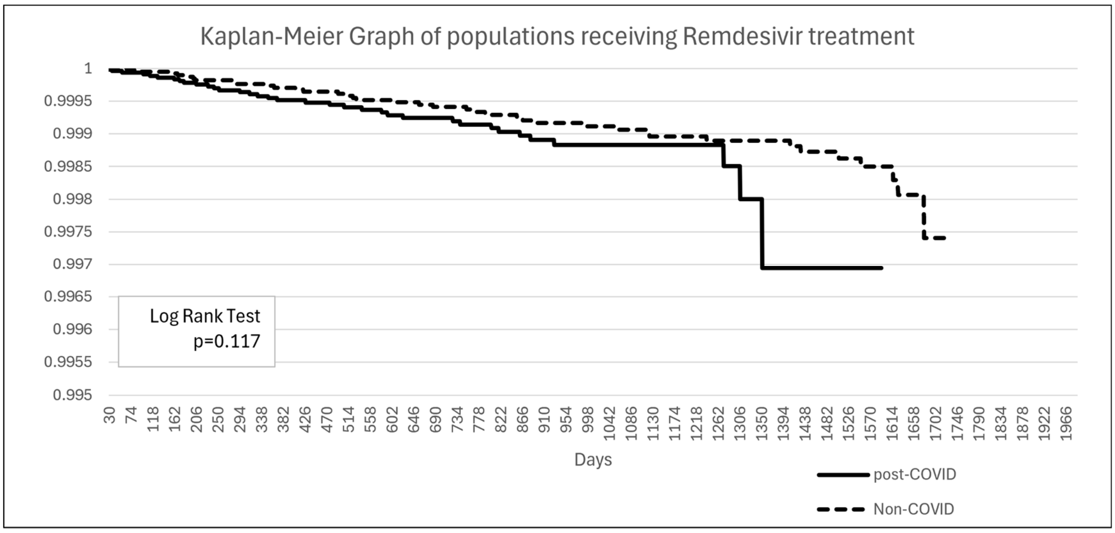

Supplementary Figure S4.10. Kaplan - Meier survival curve of thyroid cancer risk in post-COVID populations who received Remdesivir treatment vs non-COVID populations.

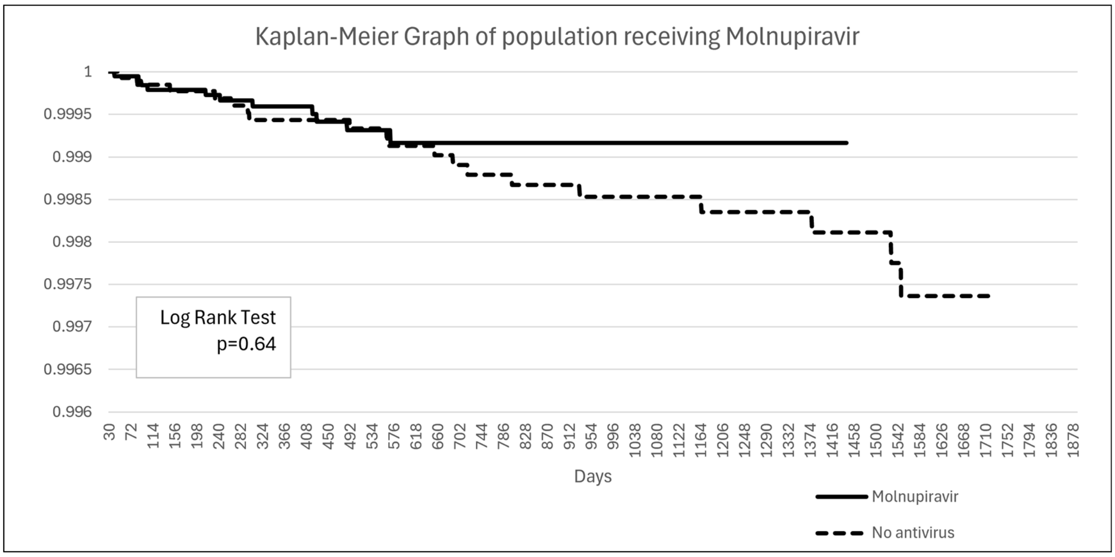

Supplementary Figure S4.11. Kaplan - Meier survival curve of thyroid cancer risk in post-COVID populations who received Molnupiravir treatment vs non-COVID populations.
