# Supplementary material for: Association Between COVID-19 Infection and Thyroid Cancer Development: A Retrospective Cohort Study Using the TriNetX Database
Source: Biomedicines. 2025 Aug 8;13(8):1933. doi: 10.3390/biomedicines13081933 (PMC12383963; doi:10.3390/biomedicines13081933)
Supplement: Supplementary file 1 [file biomedicines-13-01933-s001.zip › Supplementary File S5.pdf]

Supplementary File S5. Kaplan-Meier graph of thyroid cancer risk in different subpopulations following COVID-19.

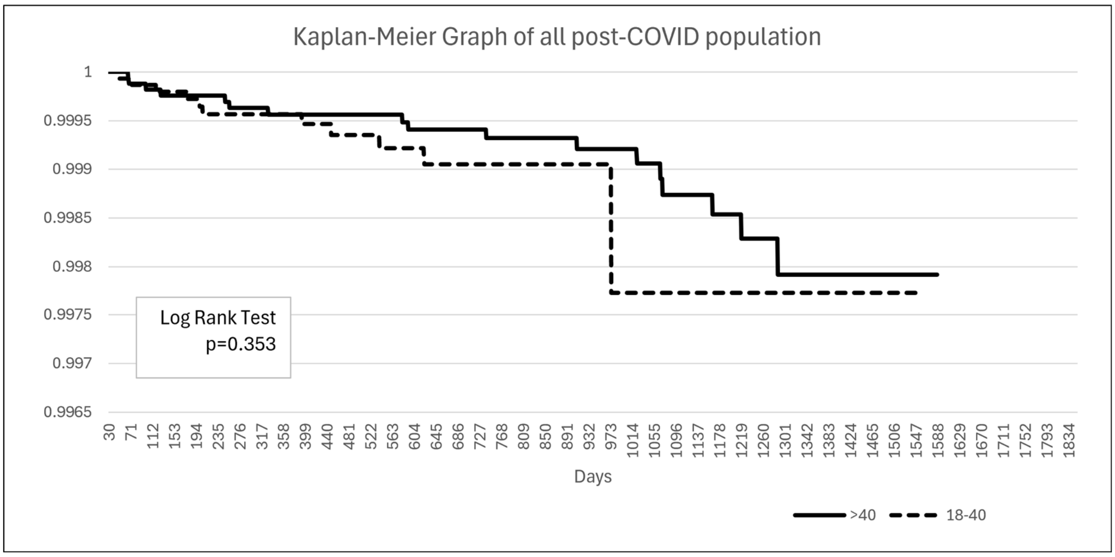

Supplementary Figure S5.1. Kaplan - Meier survival curve of thyroid cancer risk in different age interval of post-COVID populations.

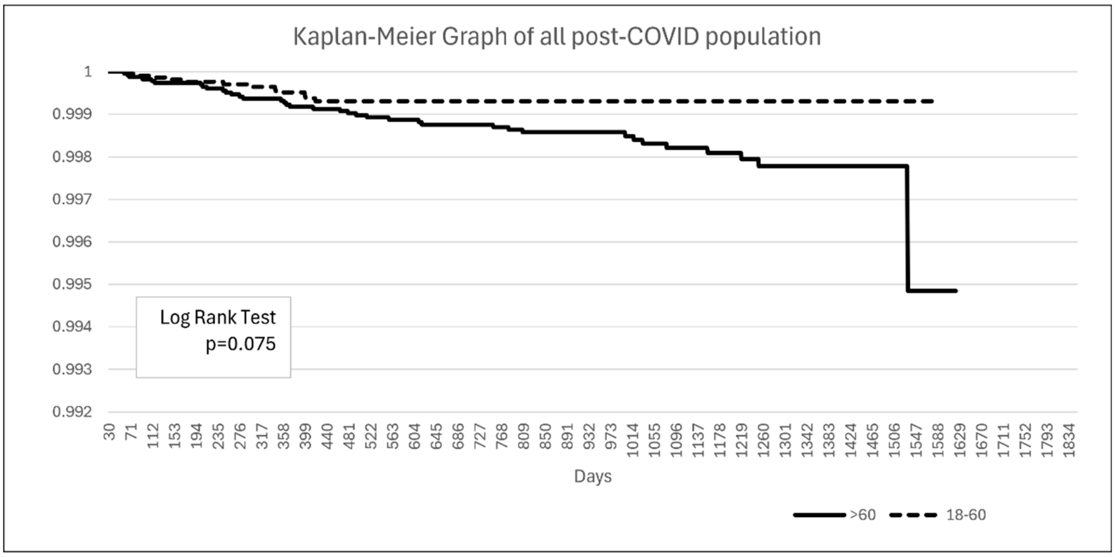

Supplementary Figure S5.2. Kaplan - Meier survival curve of thyroid cancer risk in different age interval of post-COVID populations.

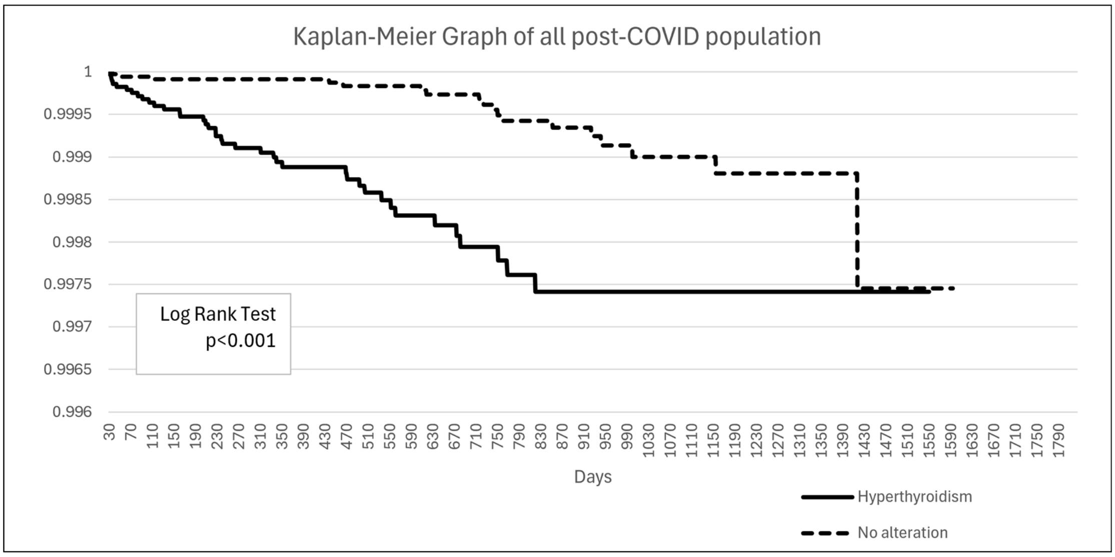

Supplementary Figure S5.3. Kaplan - Meier survival curve of thyroid cancer risk in post-COVID populations with hyperthyroidism vs no thyroid function alteration.

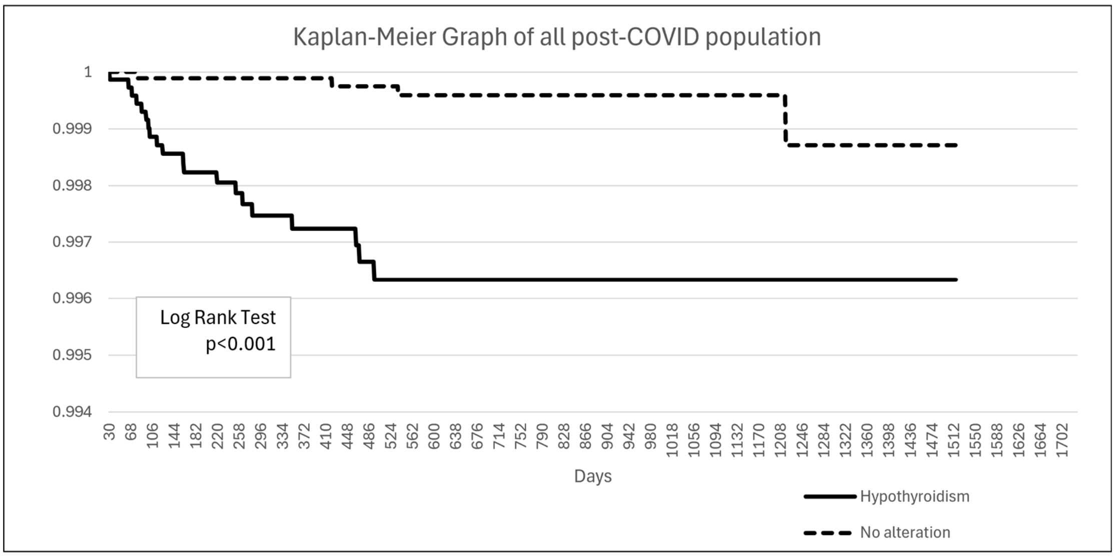

Supplementary Figure S5.4. Kaplan - Meier survival curve of thyroid cancer risk in post-COVID populations with hypothyroidism vs no thyroid function alteration.

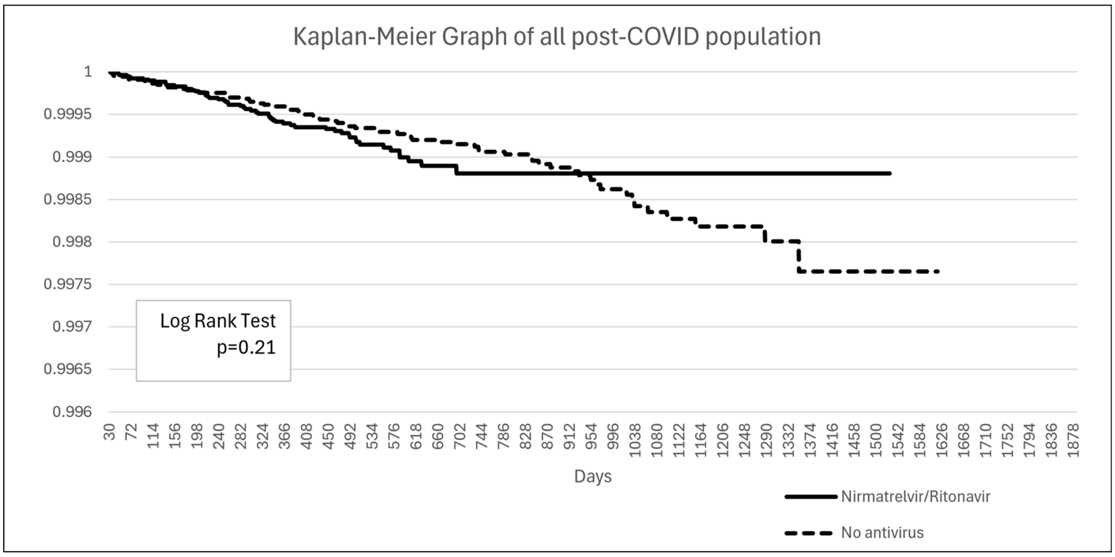

Supplementary Figure S5.5. Kaplan - Meier survival curve of thyroid cancer risk in post-COVID populations who received Nirmatrelvir/Ritonavir vs no antiviral treatment.

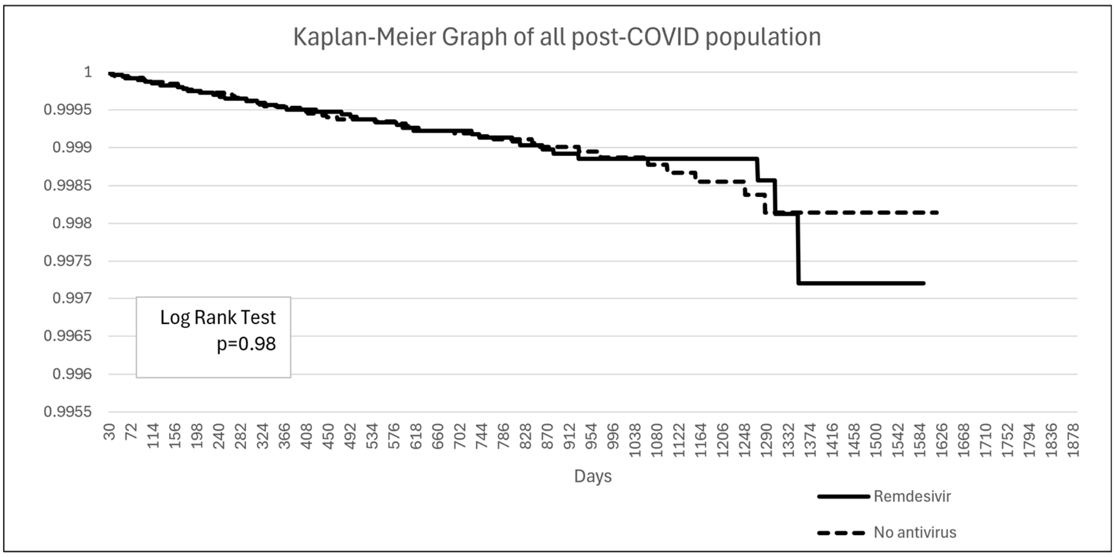

Supplementary Figure S5.6. Kaplan - Meier survival curve of thyroid cancer risk in post-COVID populations who received Remdesivir vs no antiviral treatment.

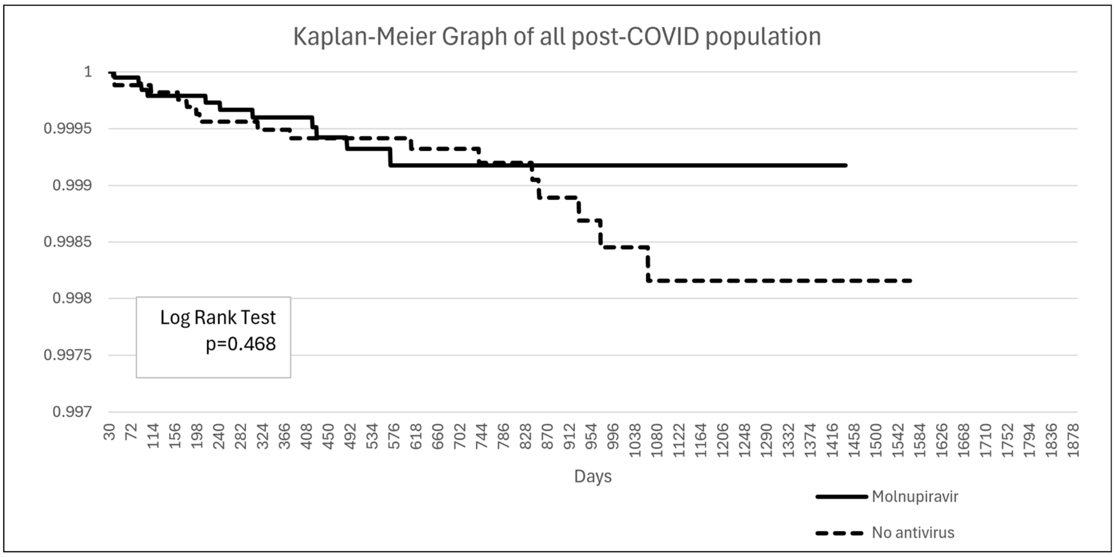

Supplementary Figure S5.7. Kaplan - Meier survival curve of thyroid cancer risk in post-COVID populations who received Molnupiravir vs no antiviral treatment.
