# Supplementary material for: Association Between COVID-19 Infection and Thyroid Cancer Development: A Retrospective Cohort Study Using the TriNetX Database
Source: Biomedicines. 2025 Aug 8;13(8):1933. doi: 10.3390/biomedicines13081933 (PMC12383963; doi:10.3390/biomedicines13081933)
Supplement: Supplementary file 1 [file biomedicines-13-01933-s001.zip › Supplementary File S6.pdf]

Supplementary File S6. Kaplan–Meier curve illustrating the risk of thyroid cancer in subpopulations with thyroid dysfunction, stratified by COVID-19 status.

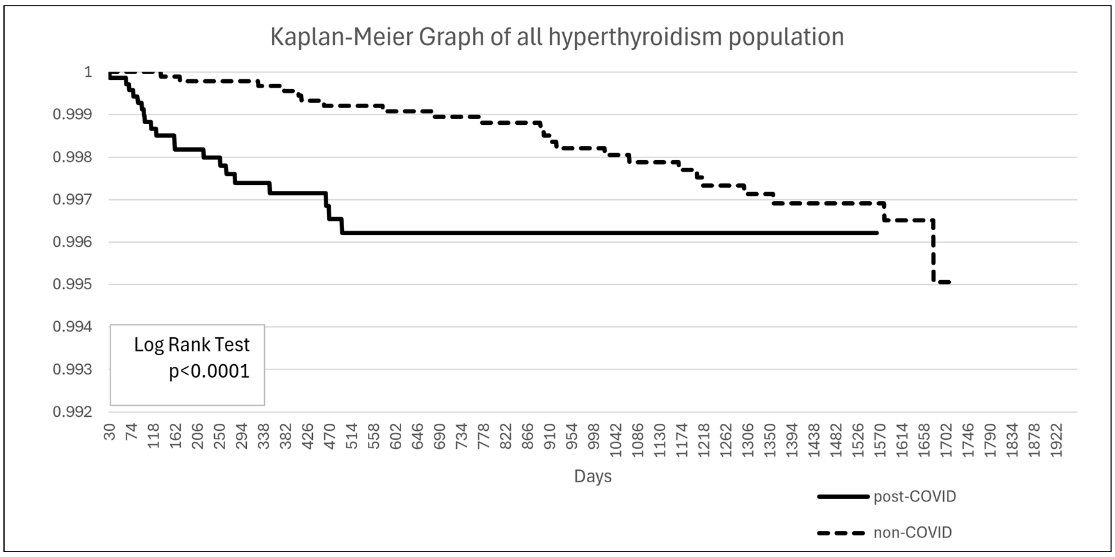

Supplementary Figure S6.1. Kaplan - Meier survival curve of thyroid cancer risk in hyperthyroidism populations with or without COVID-19.

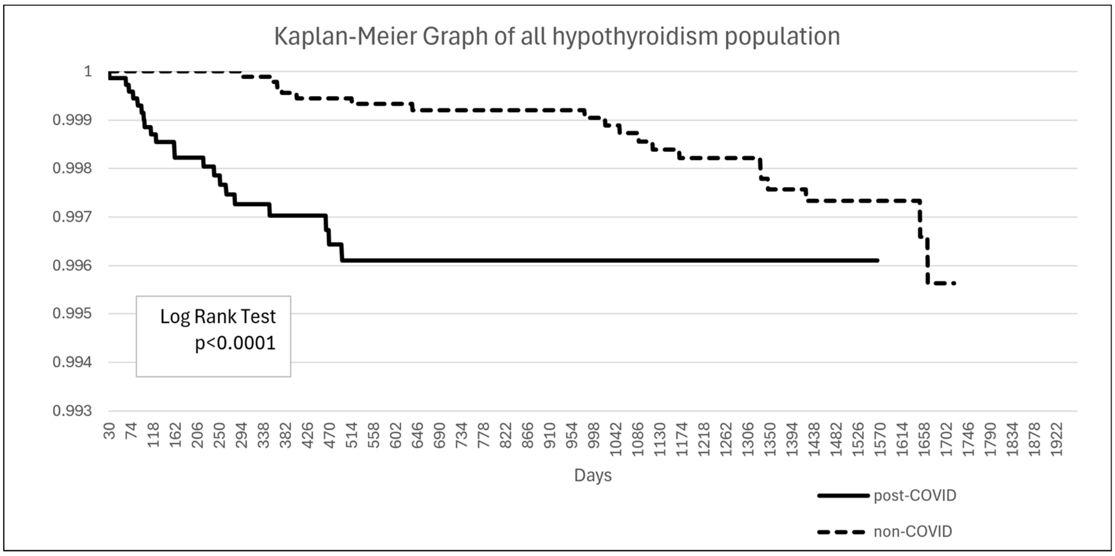

Supplementary Figure S6.2. Kaplan - Meier survival curve of thyroid cancer risk in hypothyroidism populations with or without COVID-19.
